# Supplementary material for: Quantitative cross-linking/mass spectrometry reveals subtle protein conformational changes
Source: Wellcome Open Res. 2016 Nov 15;1:5. [Version 1] doi: 10.12688/wellcomeopenres.9896.1 (PMC5140025; doi:10.12688/wellcomeopenres.9896.1)
Supplement: Supplementary file 3 [file wellcomeopenres-1-10667-s0002.tgz › 652ea684-1241-48d2-97c3-337b922ab4d7.pdf]

General Spectral Library Format for Pinpoint Comma Separated Values (Thermo Fisher Scientific)

File extension is .txt

Modifications

The first few lines contain the index of static and dynamic modifications, with the information being separated by comma. If there are no modifications, these lines can be complete skipped

Format:

static,sites,delta_mass

dynamic,index,sites,delta_mass

index shows the number used to represent that mod

sites should be uppercase amino acids ”ARNDCEQGHILKMFPSTWYV”, or n or c, for terminal mods.

delta_mass is a decimal number (16, 15.99, 15.99999999 … are all fine)

Peptide info

Sequence, charge_state,retention_time,modifications,library_intensity,protein

mz,intensity, mz,intensity, mz,intensity, mz,intensity,mz,intensity, mz,intensity,

All this information must be there, e.g., in the following line, an empty area is left for the retention time and library intensity

FSISWAR,2,,000000000,,Apolipoprotein B

Sequence: Only valid amino acids allowed. Uppercase is a must.

Charge state: valid integral numbers (1 to 100)

Retention time: valid decimal number representing elution time in minutes

Modification: 1st digit denotes N-term, 2nd digit denotes C-term followed by every site

Library intensity: decimal number representing some ranking scheme (e.g., MS1 intensity)

Protein: all the remaining information is taken to be the protein name

mz-intensity pairs: mass-sorted MSMS spectra, each valued separated by comma (not used in cross-linking quantitation, space is hold using “0,0”).
