# Supplementary figures and images for: Quantitative cross-linking/mass spectrometry reveals subtle protein conformational changes
Source: Wellcome Open Res. 2016 Nov 15;1:5. [Version 1] doi: 10.12688/wellcomeopenres.9896.1 (PMC5140025; doi:10.12688/wellcomeopenres.9896.1)

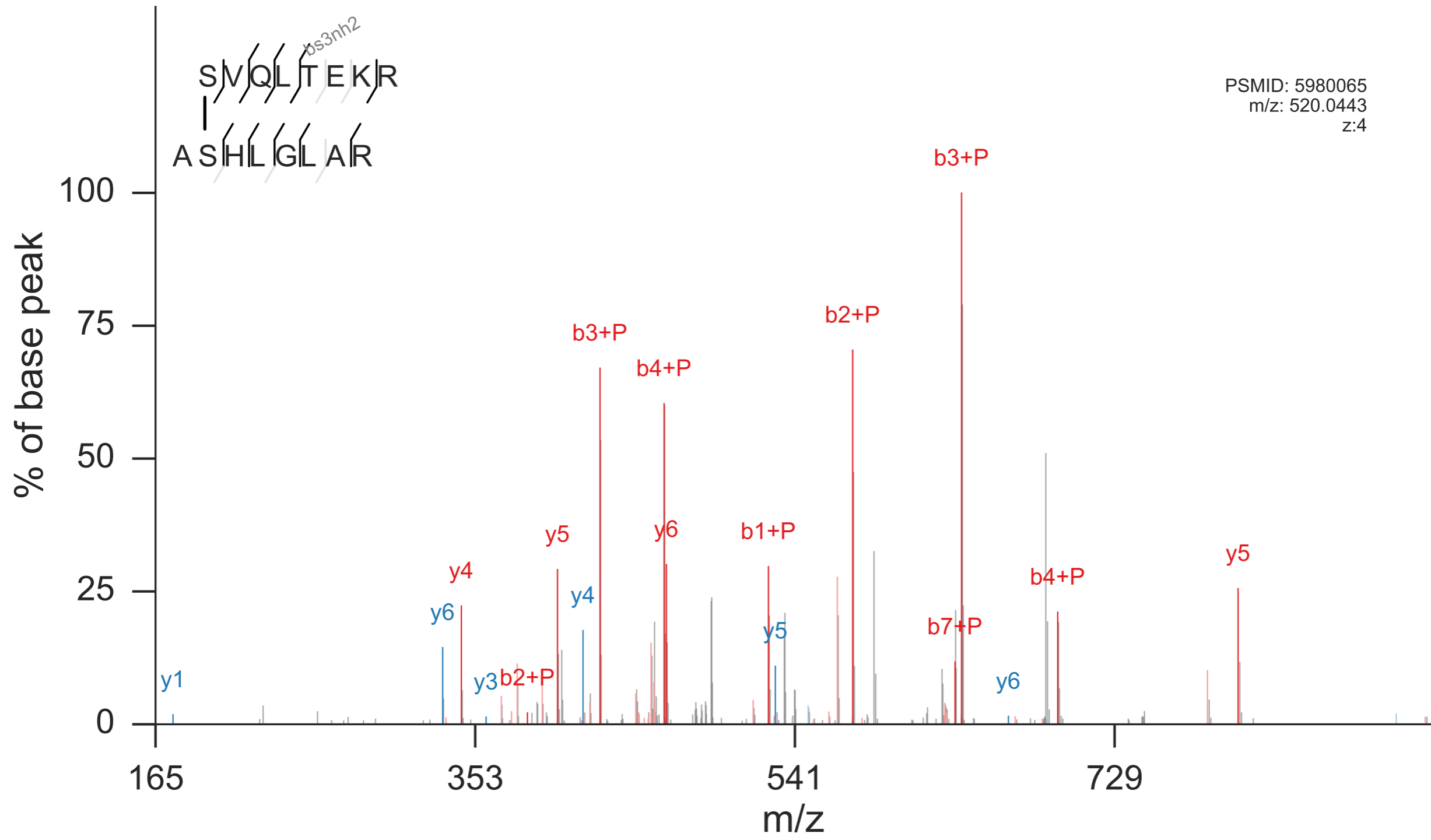

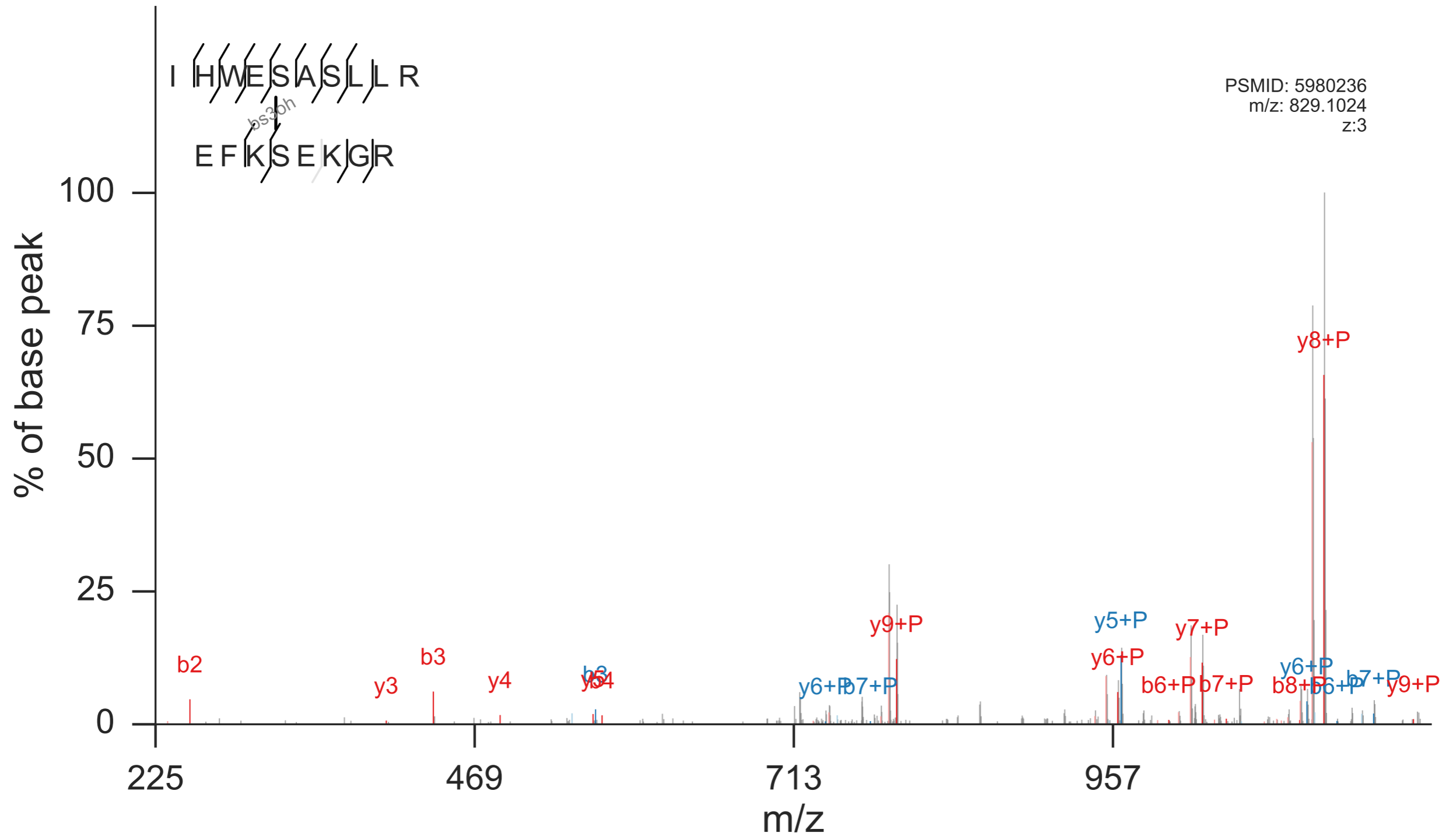

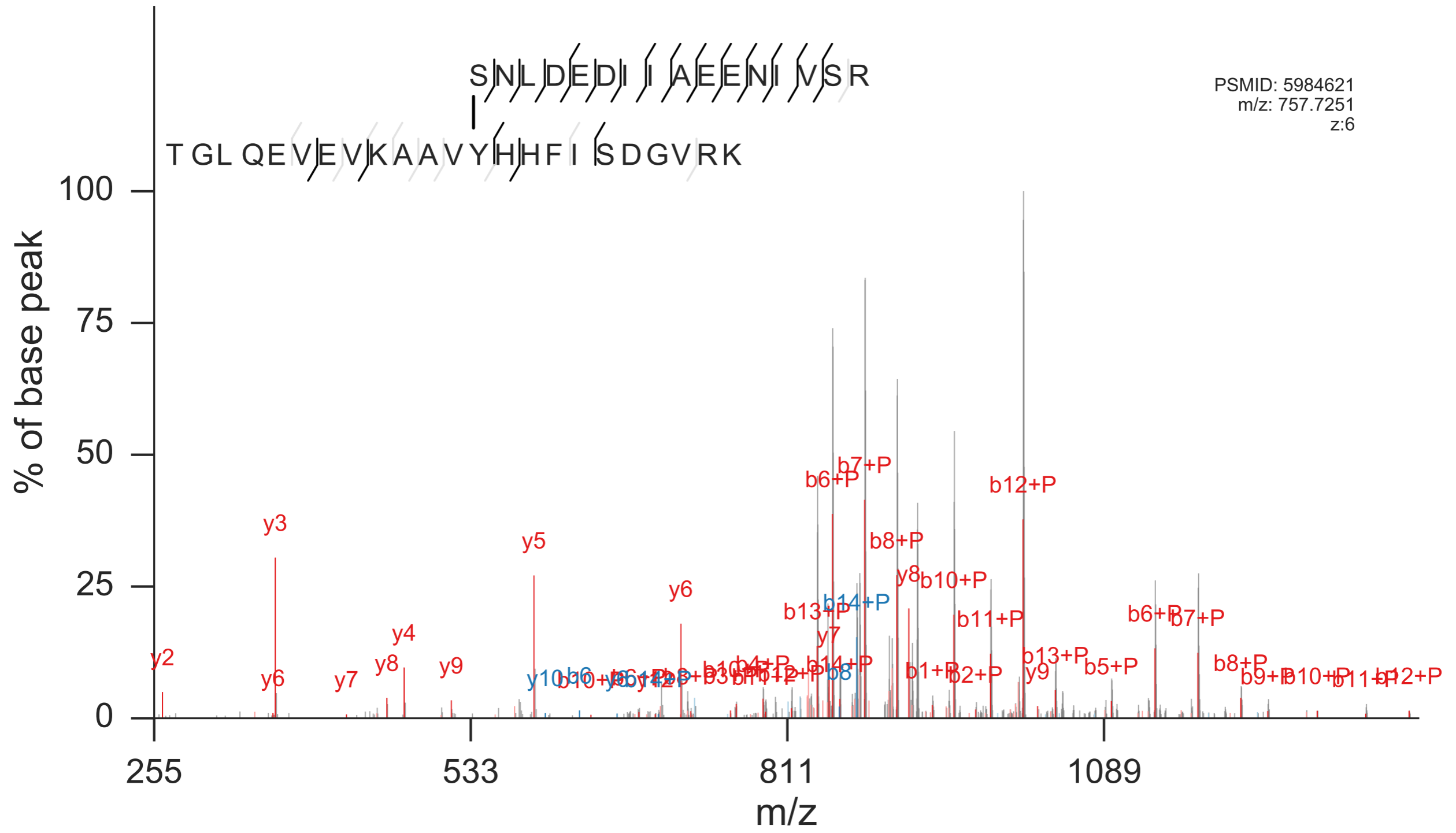

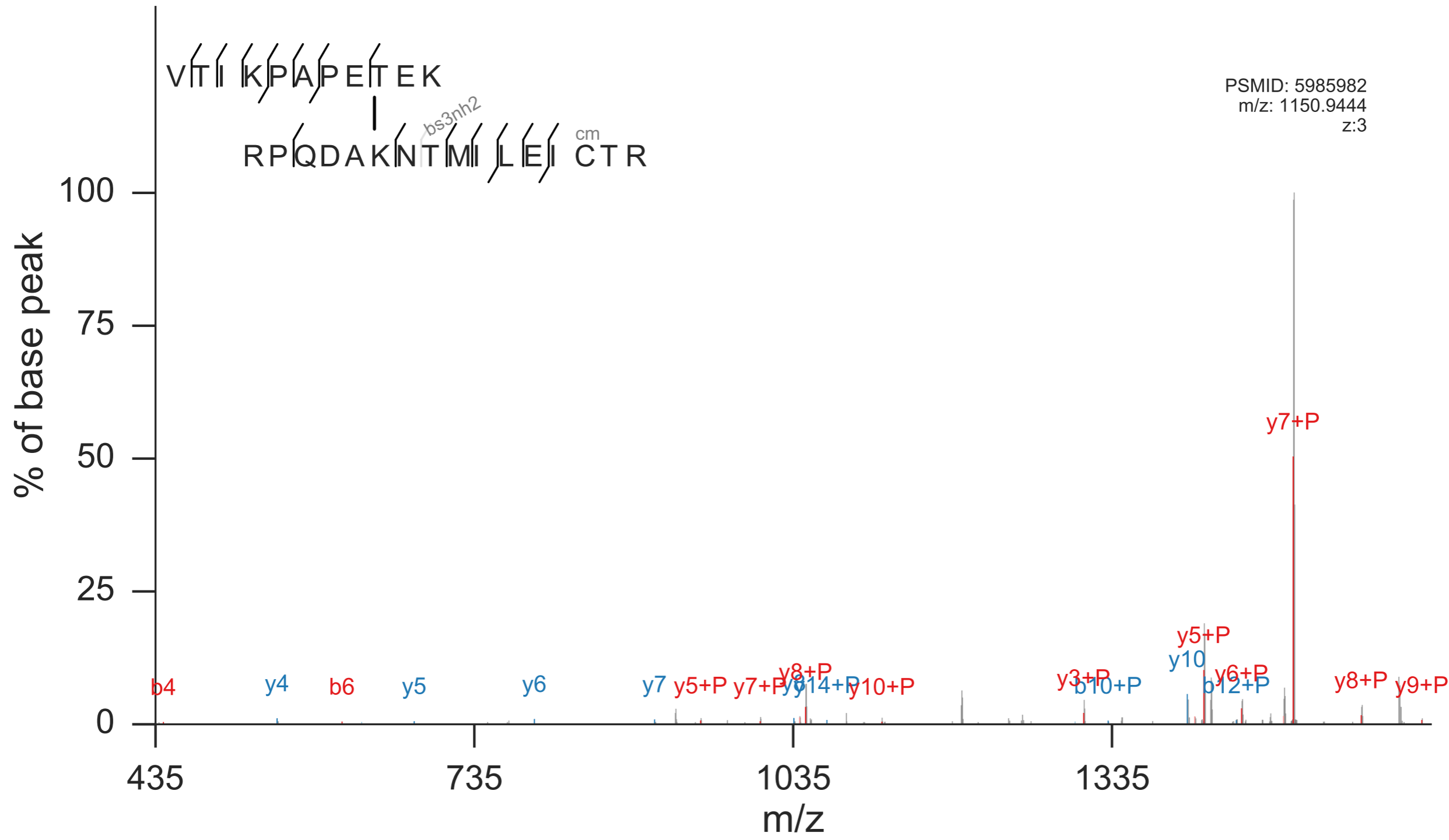

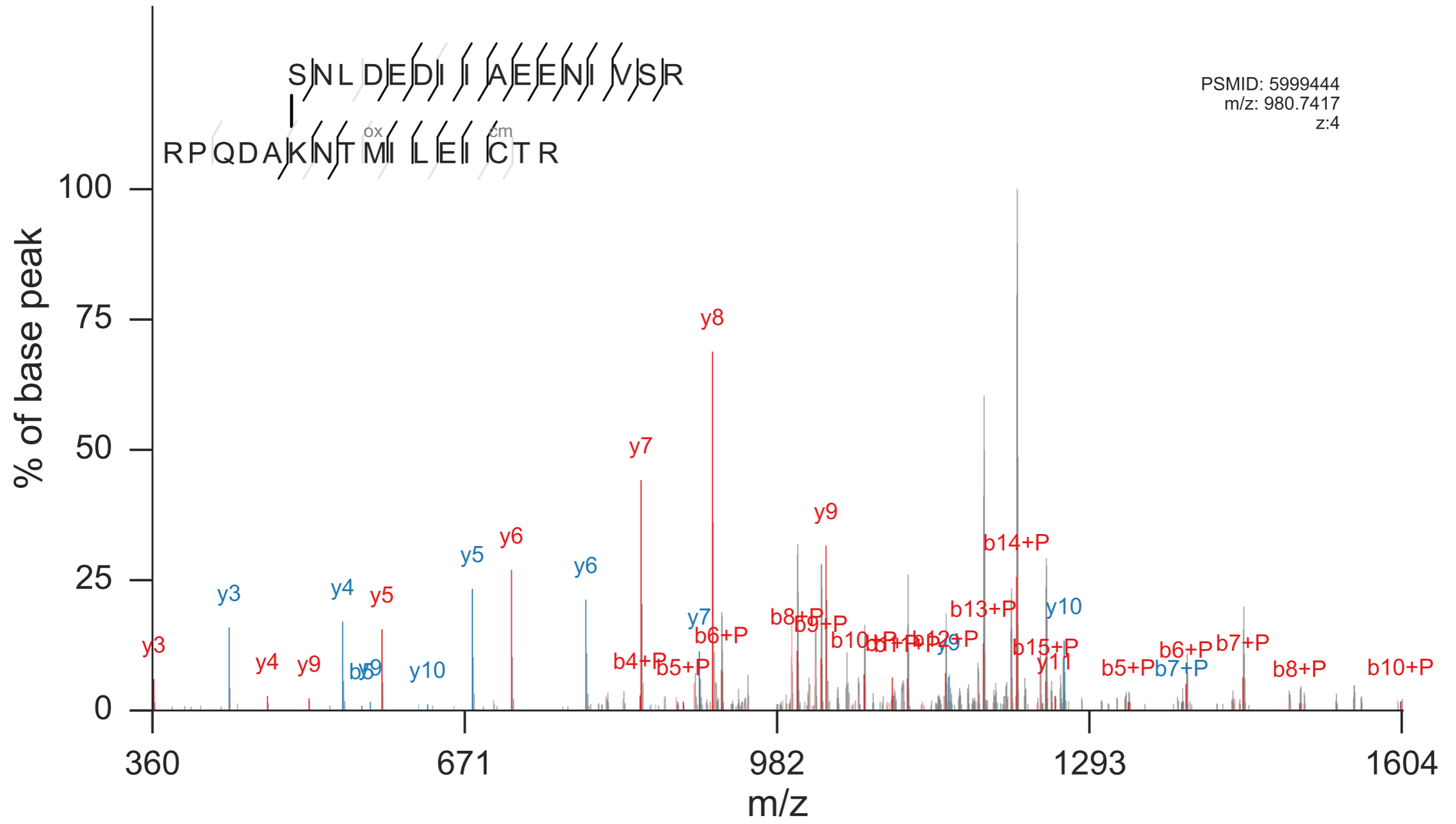

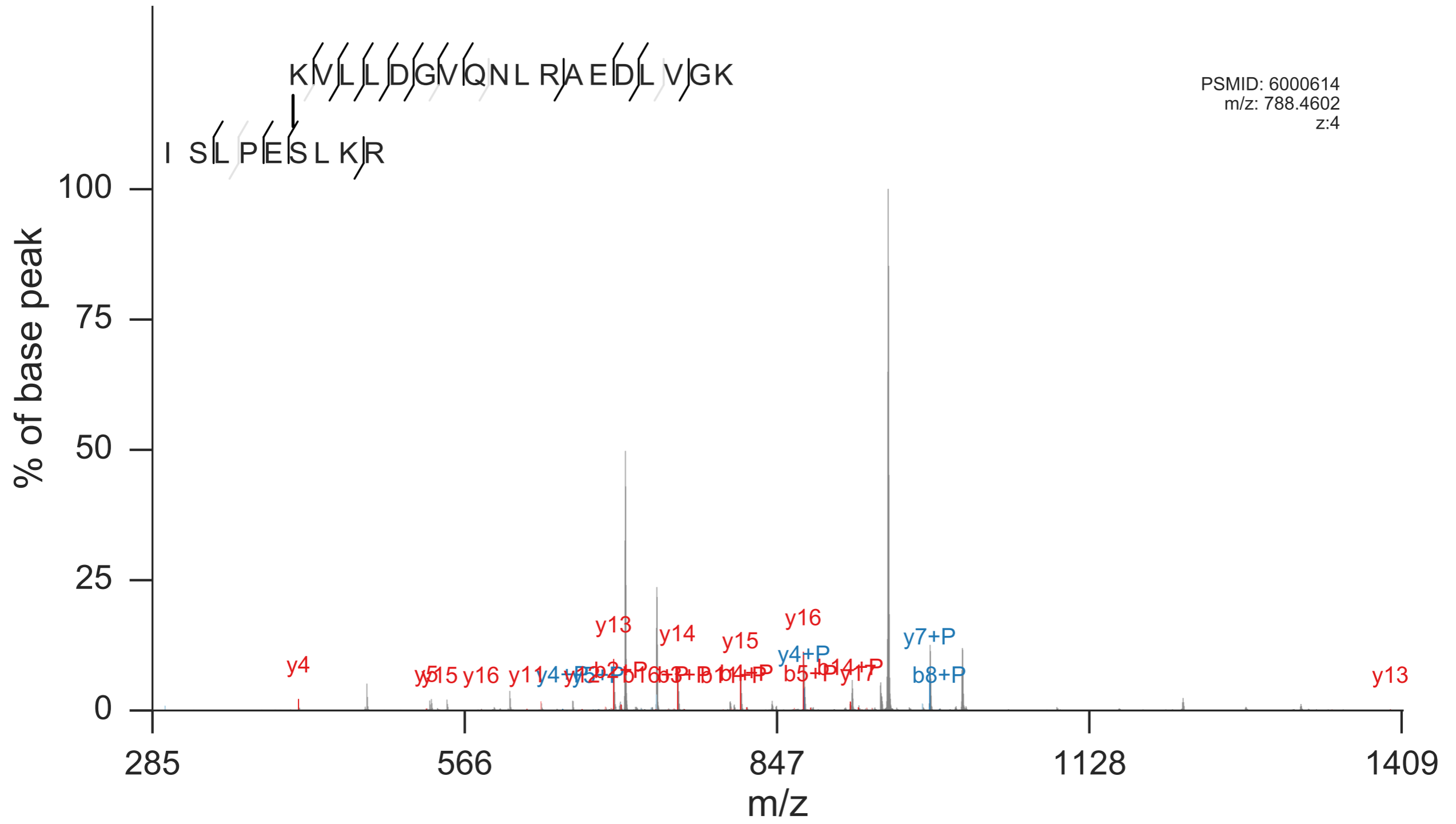

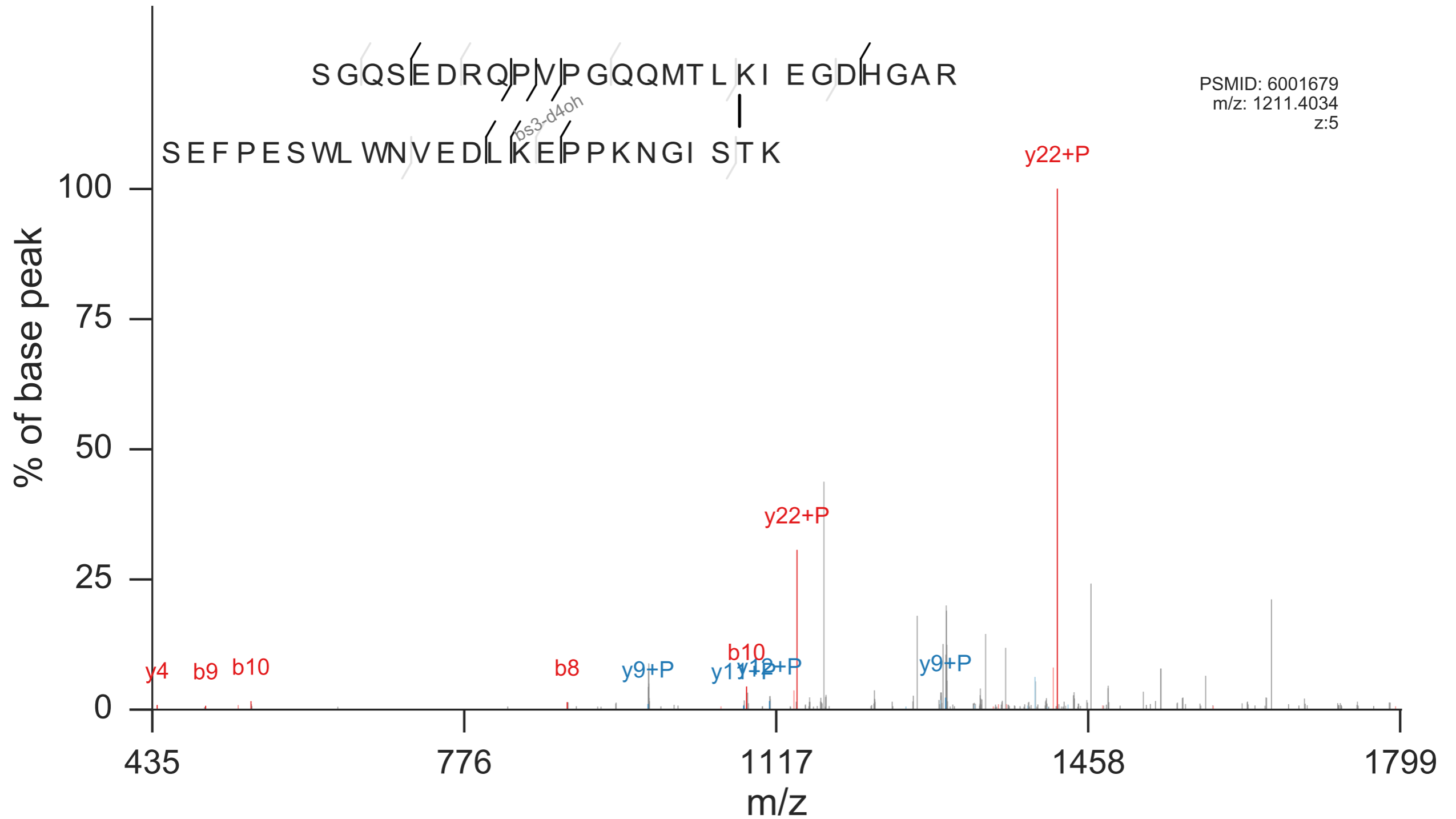

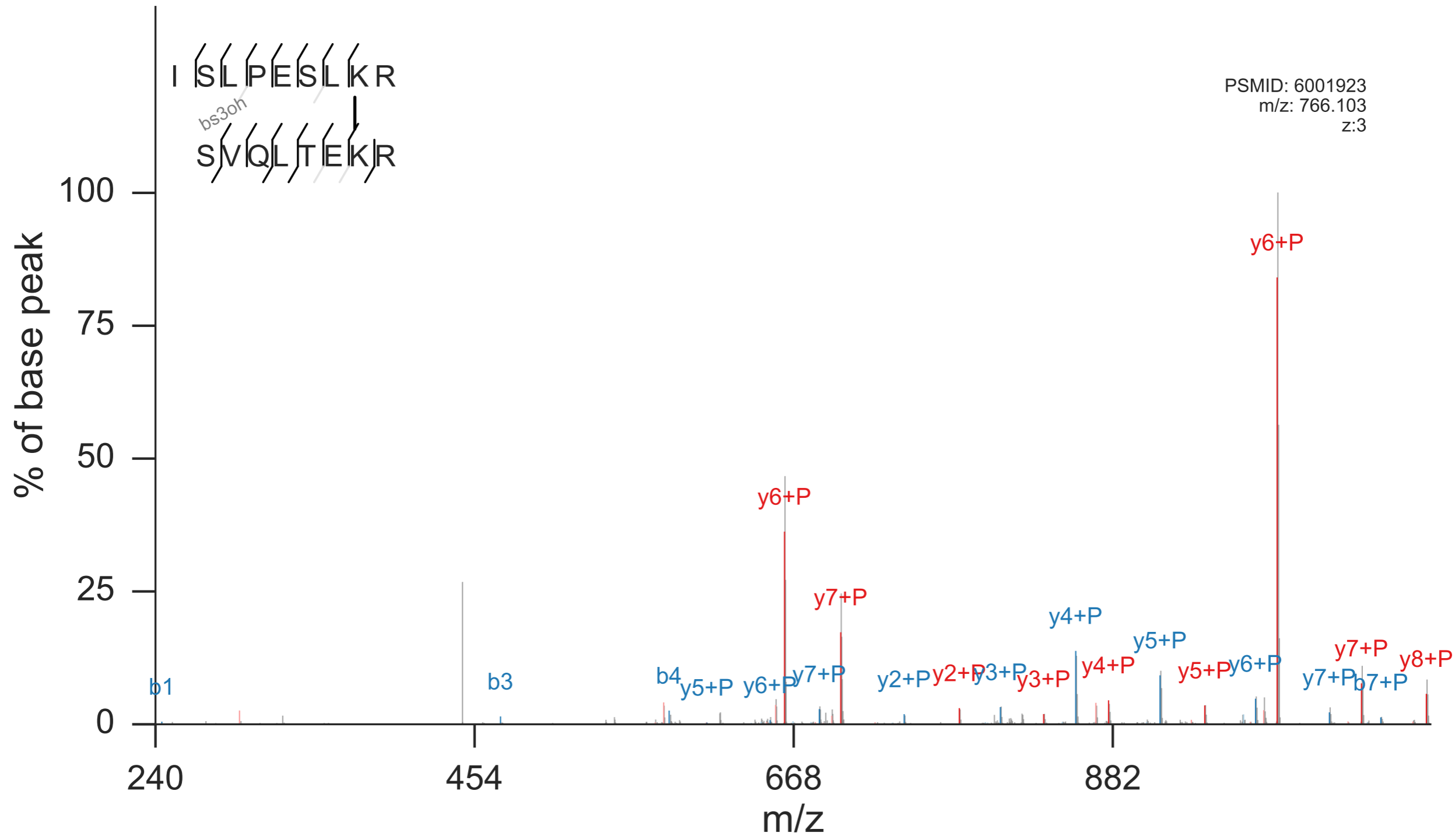

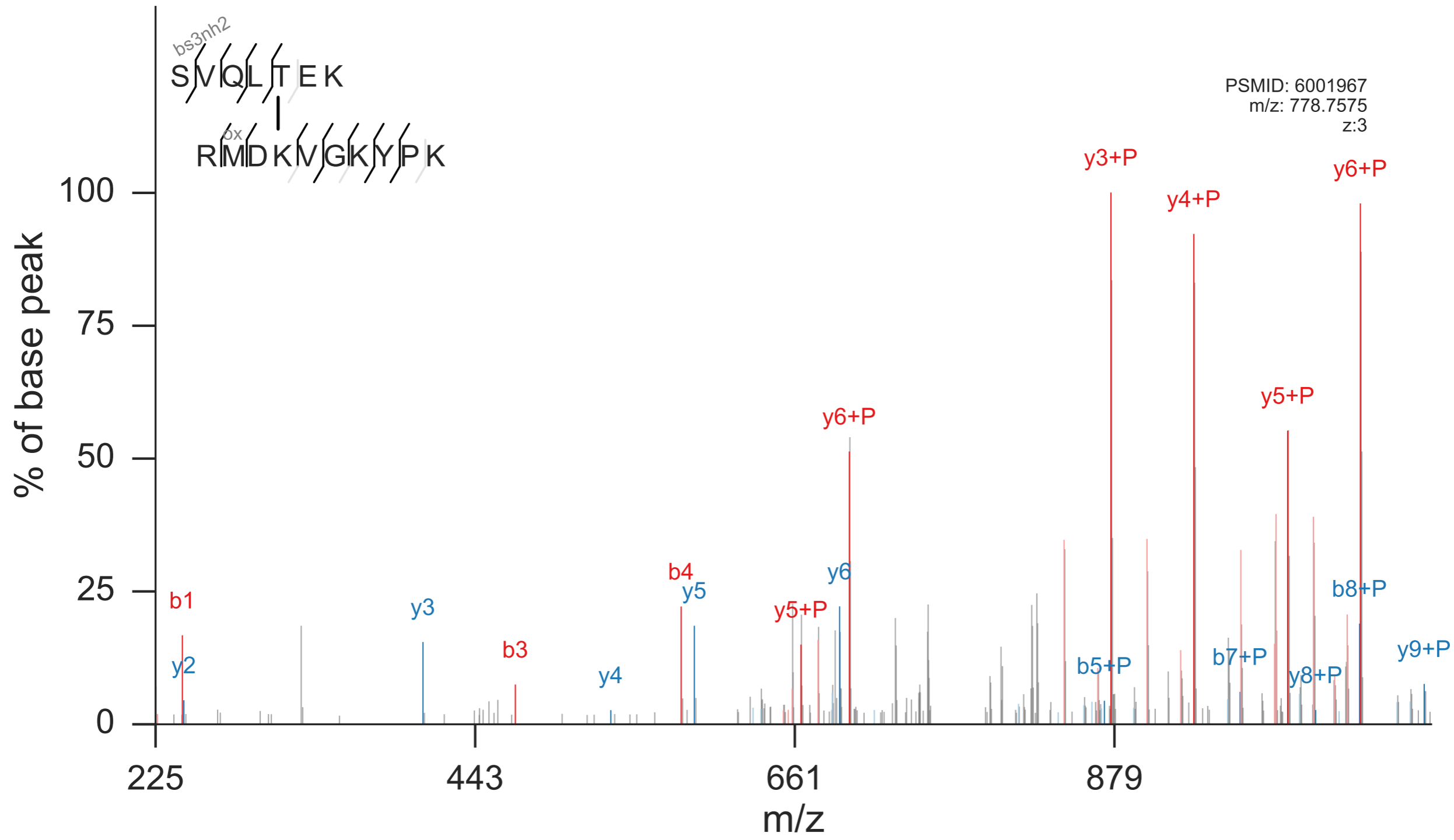

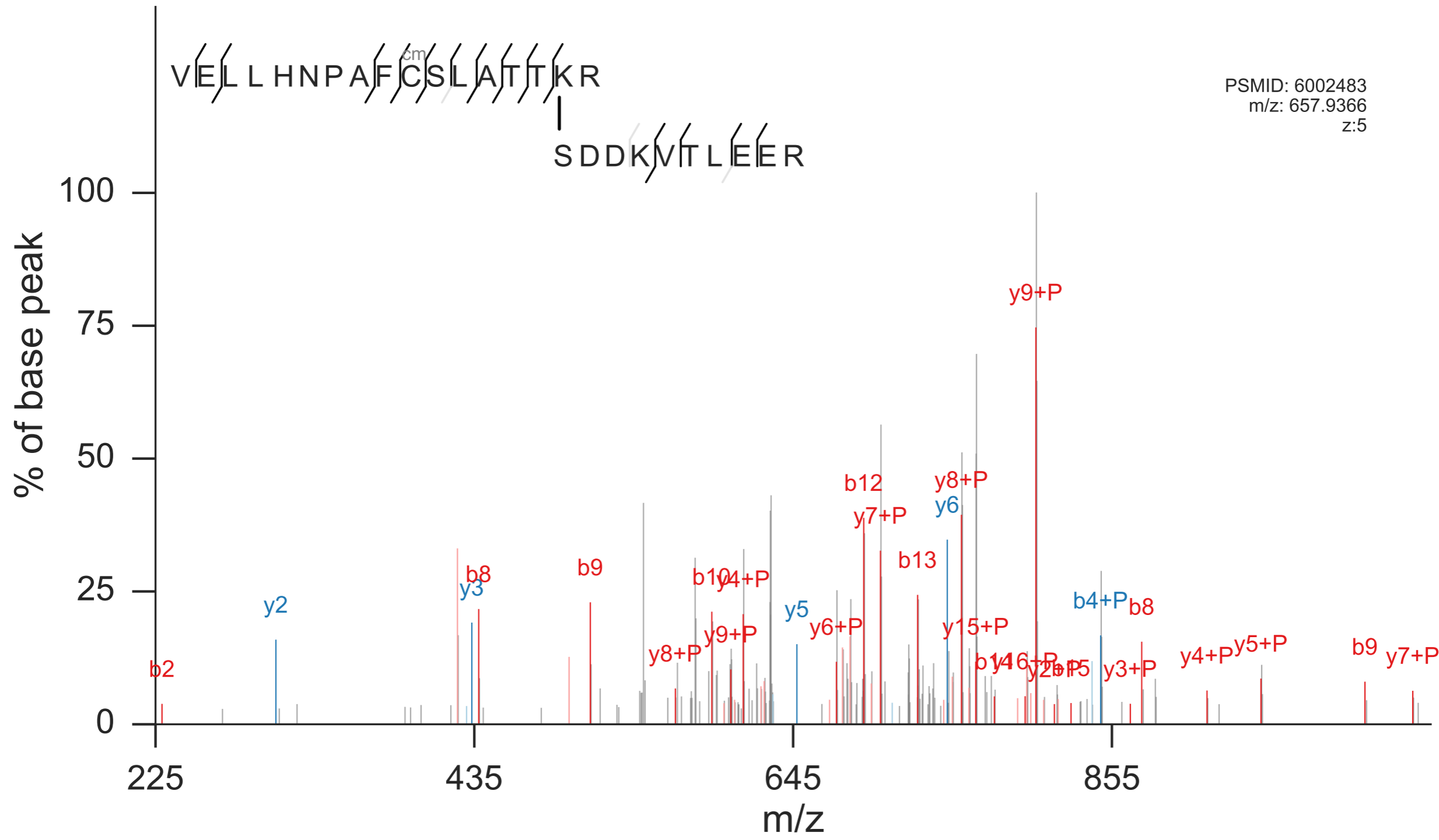

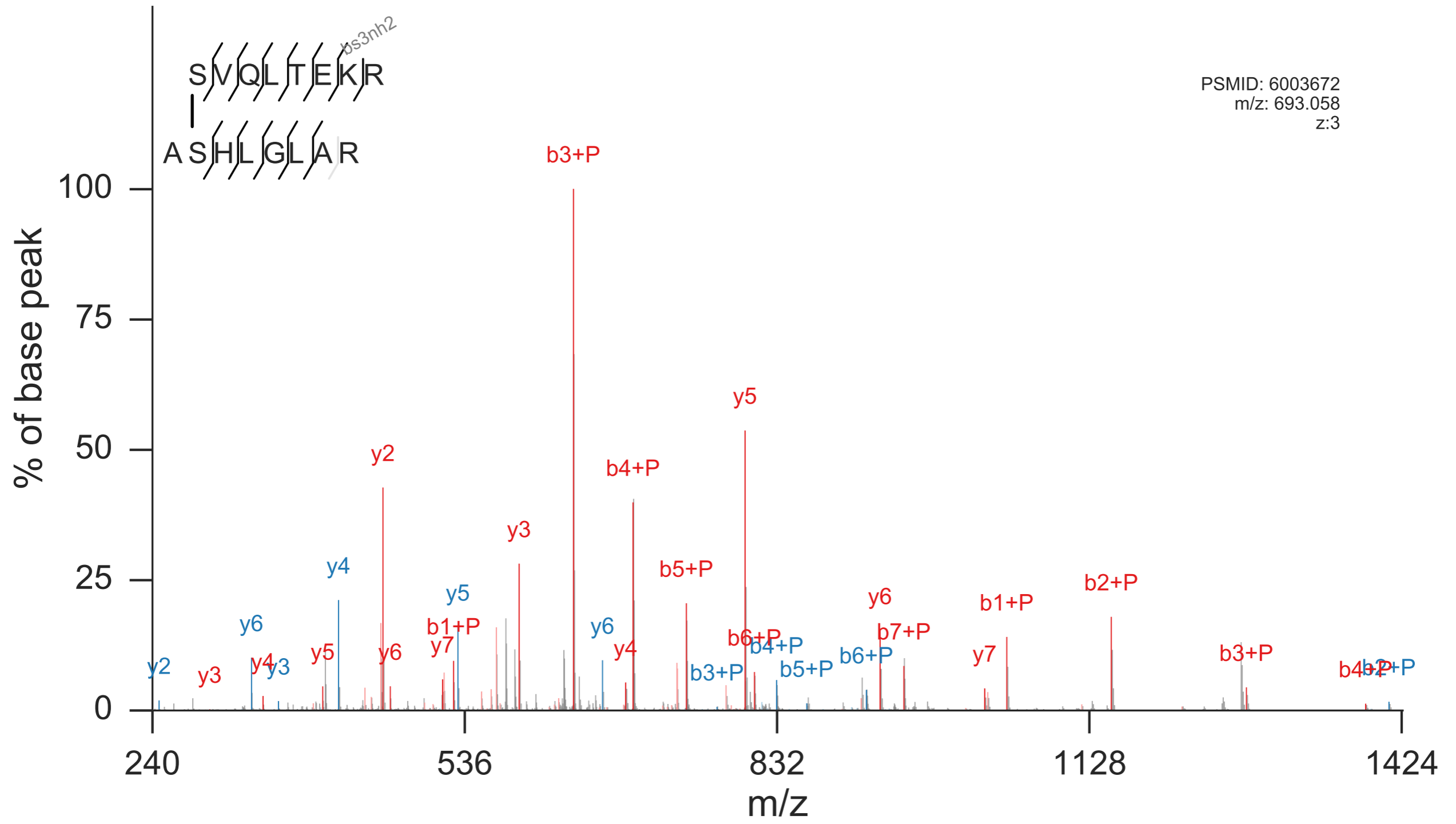

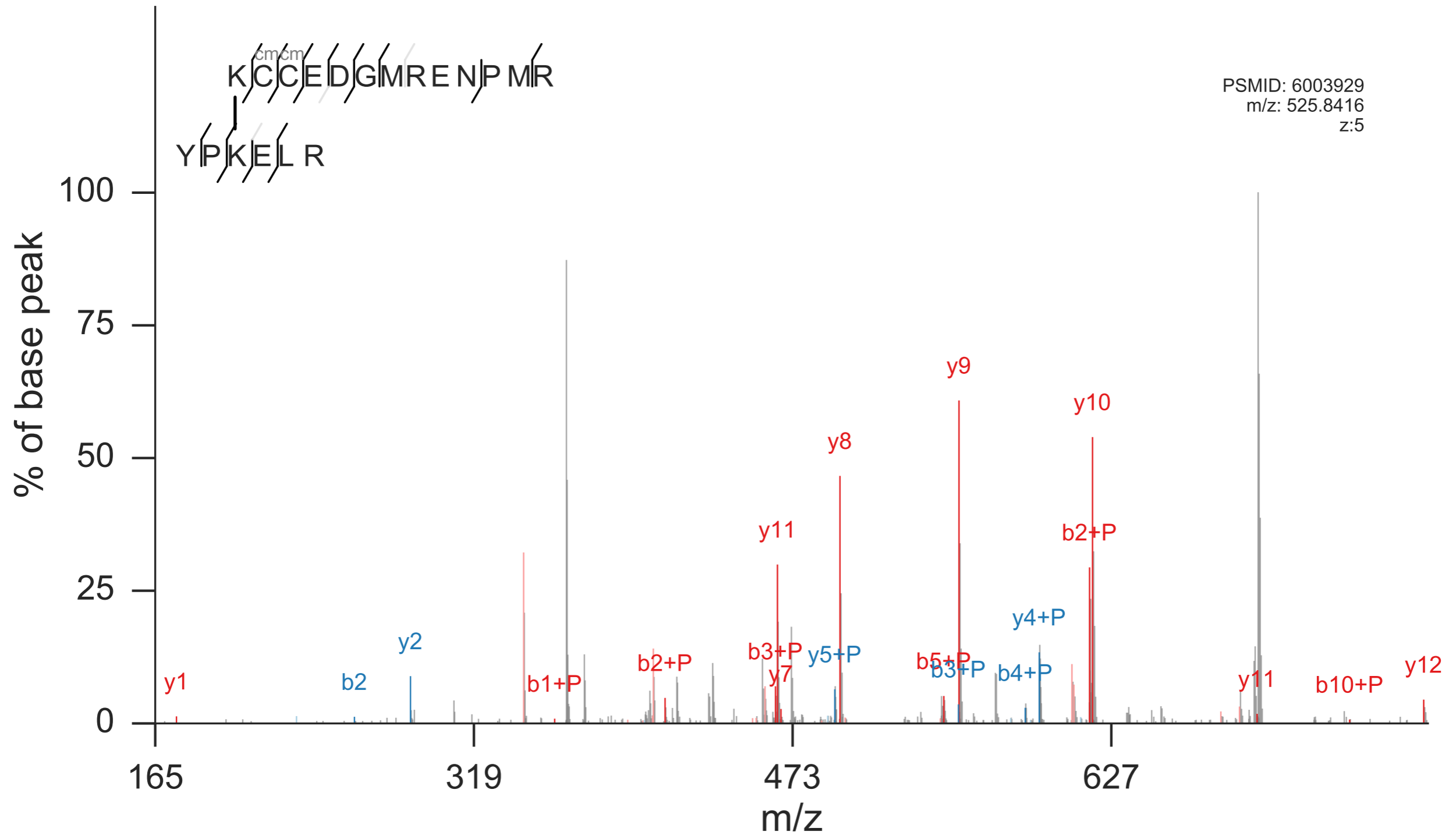

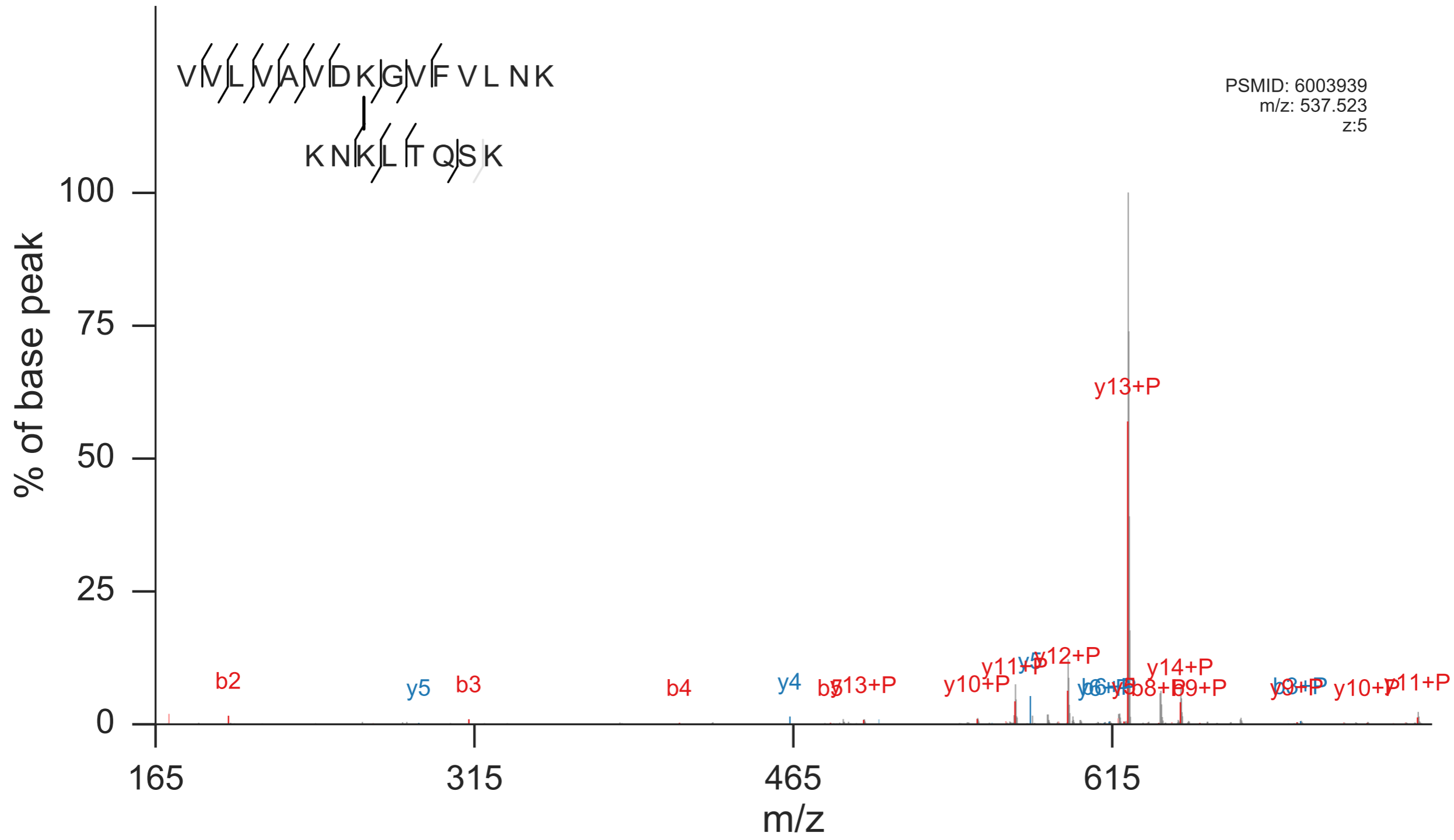

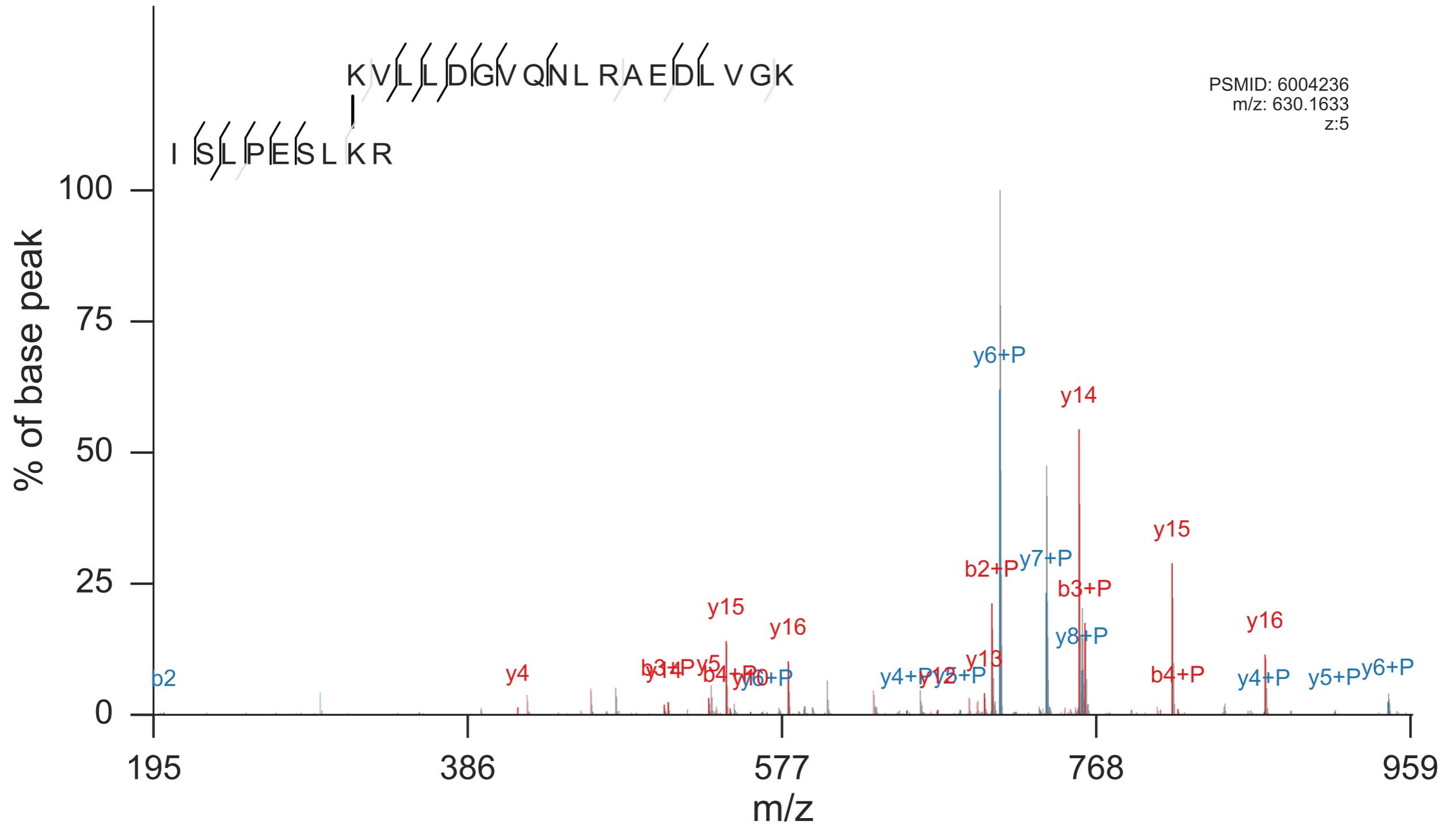

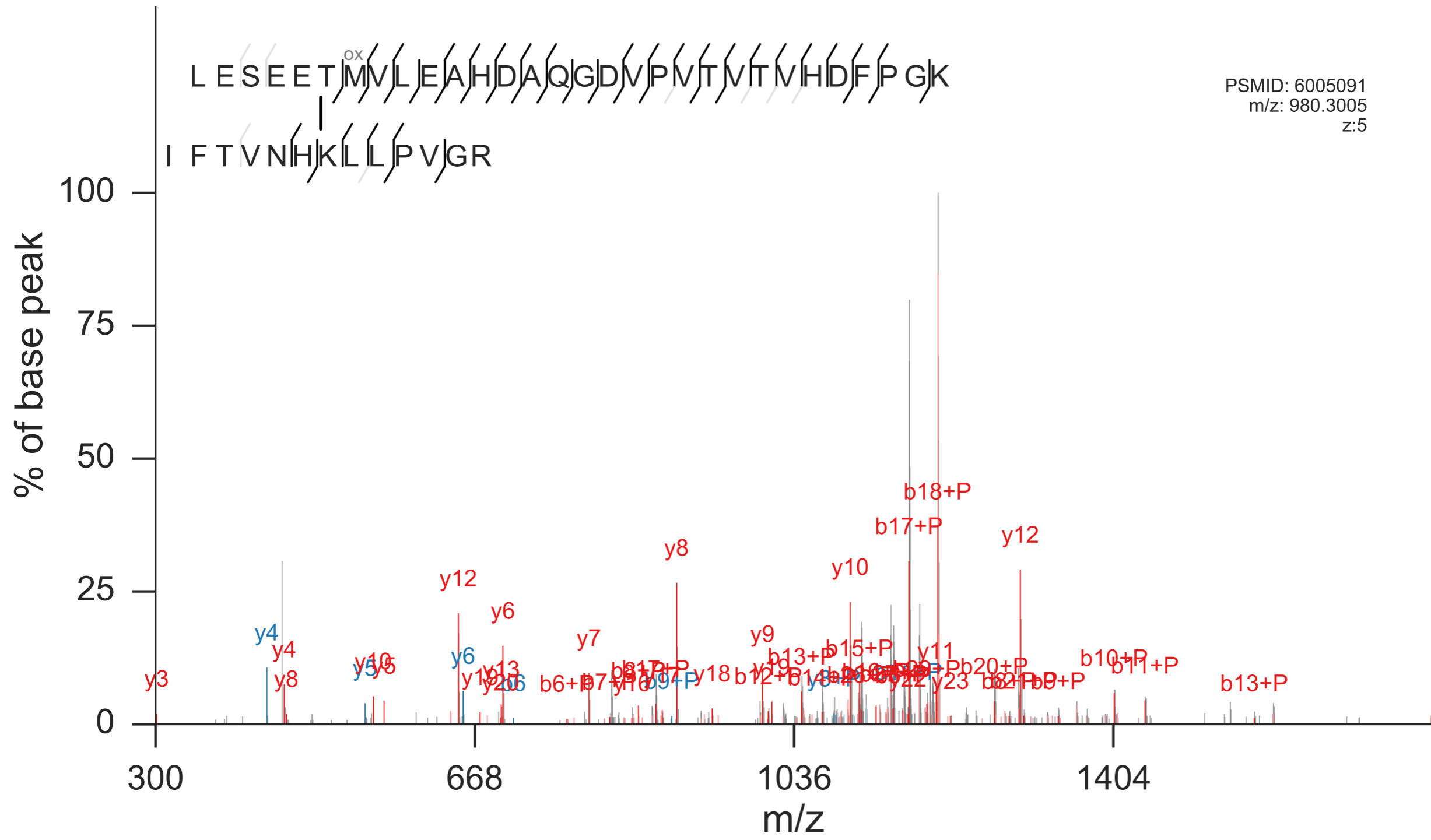

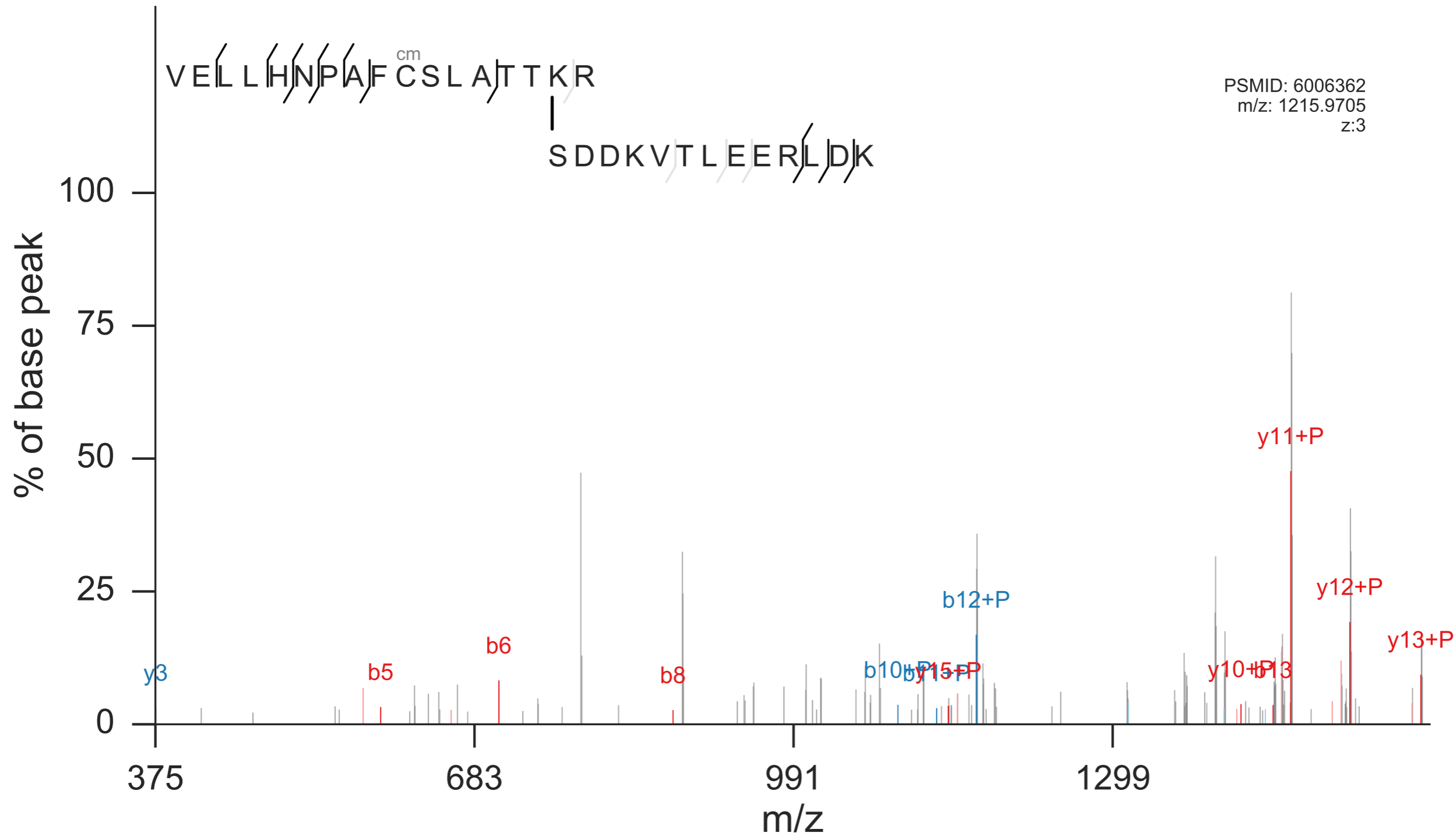

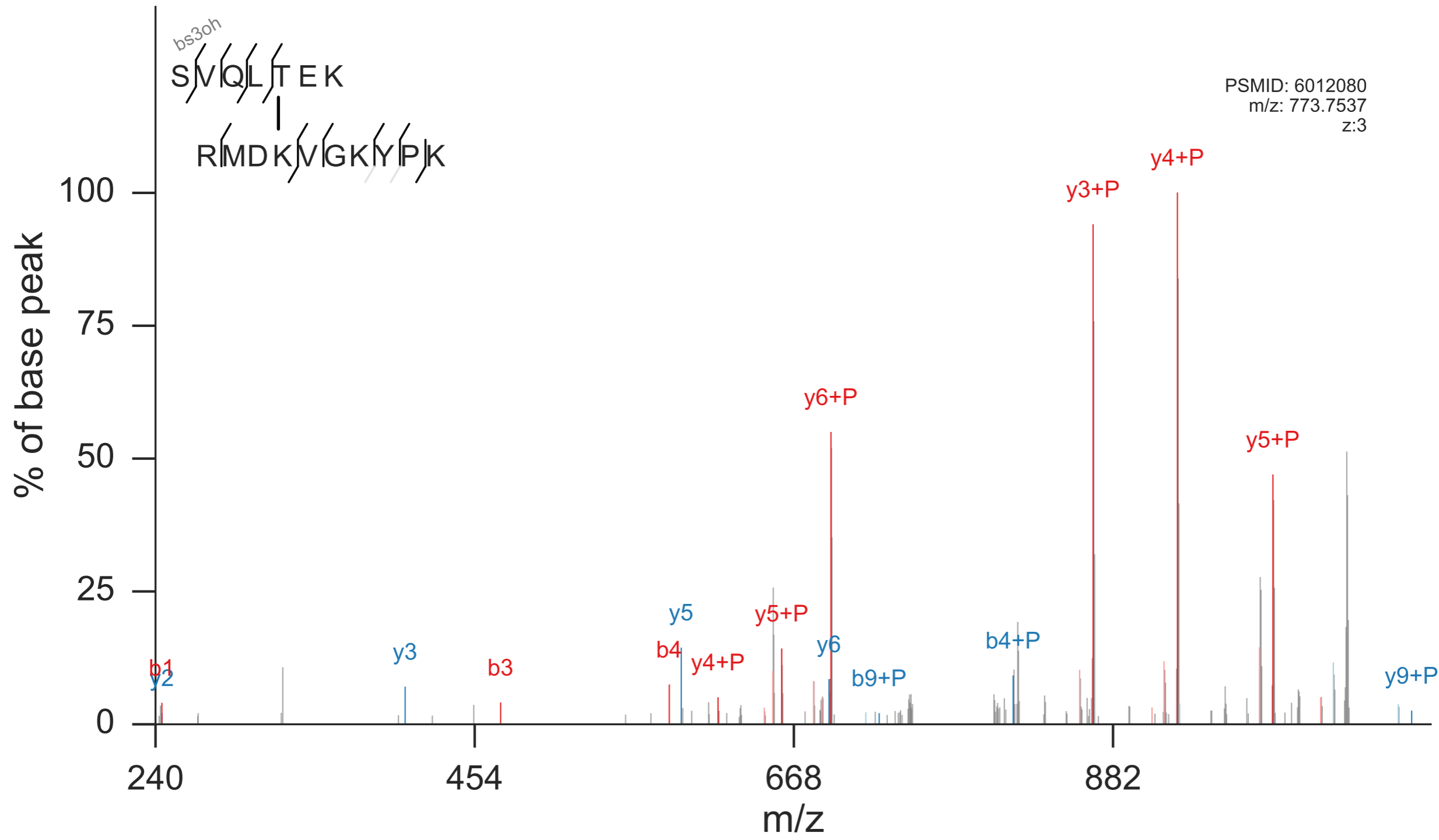

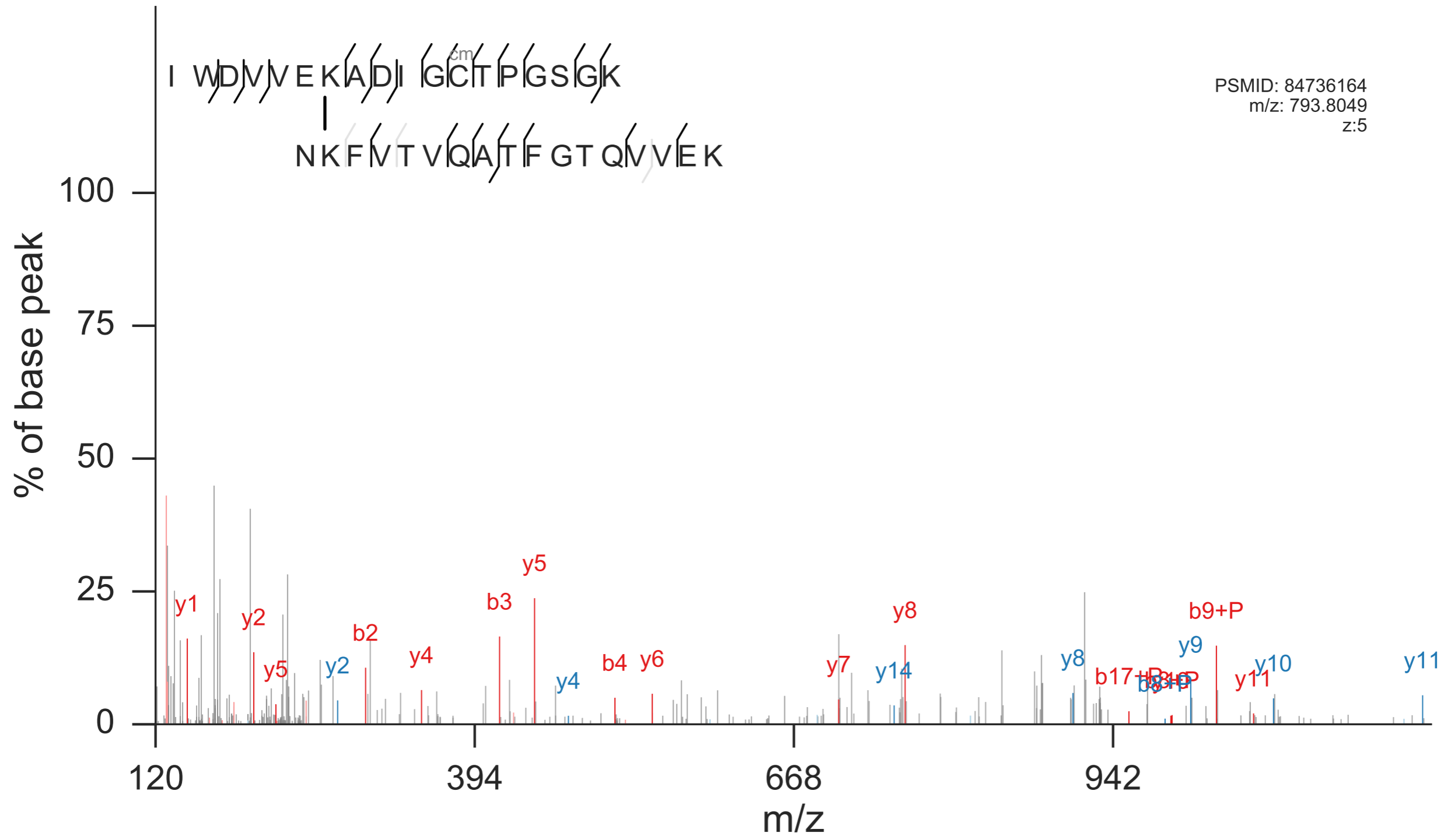

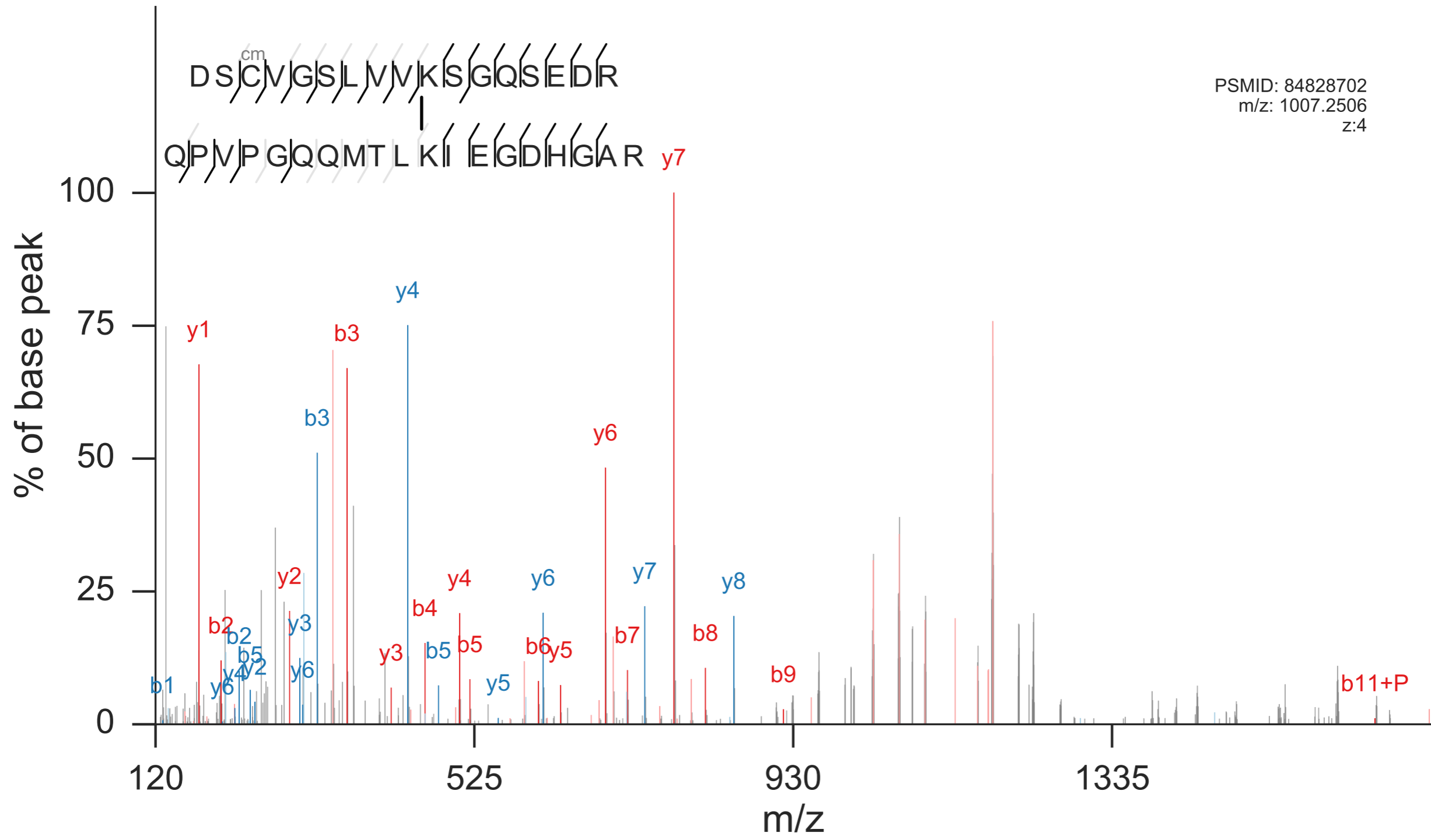

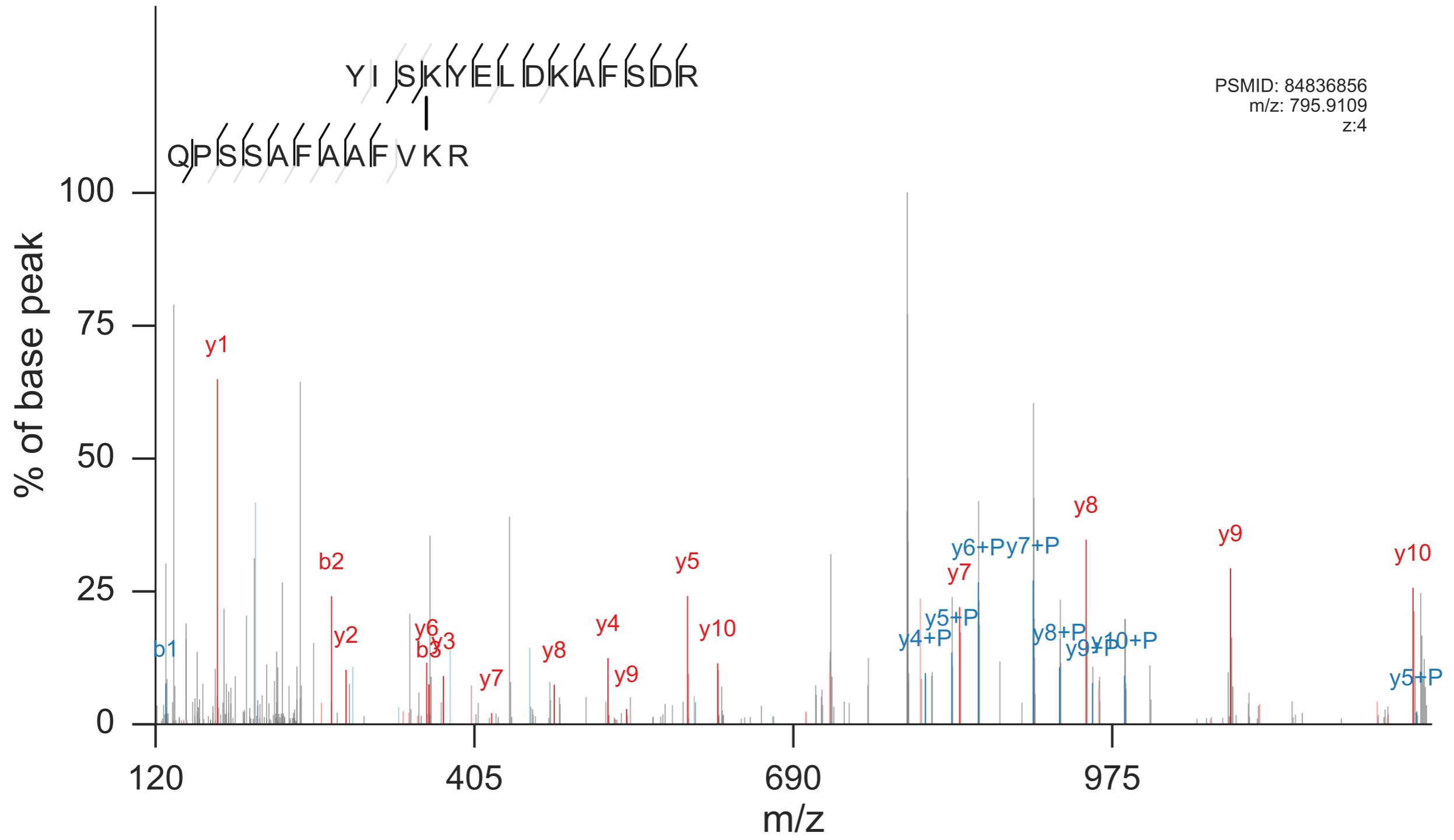

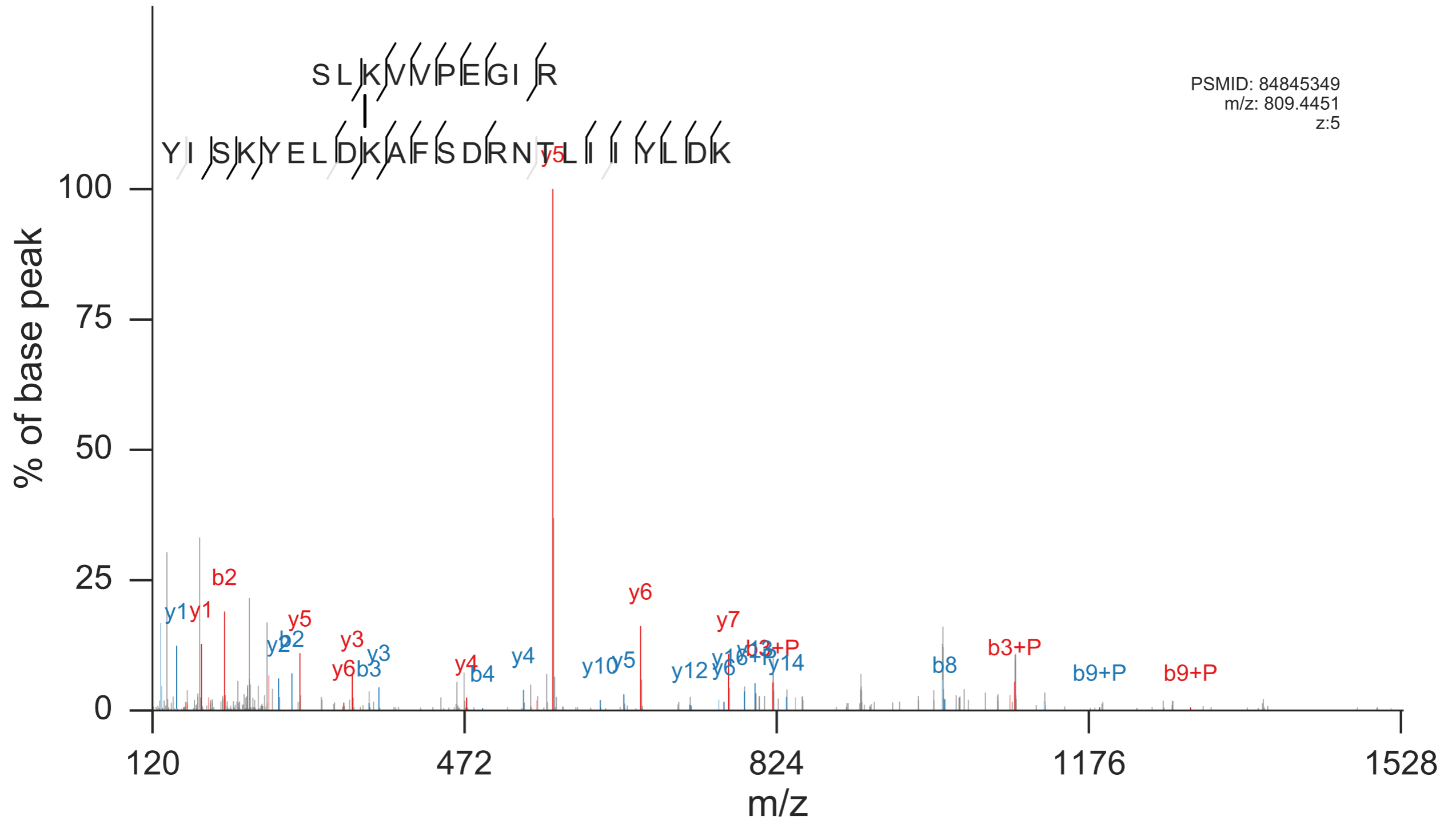

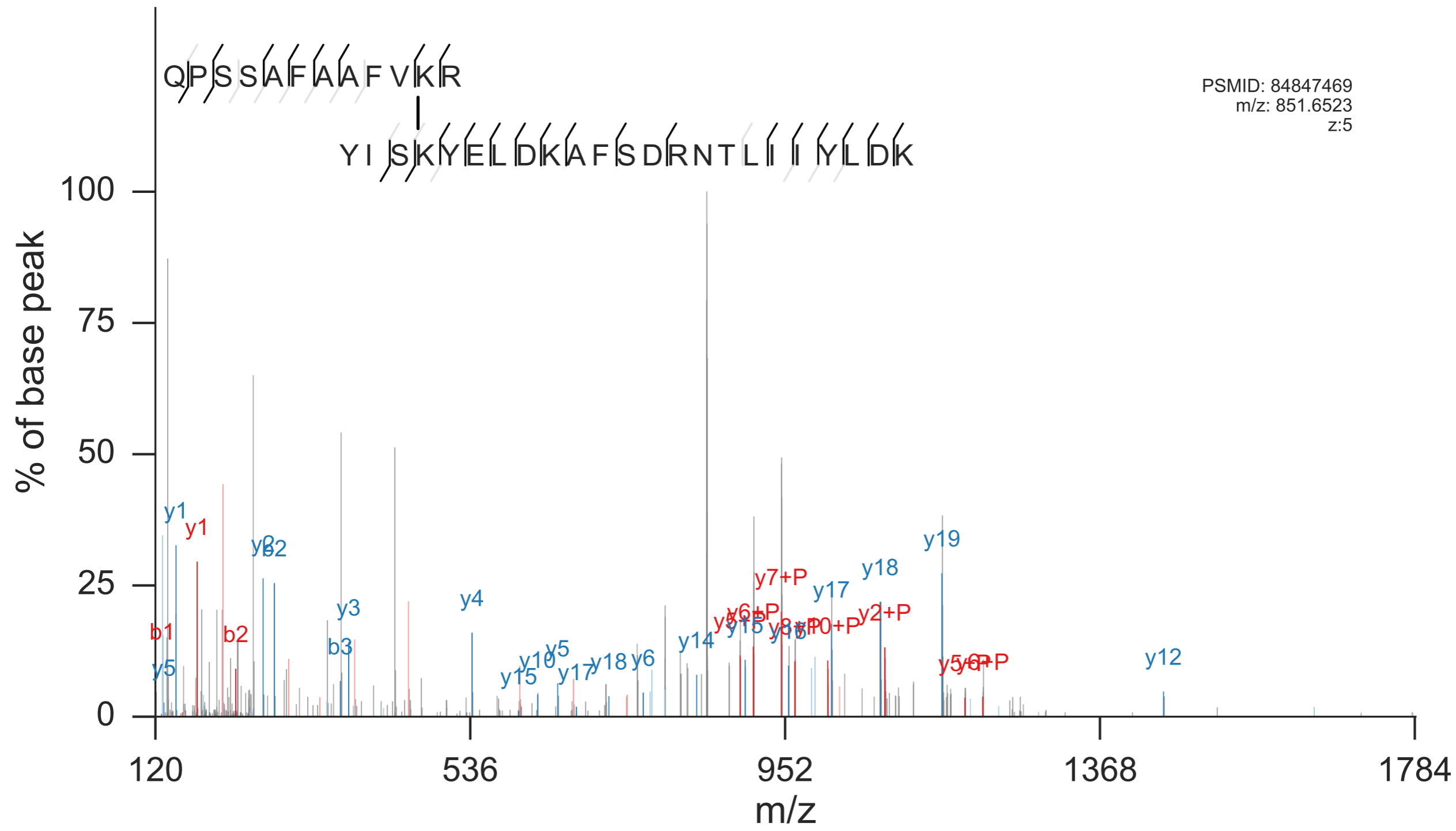

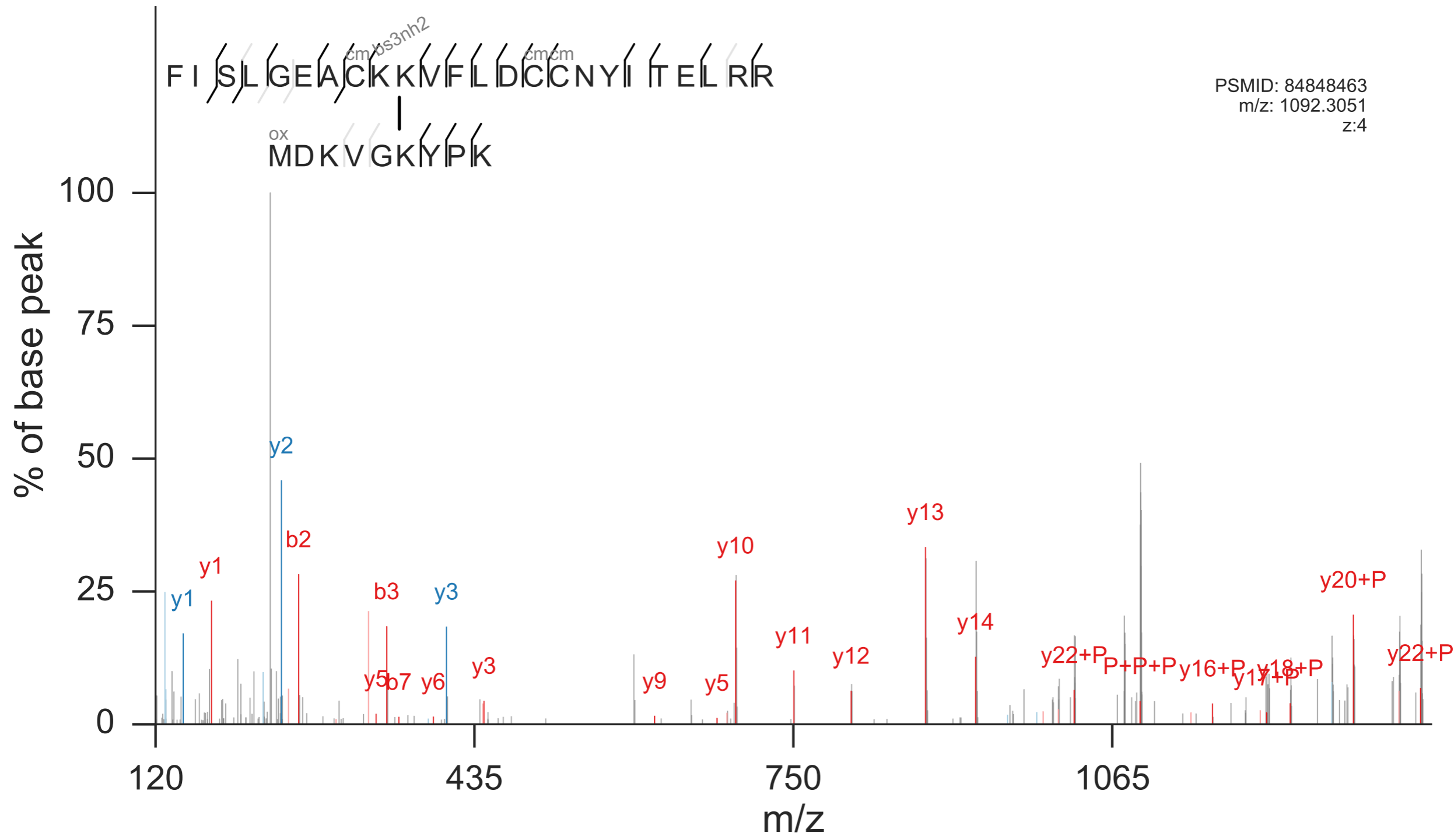

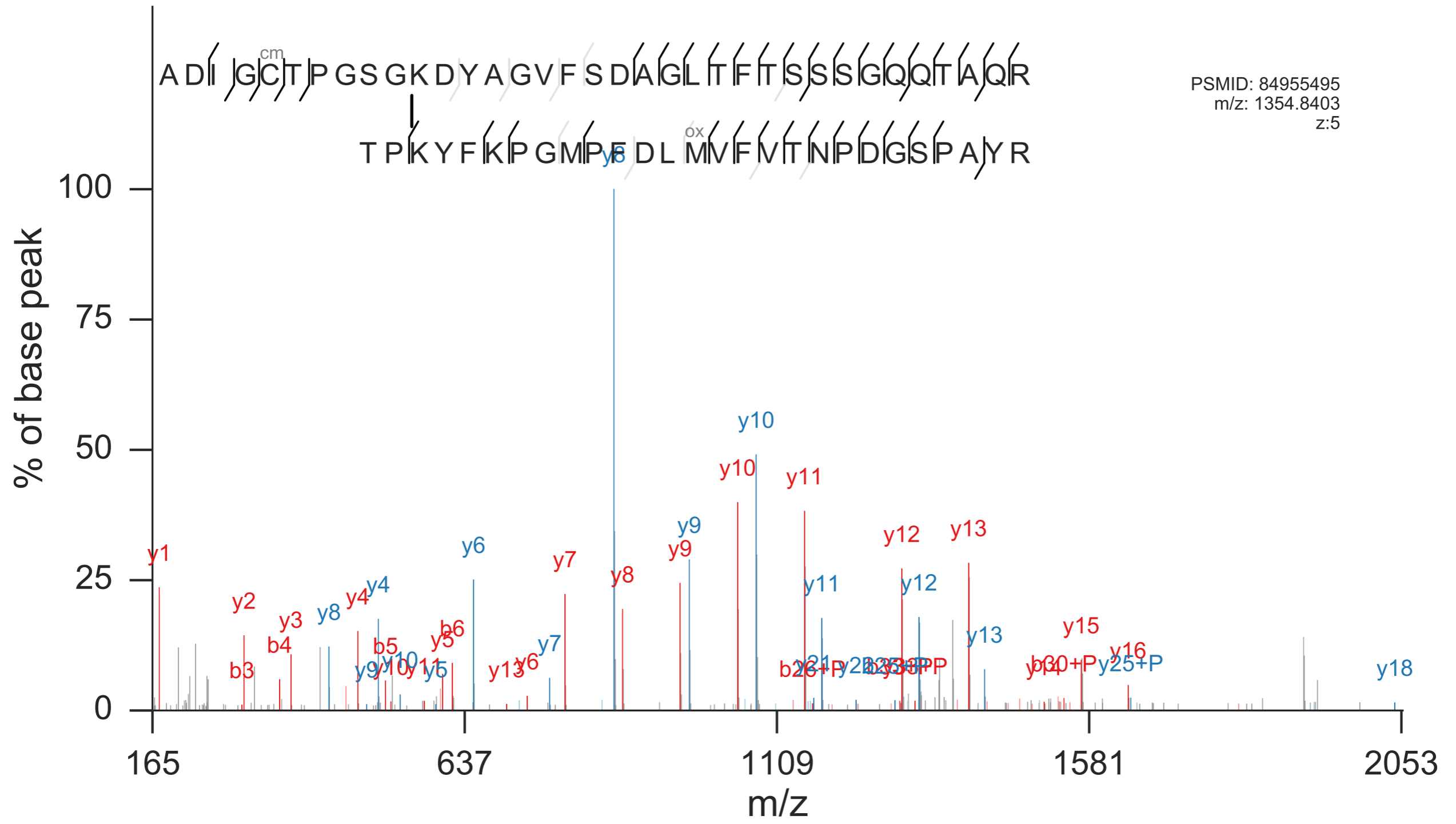

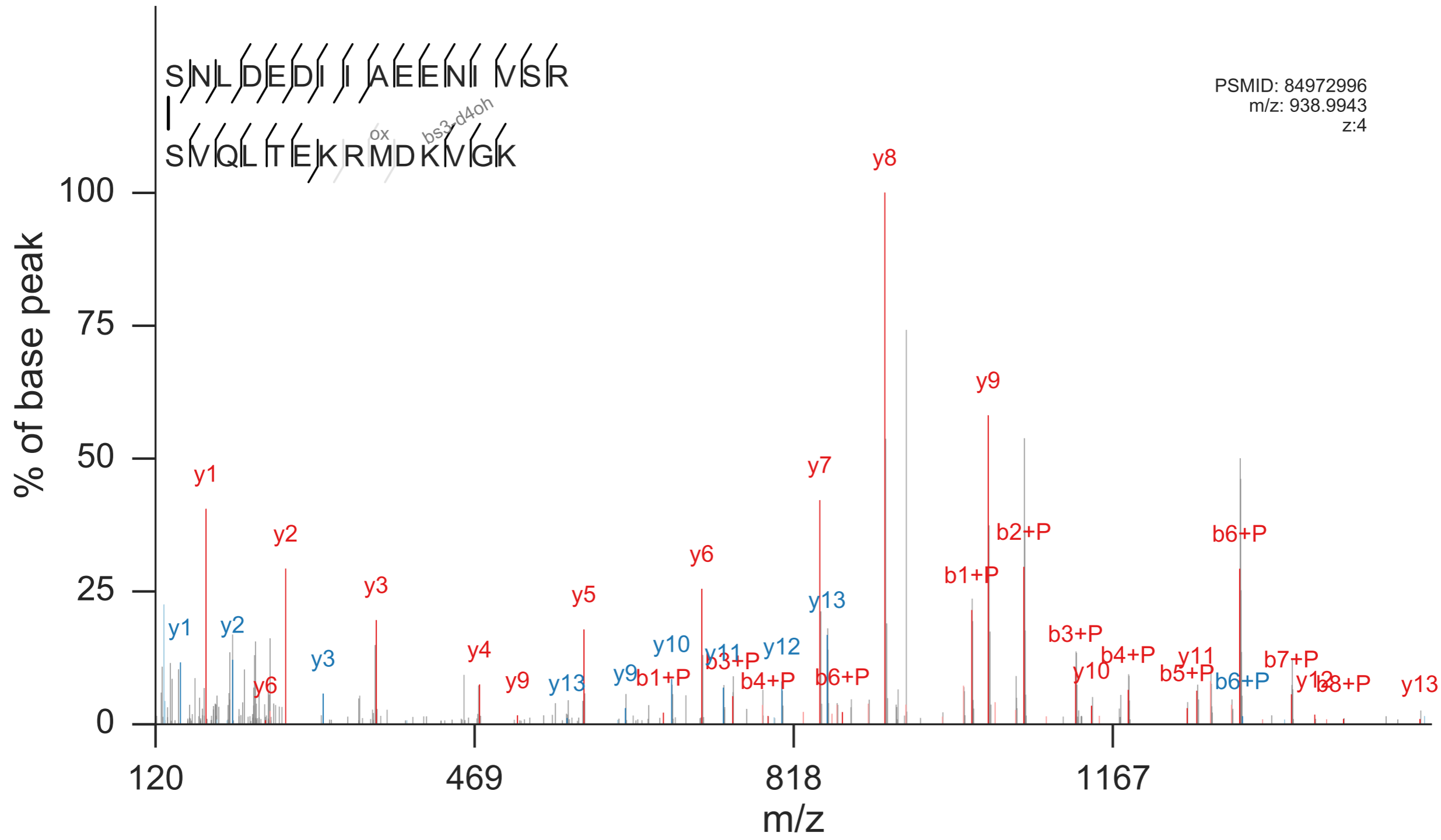

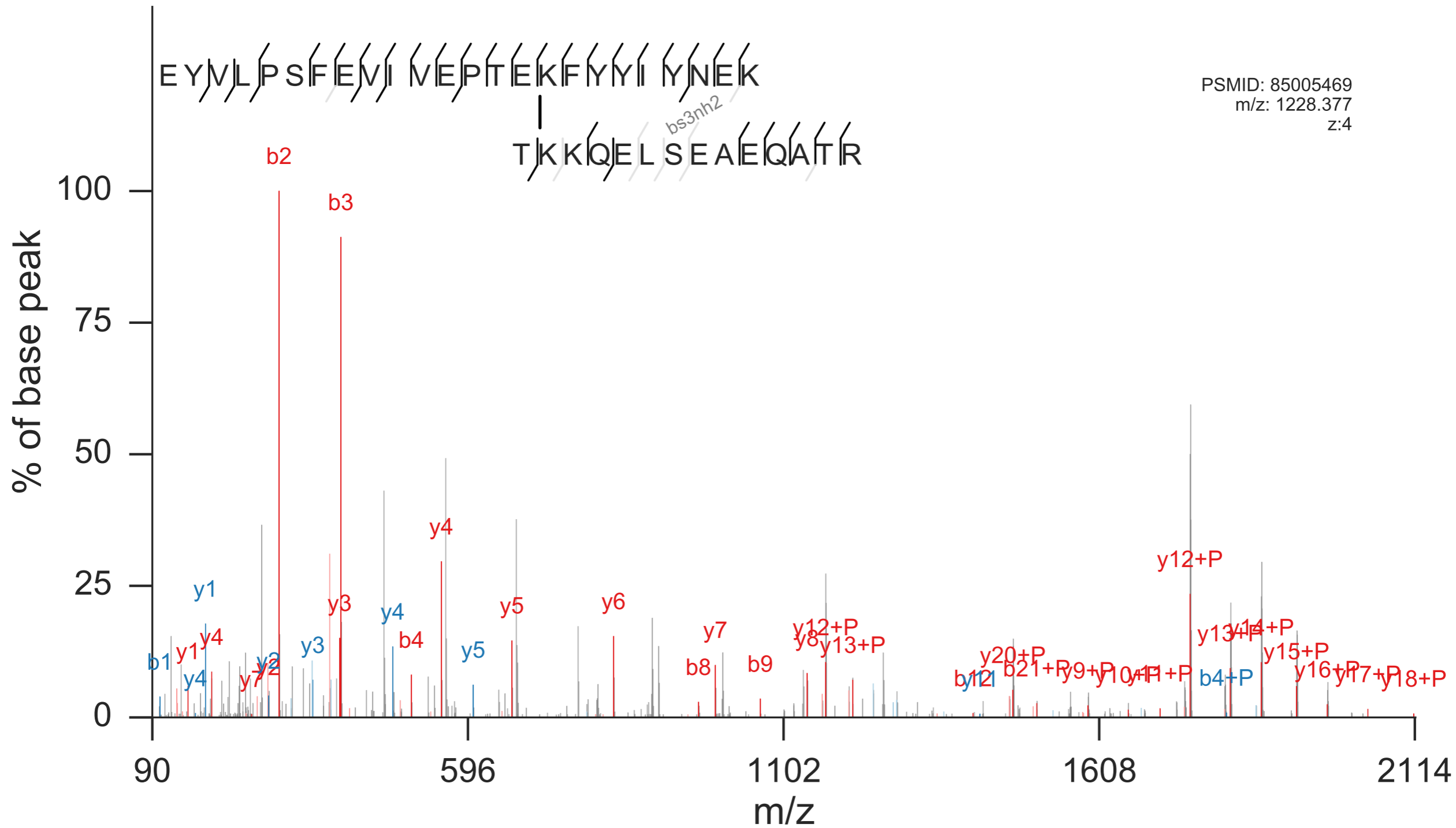

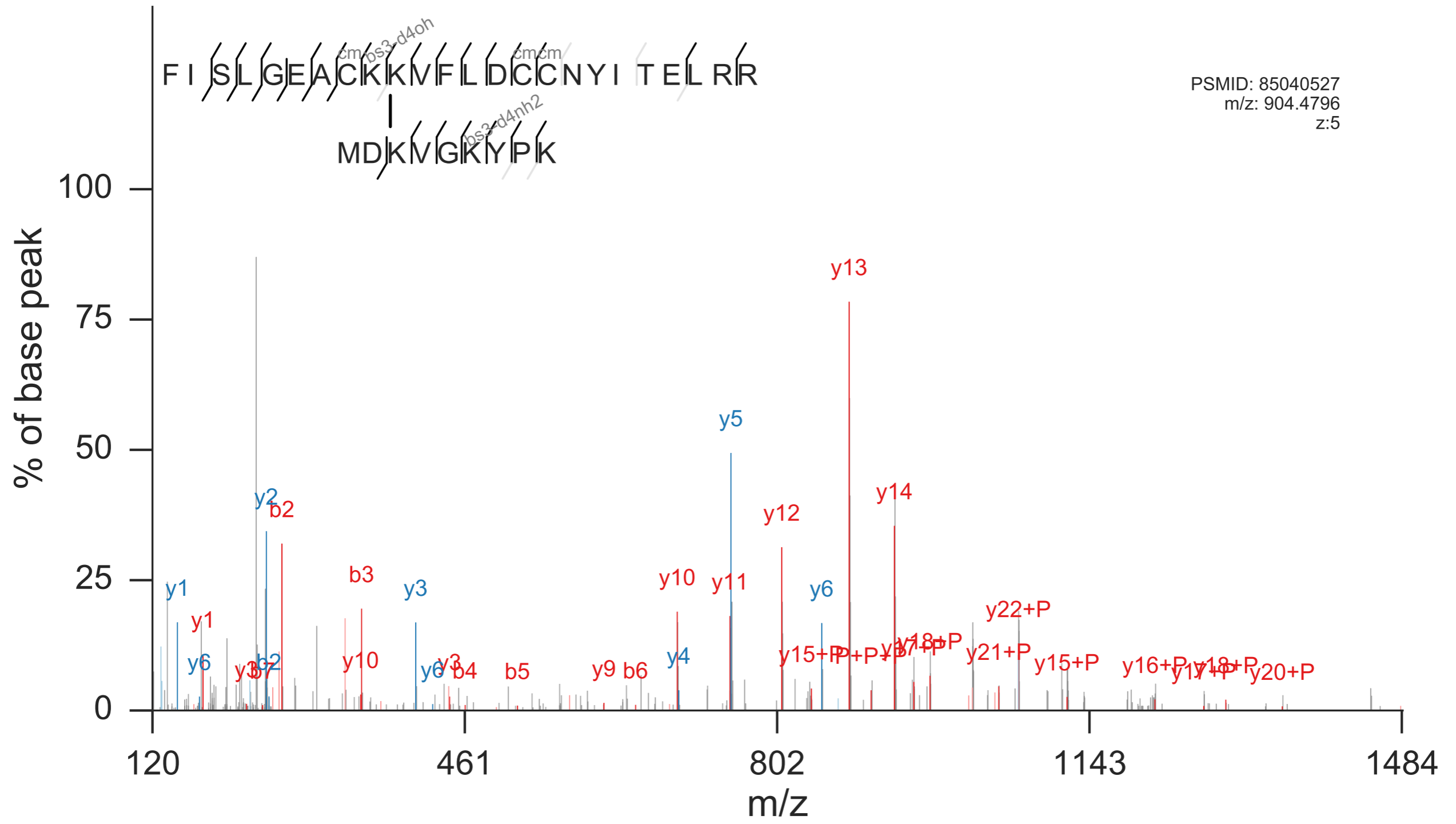

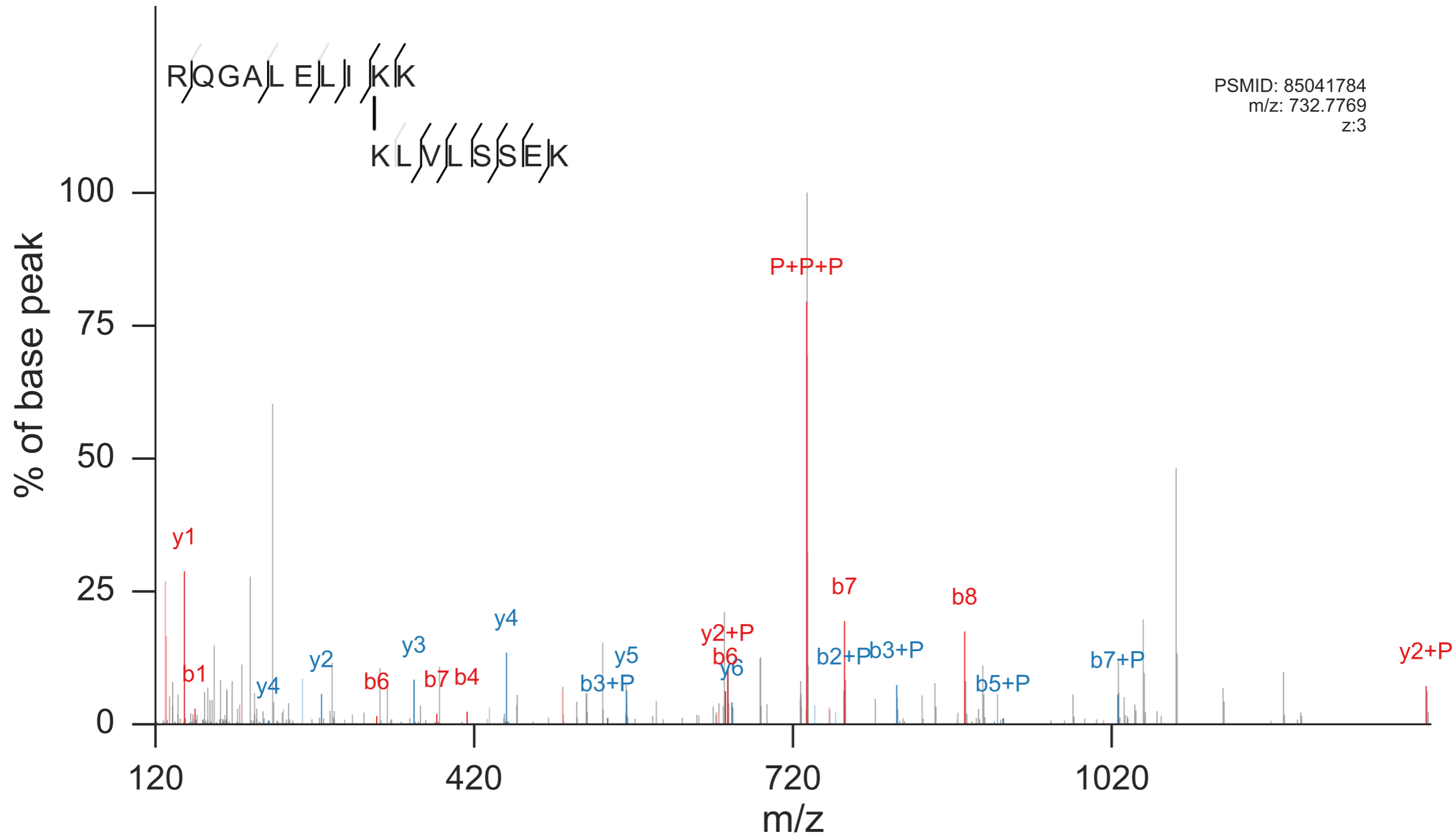

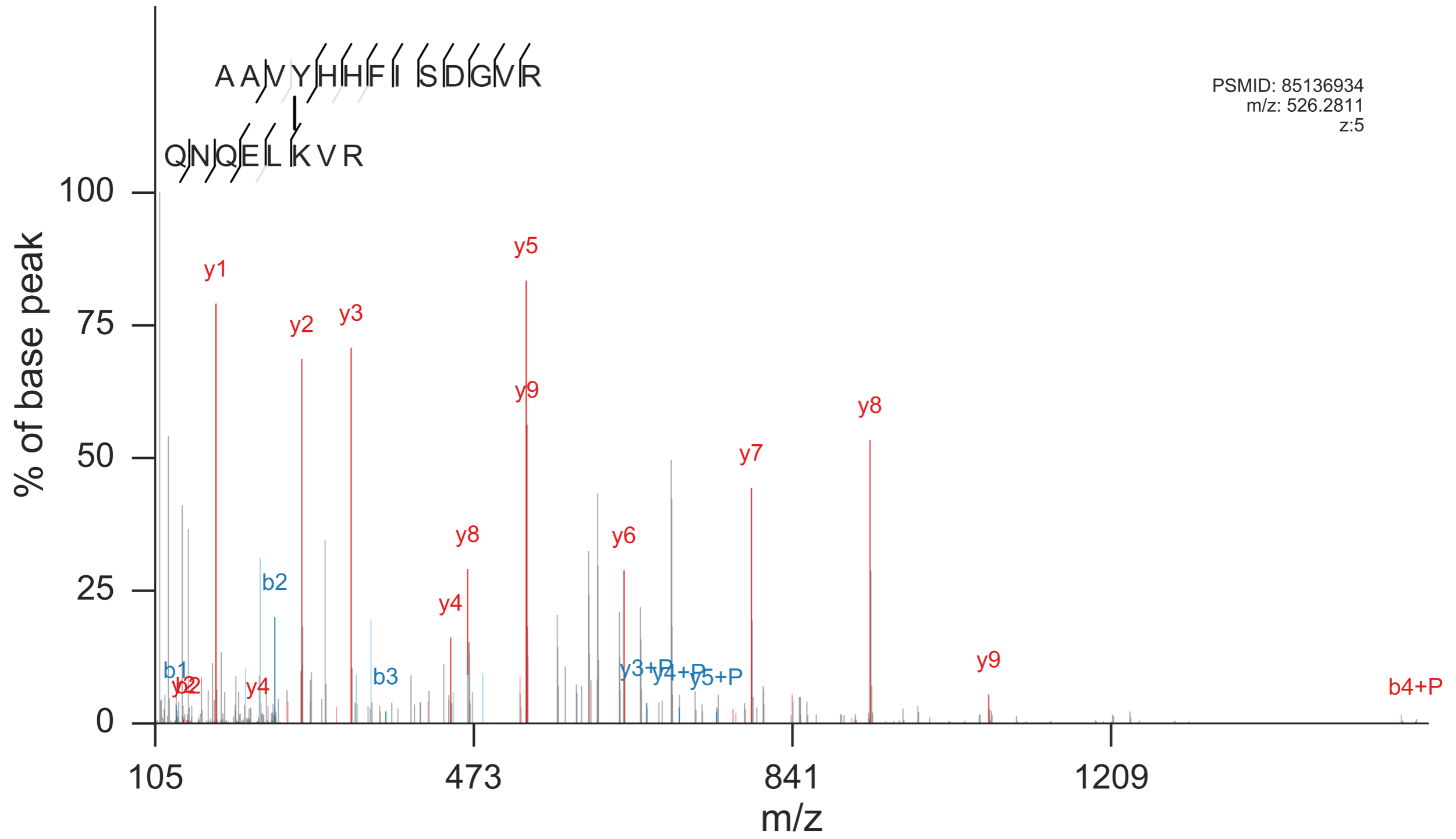

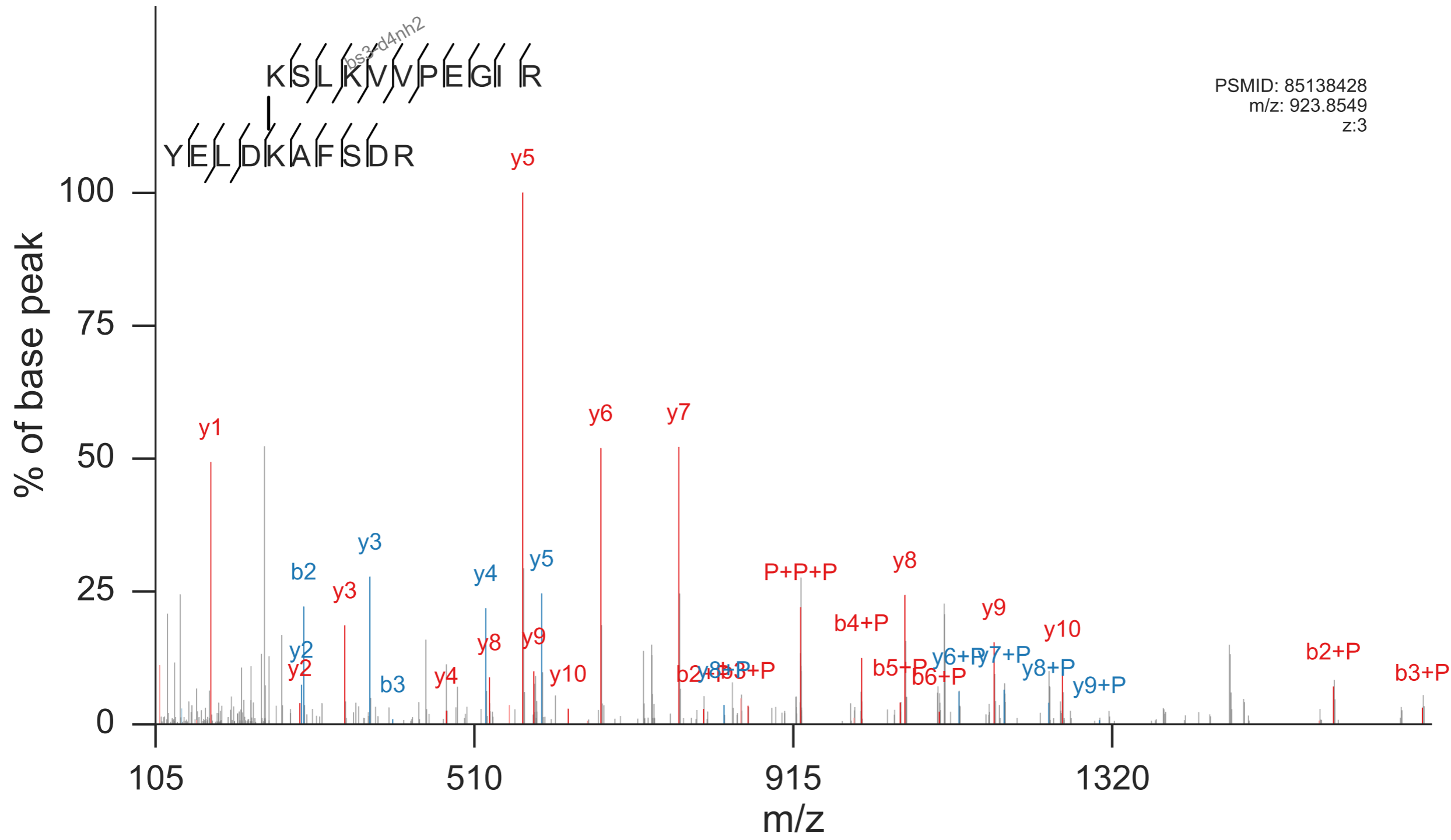

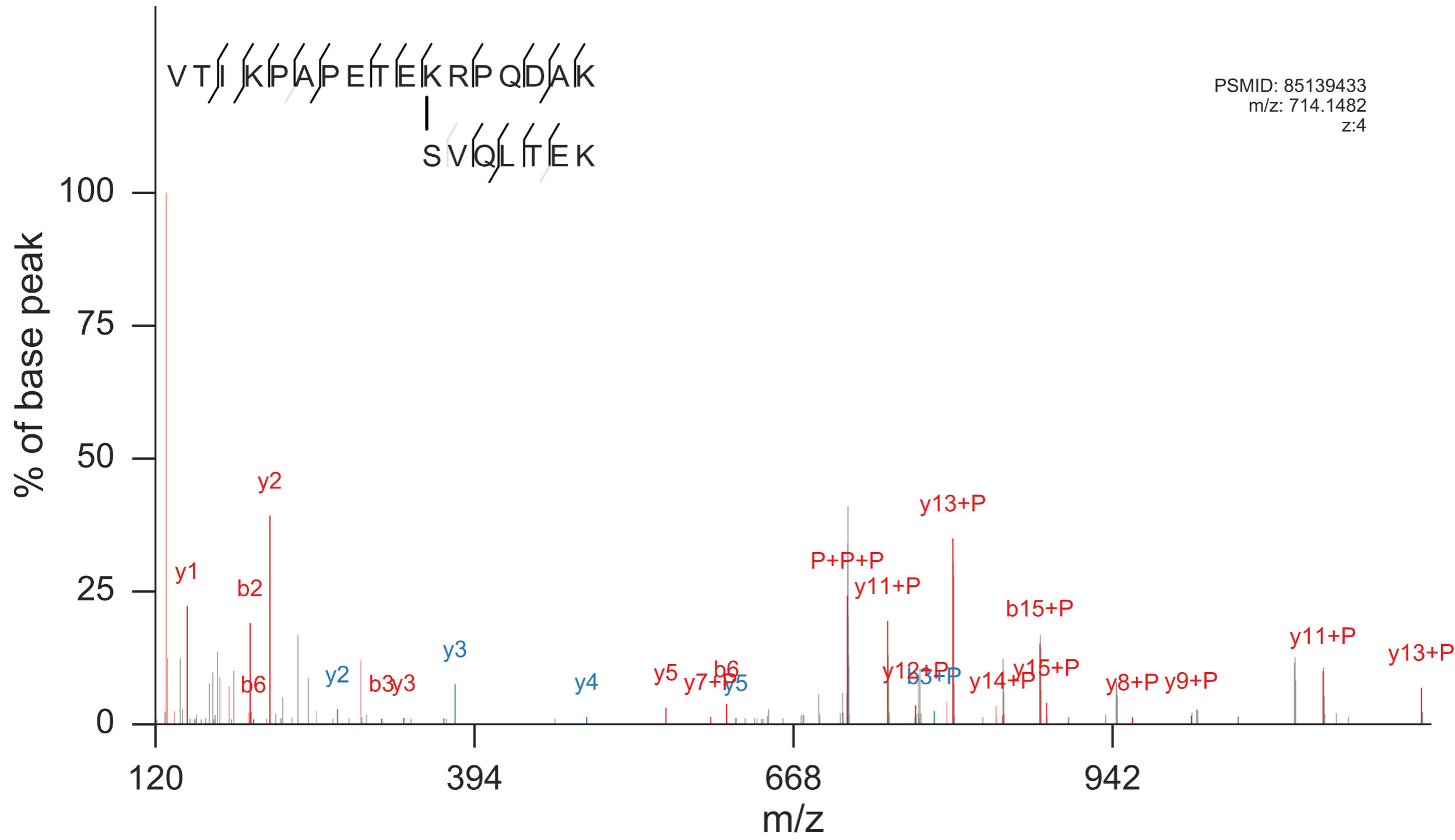

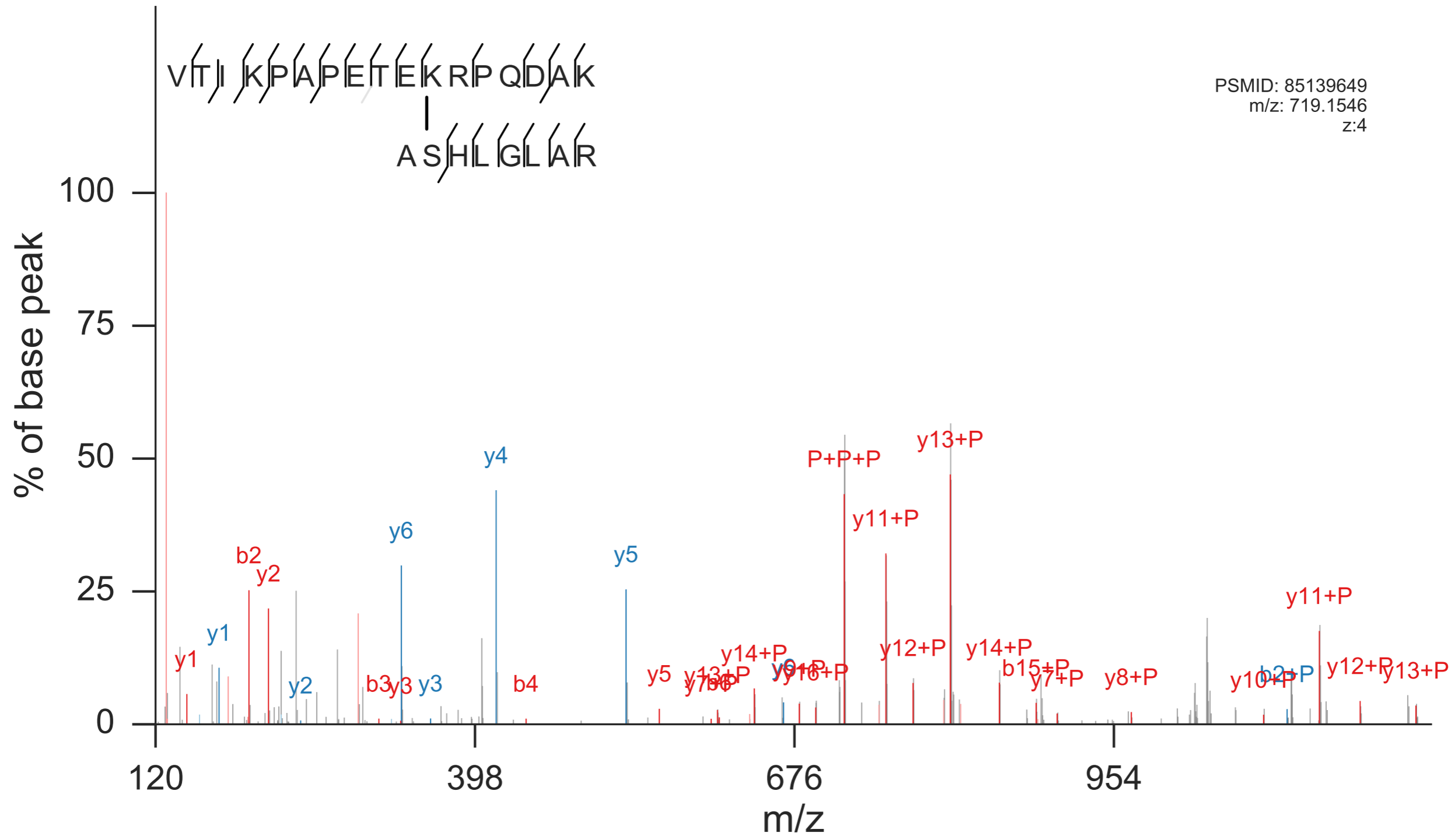

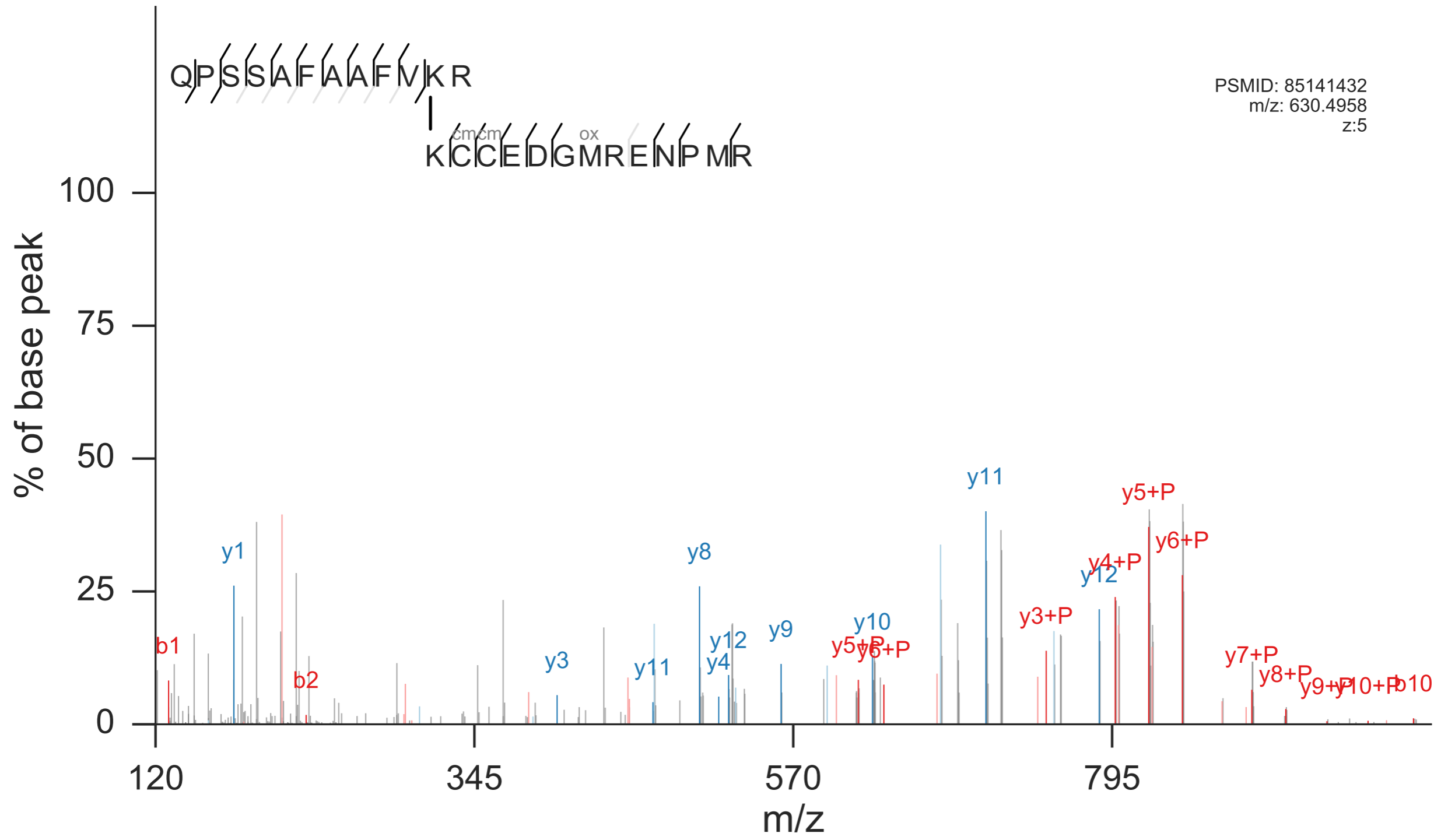

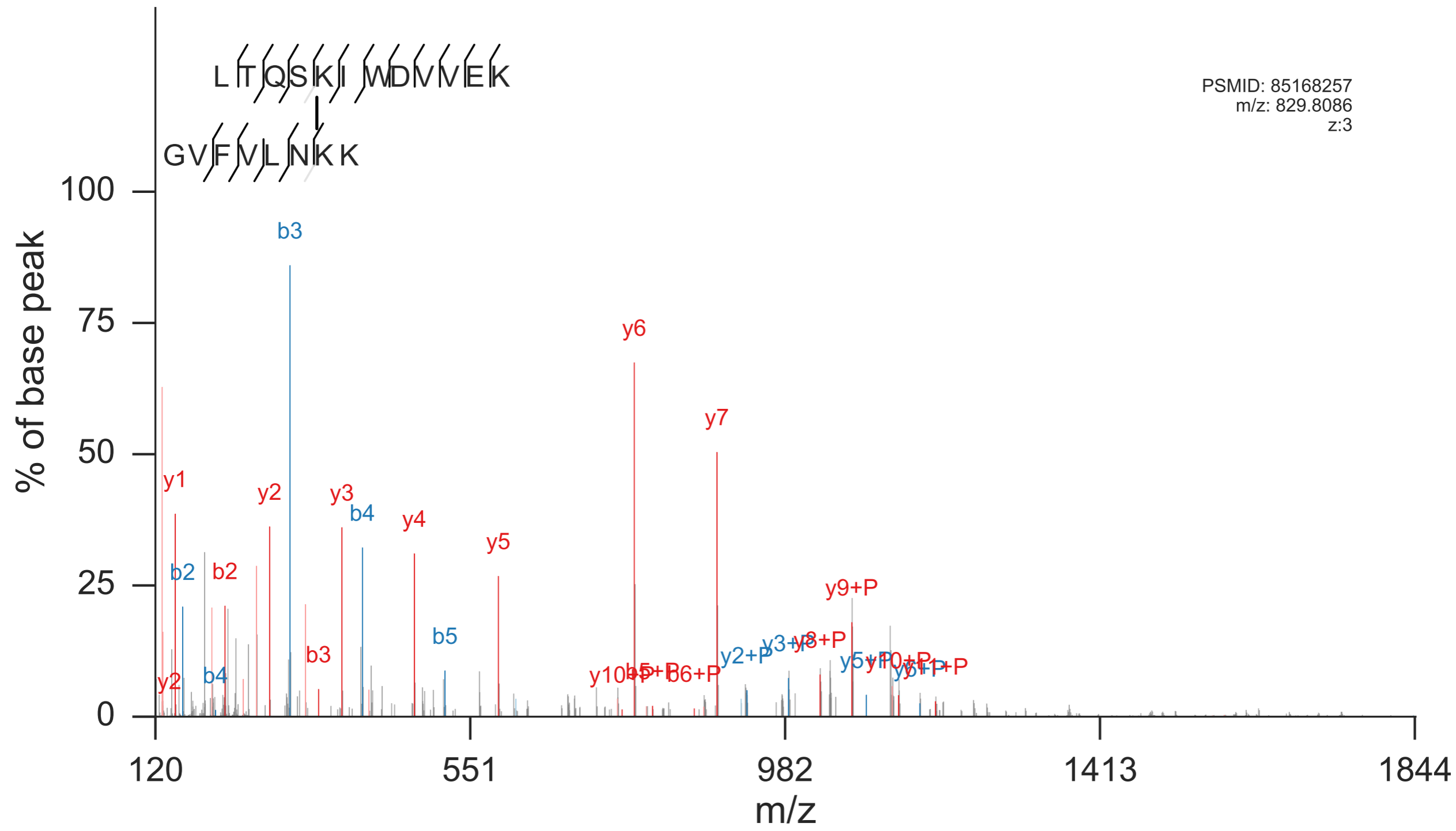

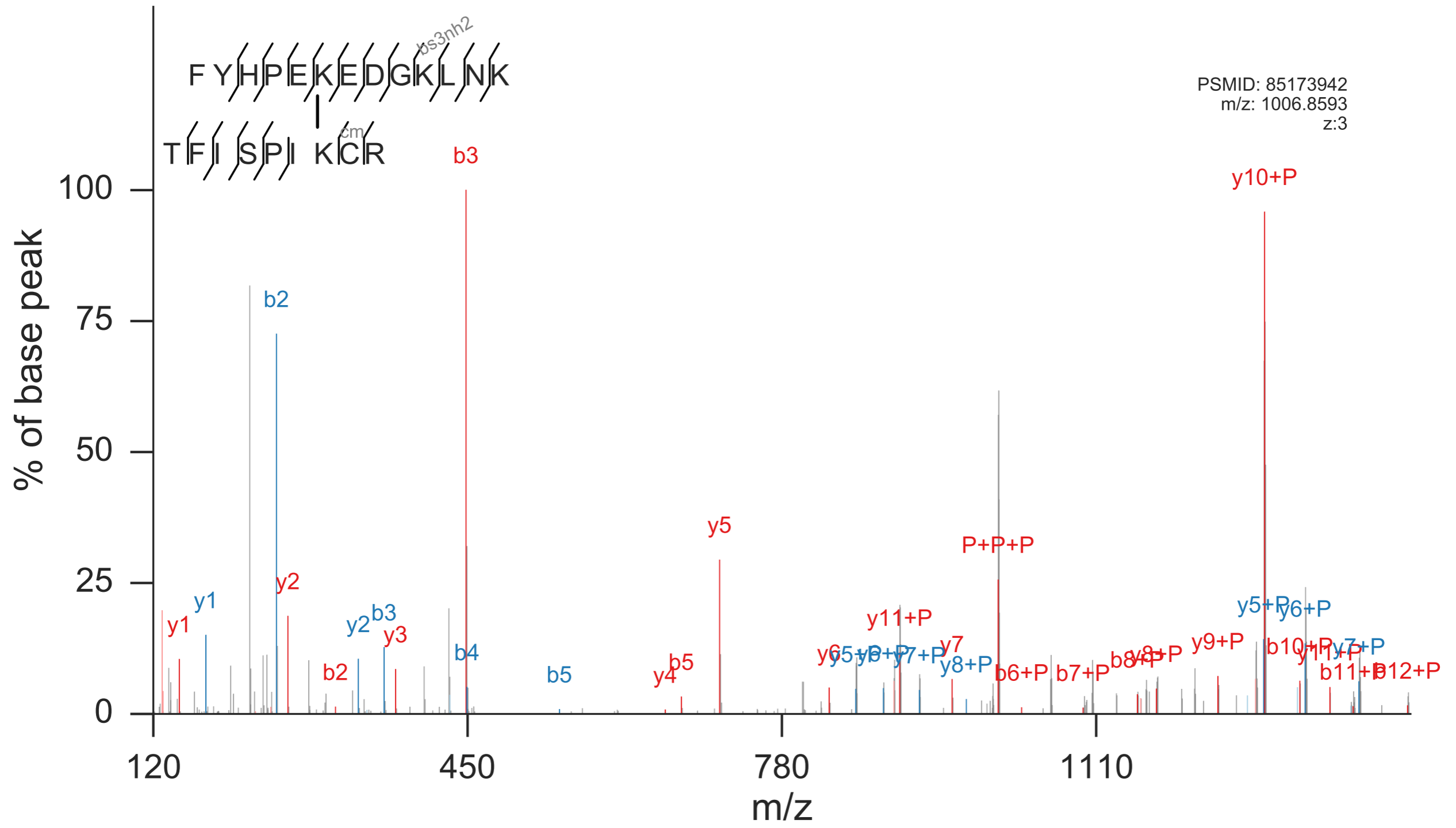

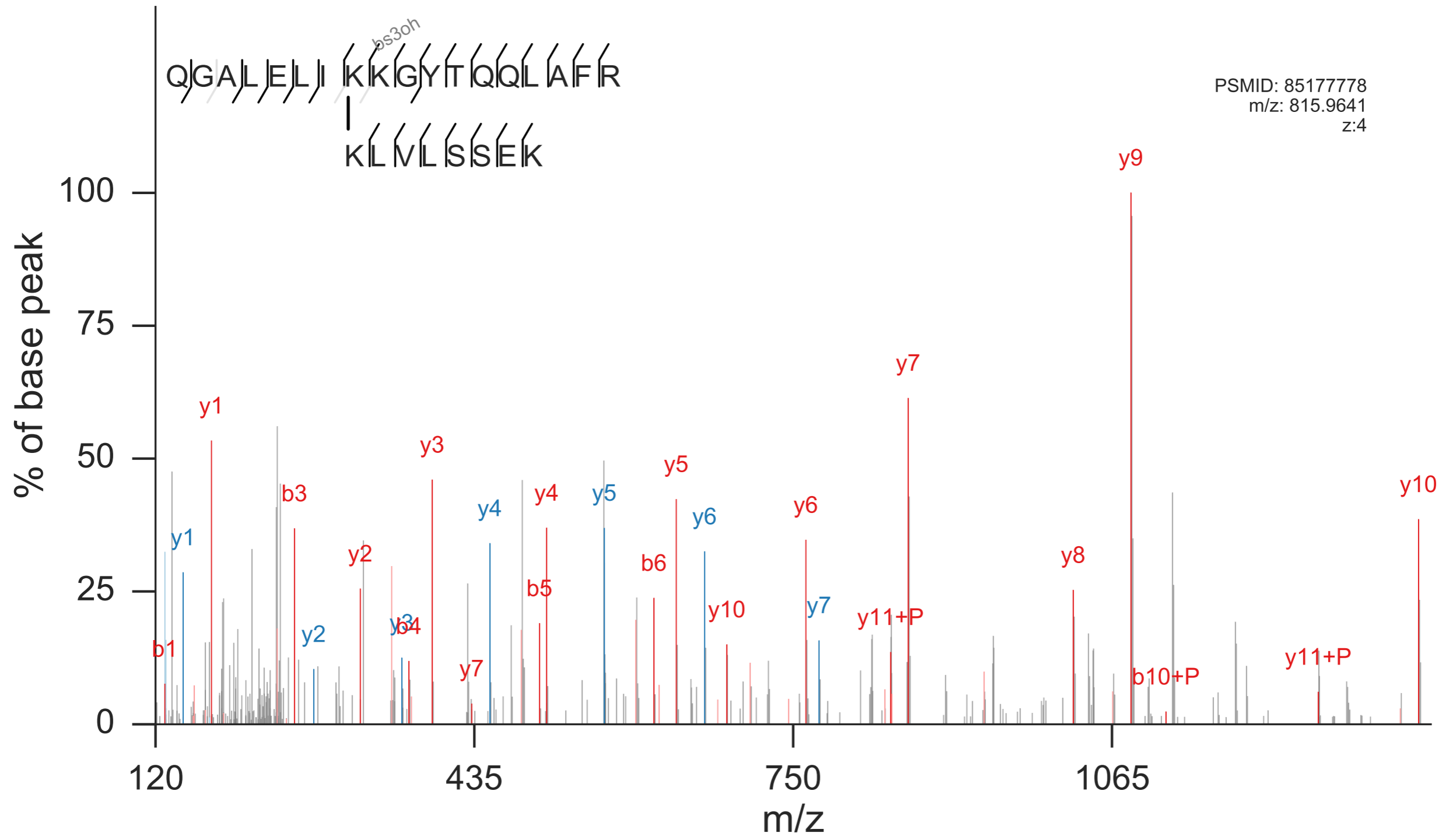

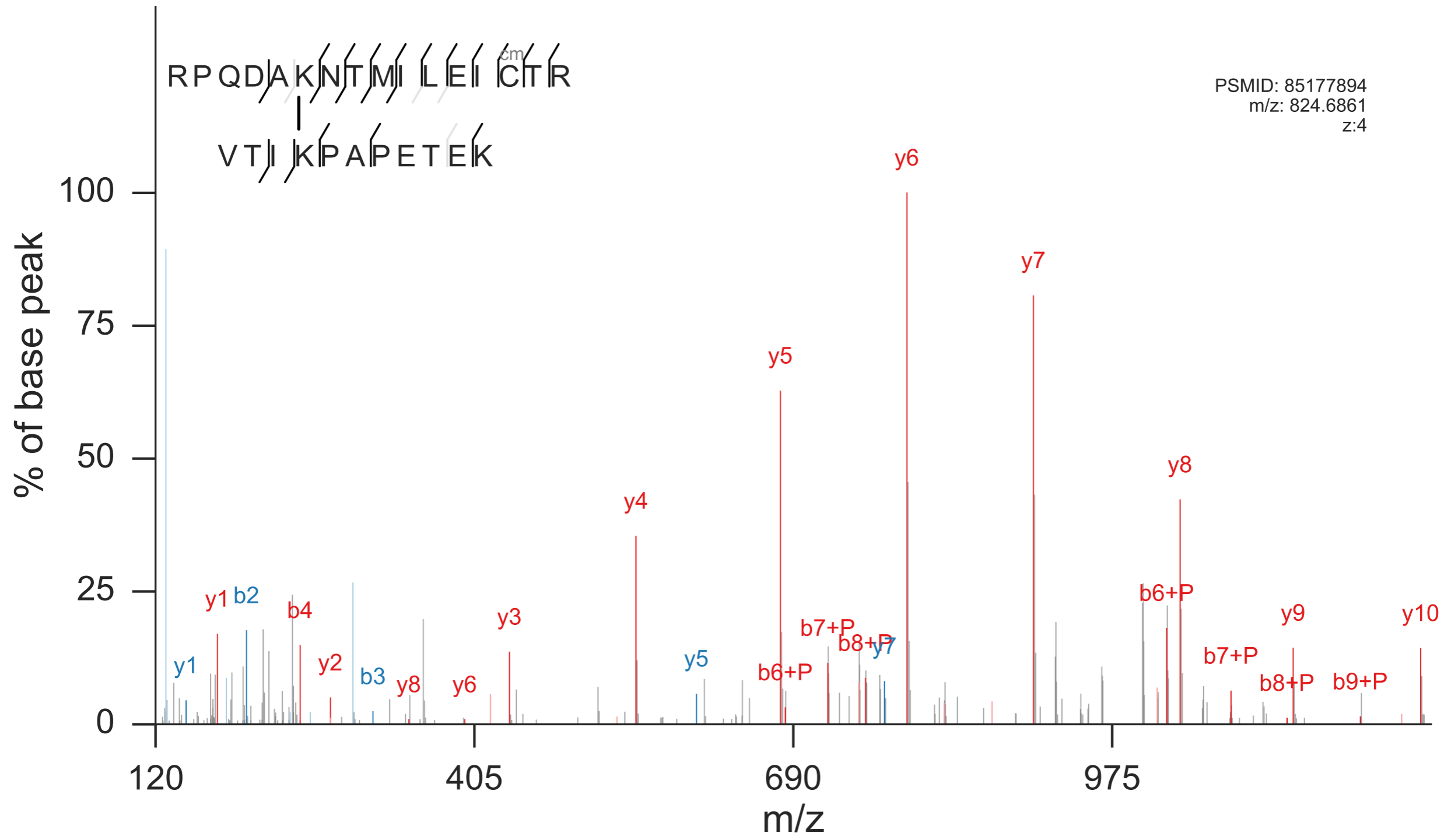

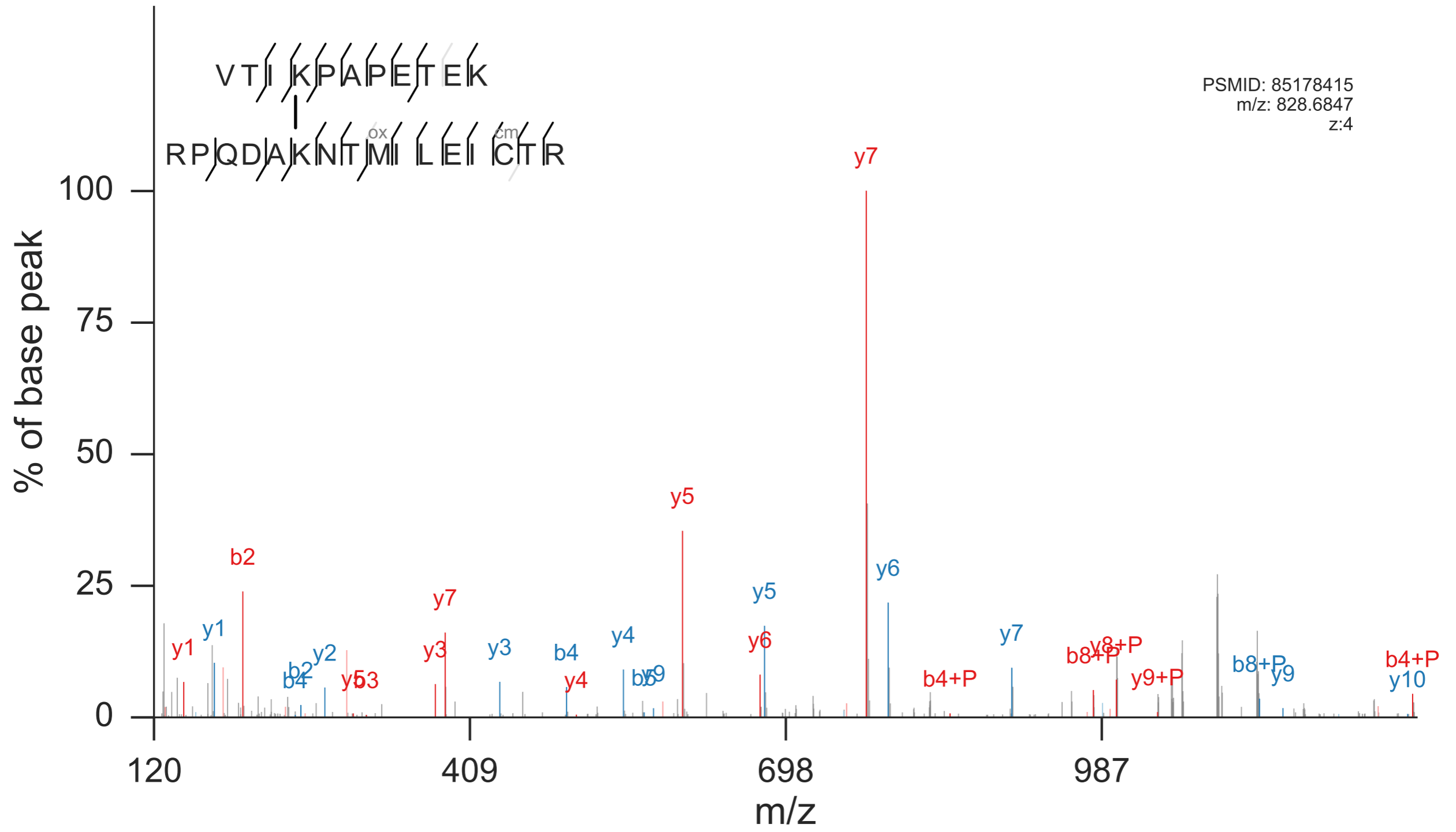

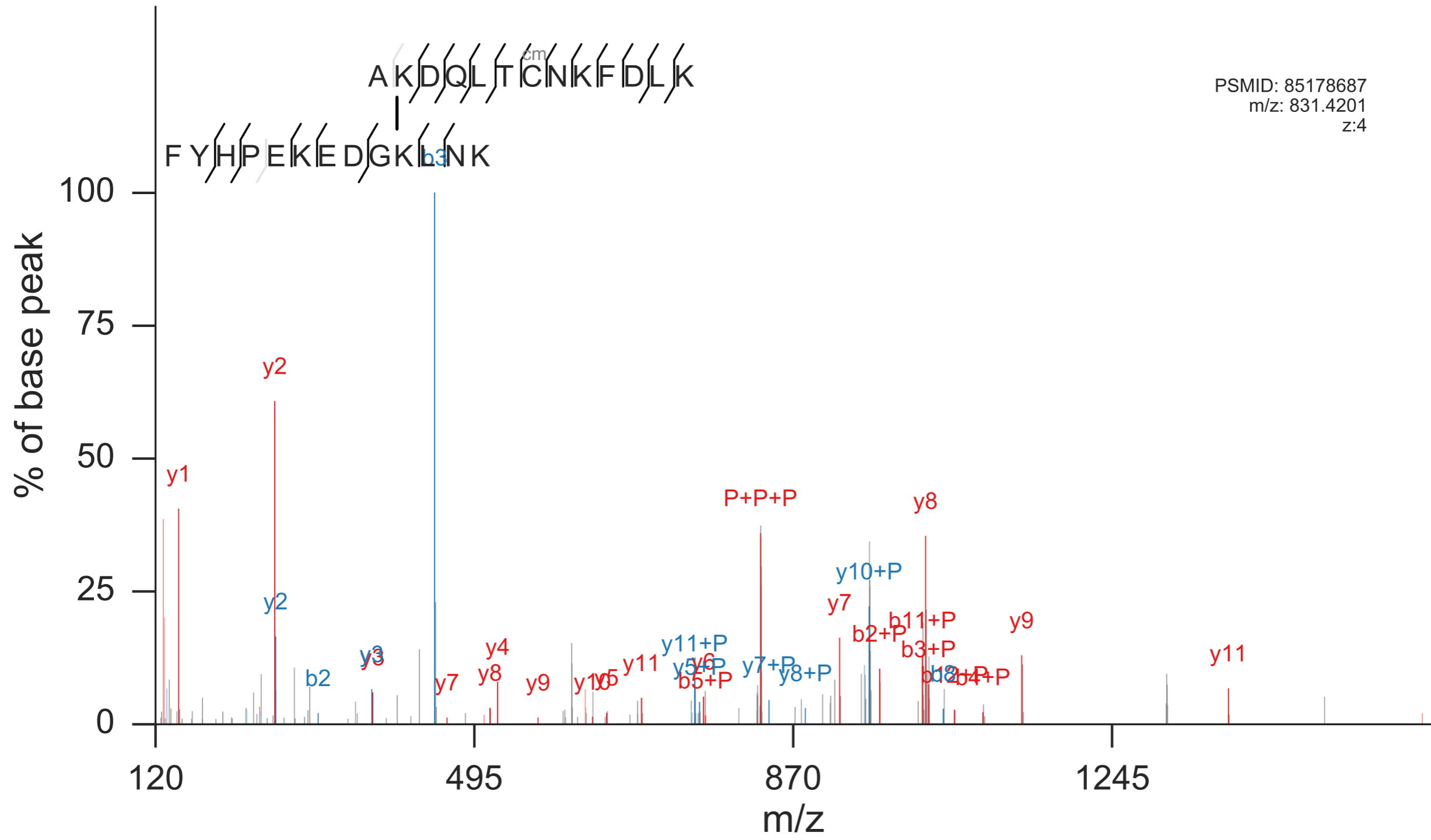

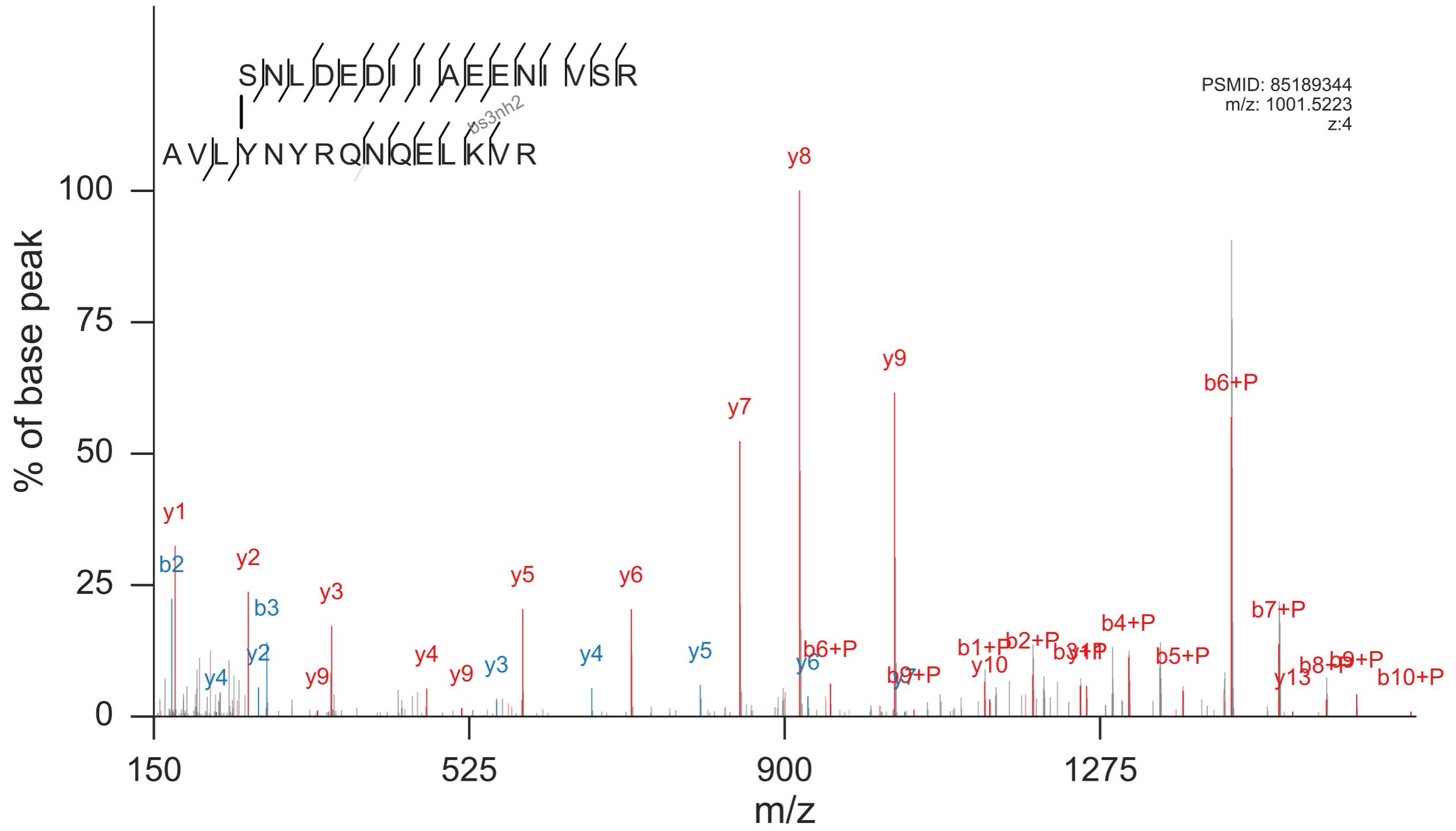

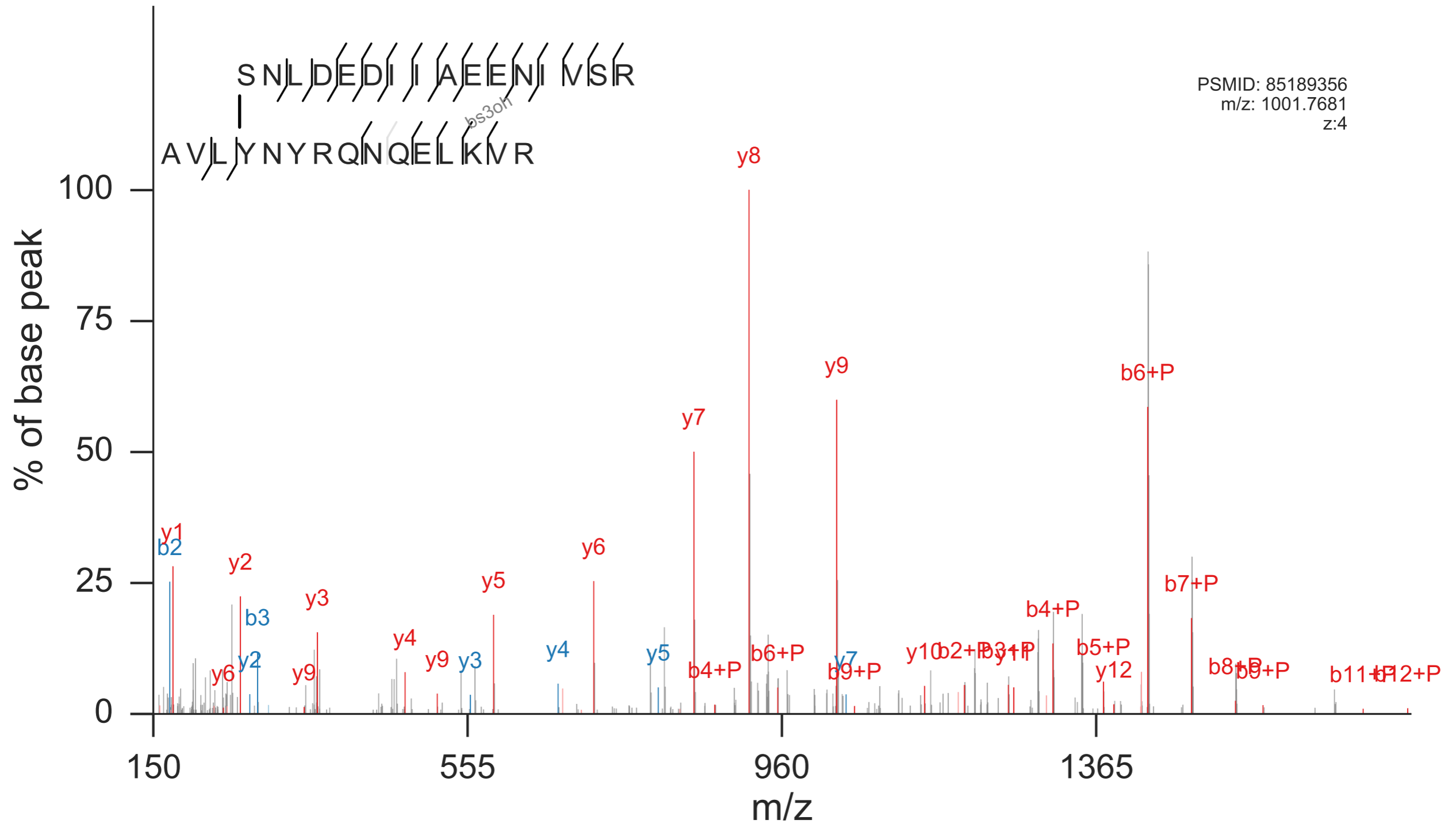

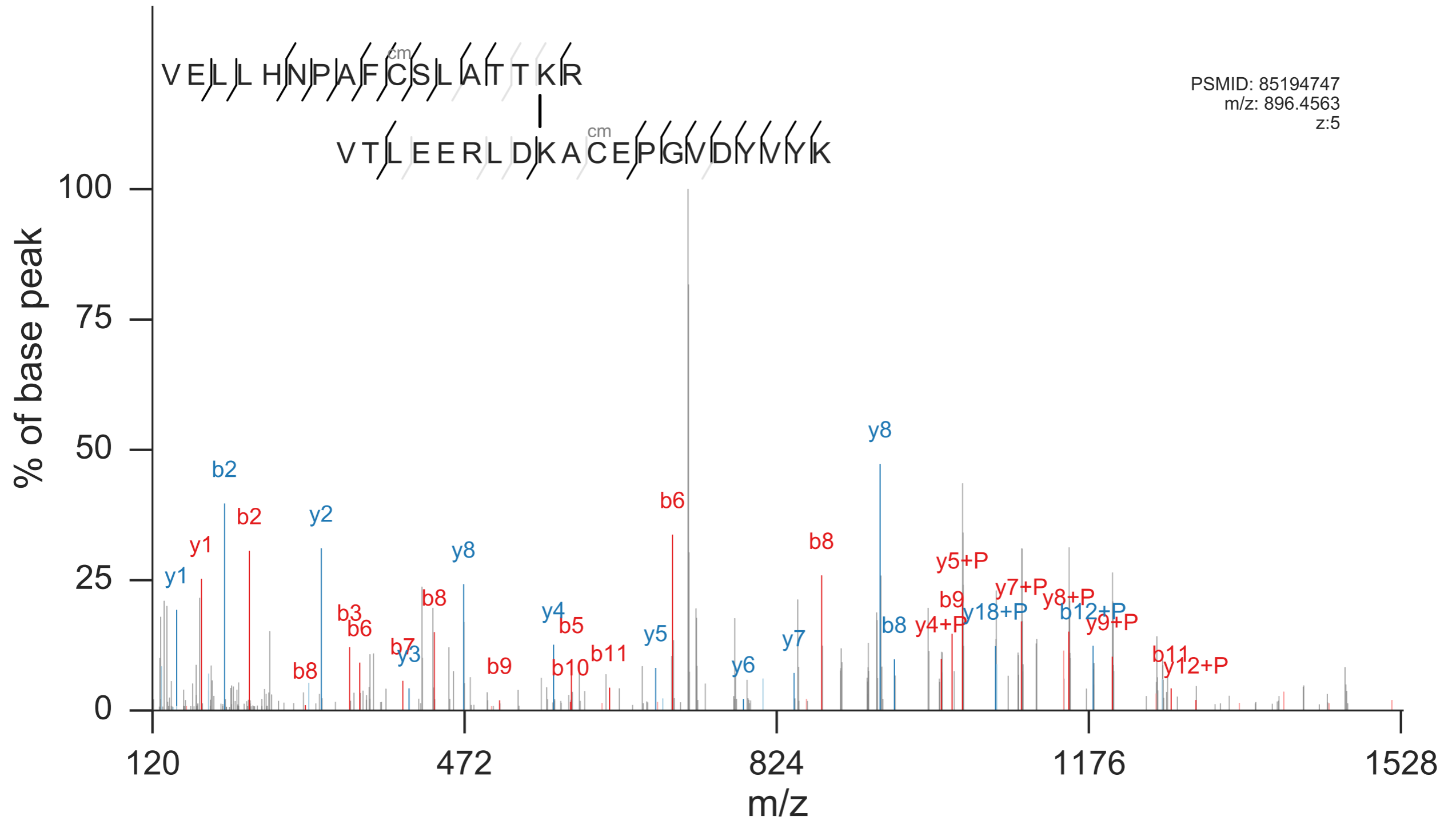

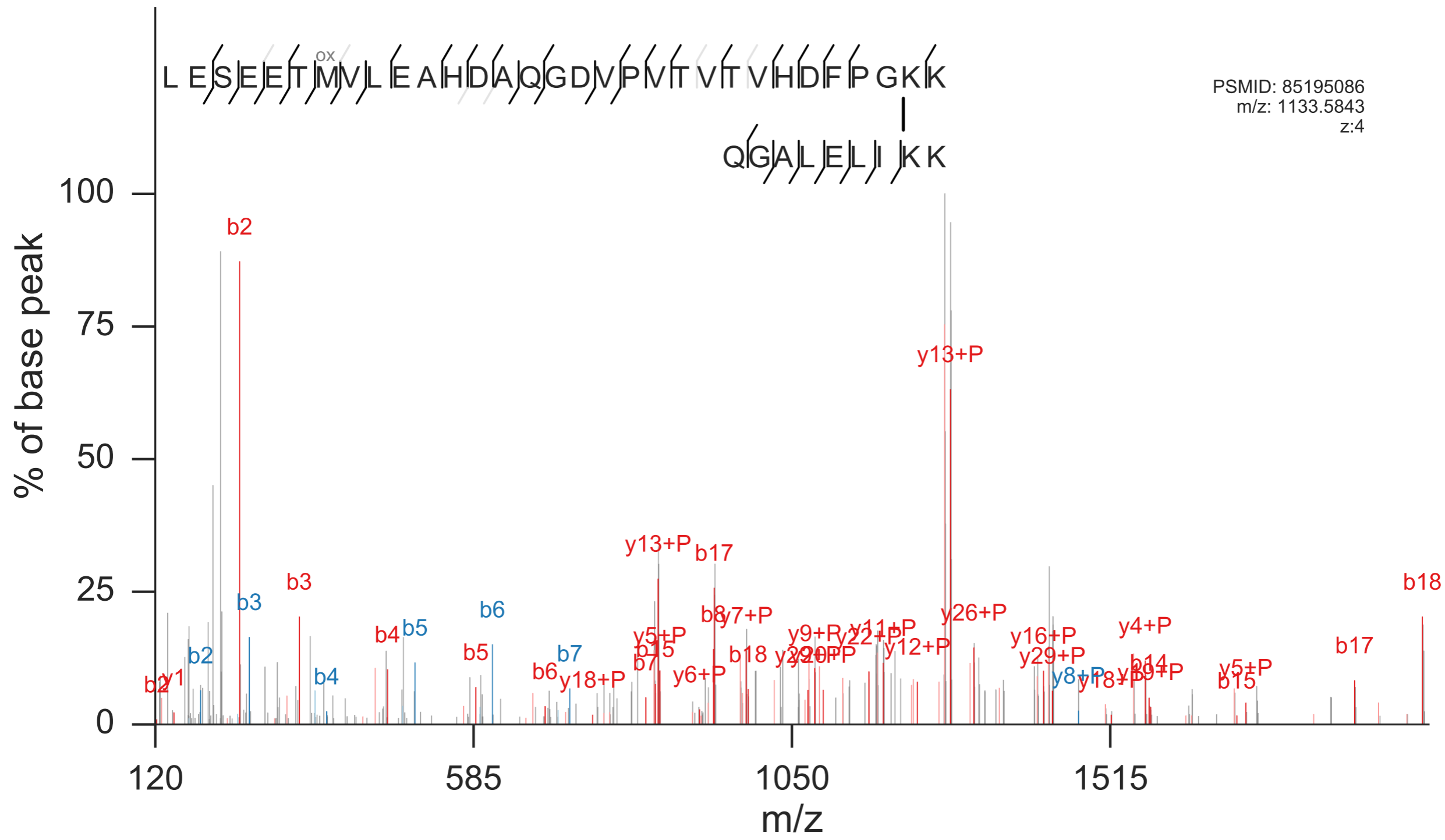

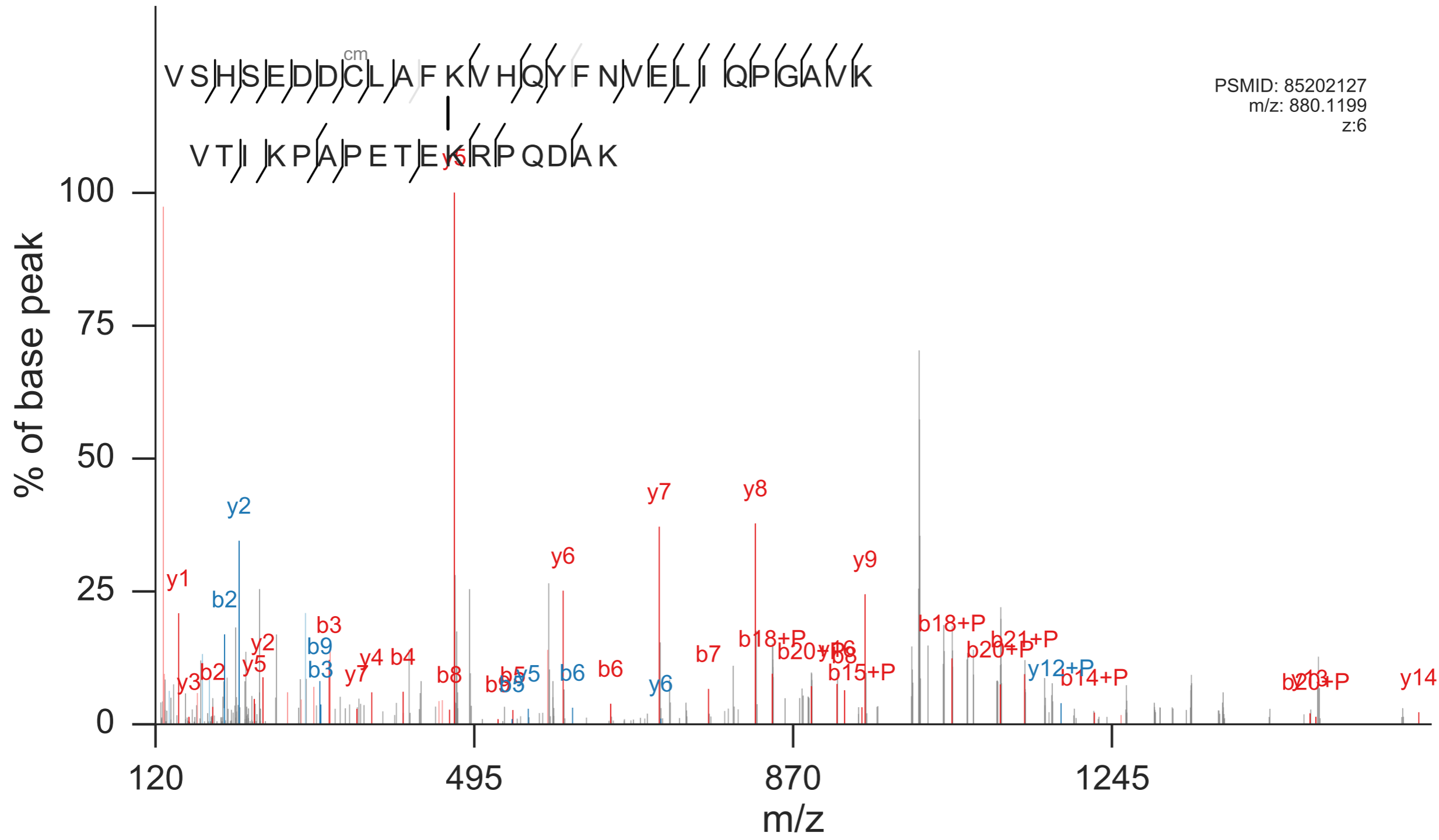

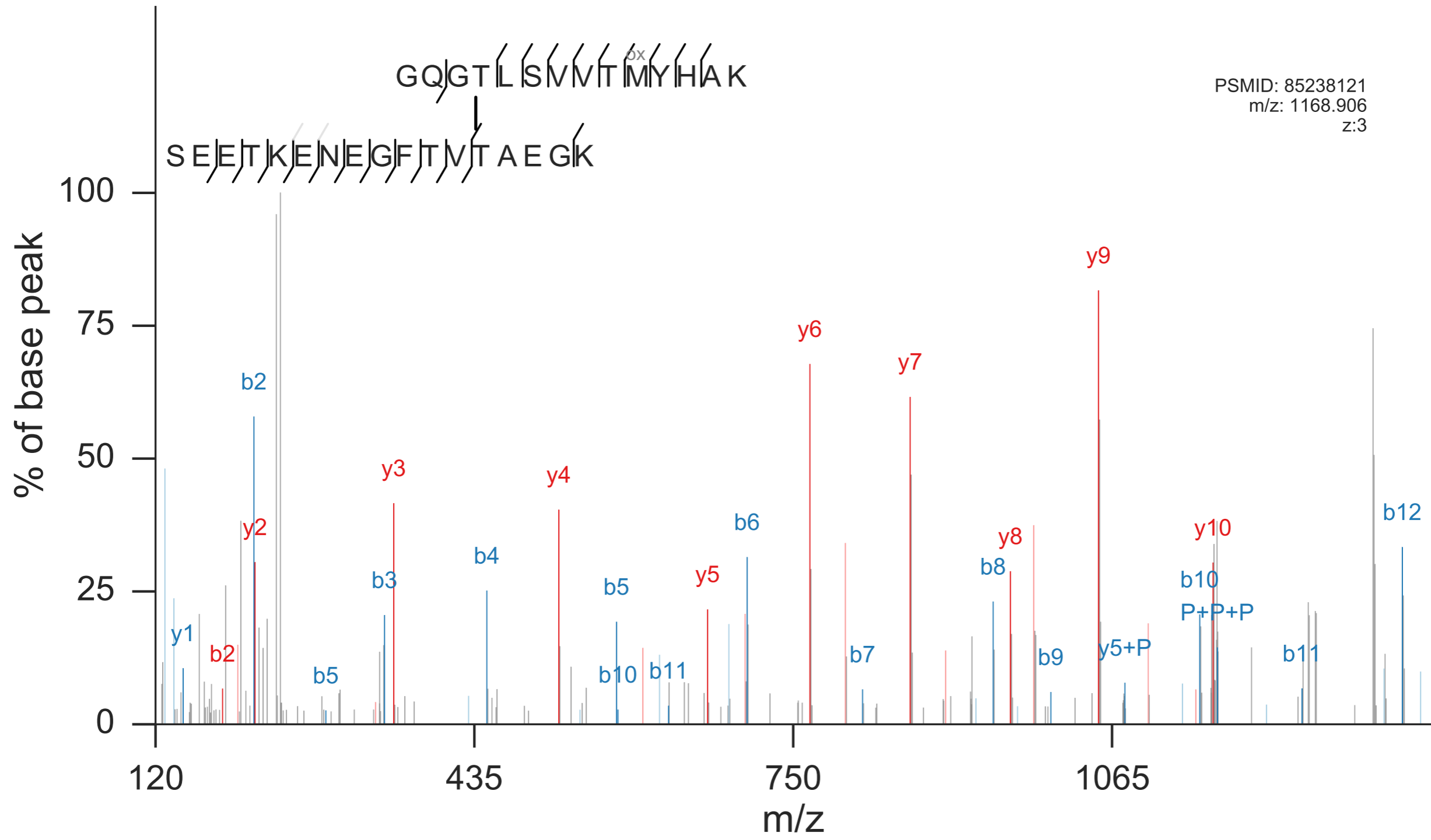

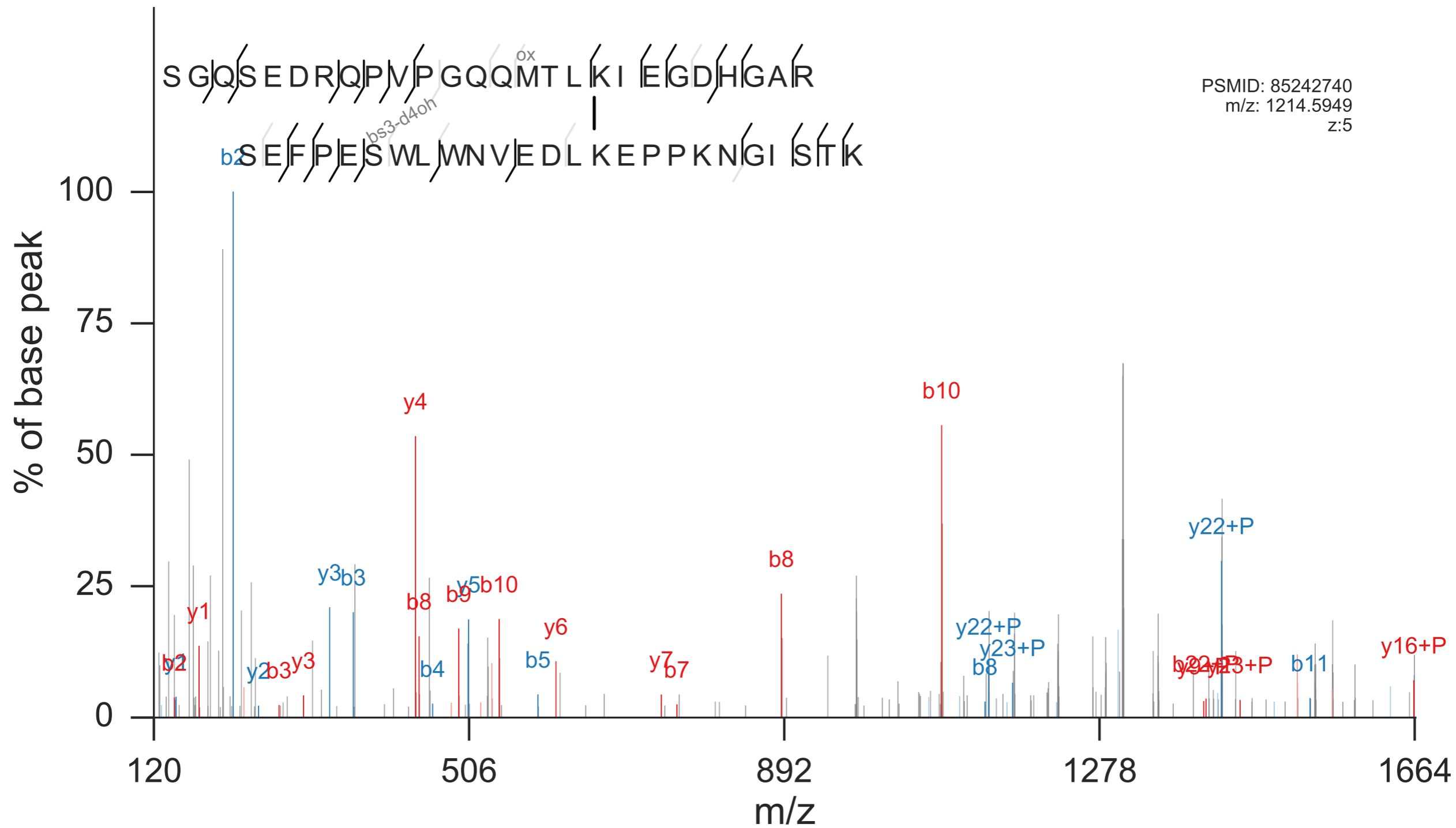

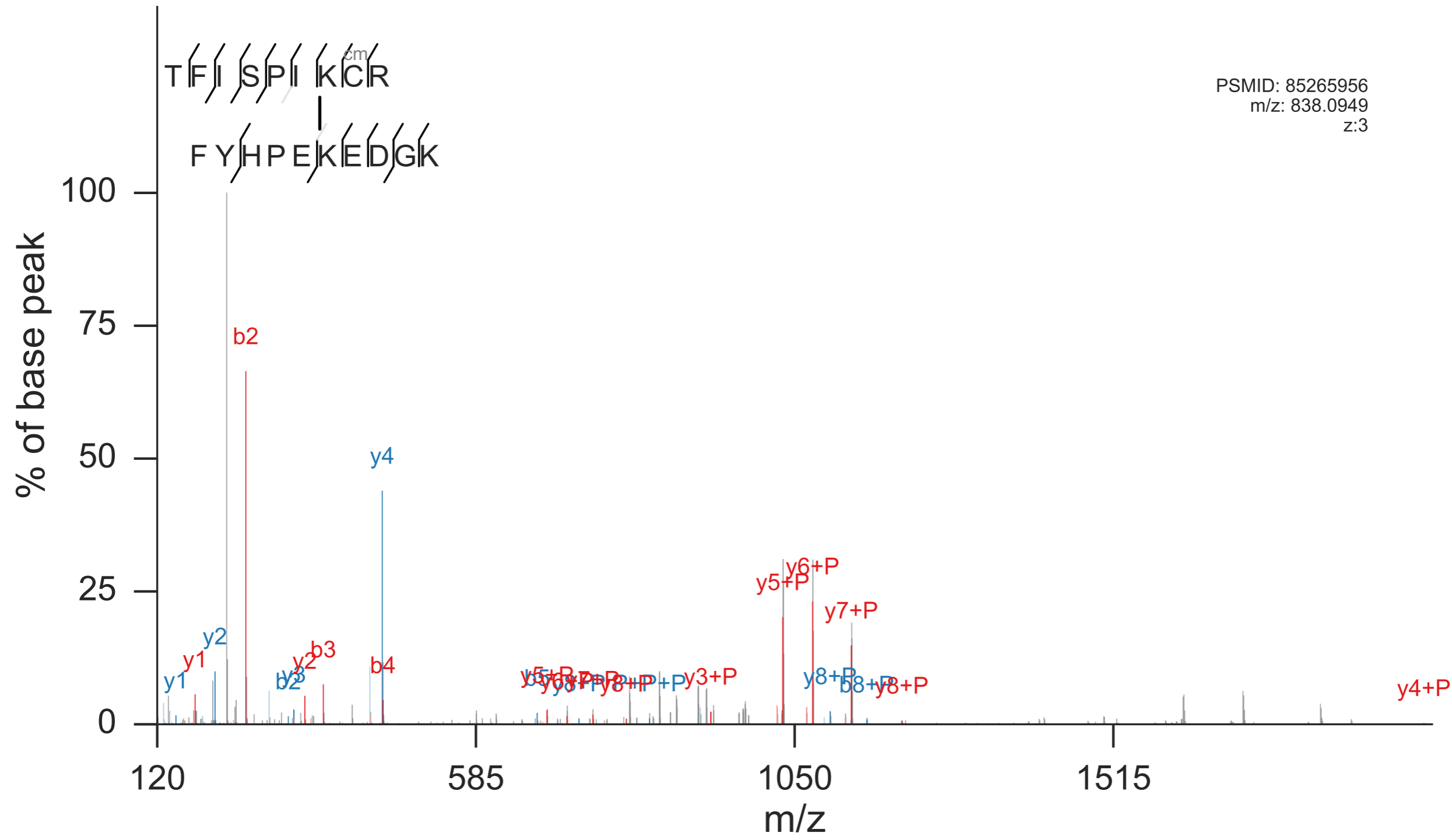

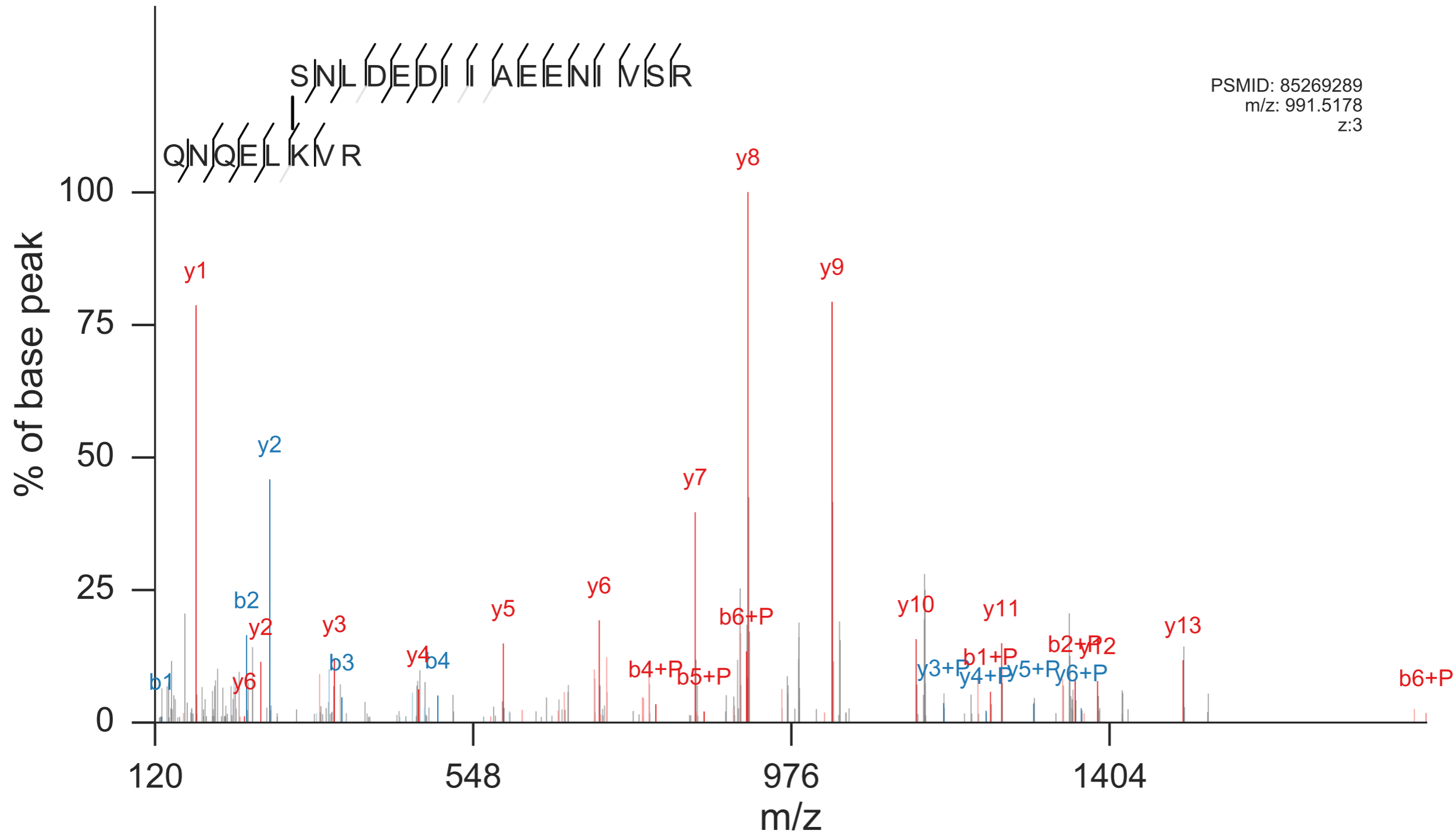

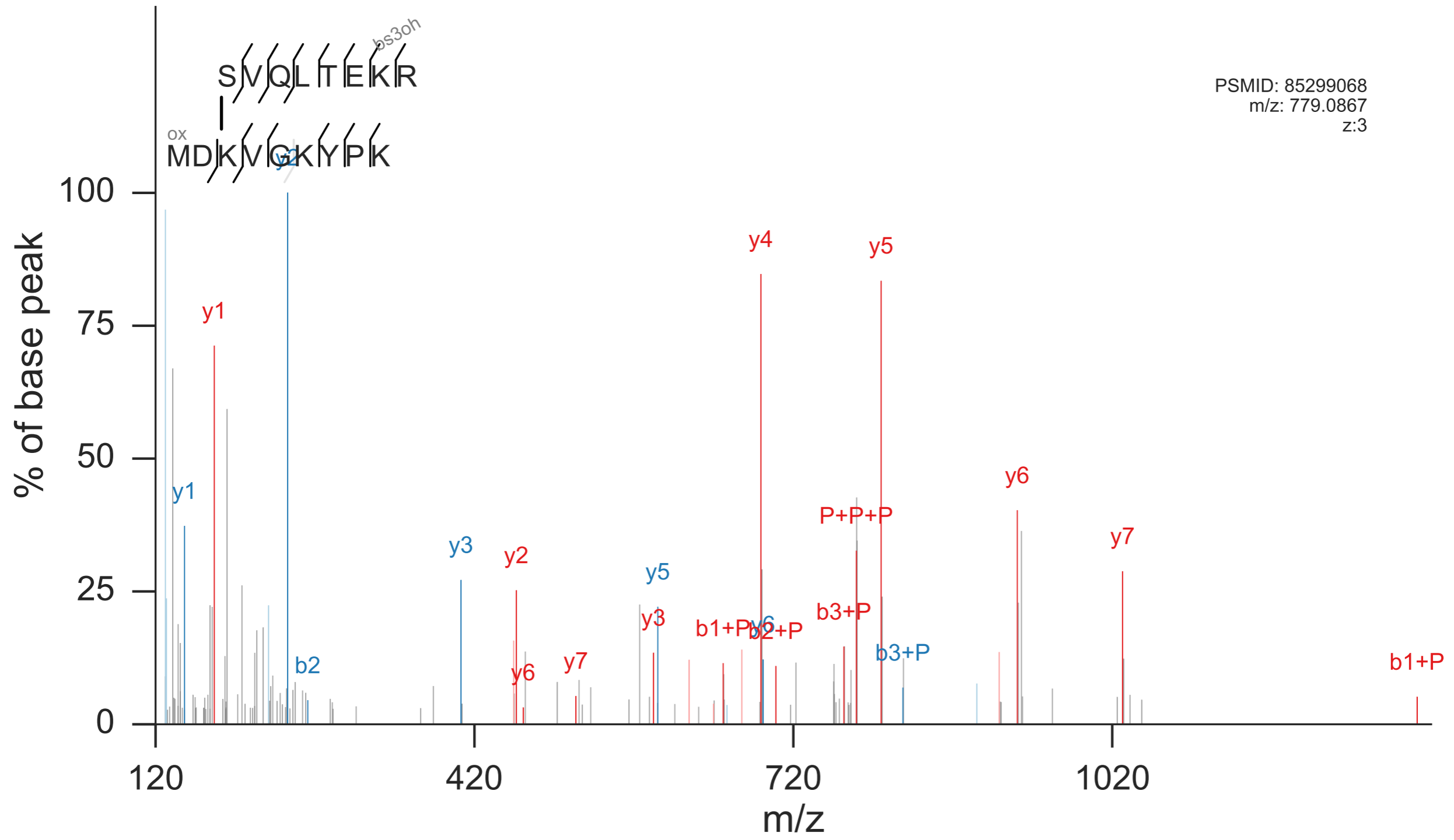

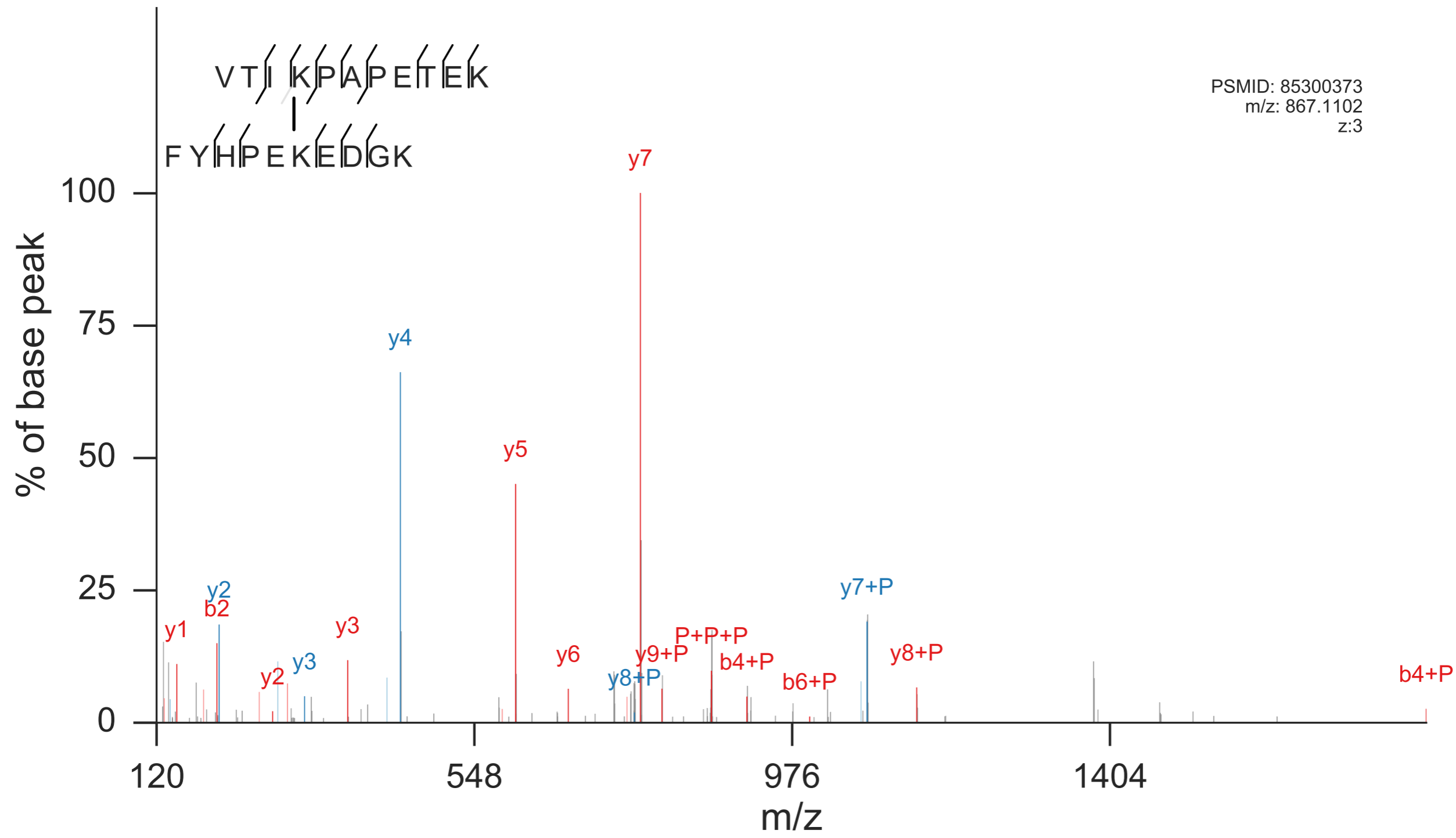

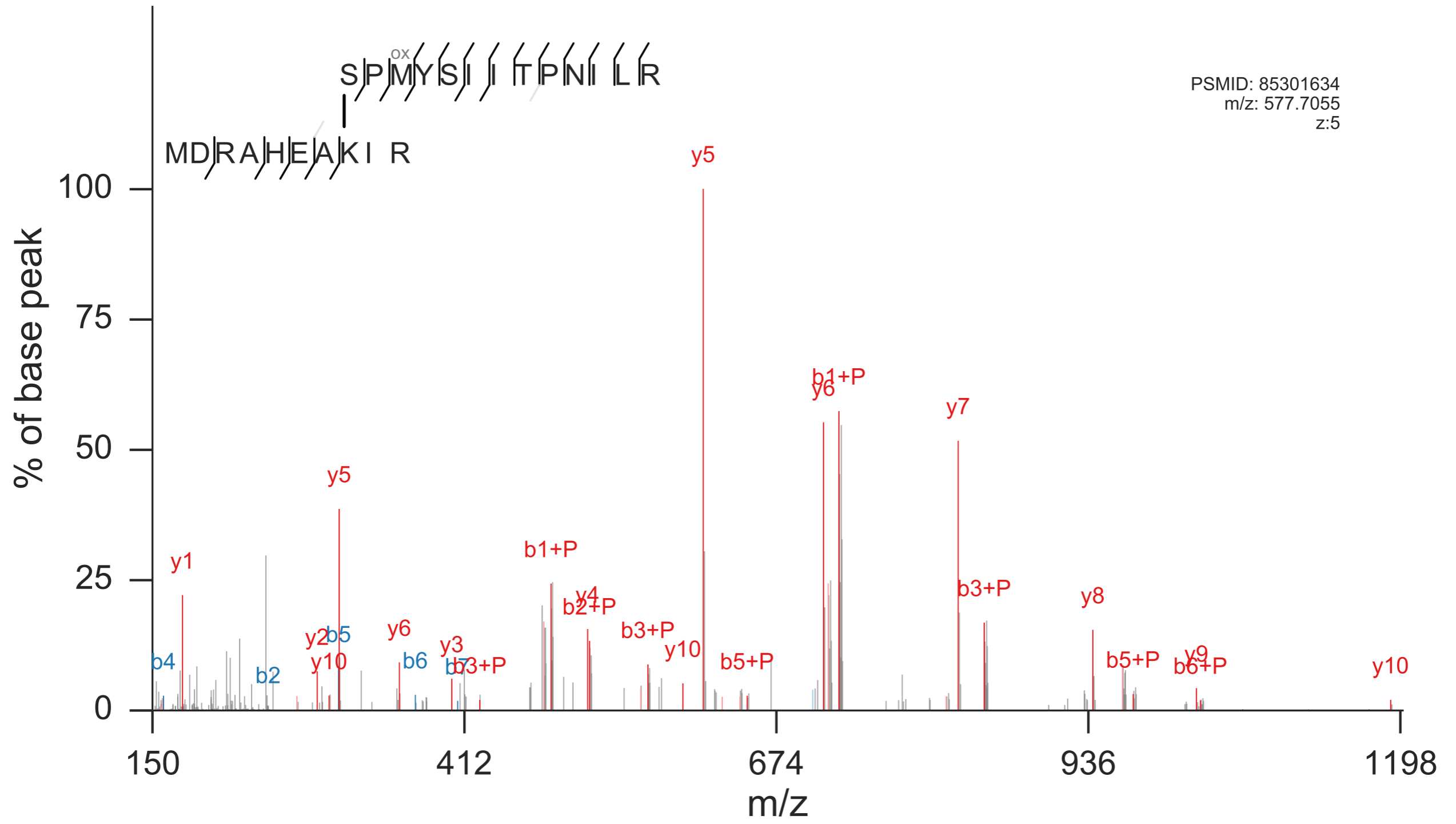

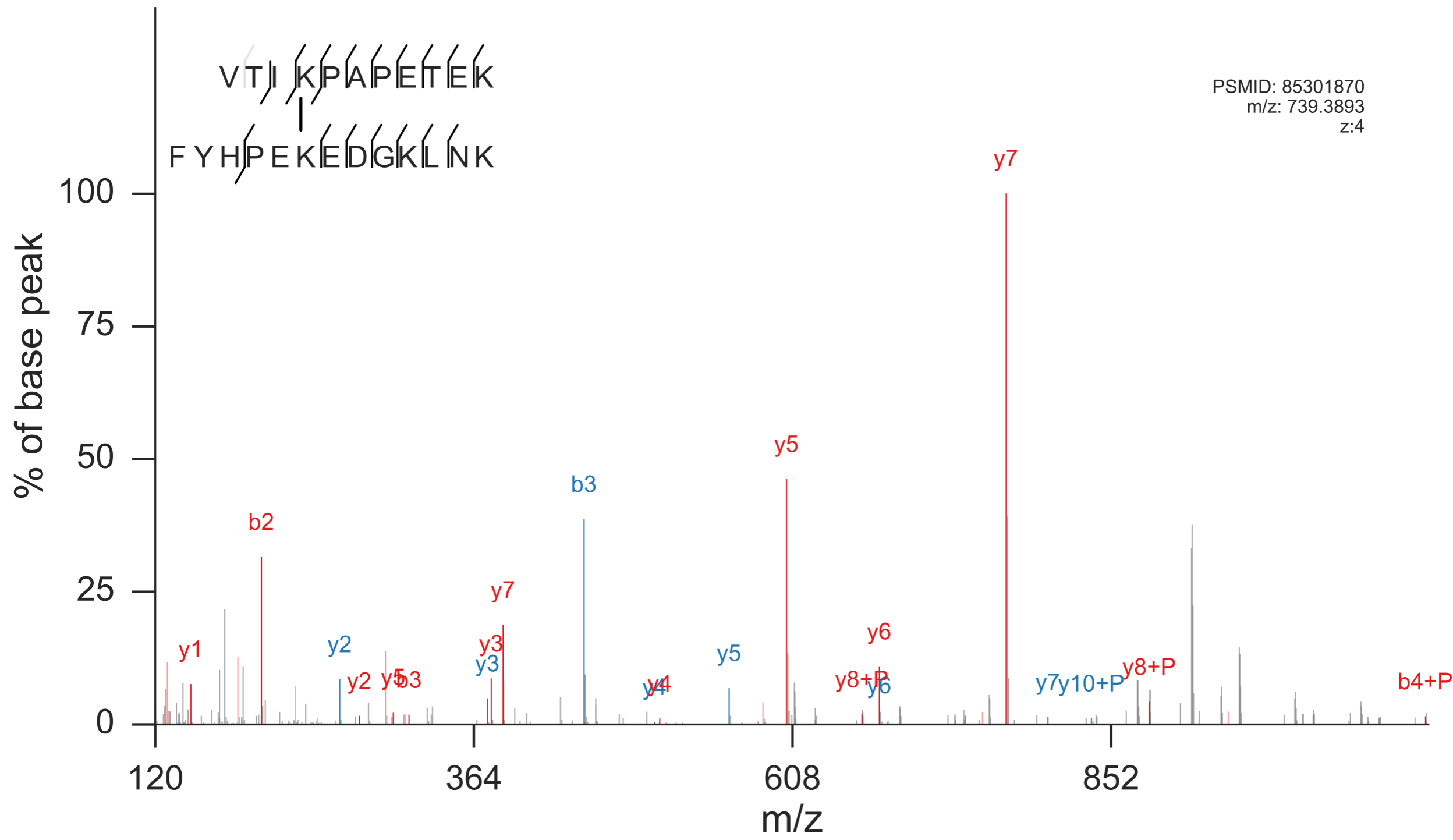

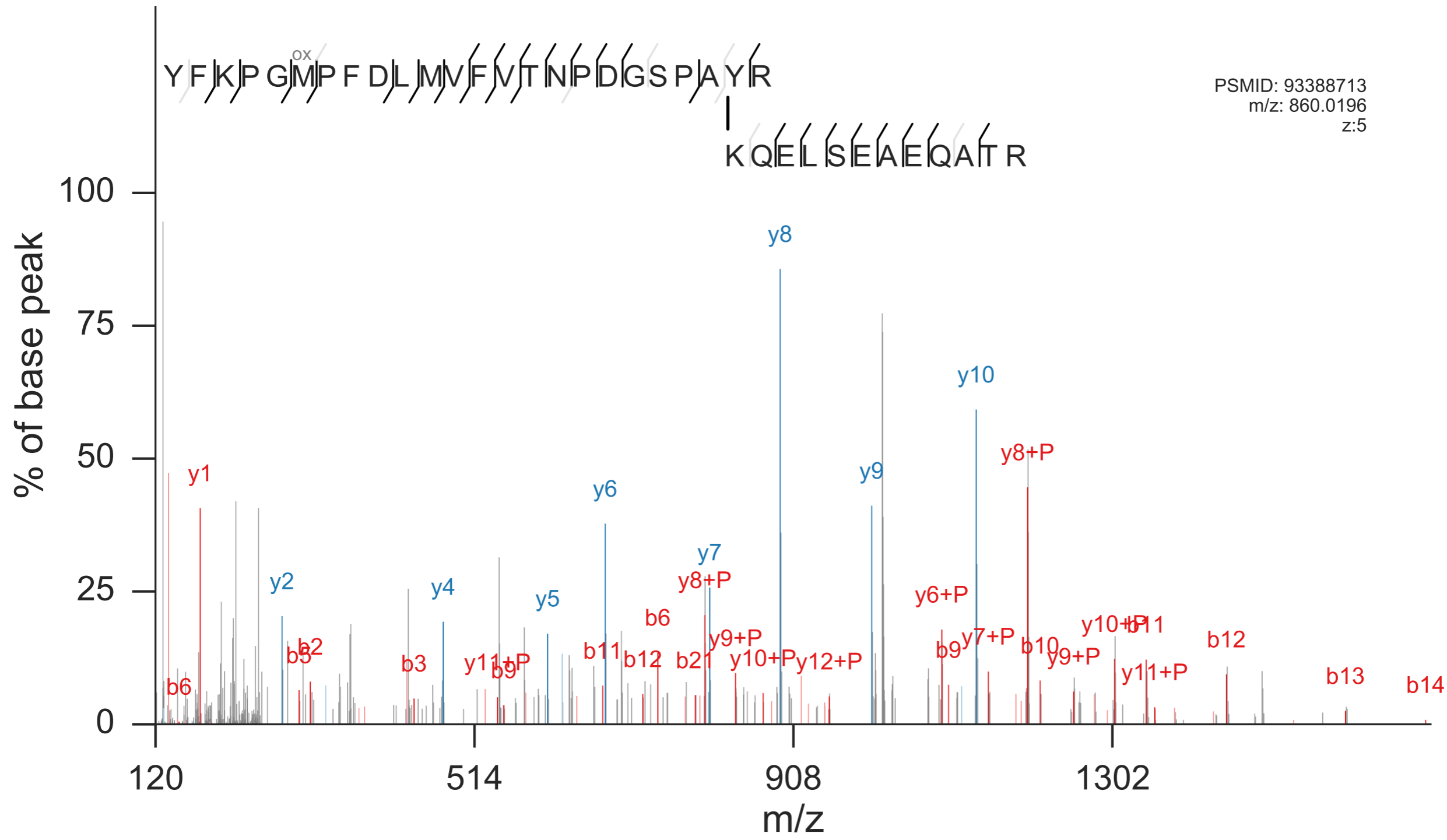

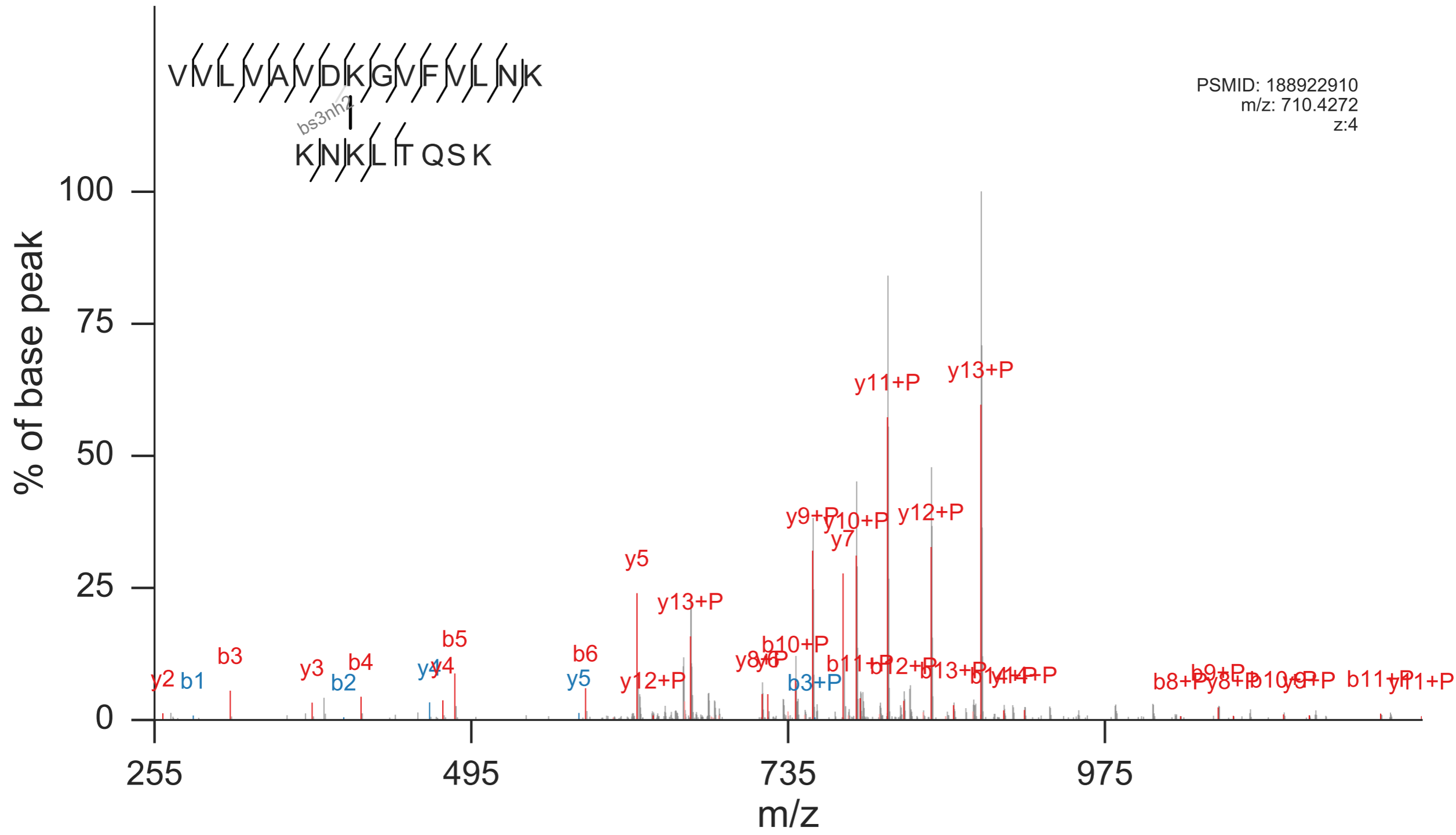

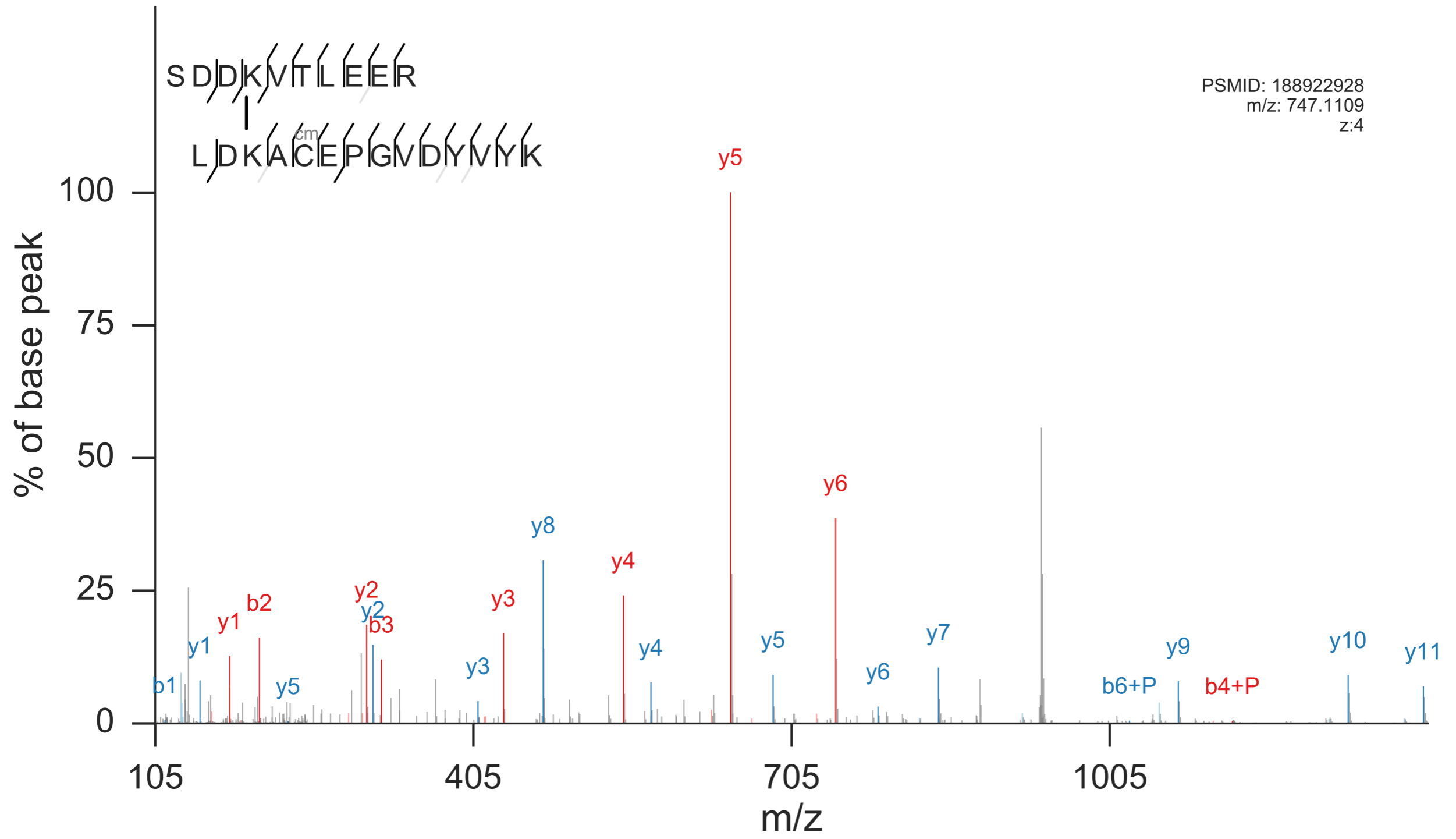

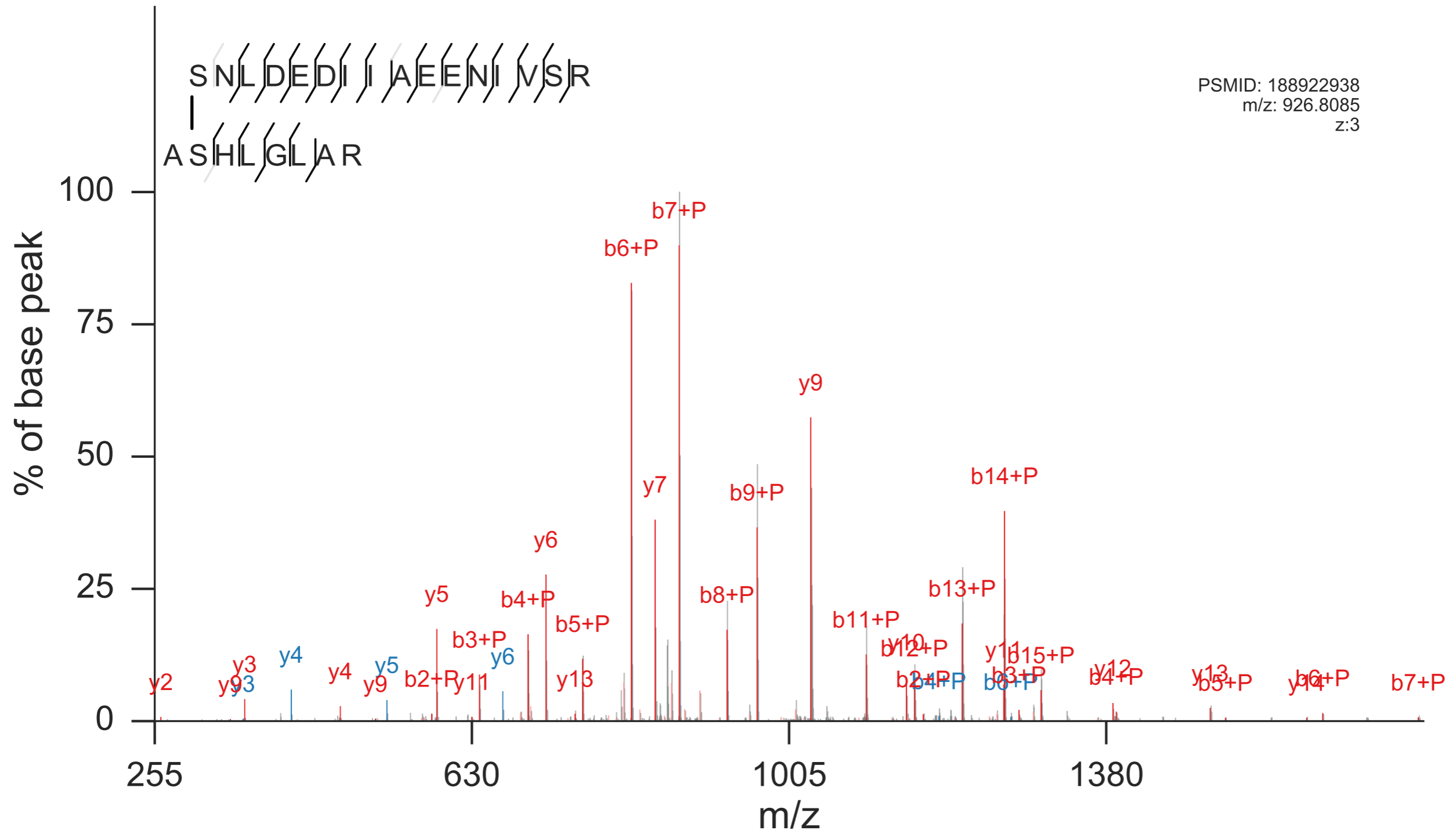

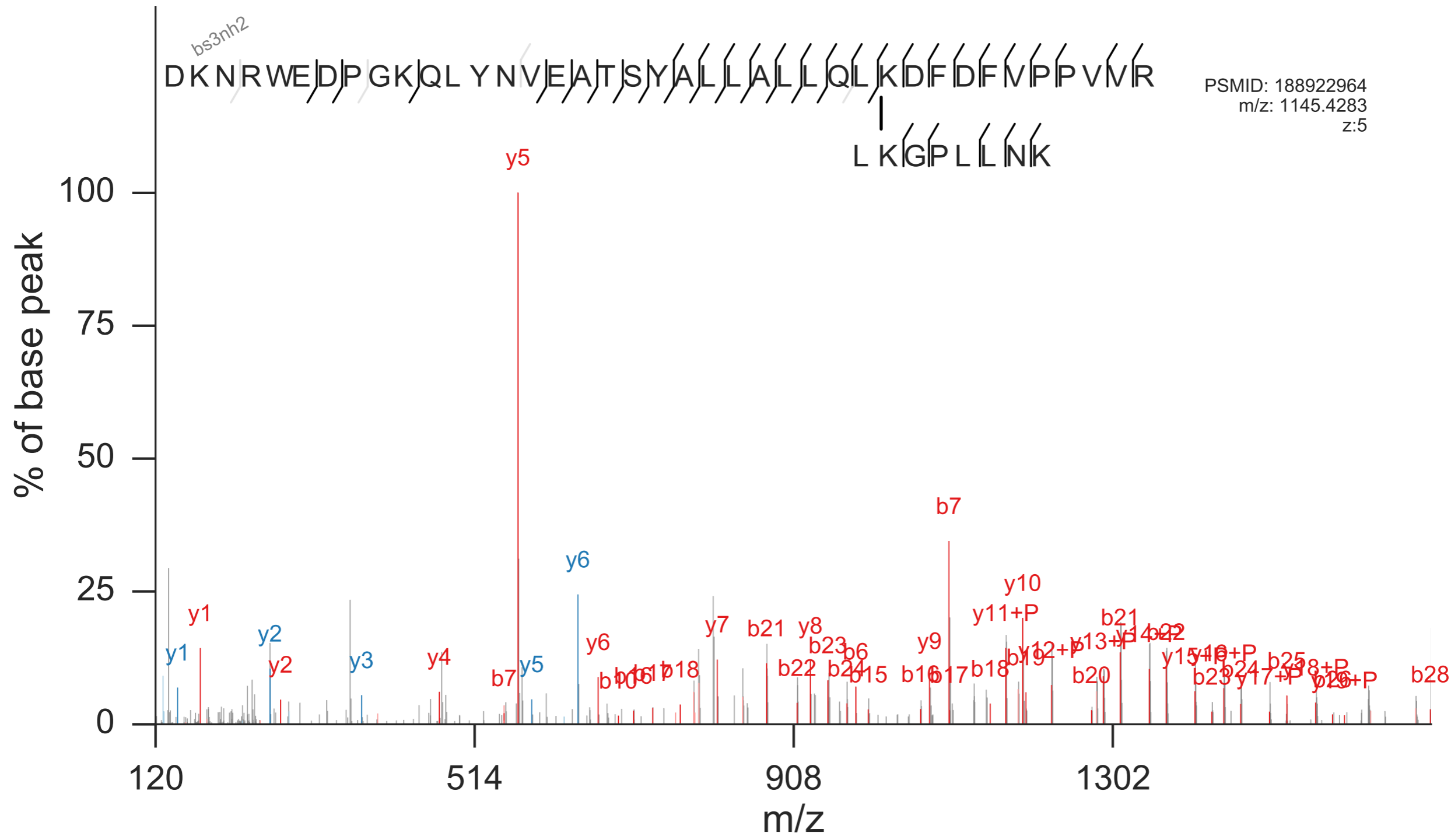

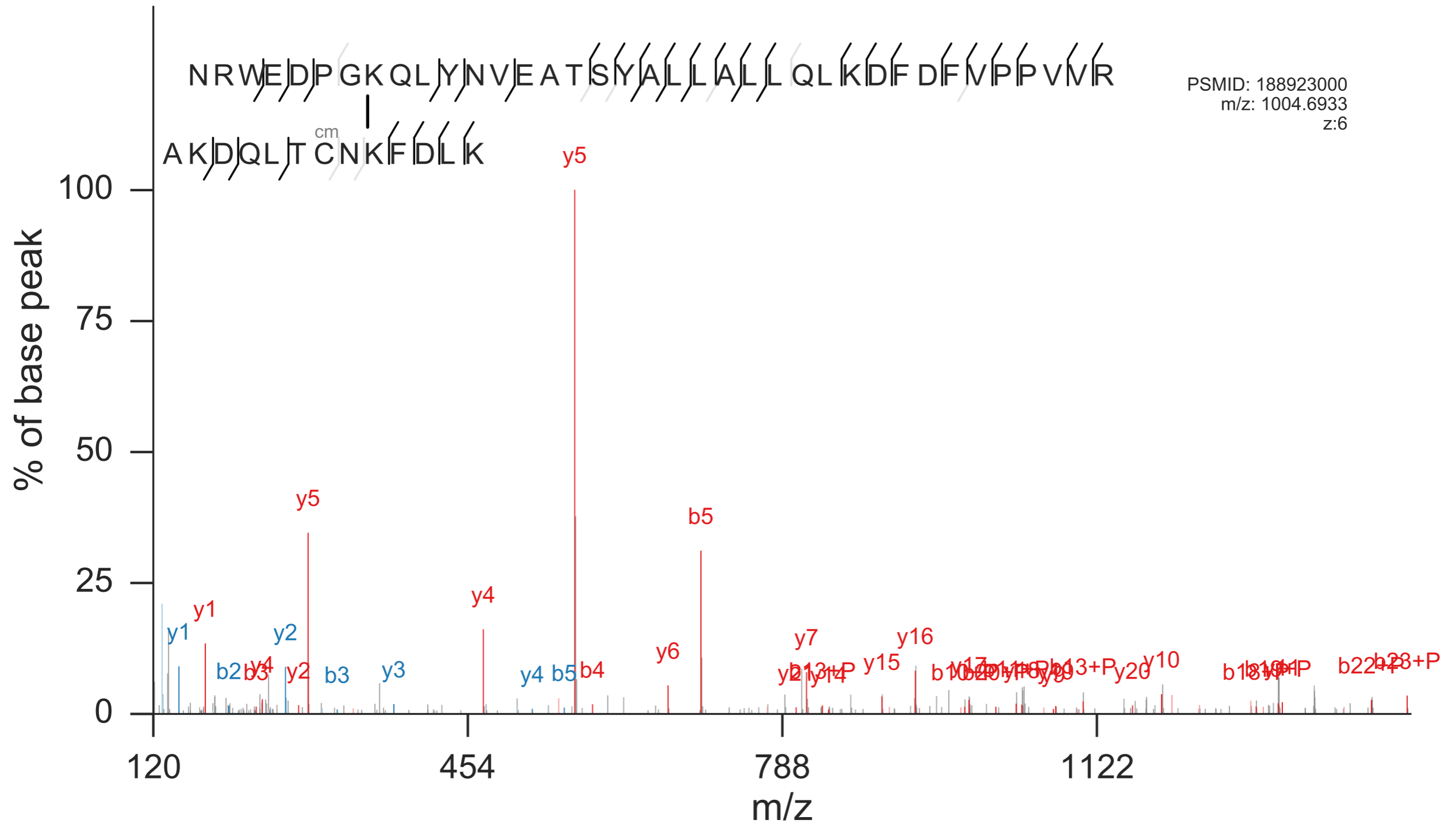

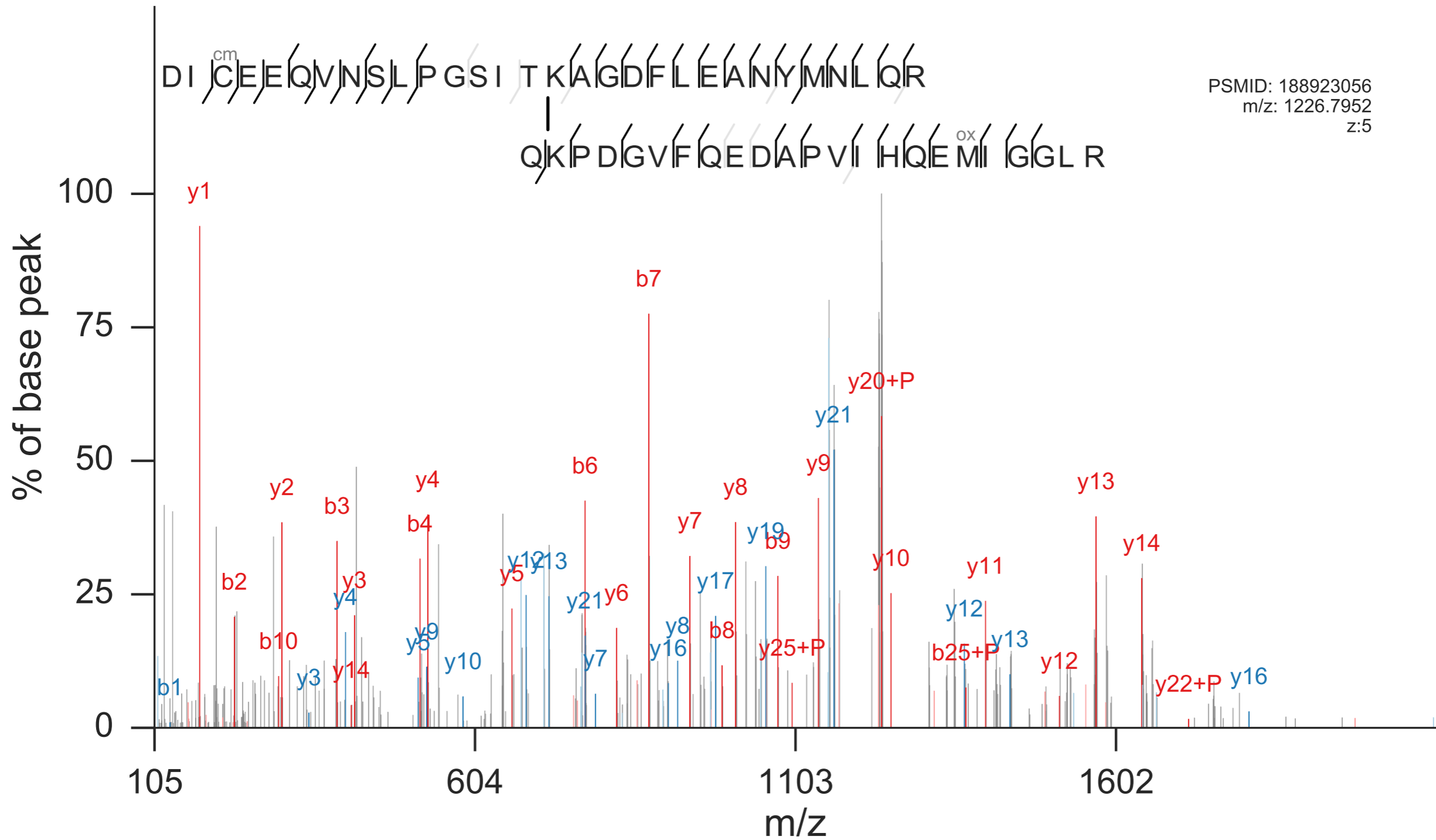

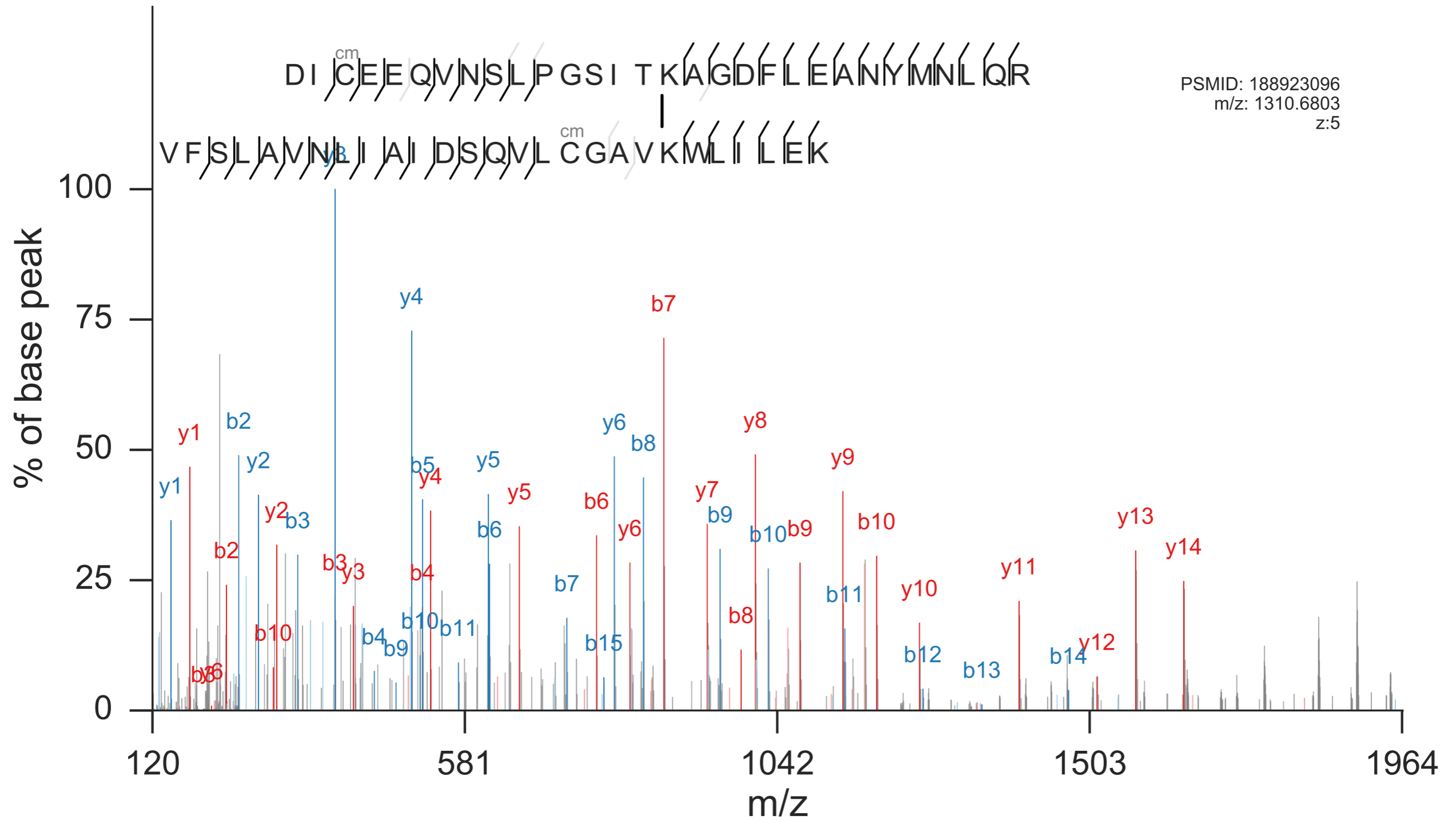

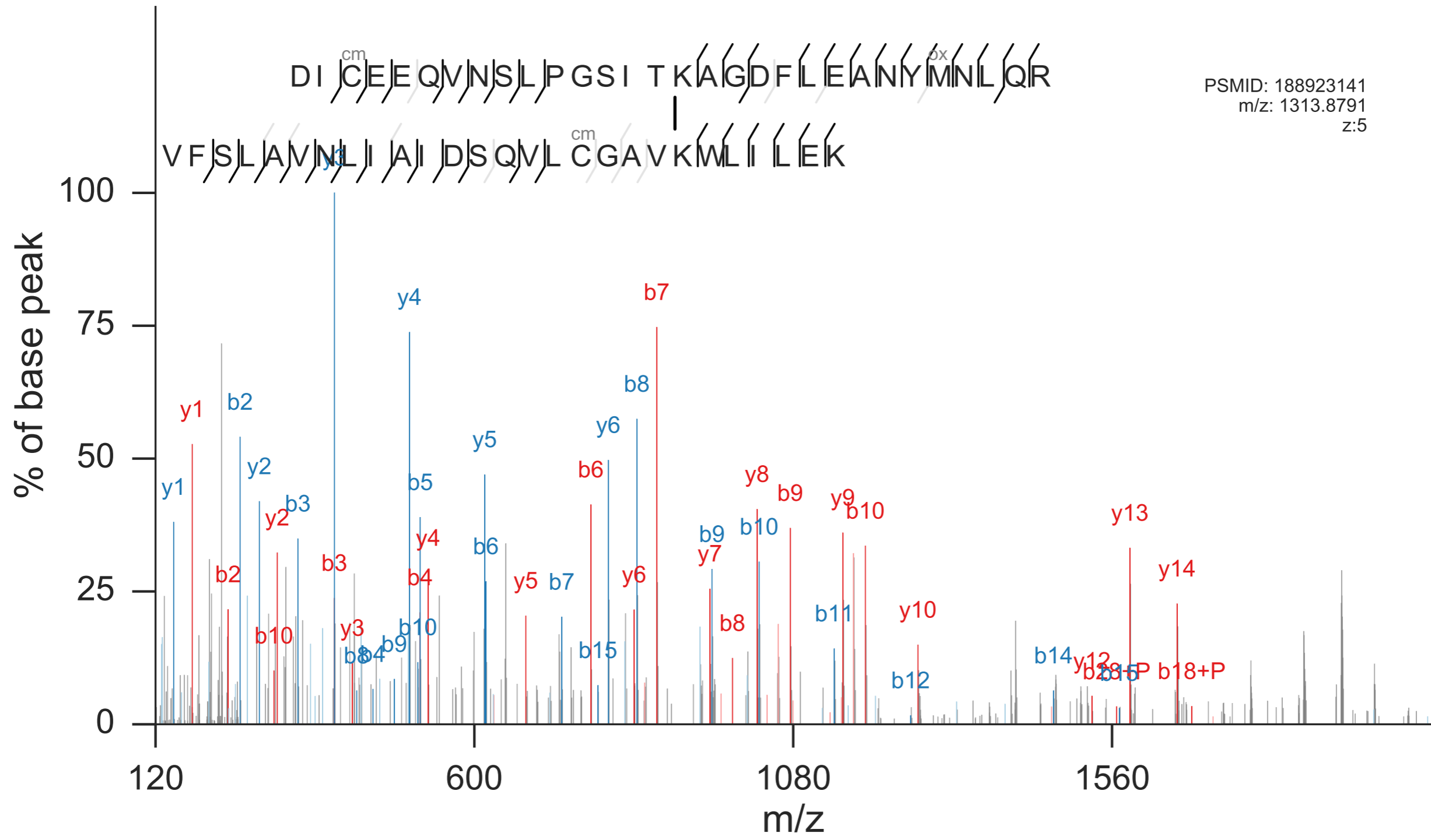

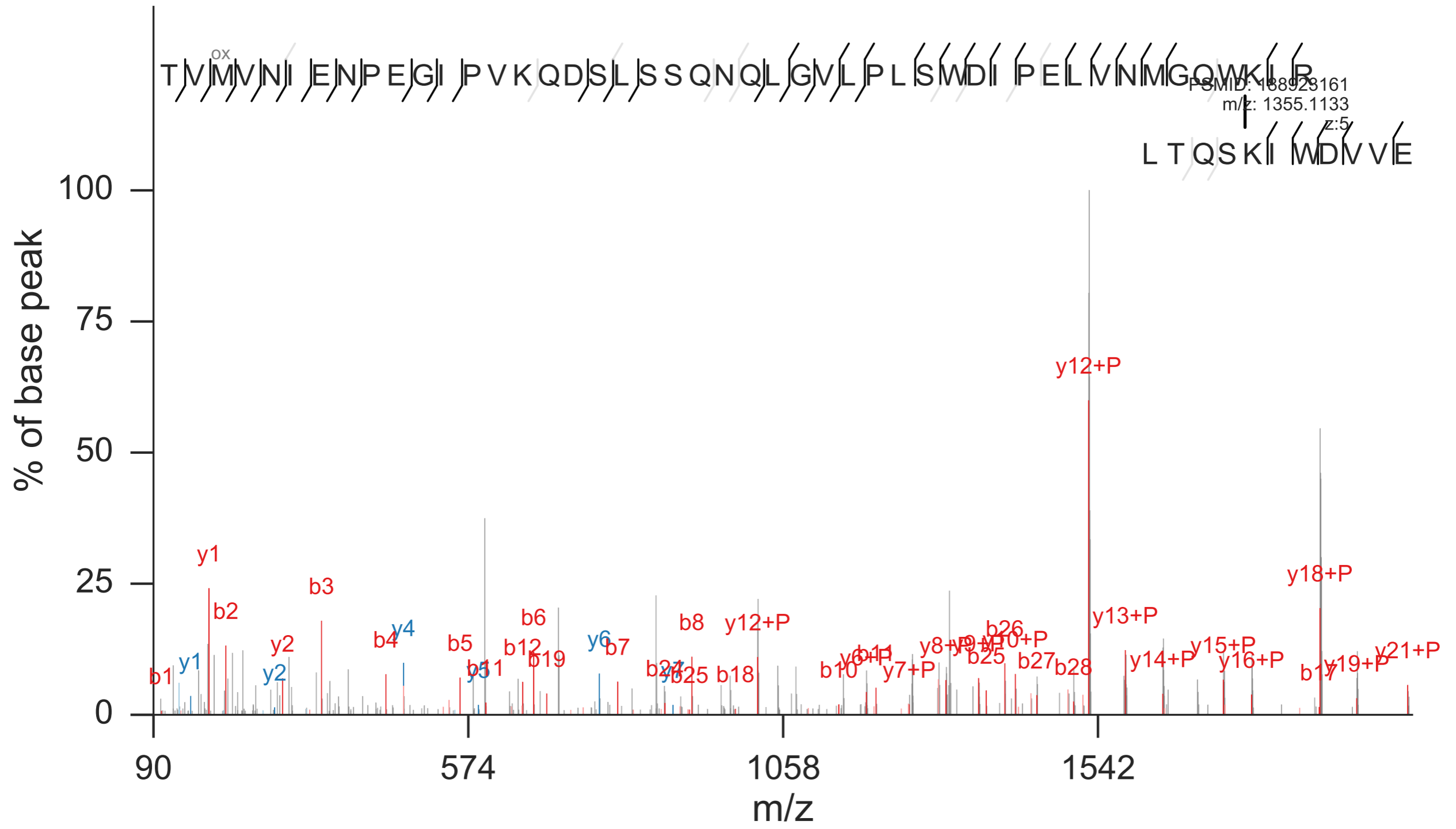

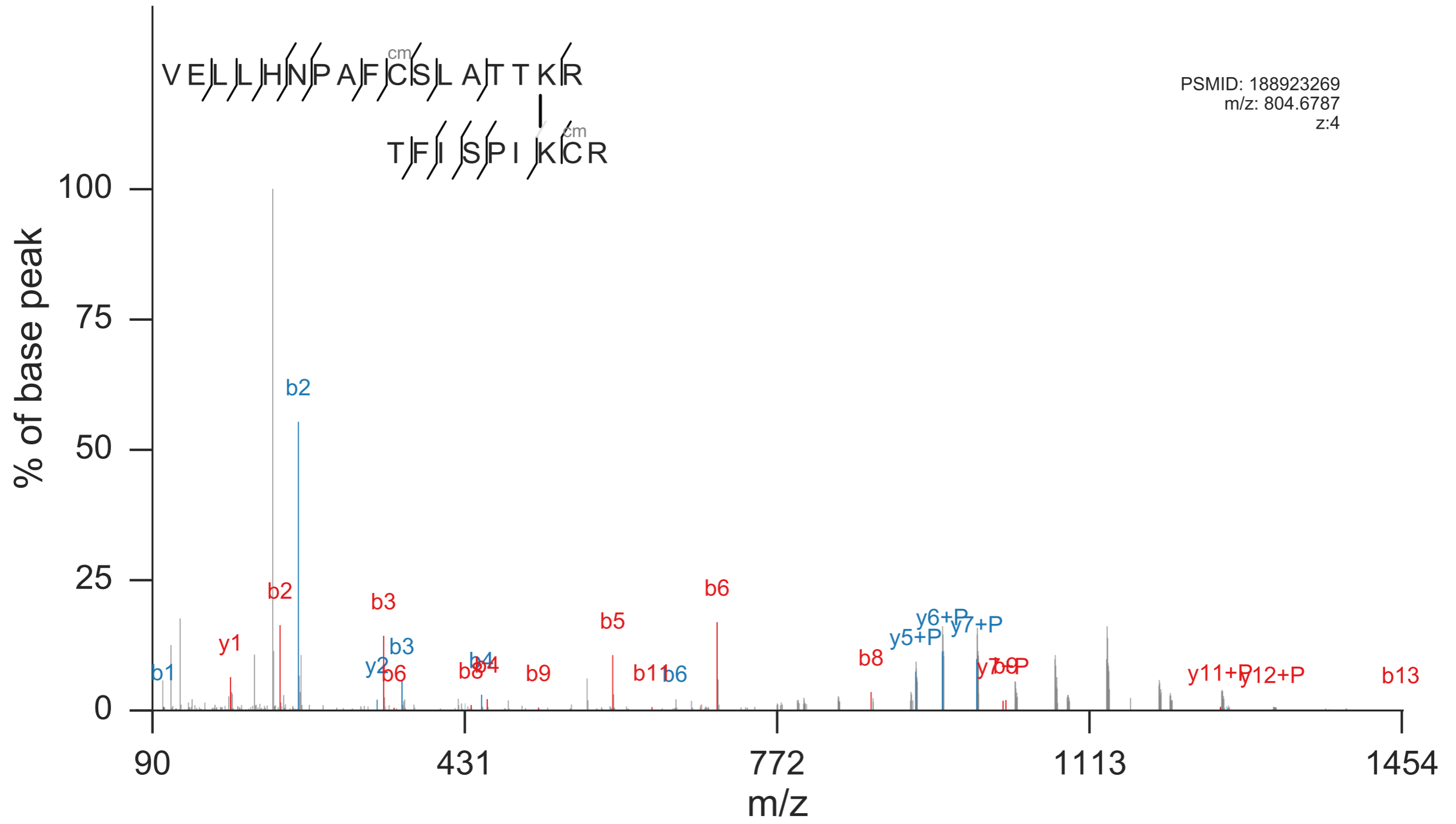

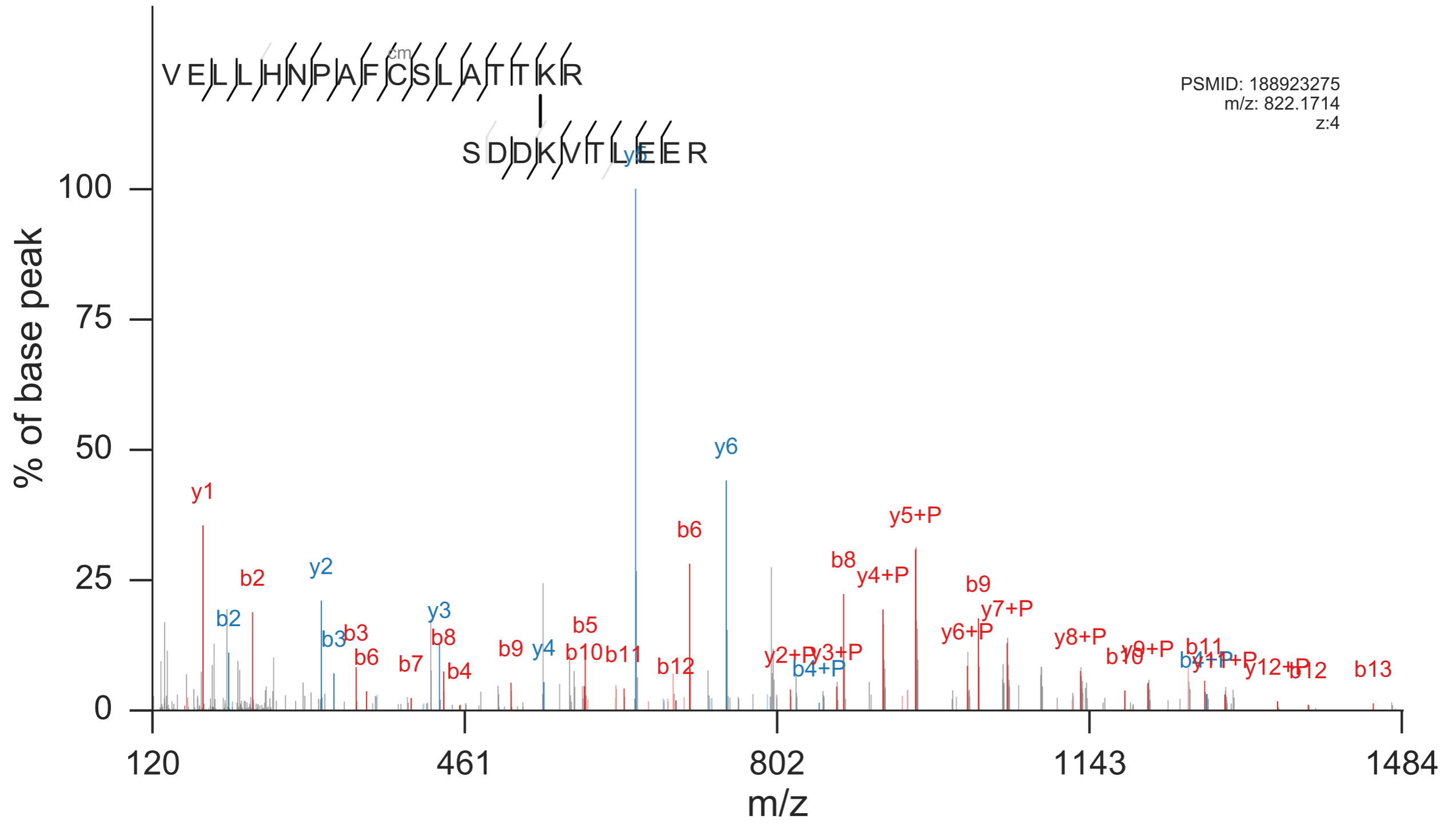

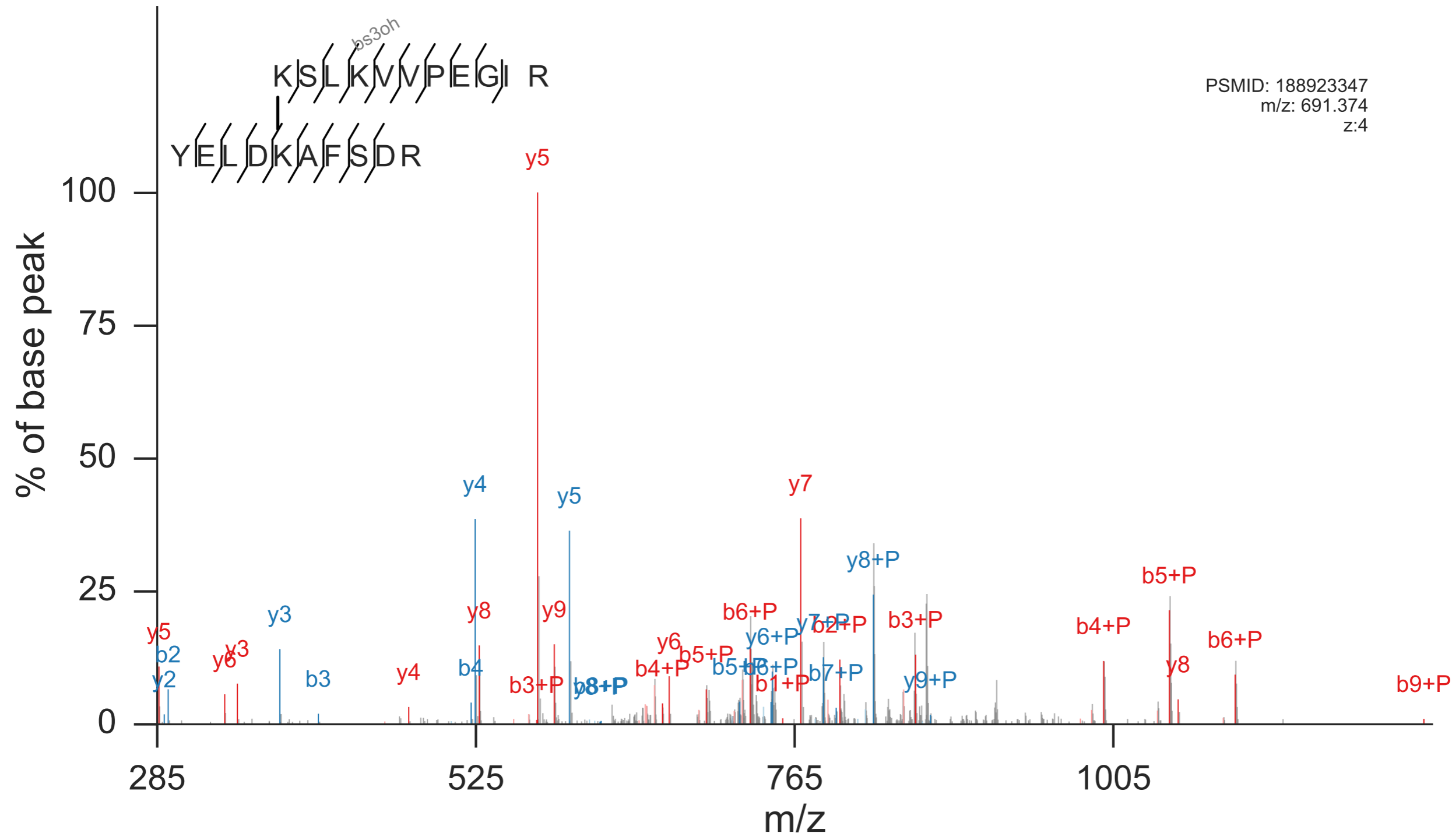

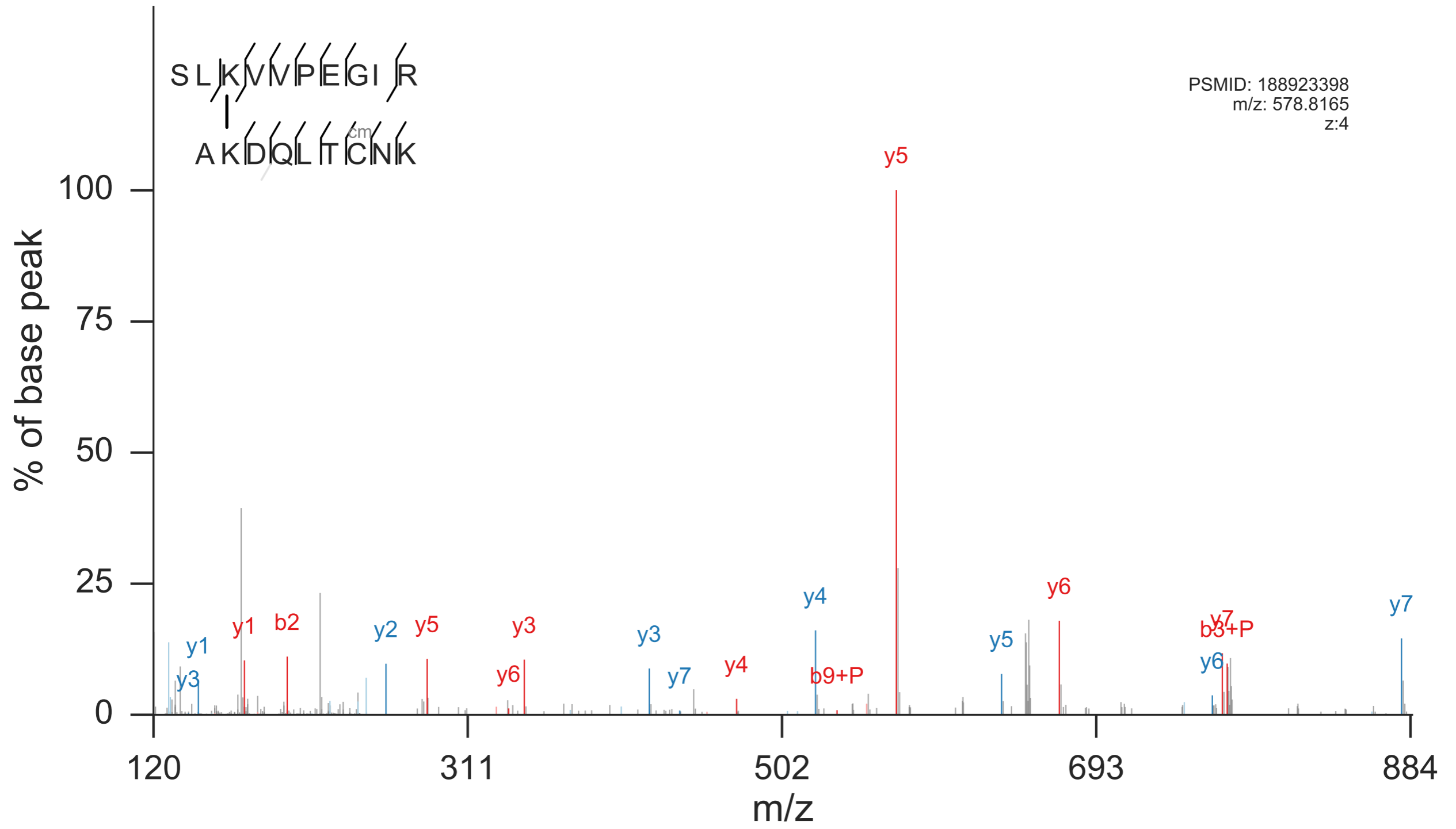

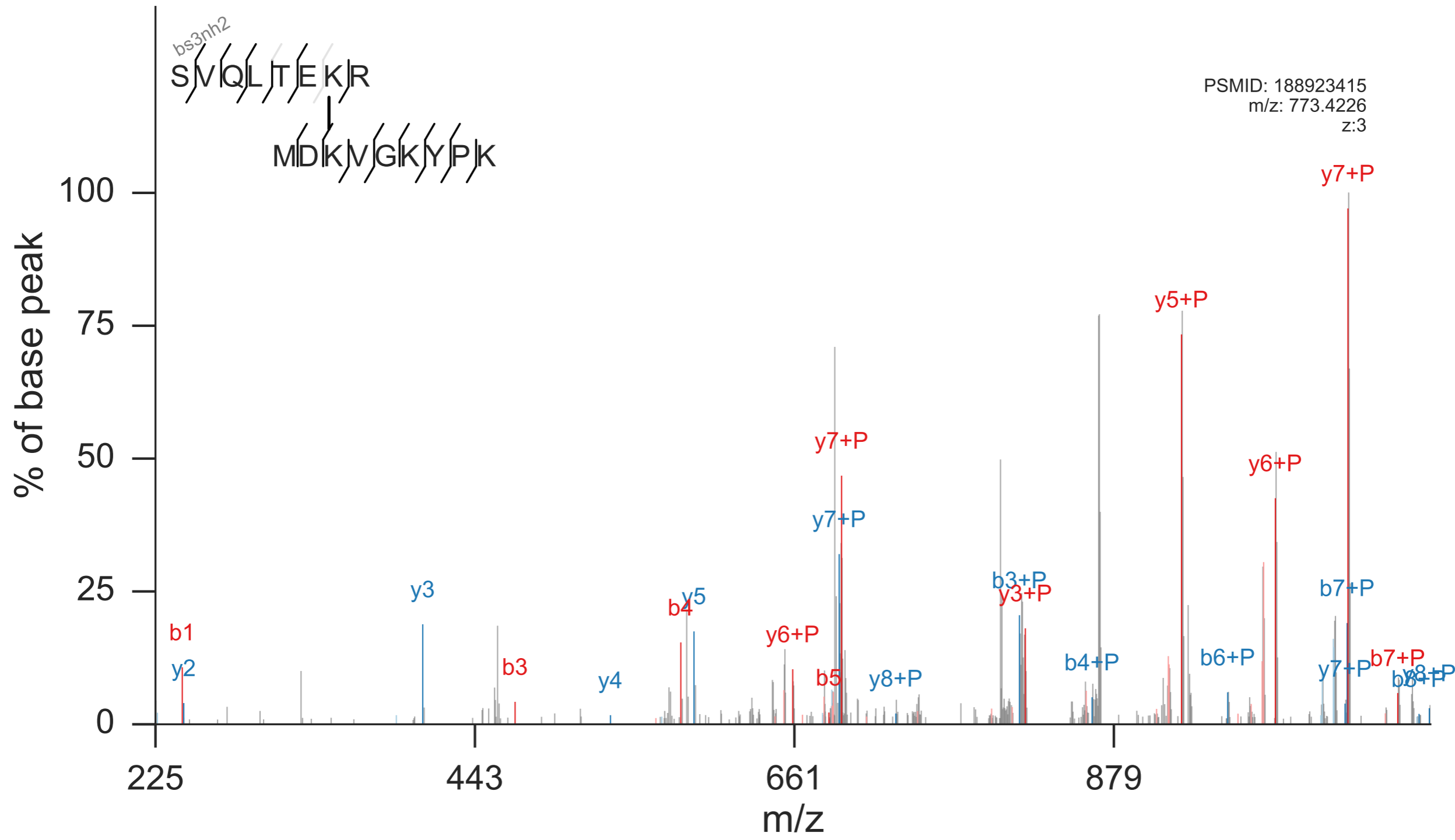

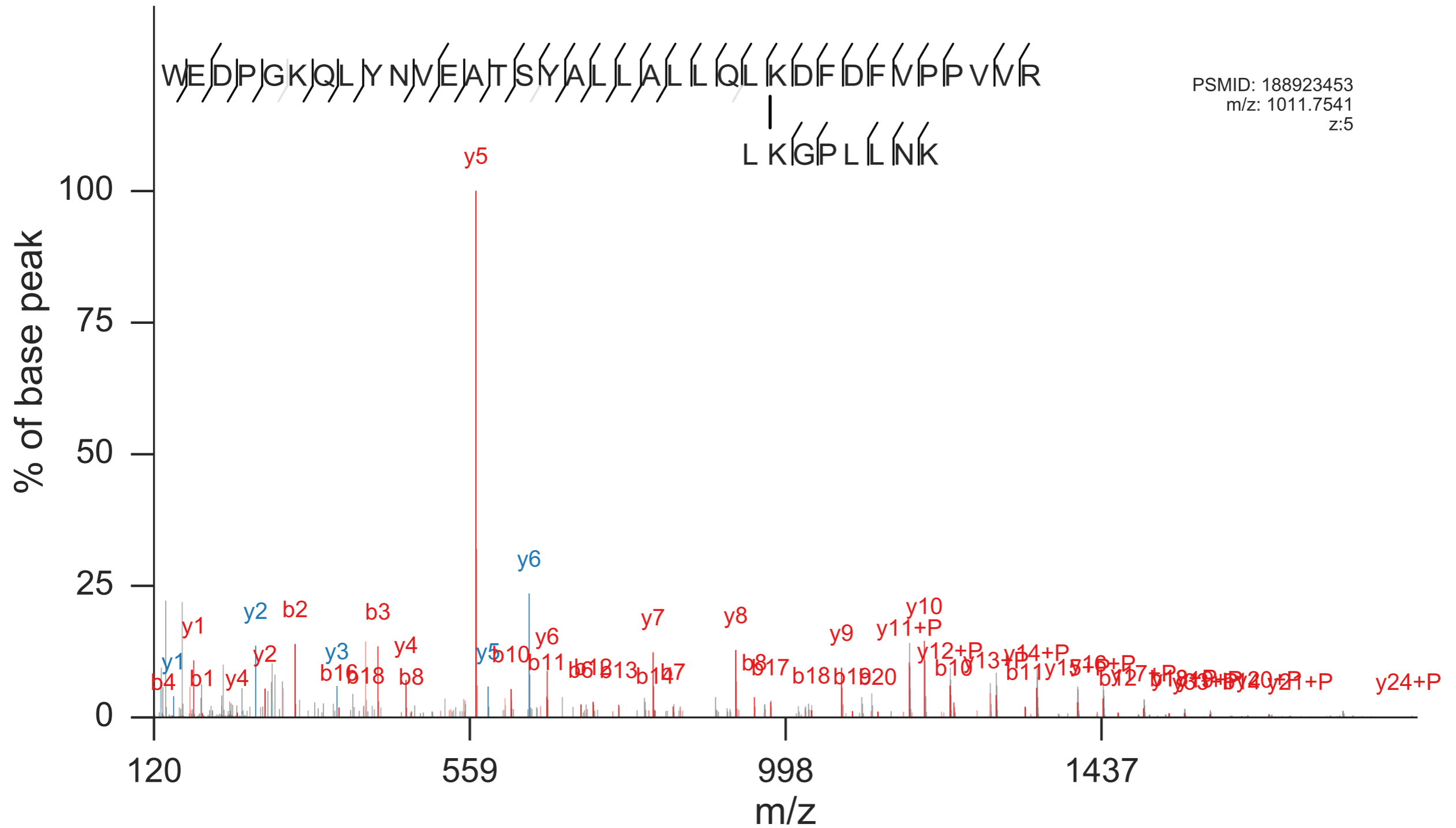

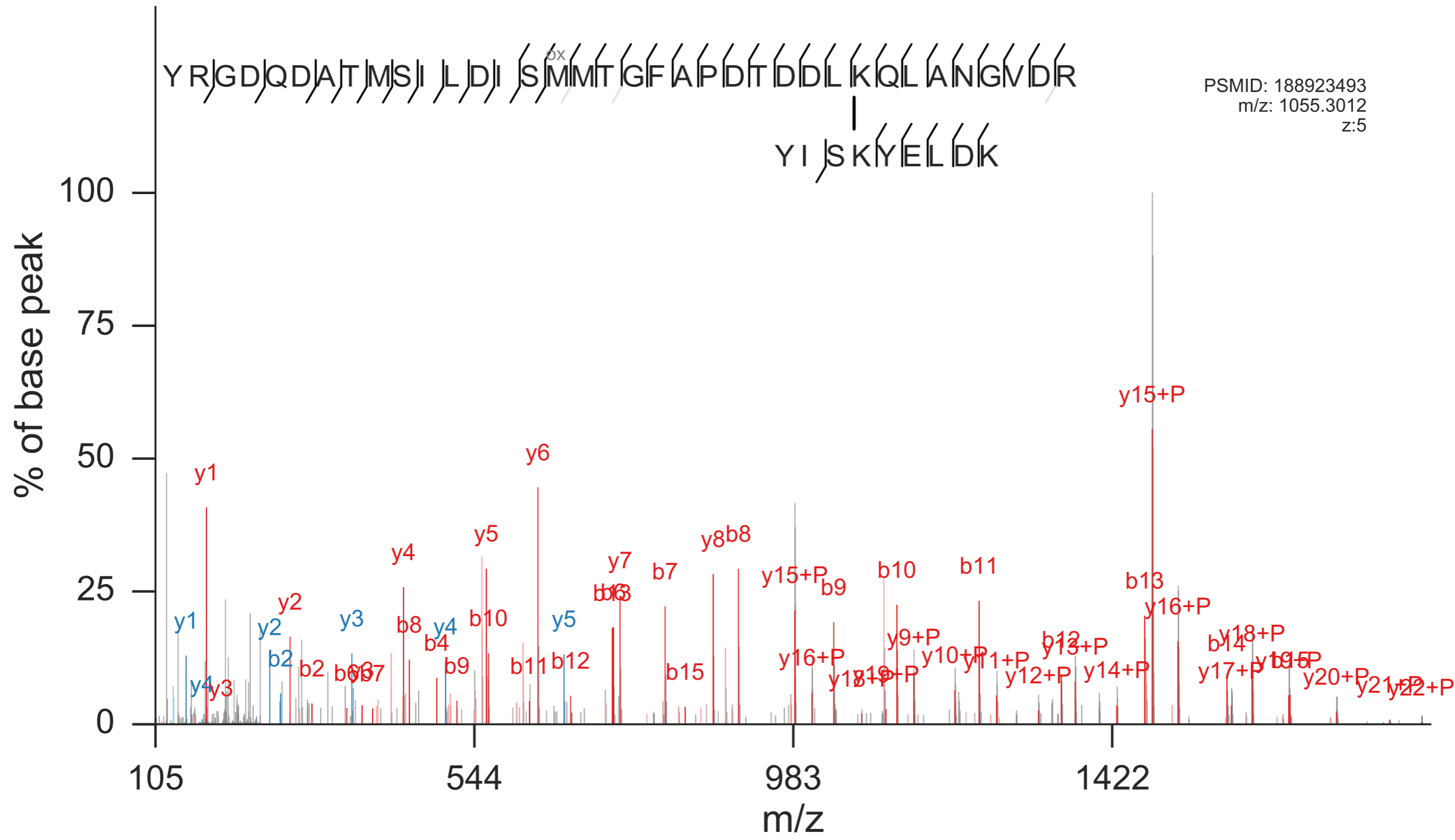

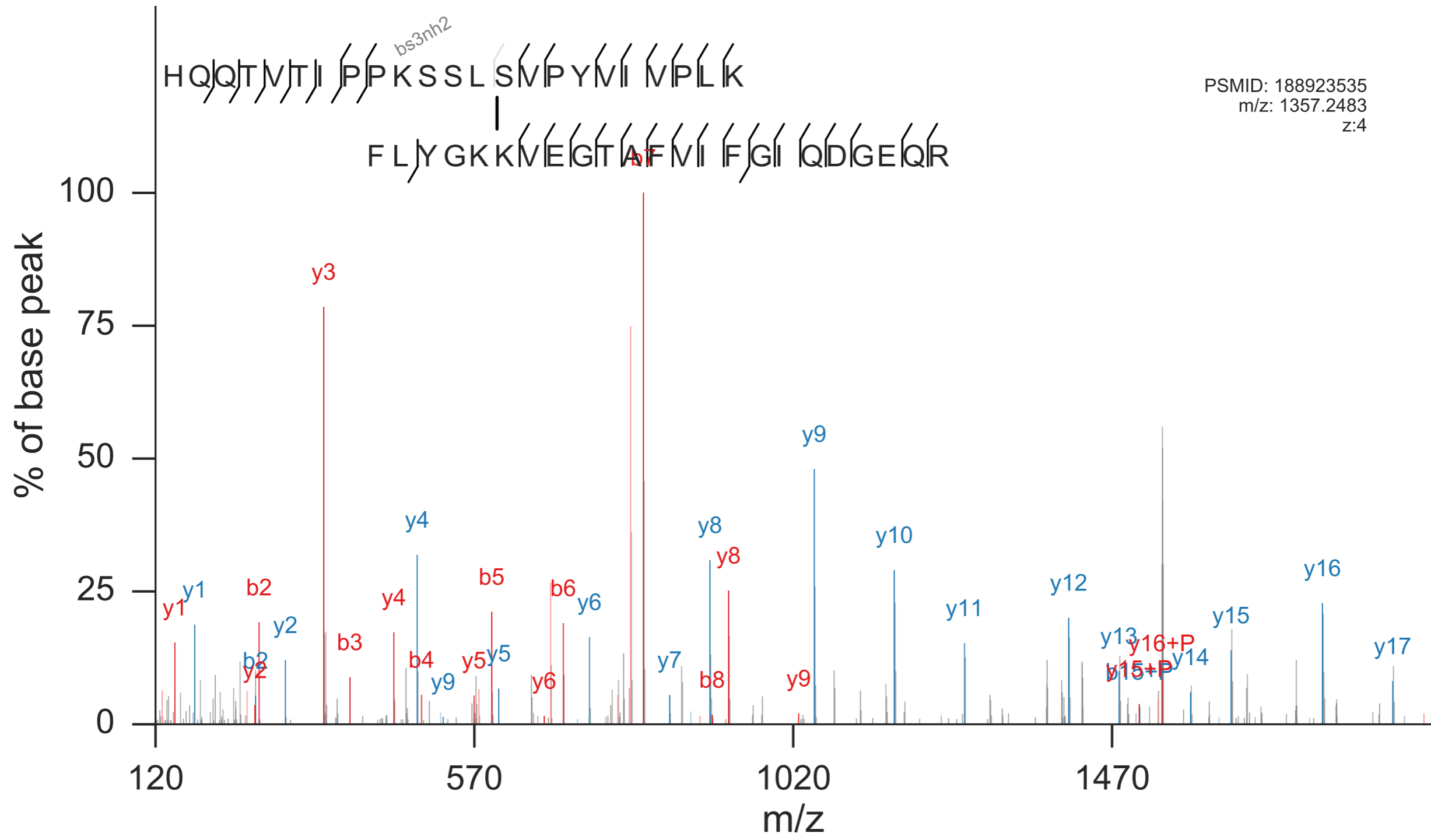

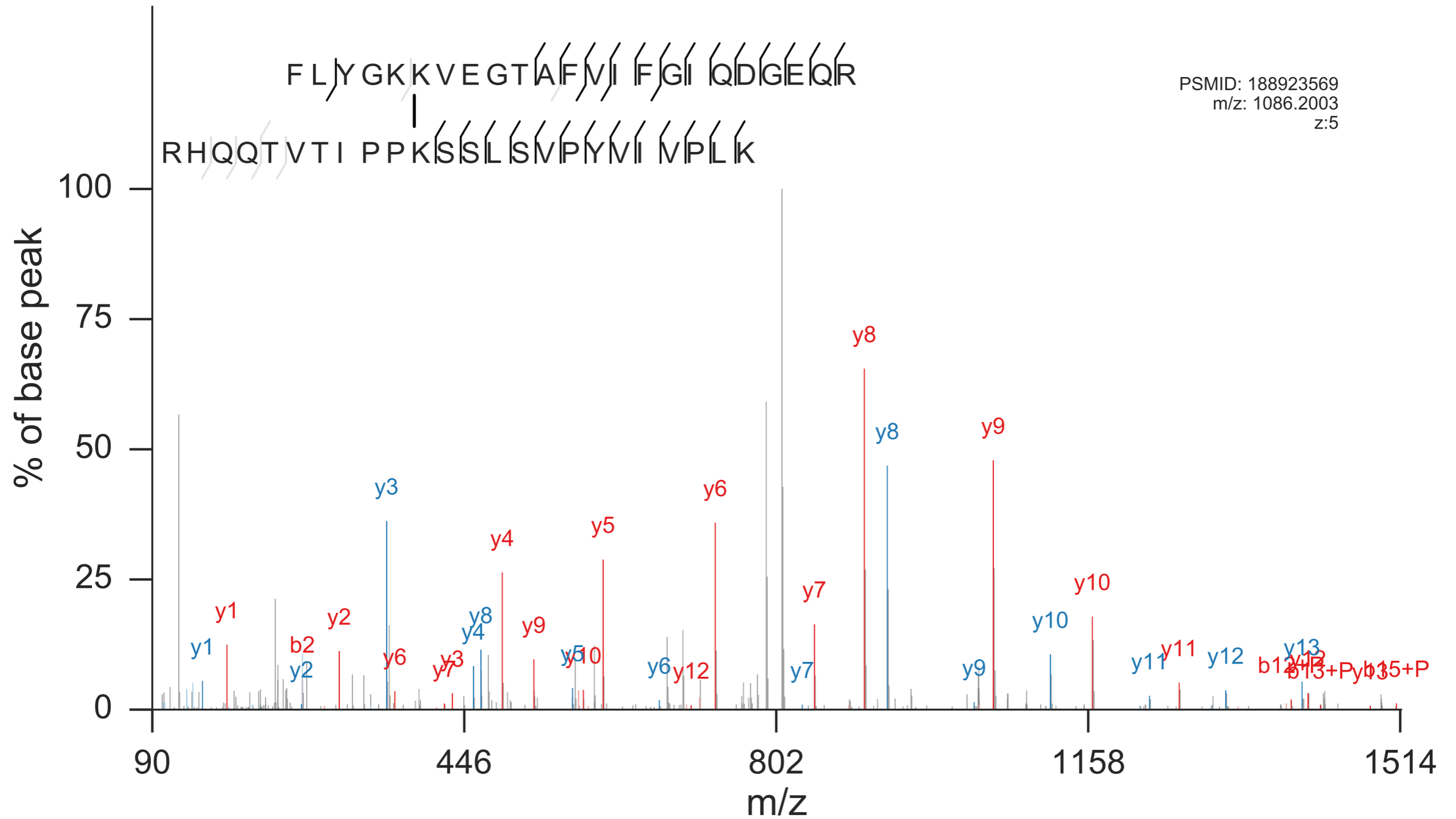

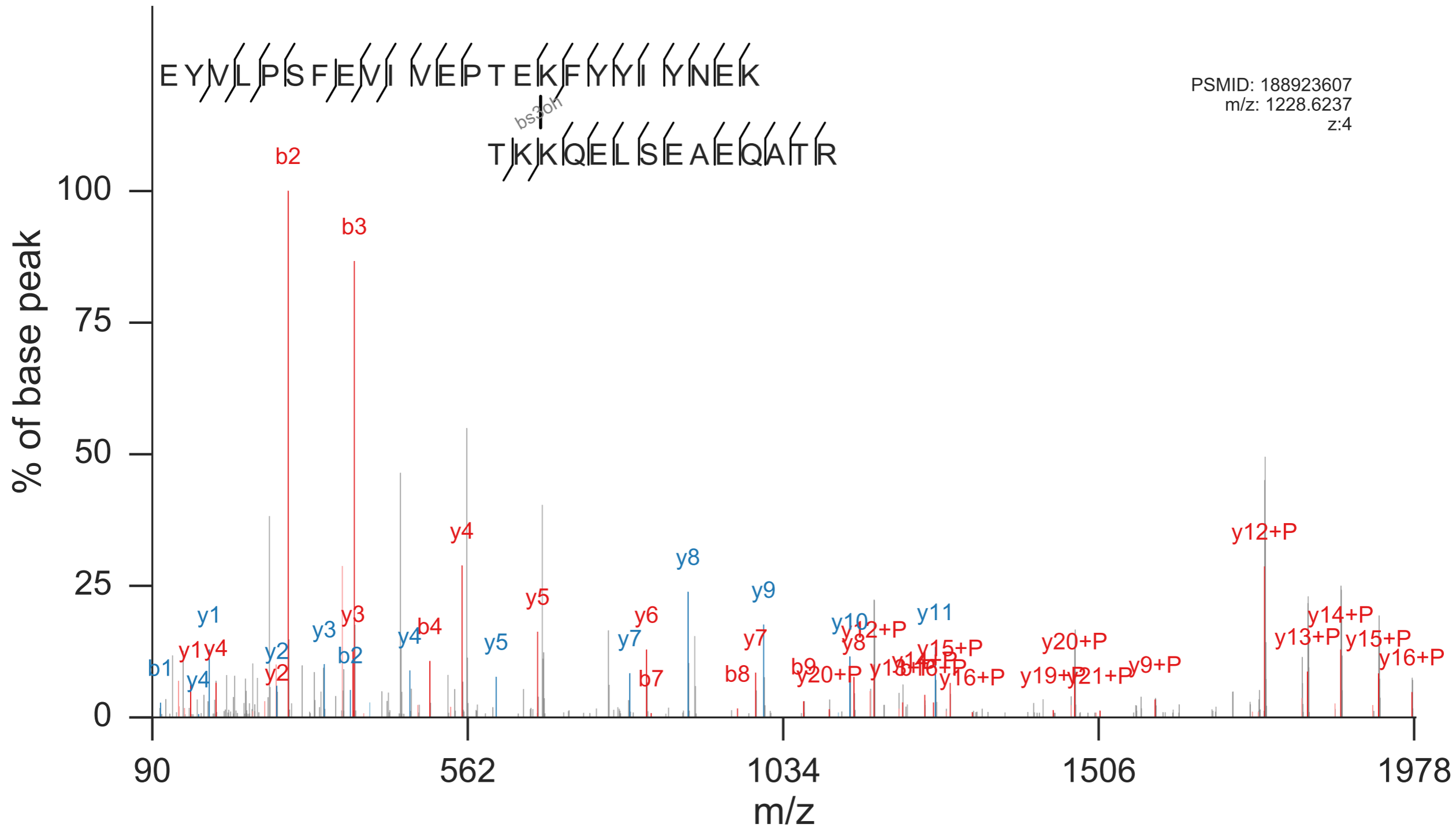

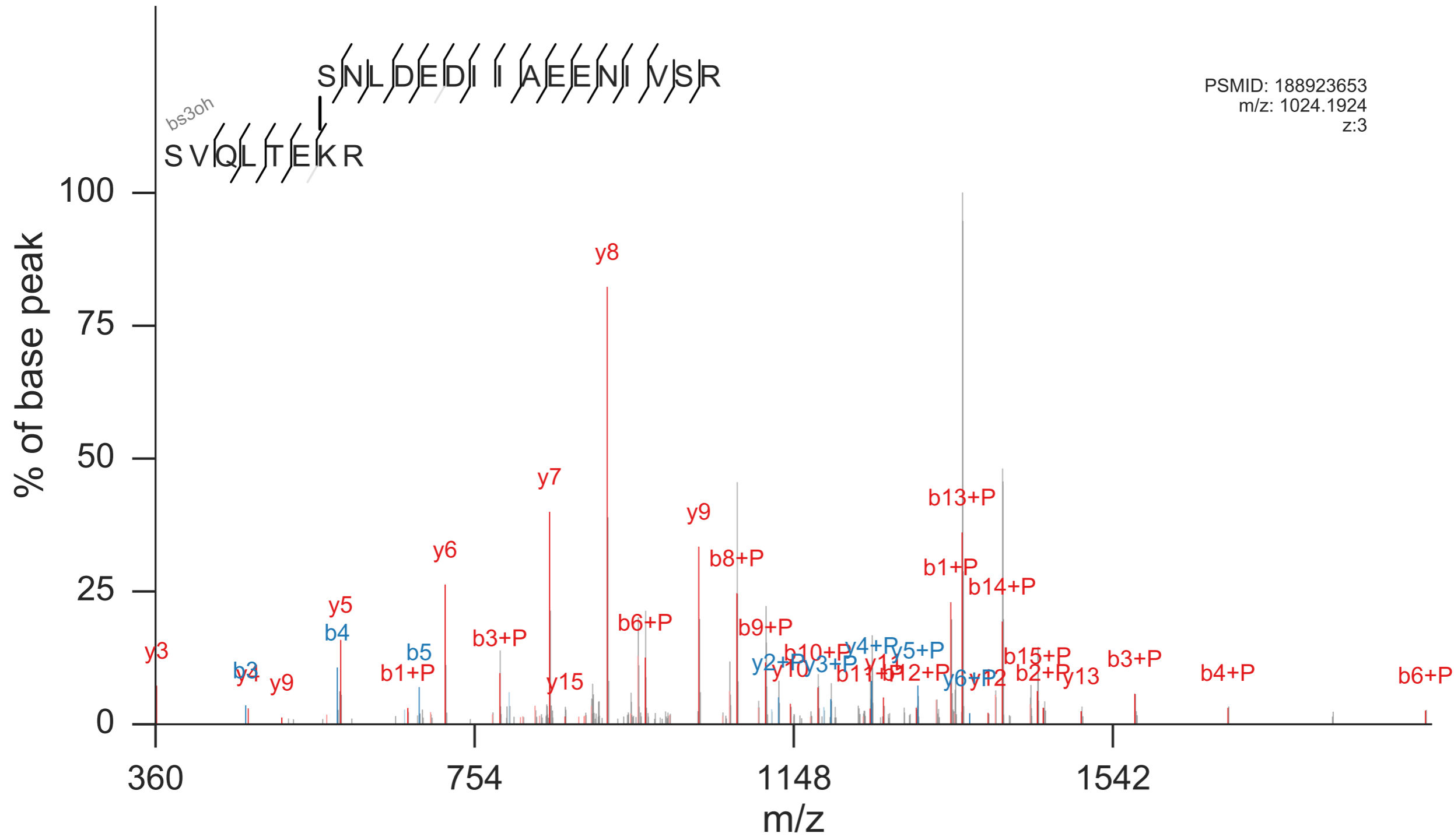

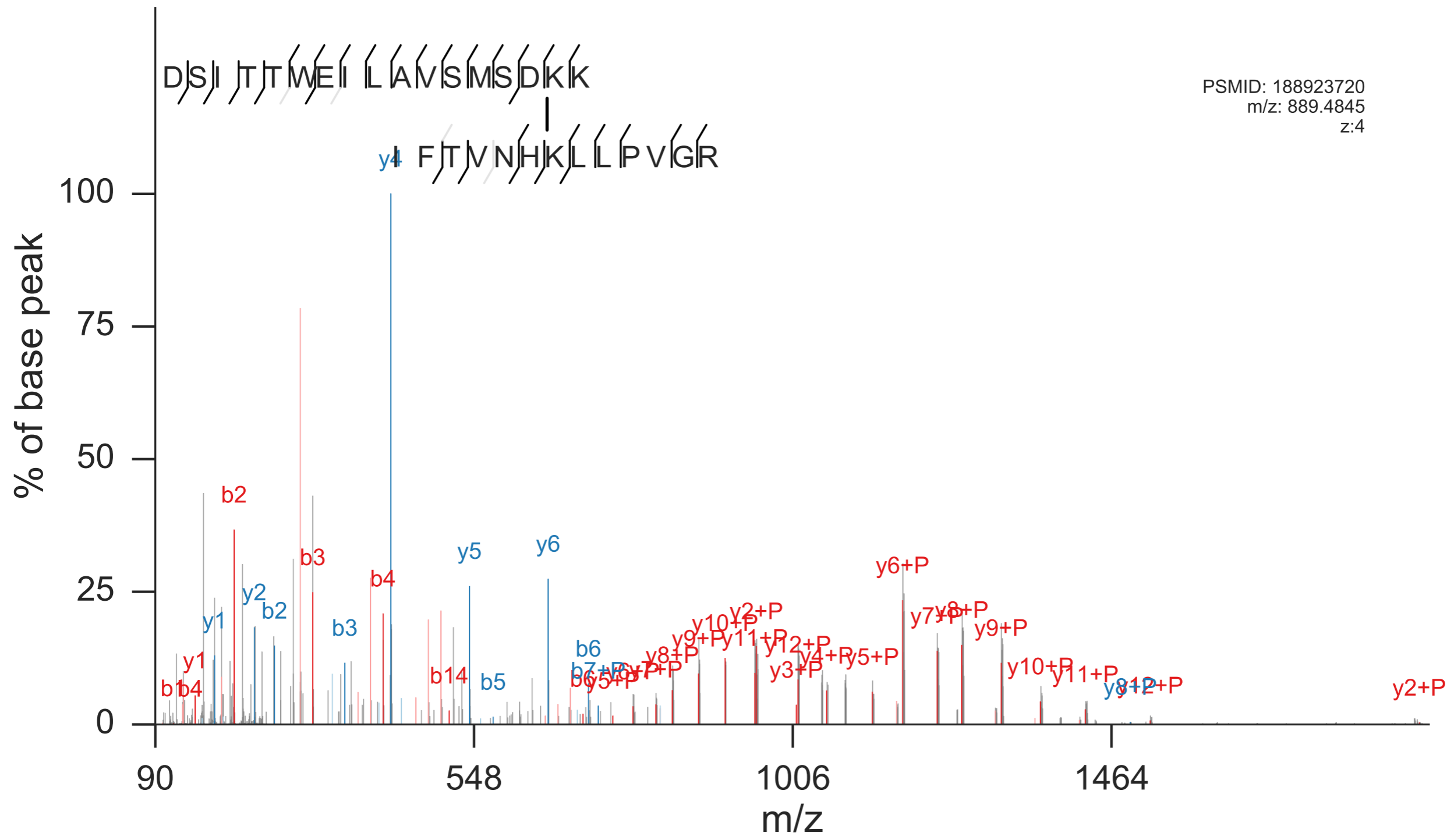







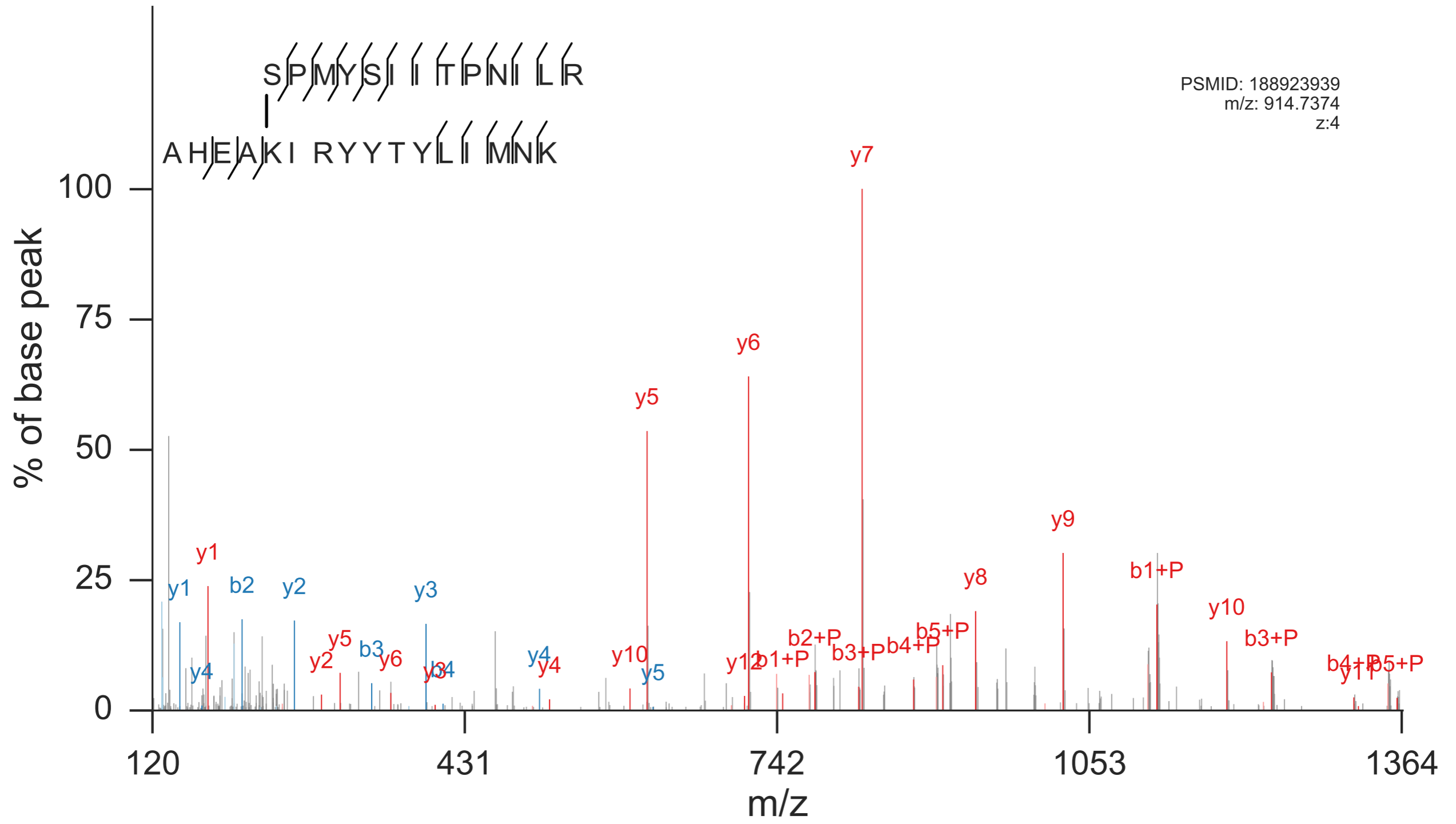

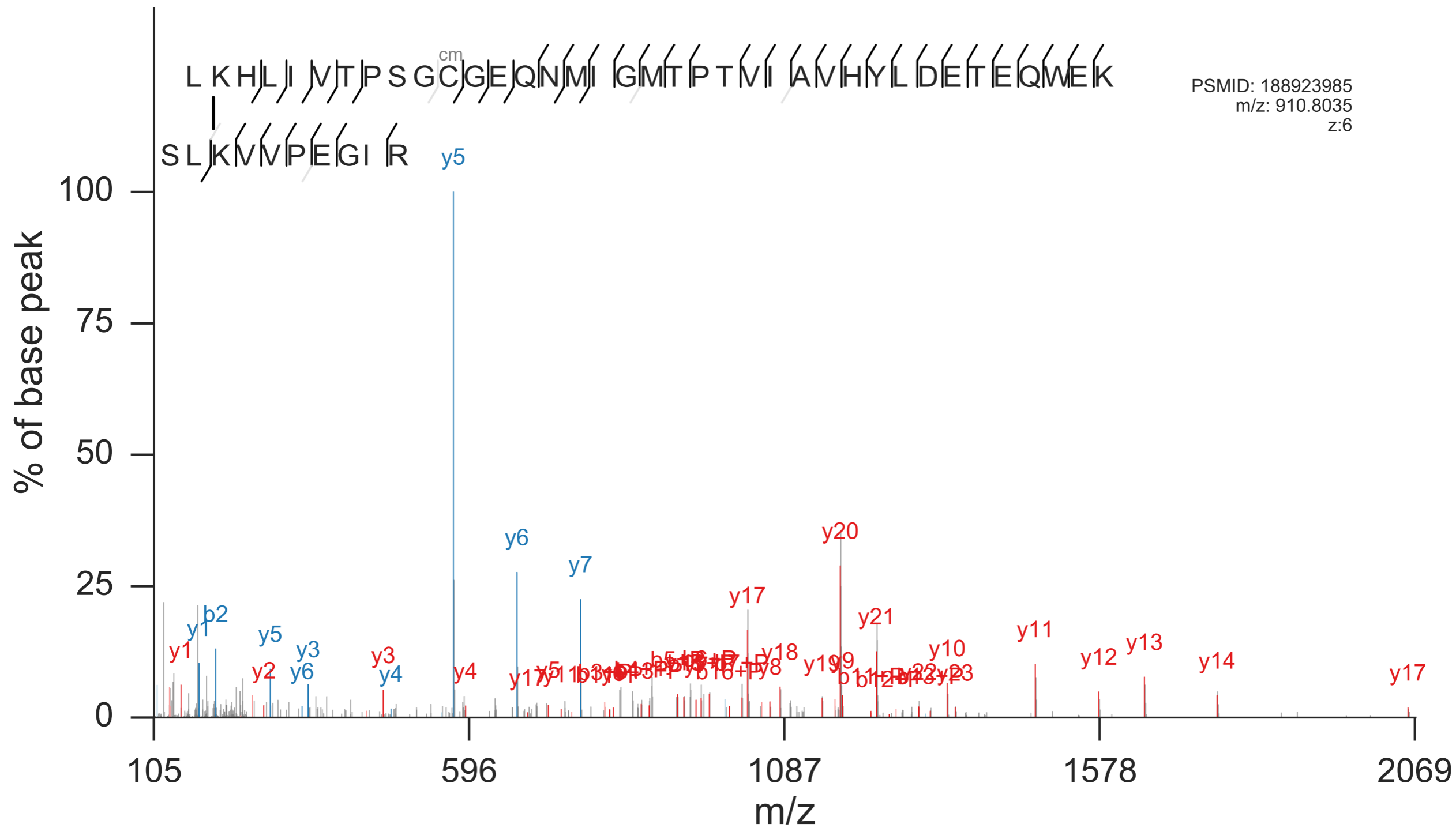

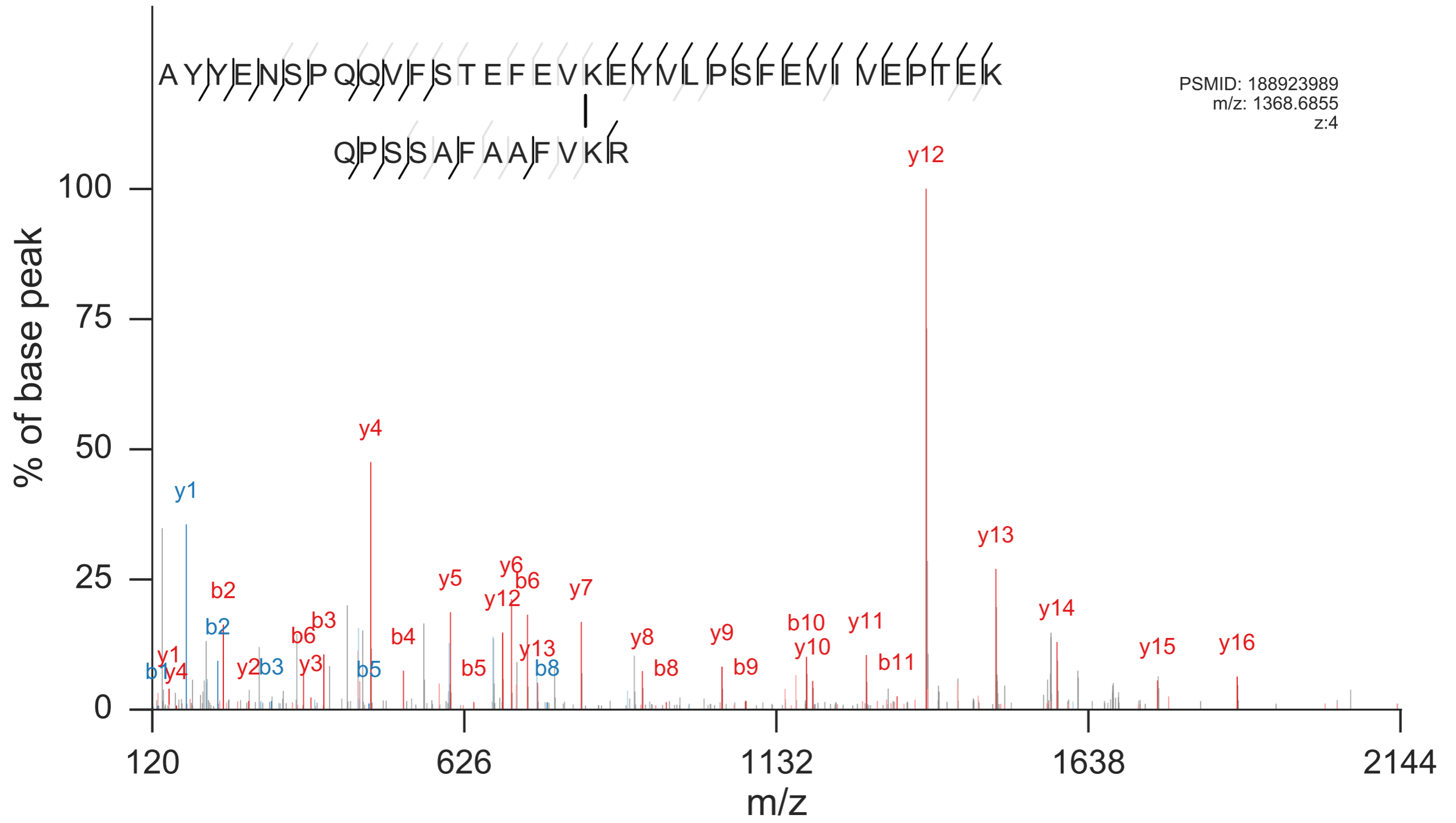

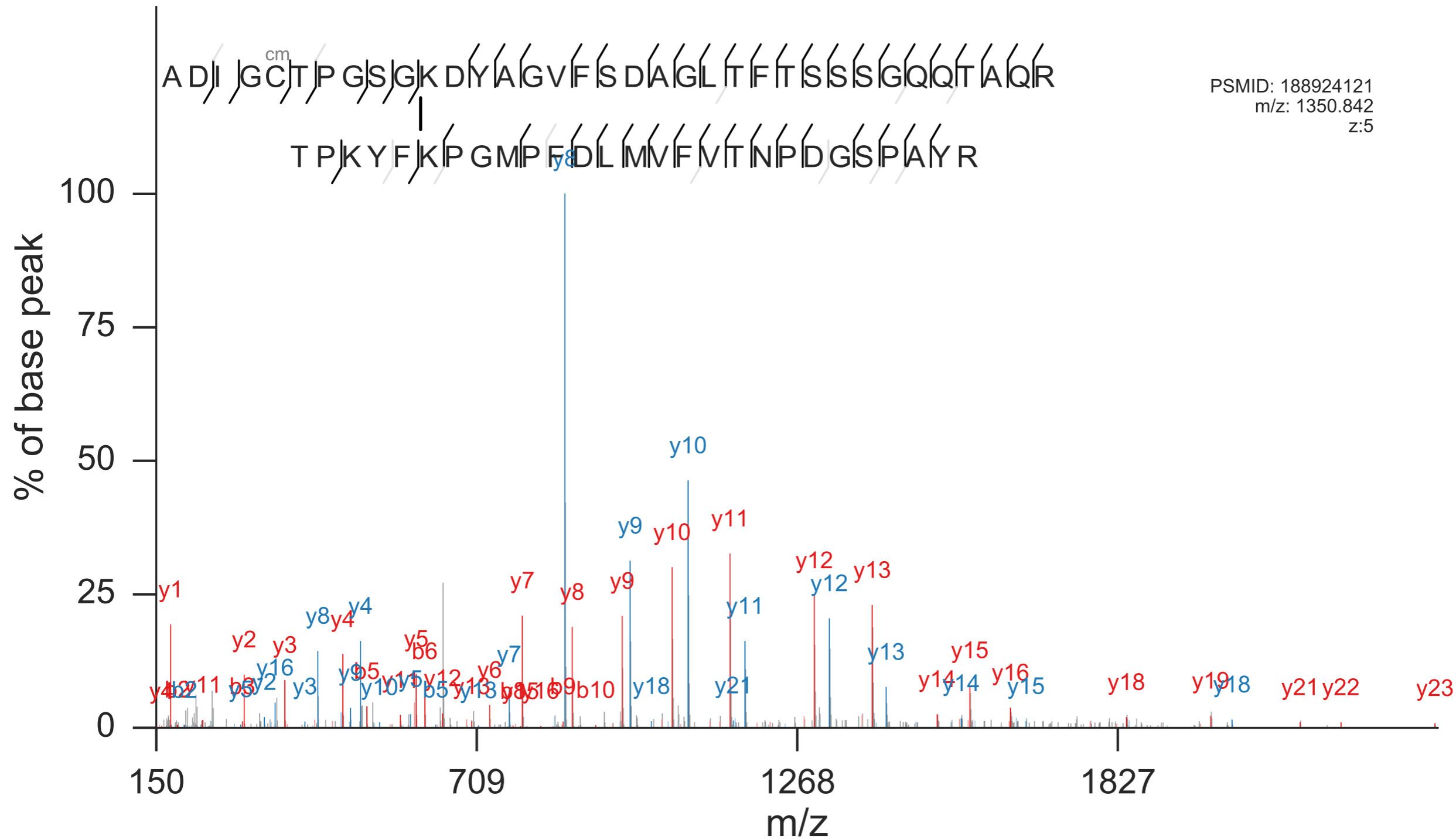

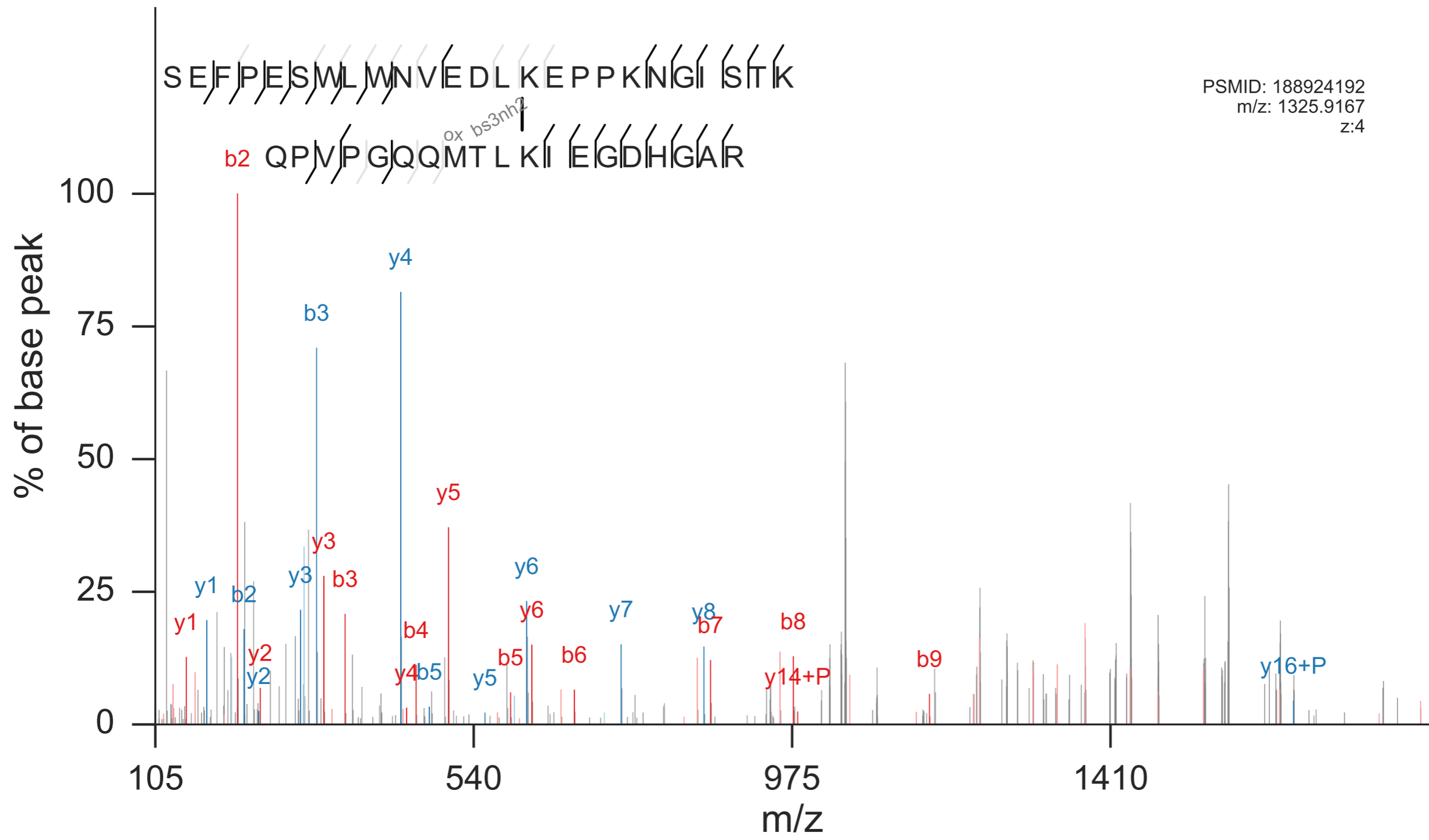

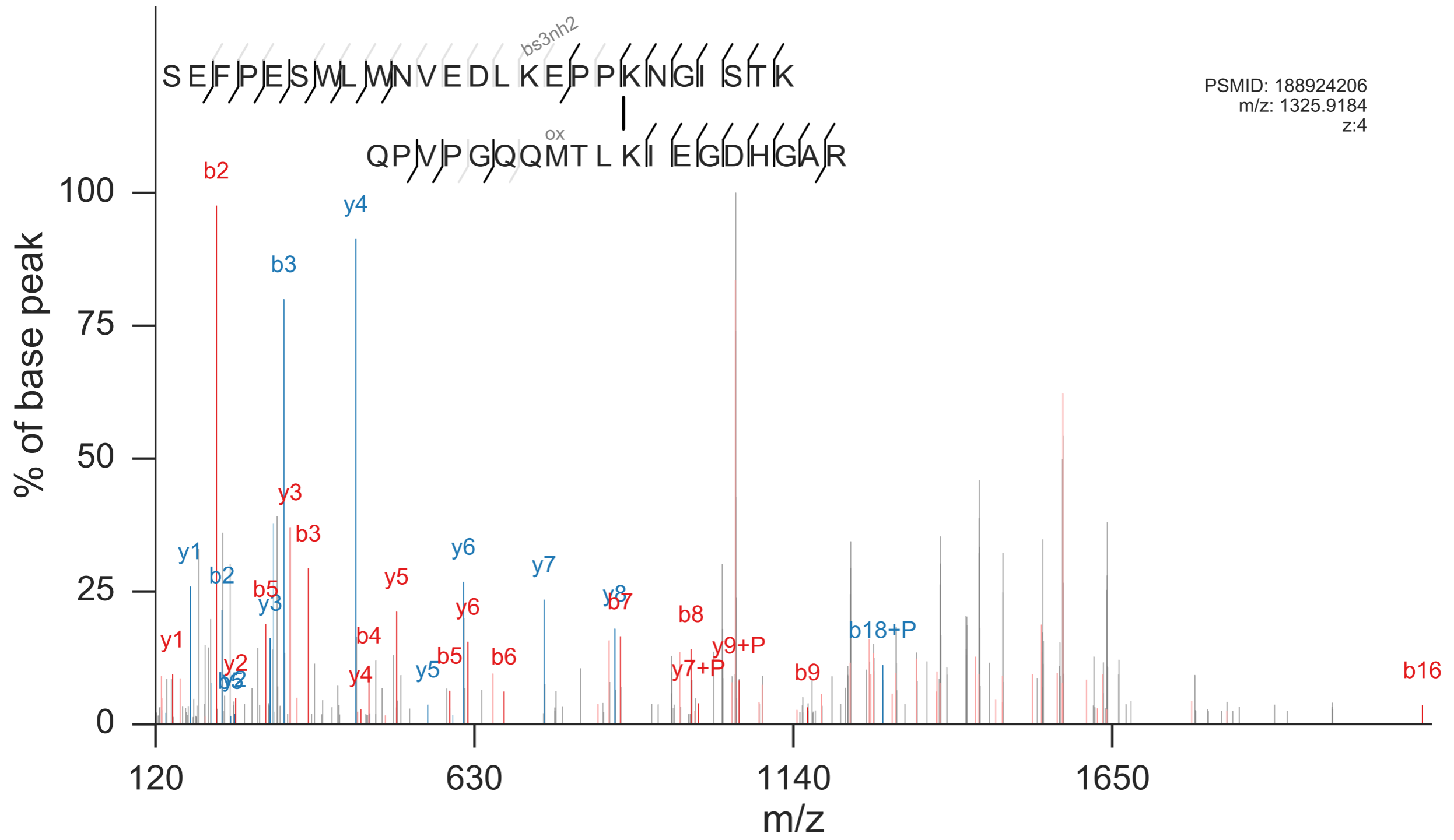

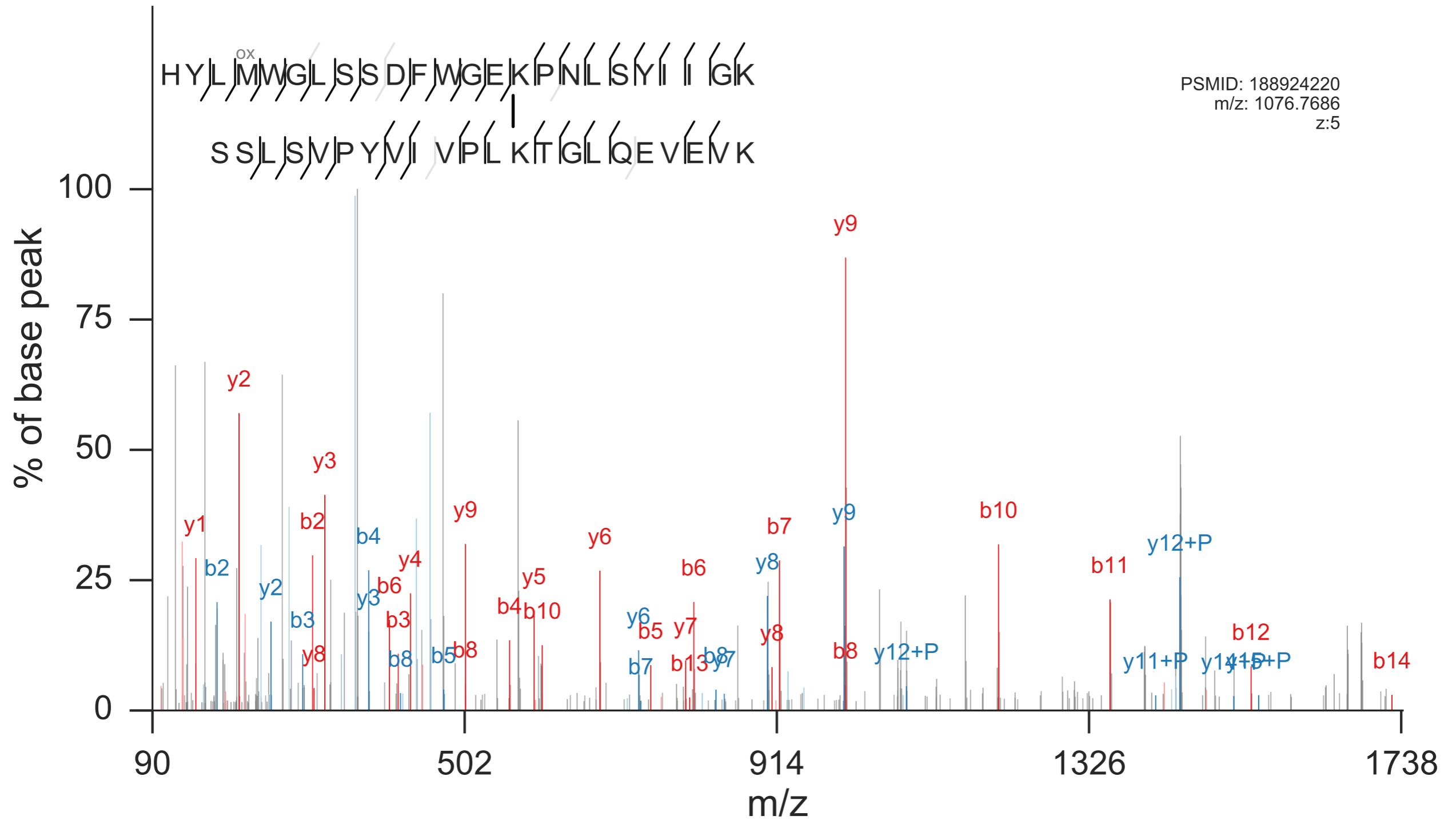

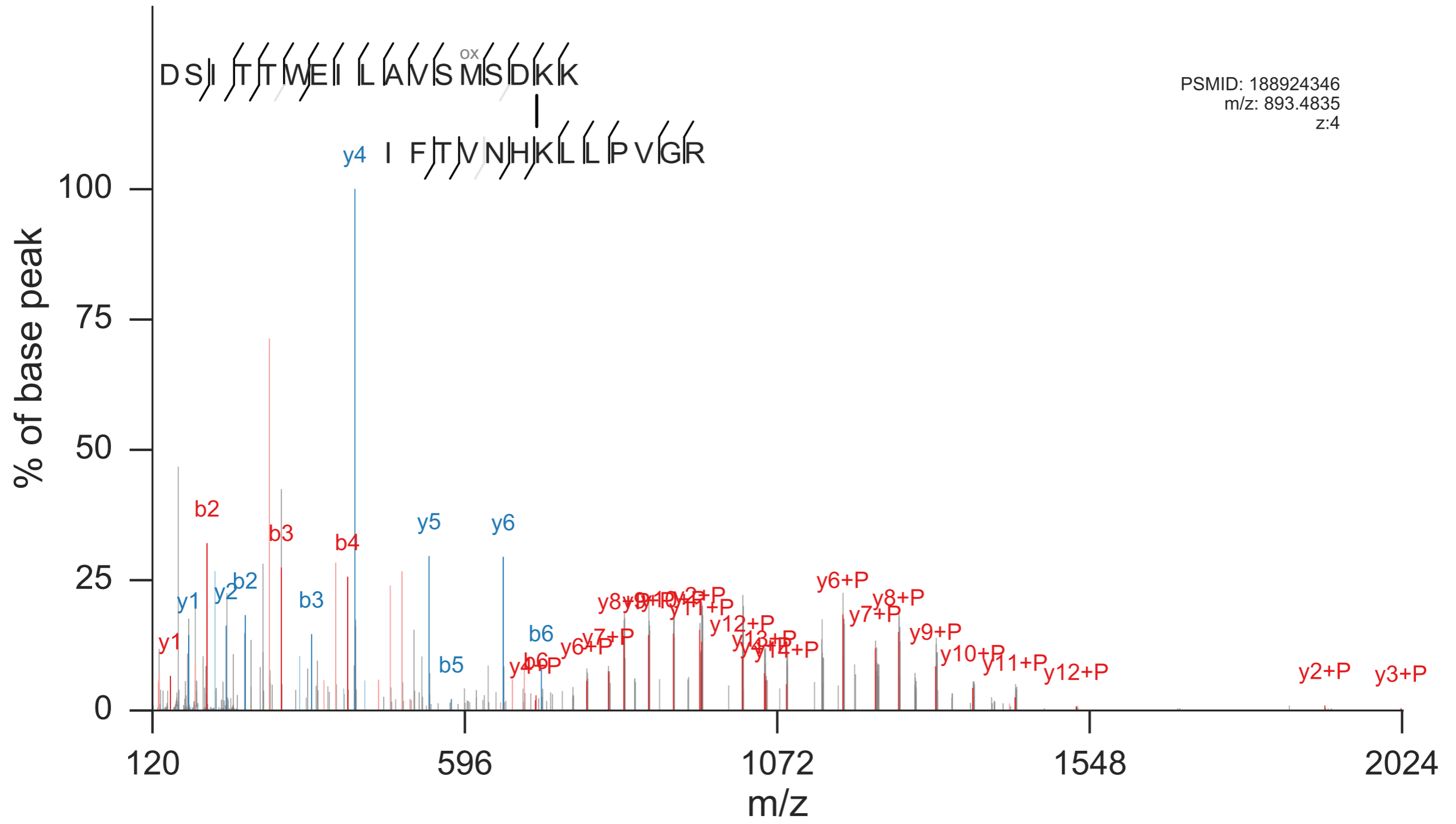

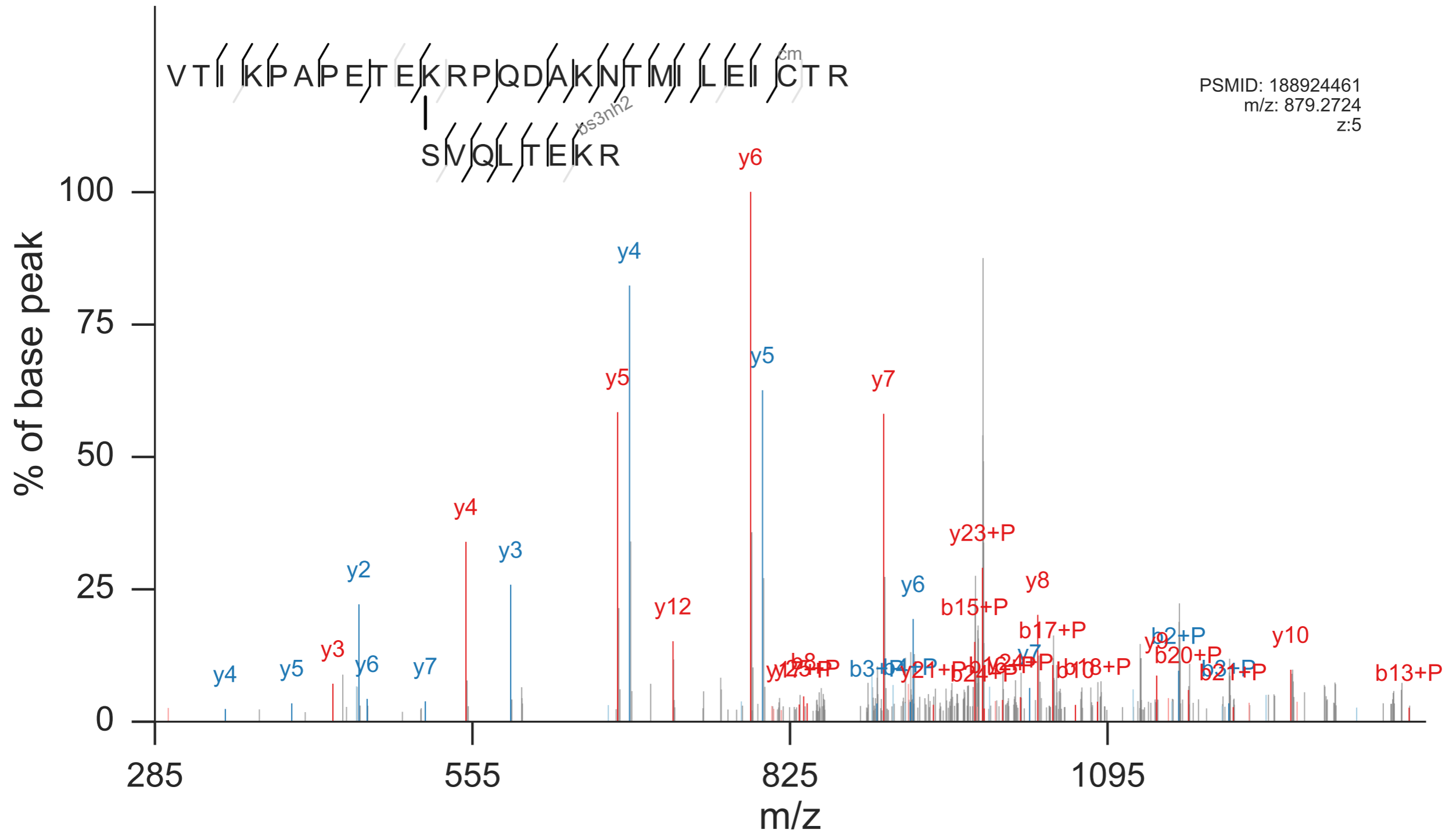

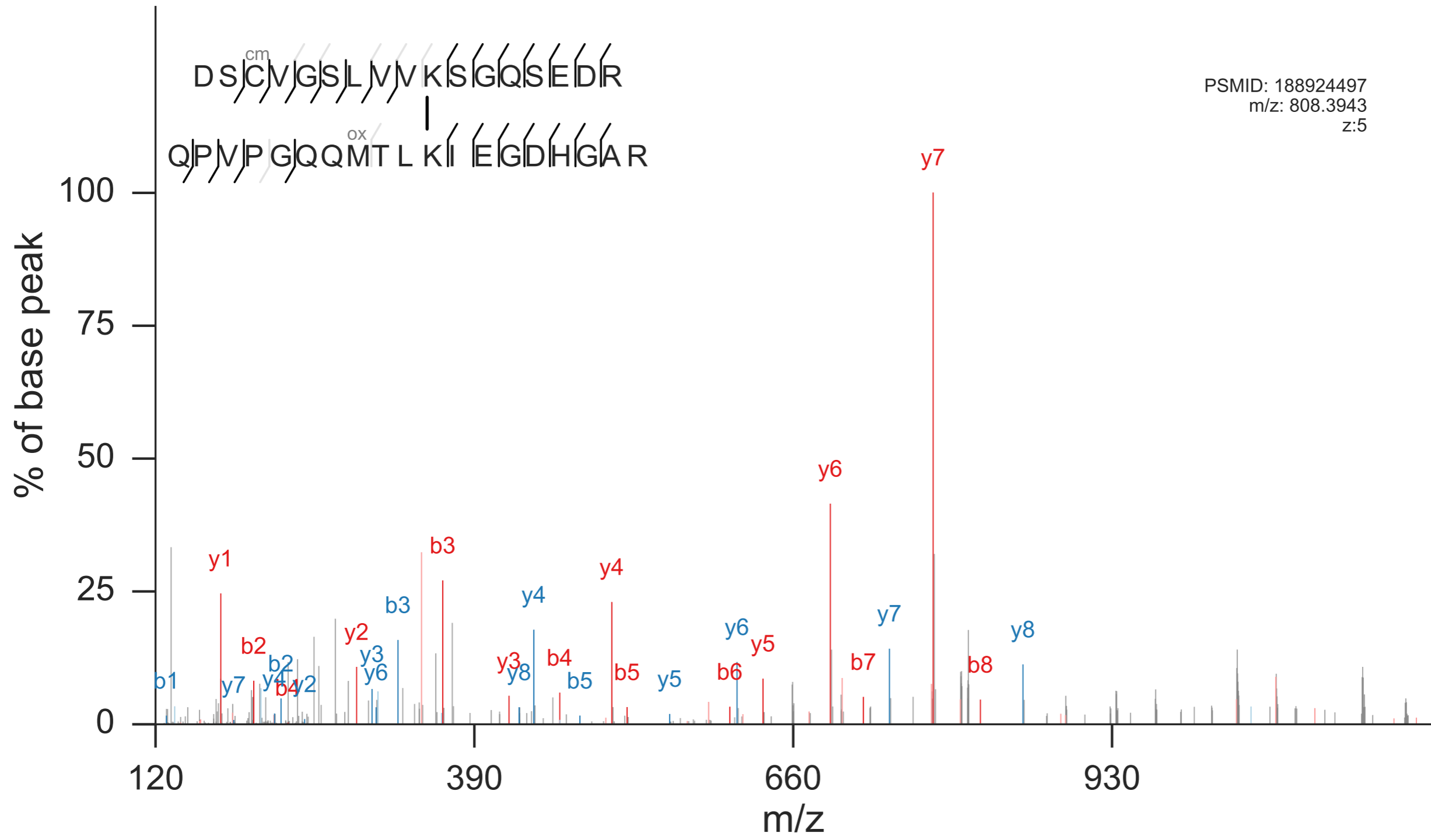

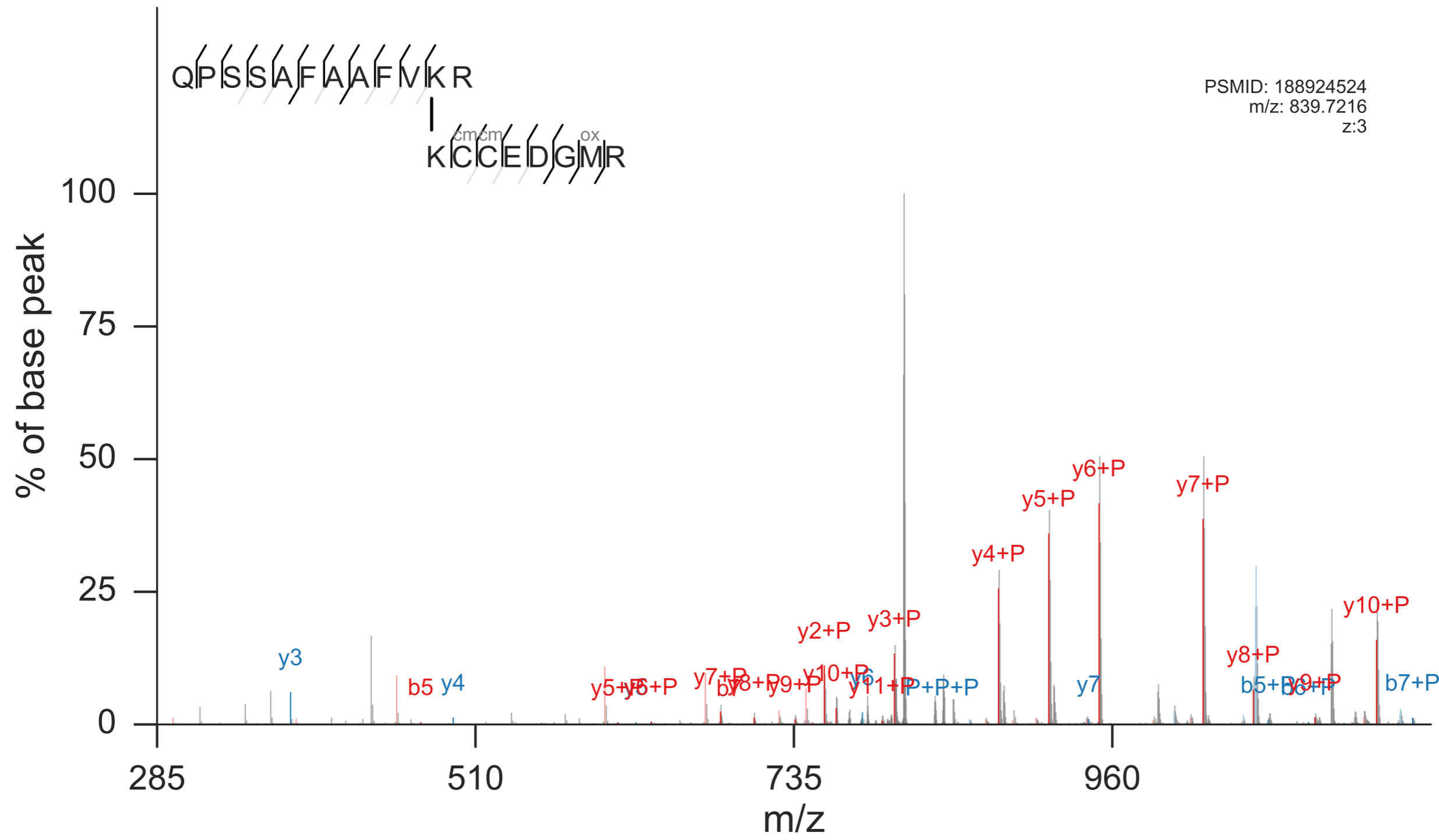

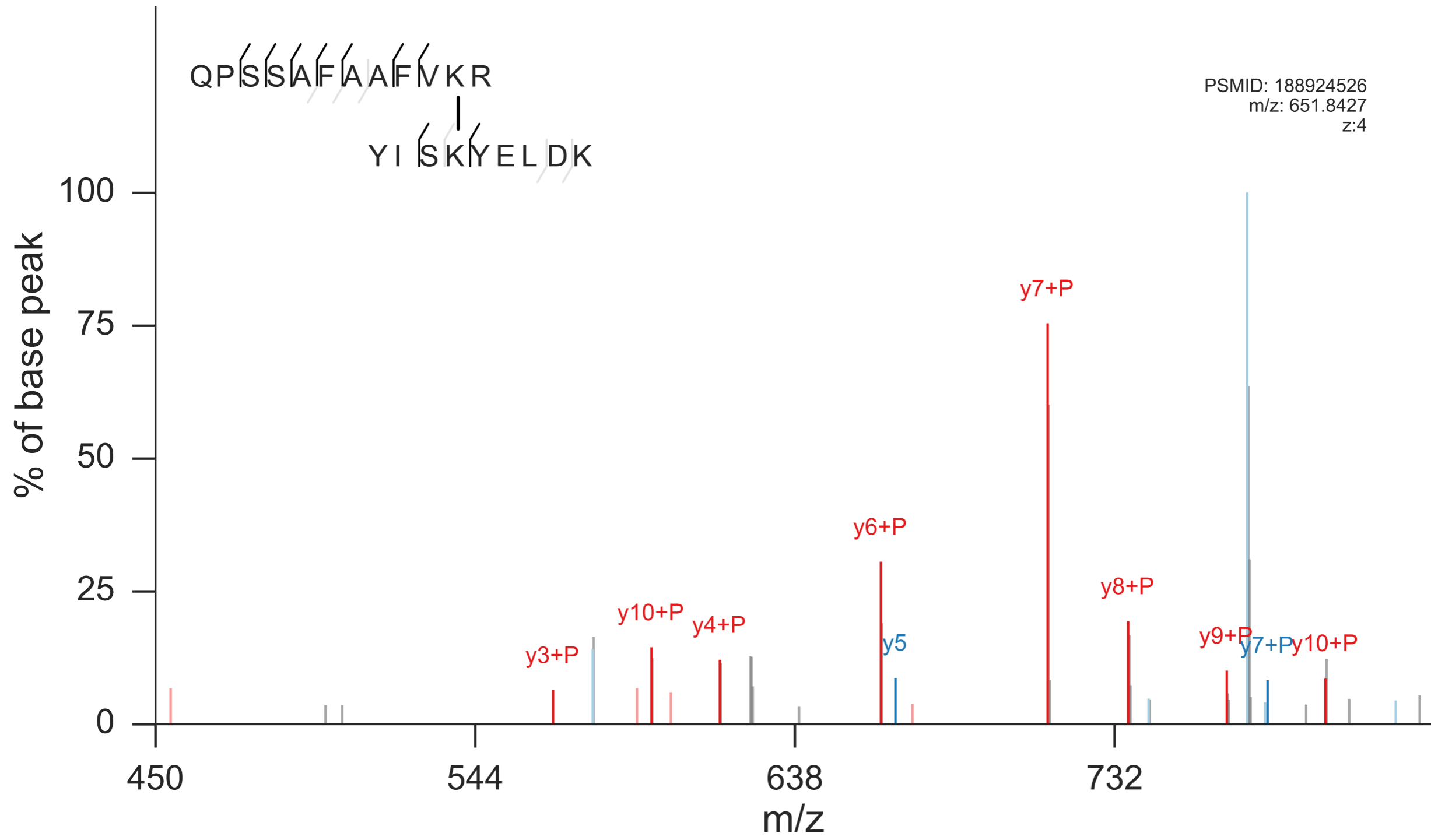

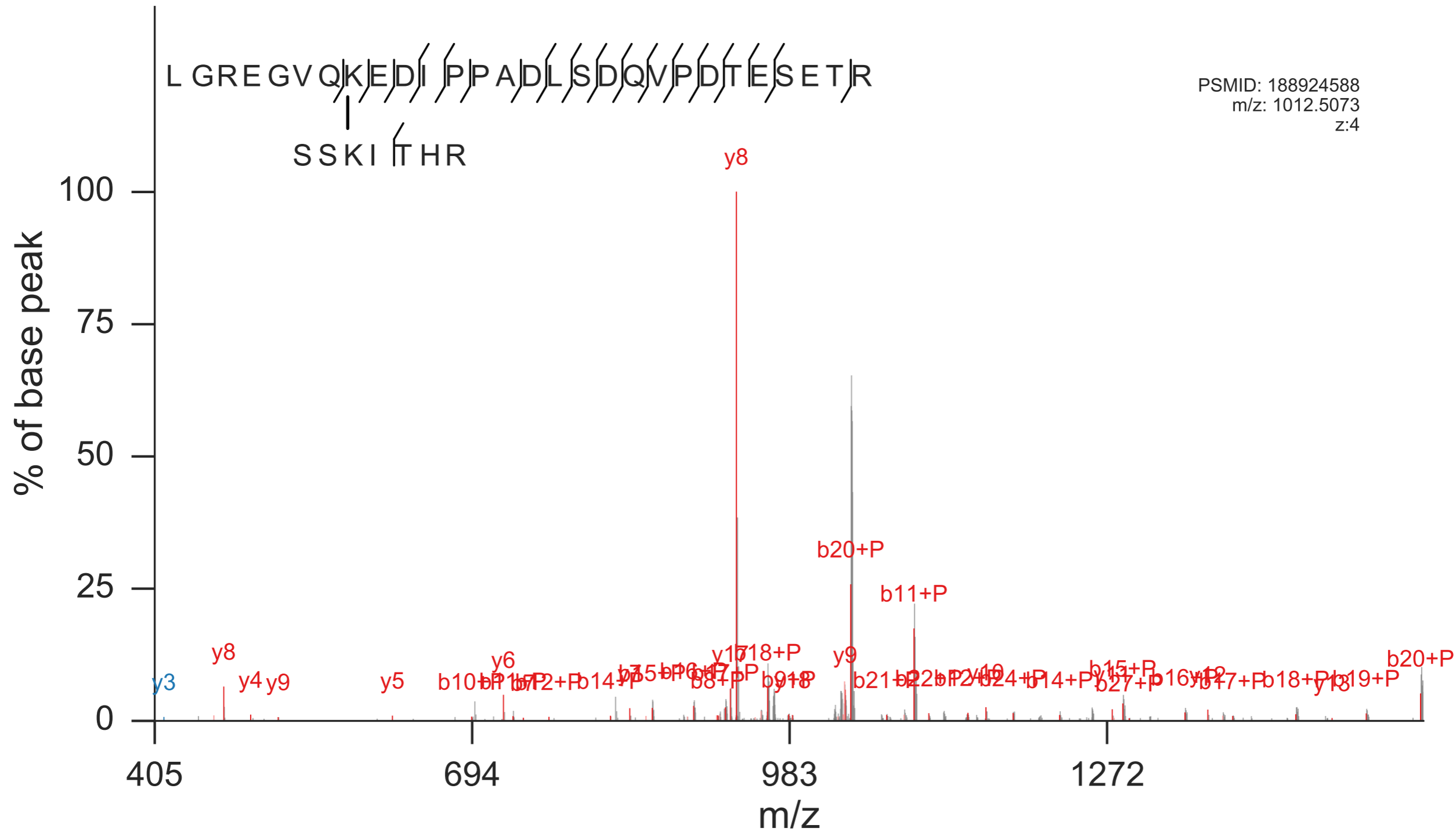

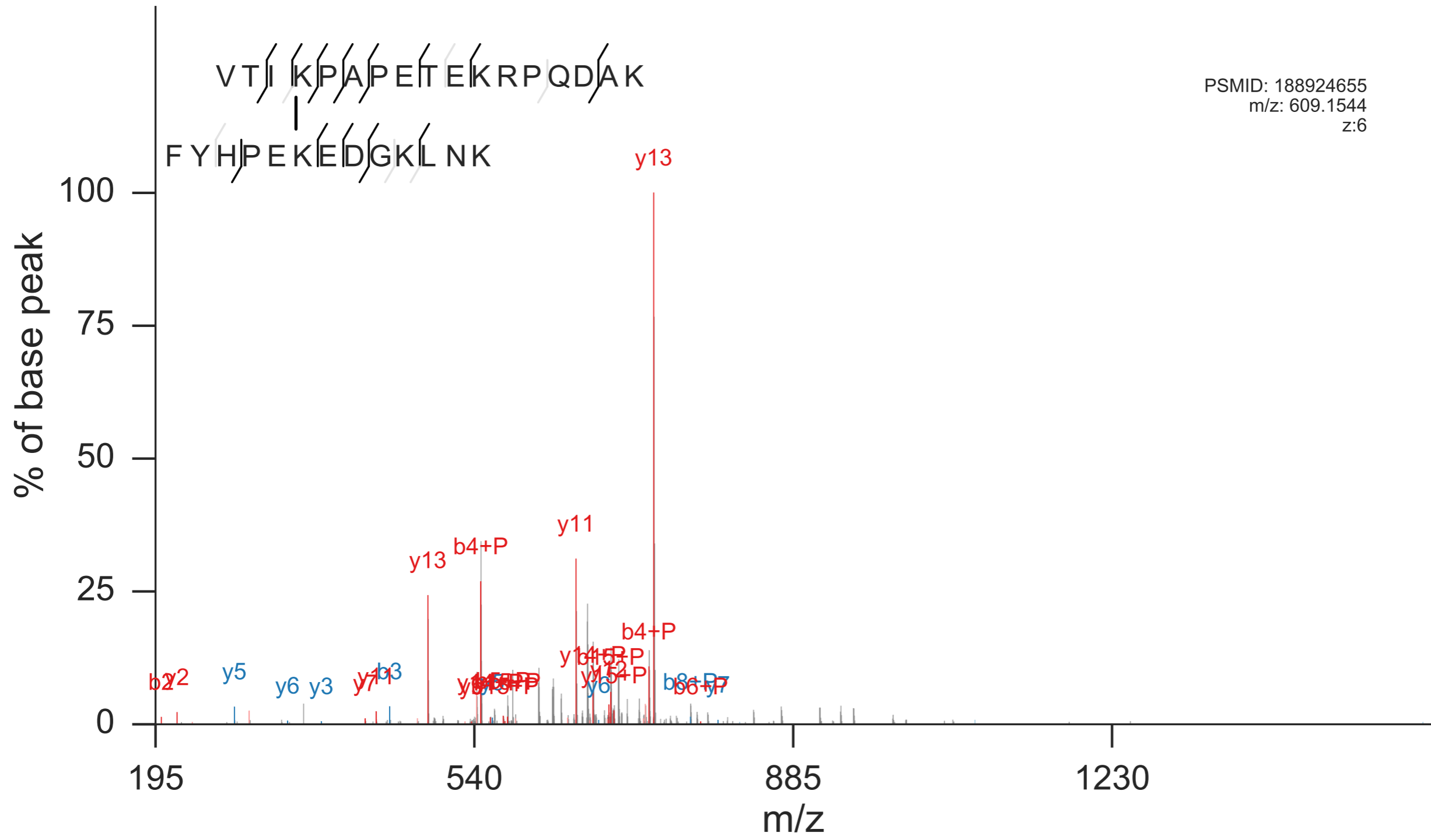

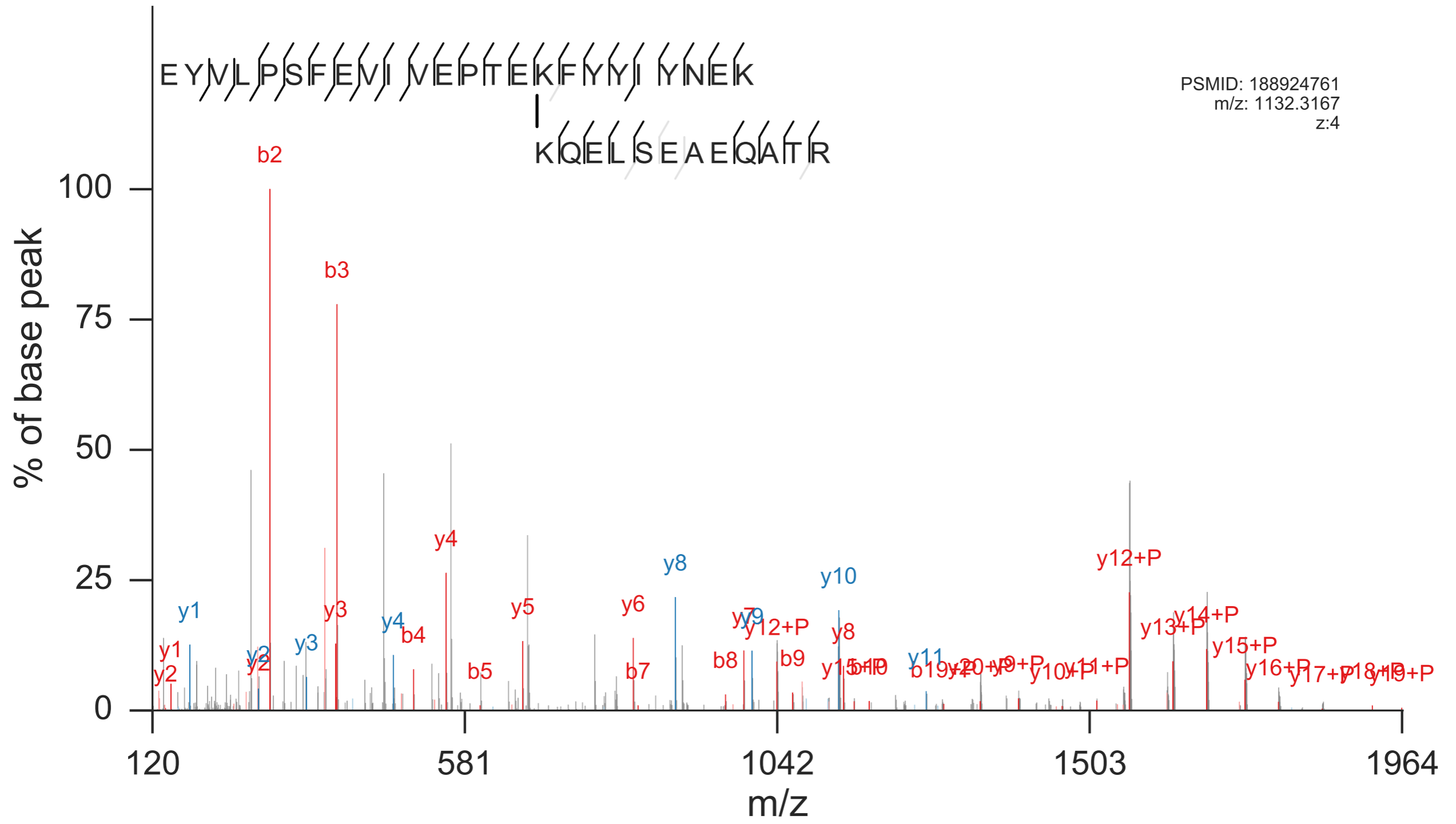

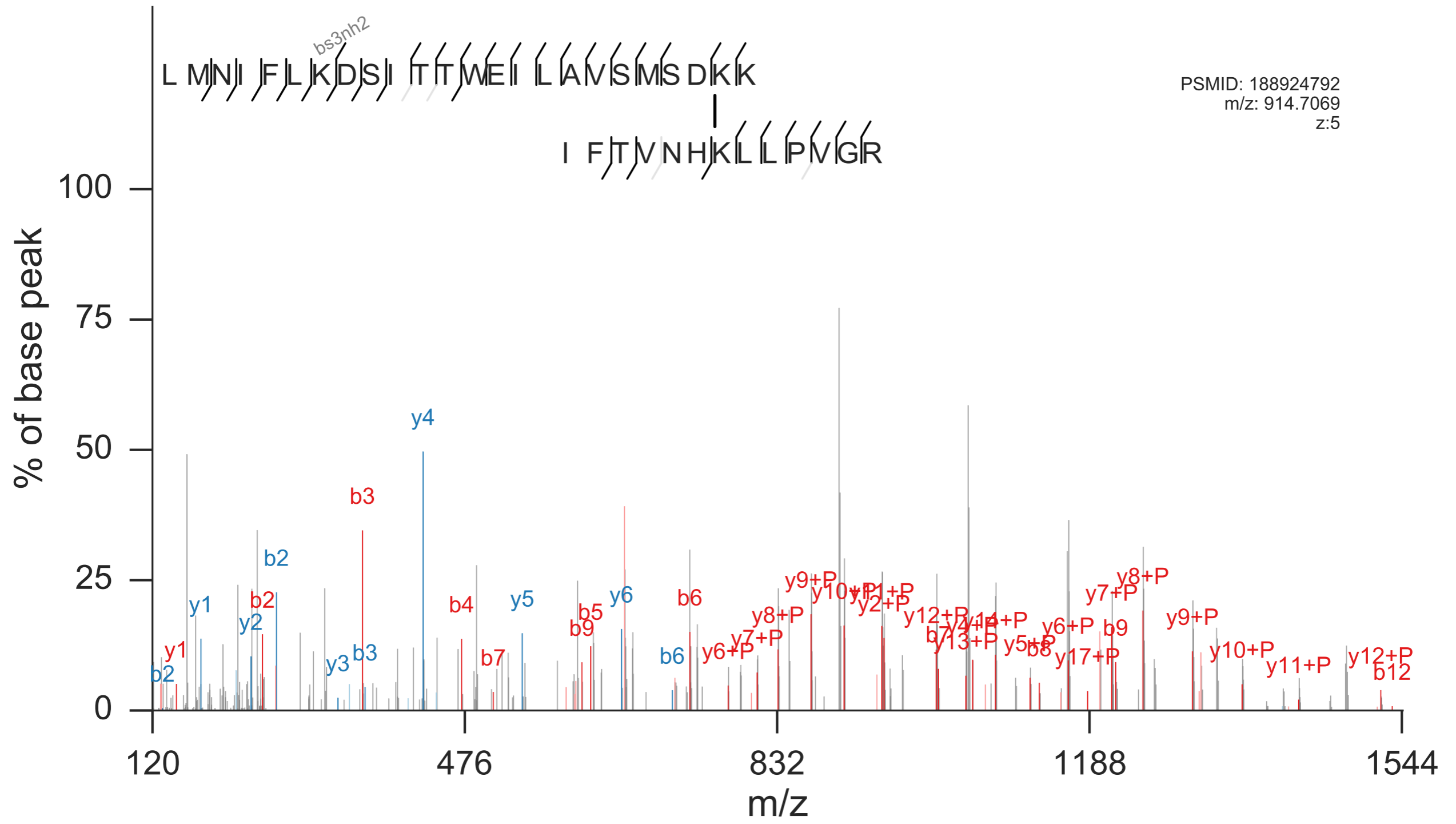

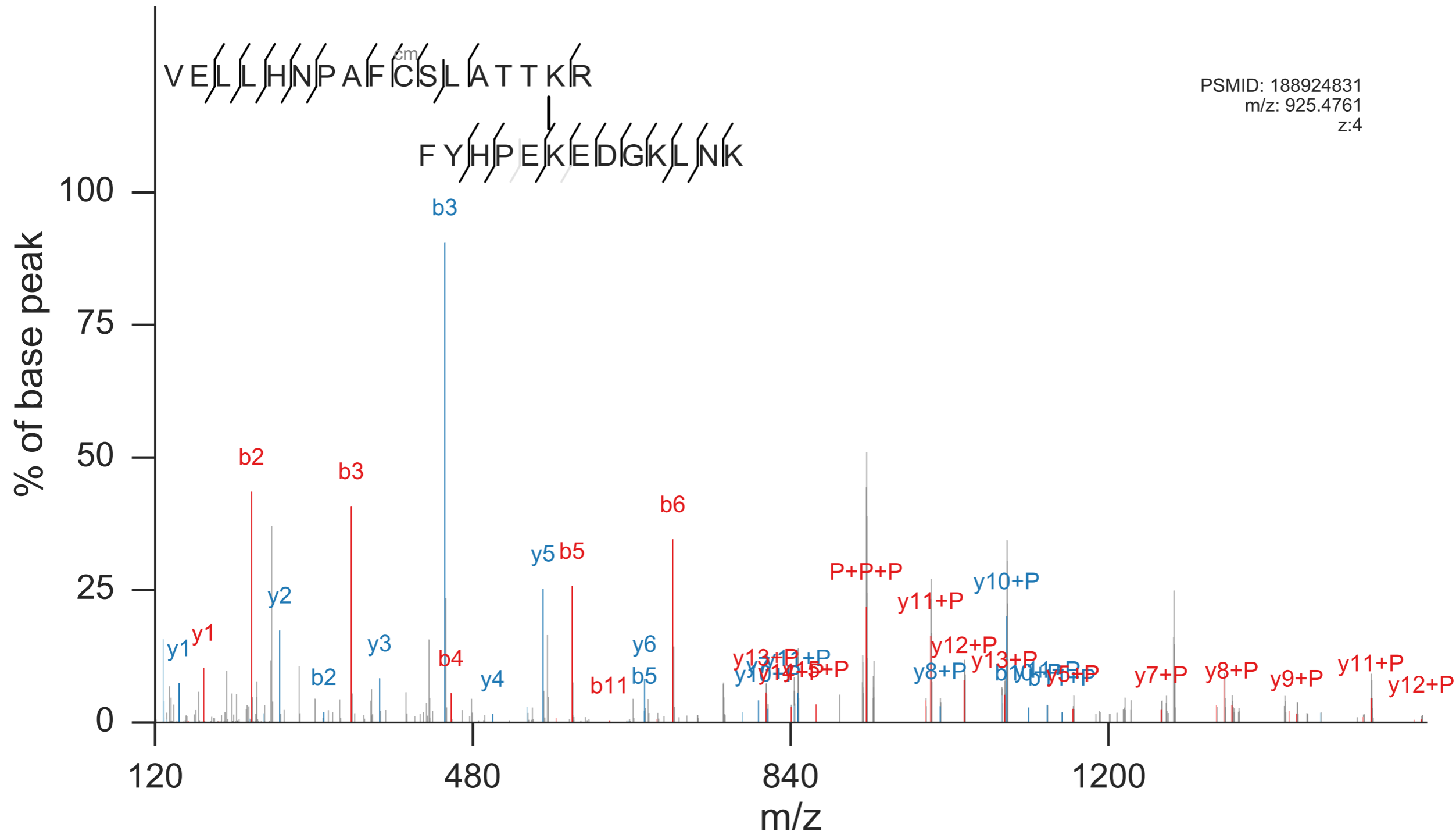

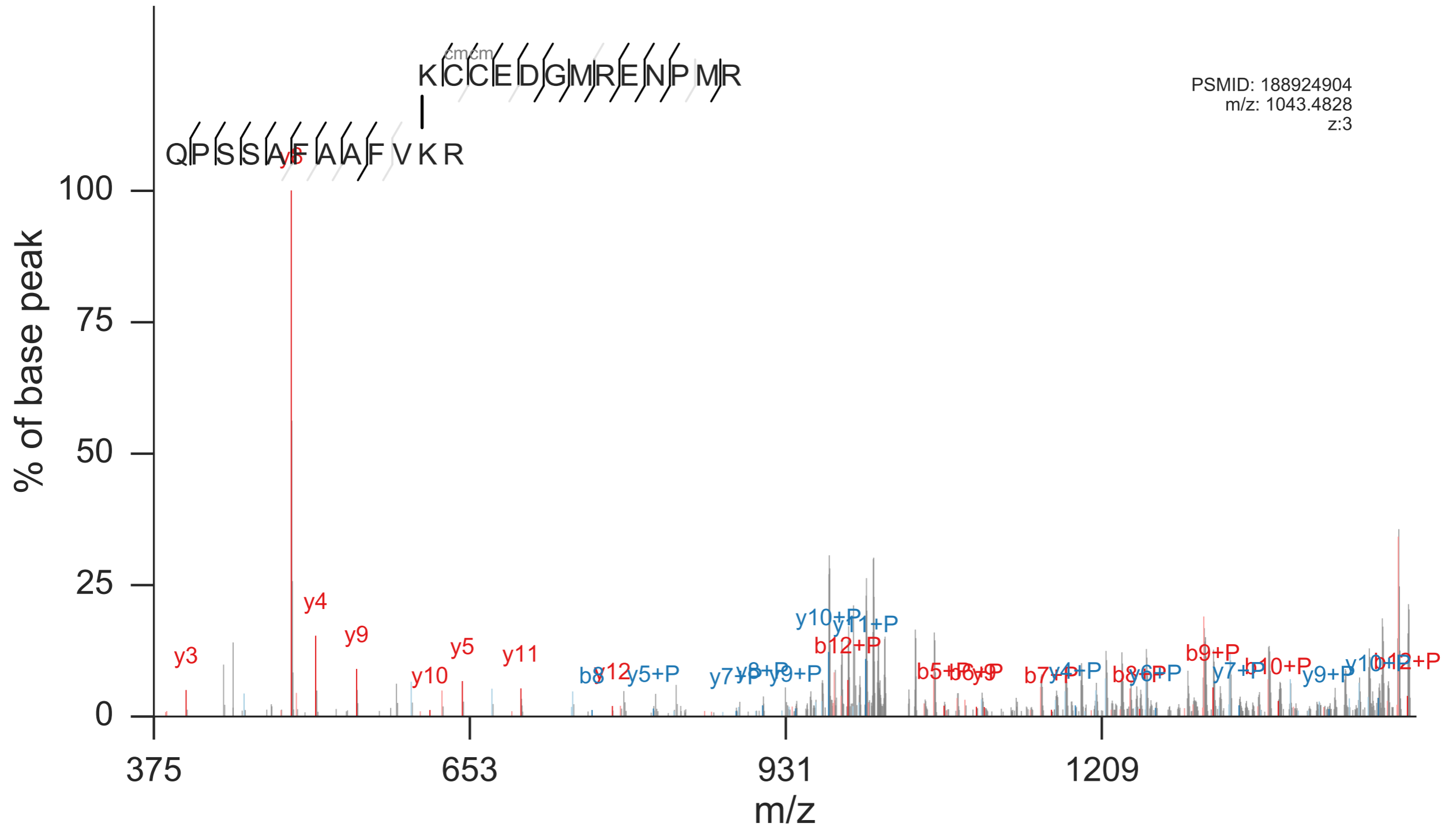

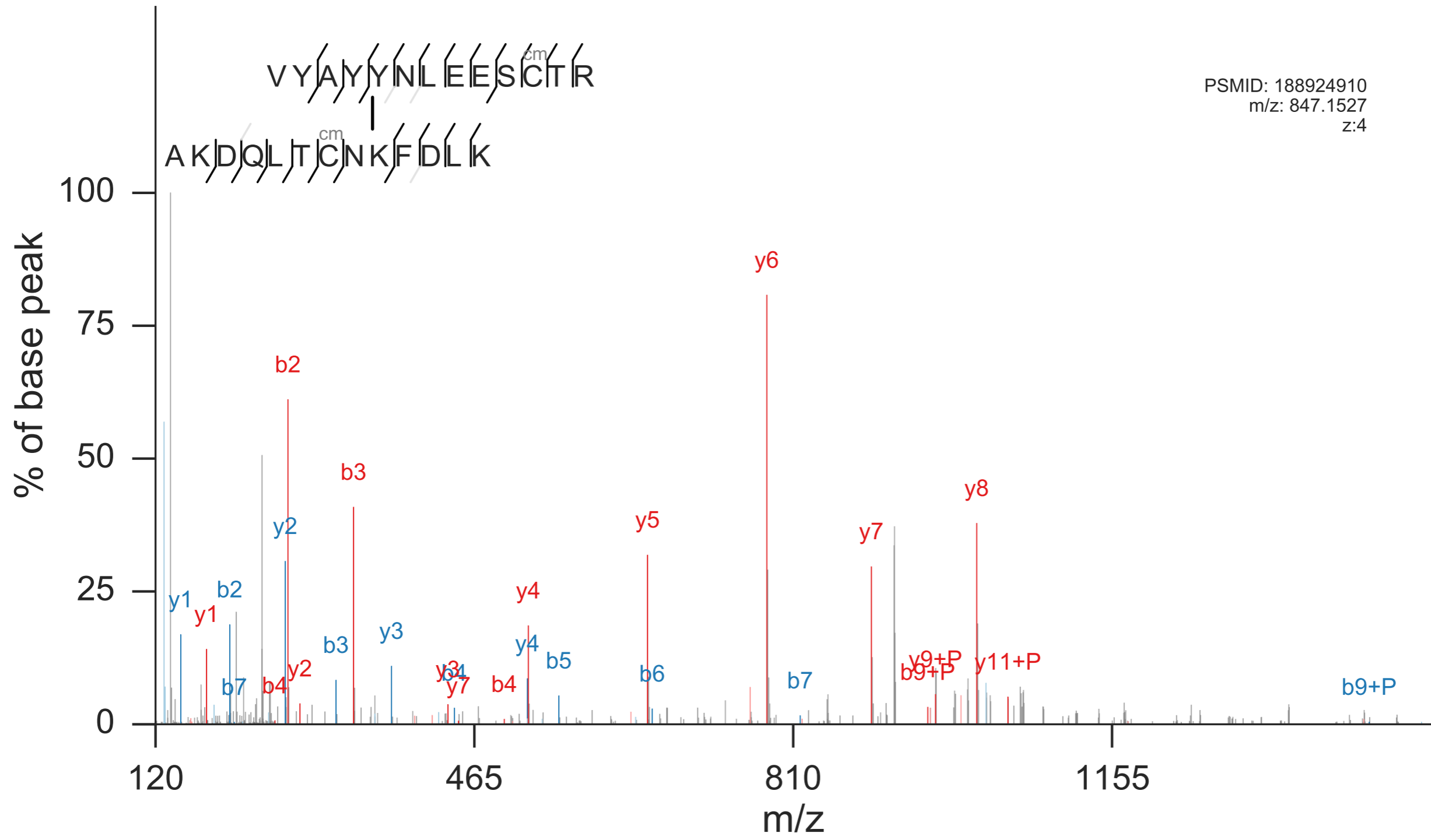

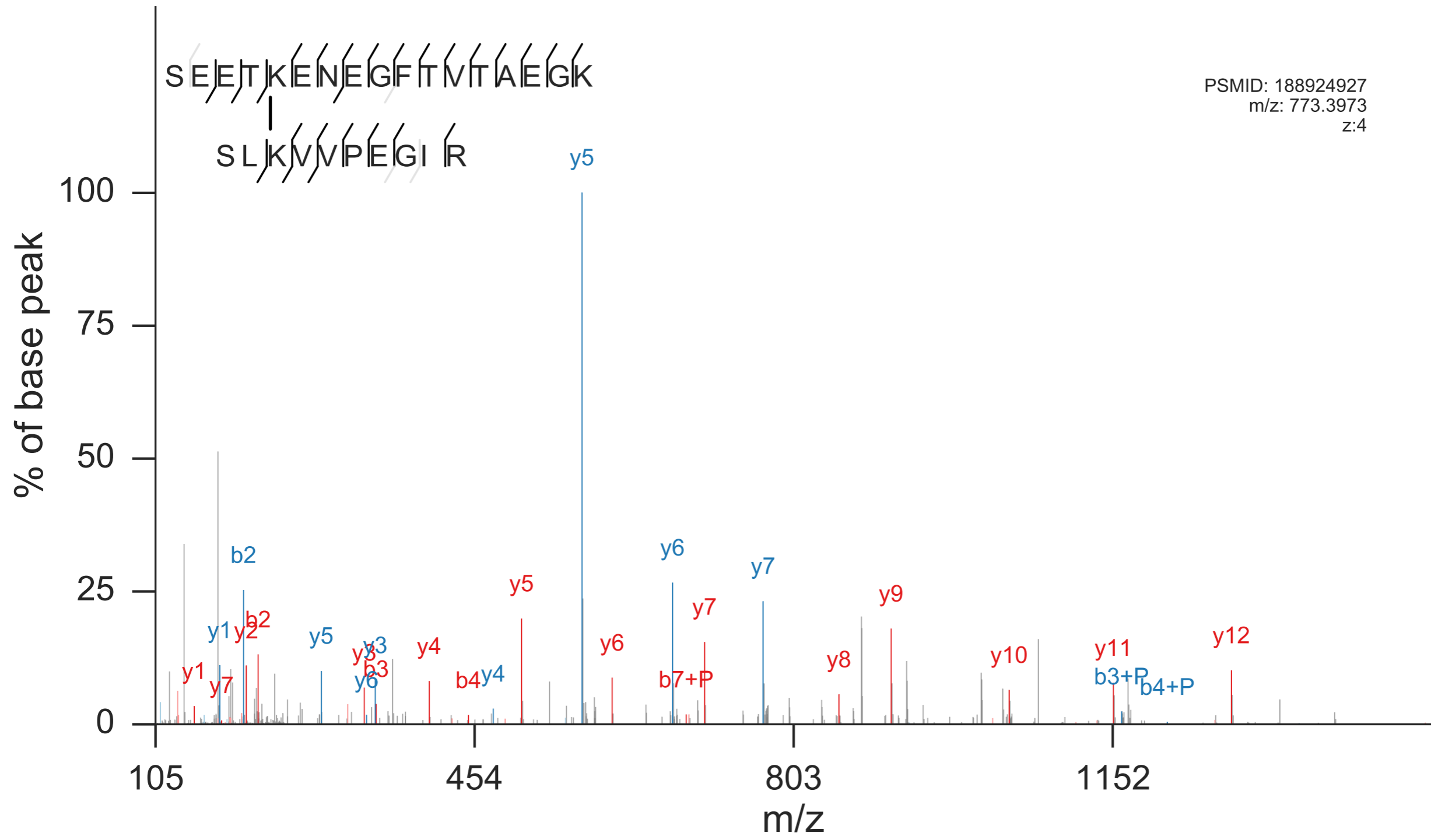

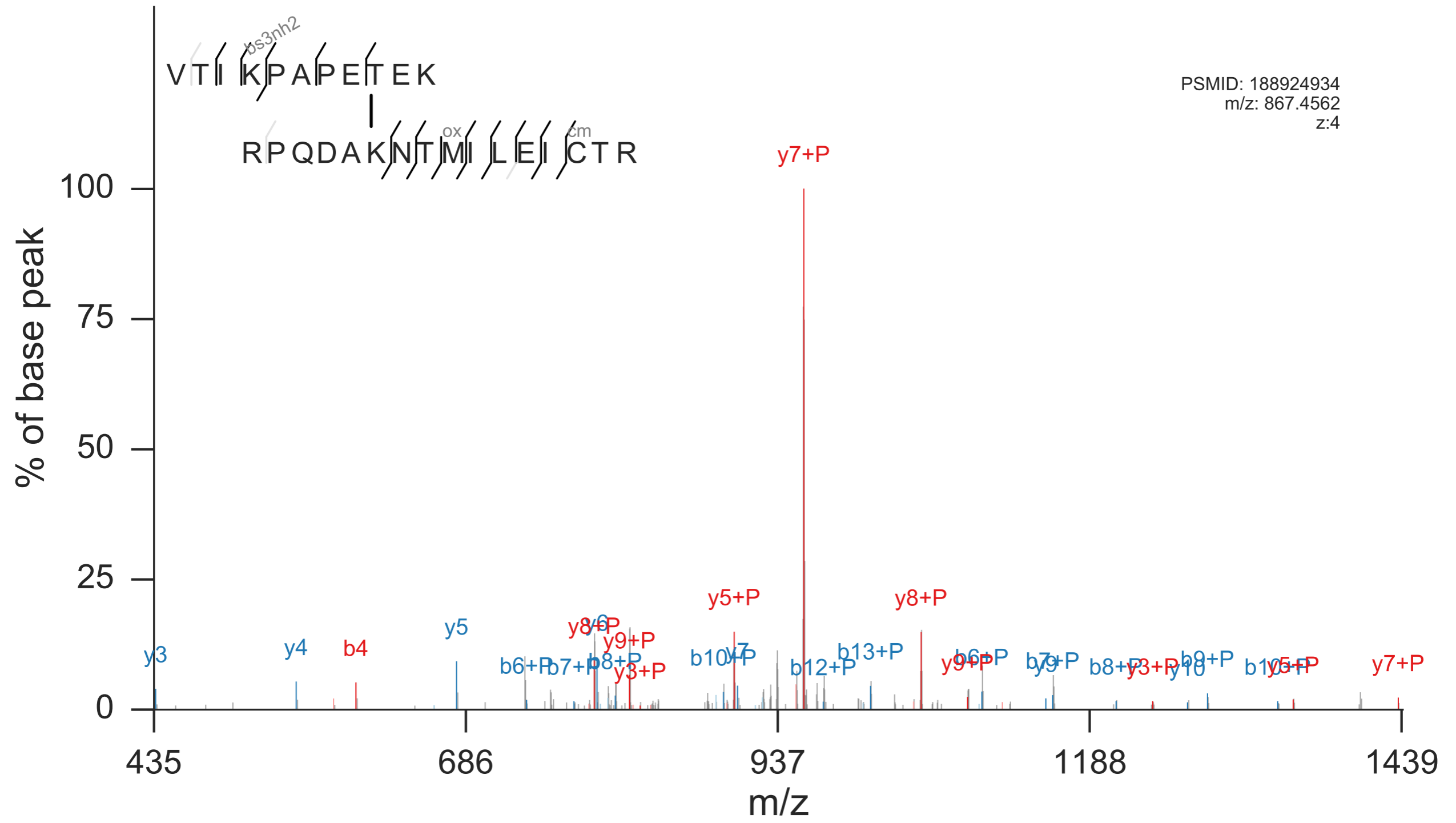

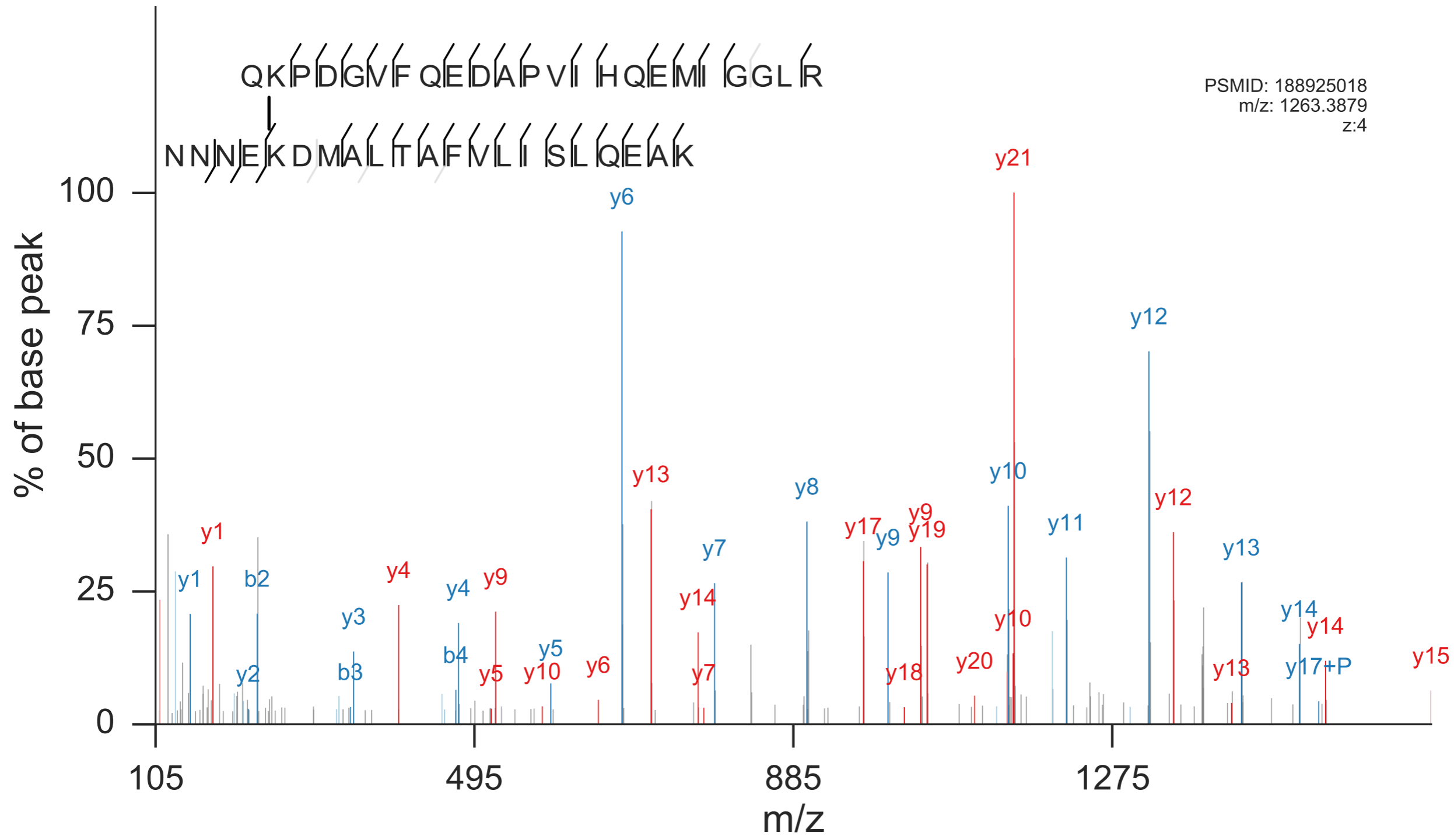

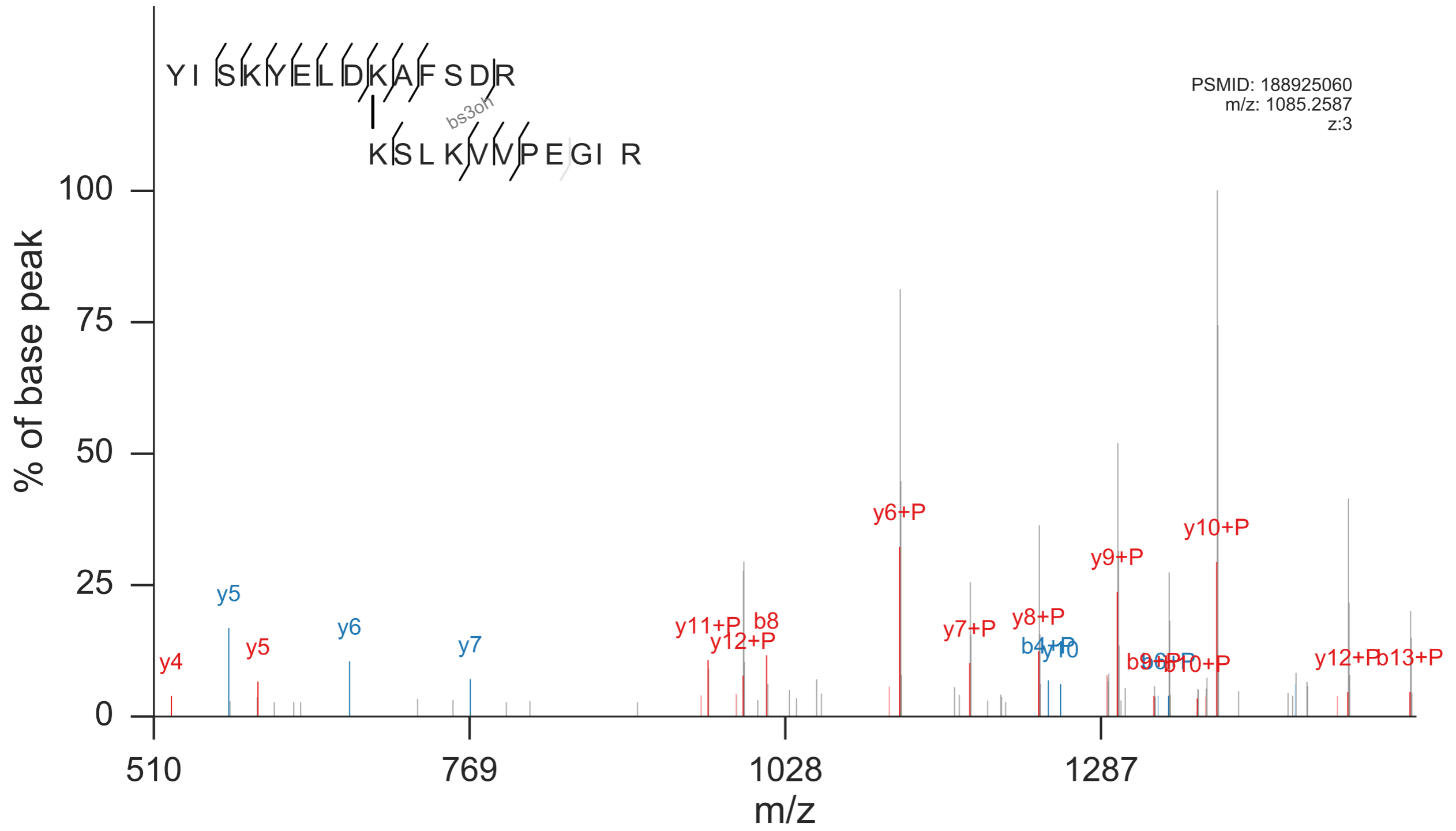

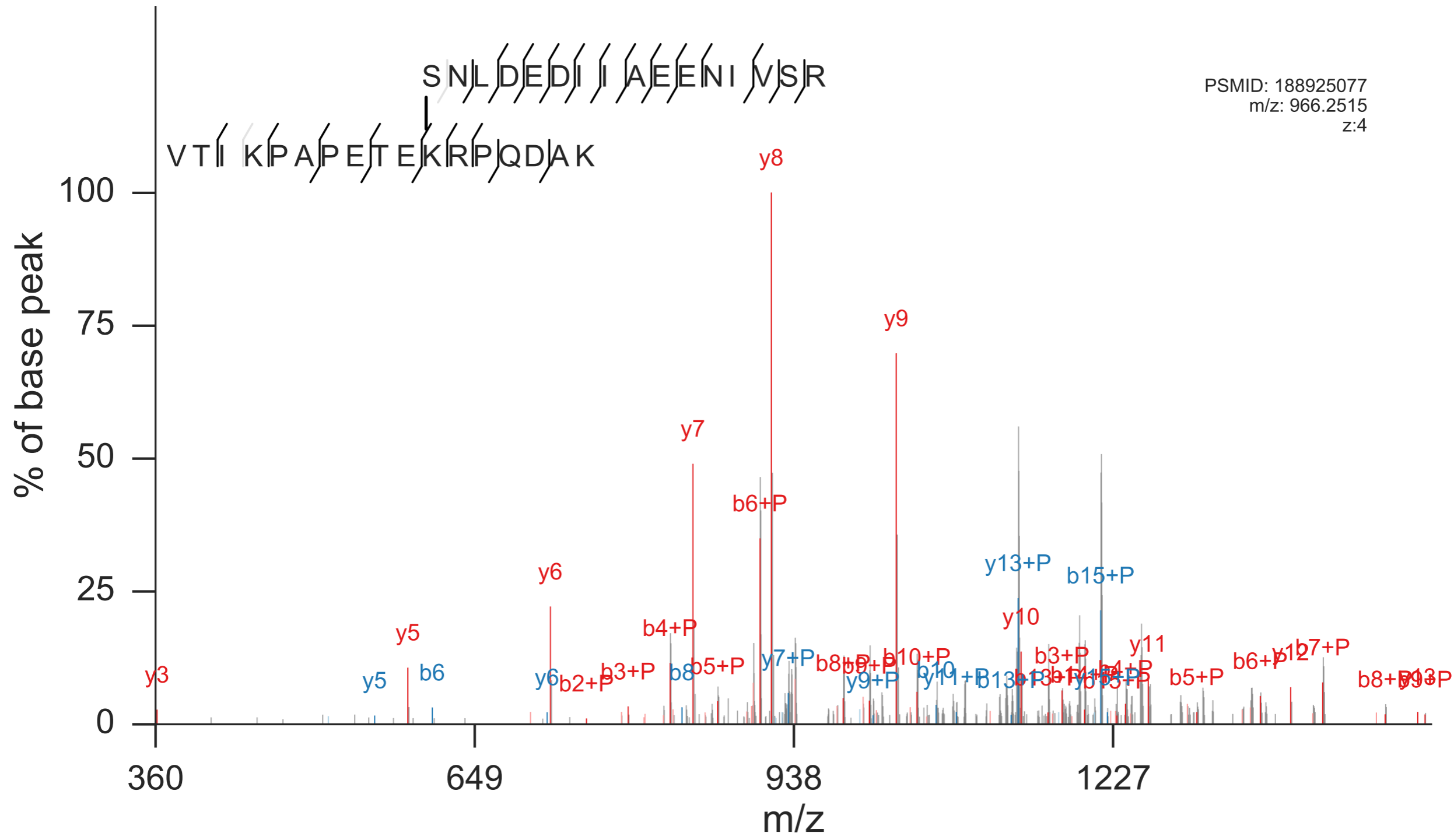

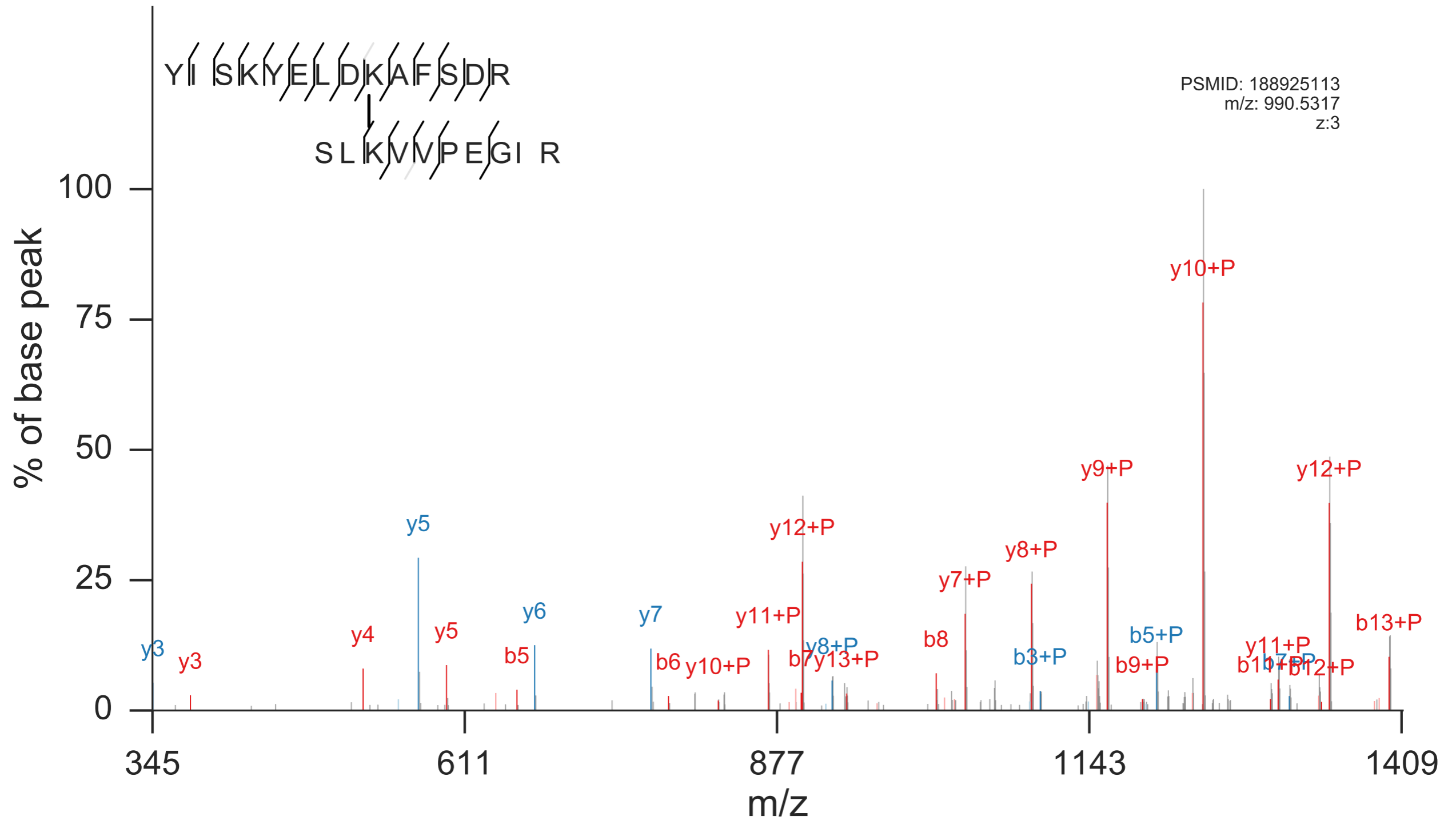

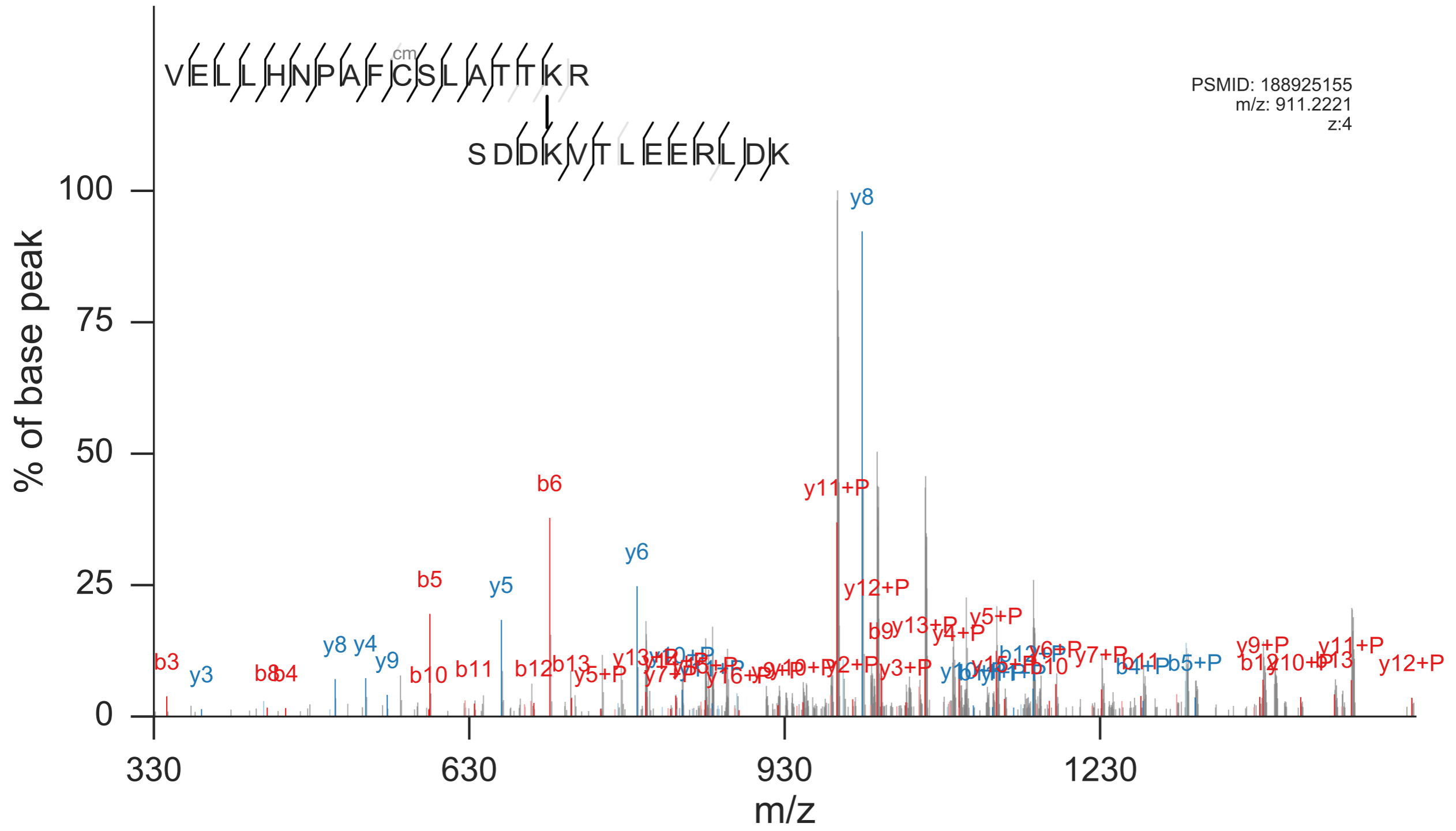

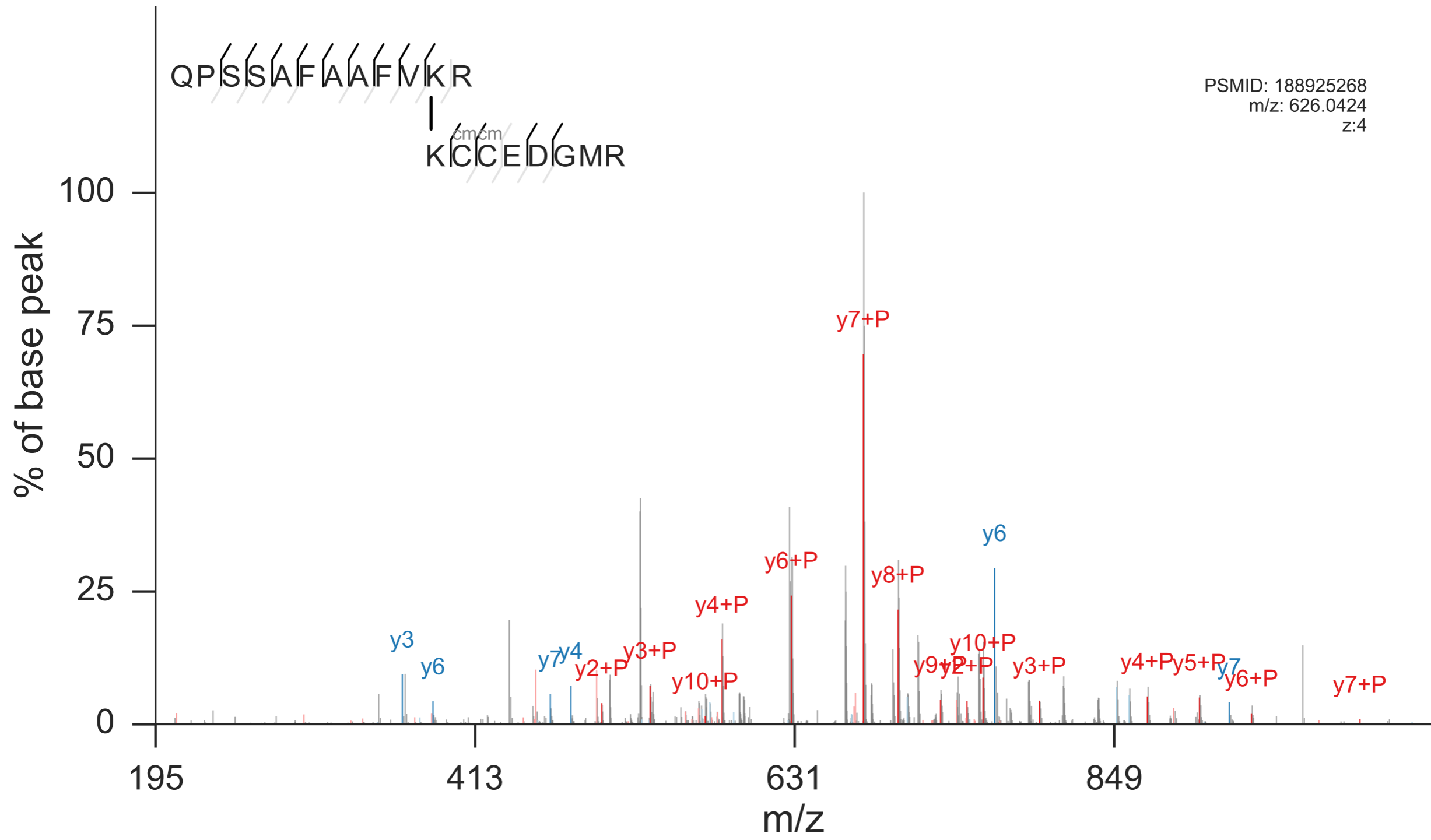

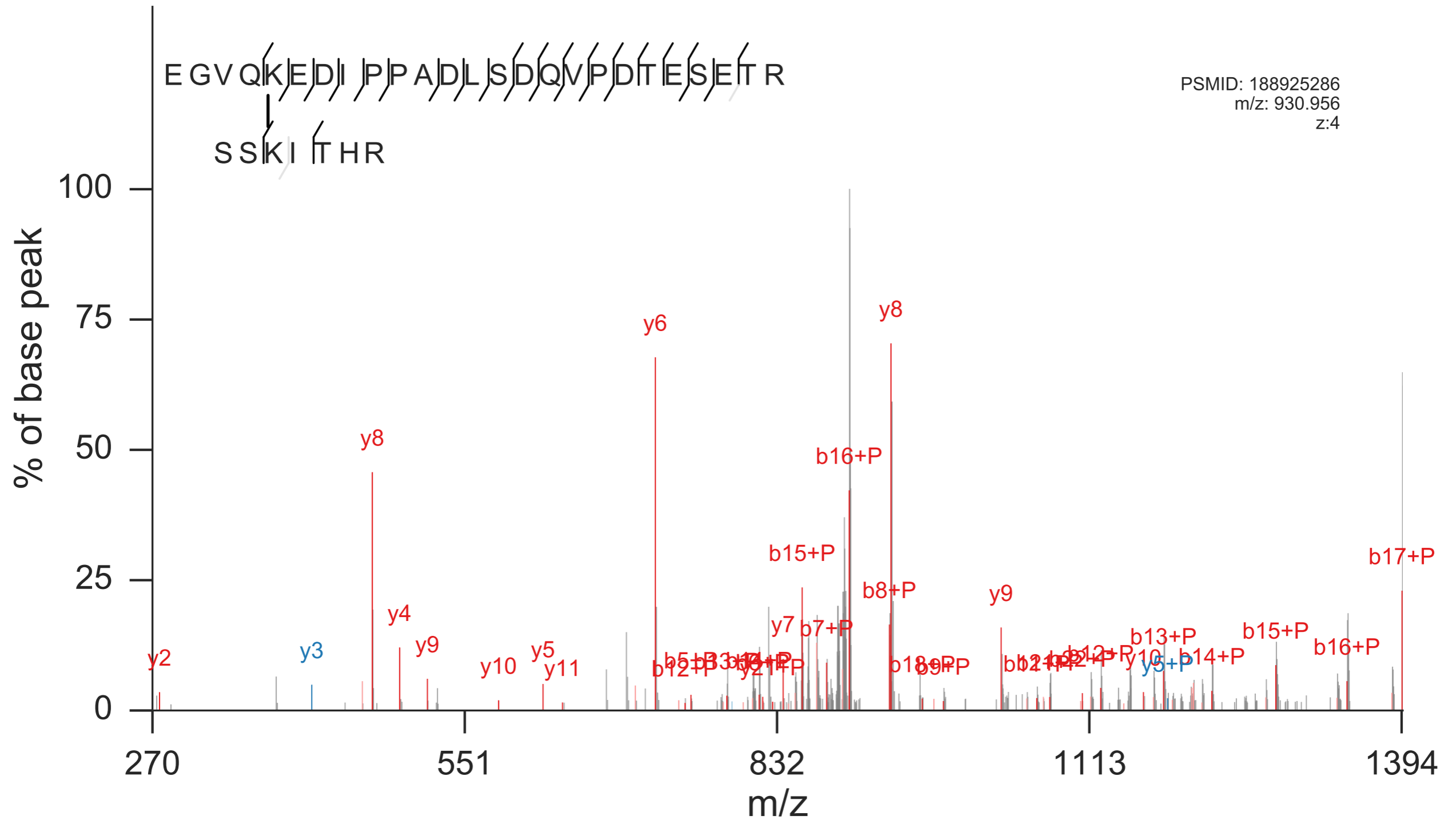

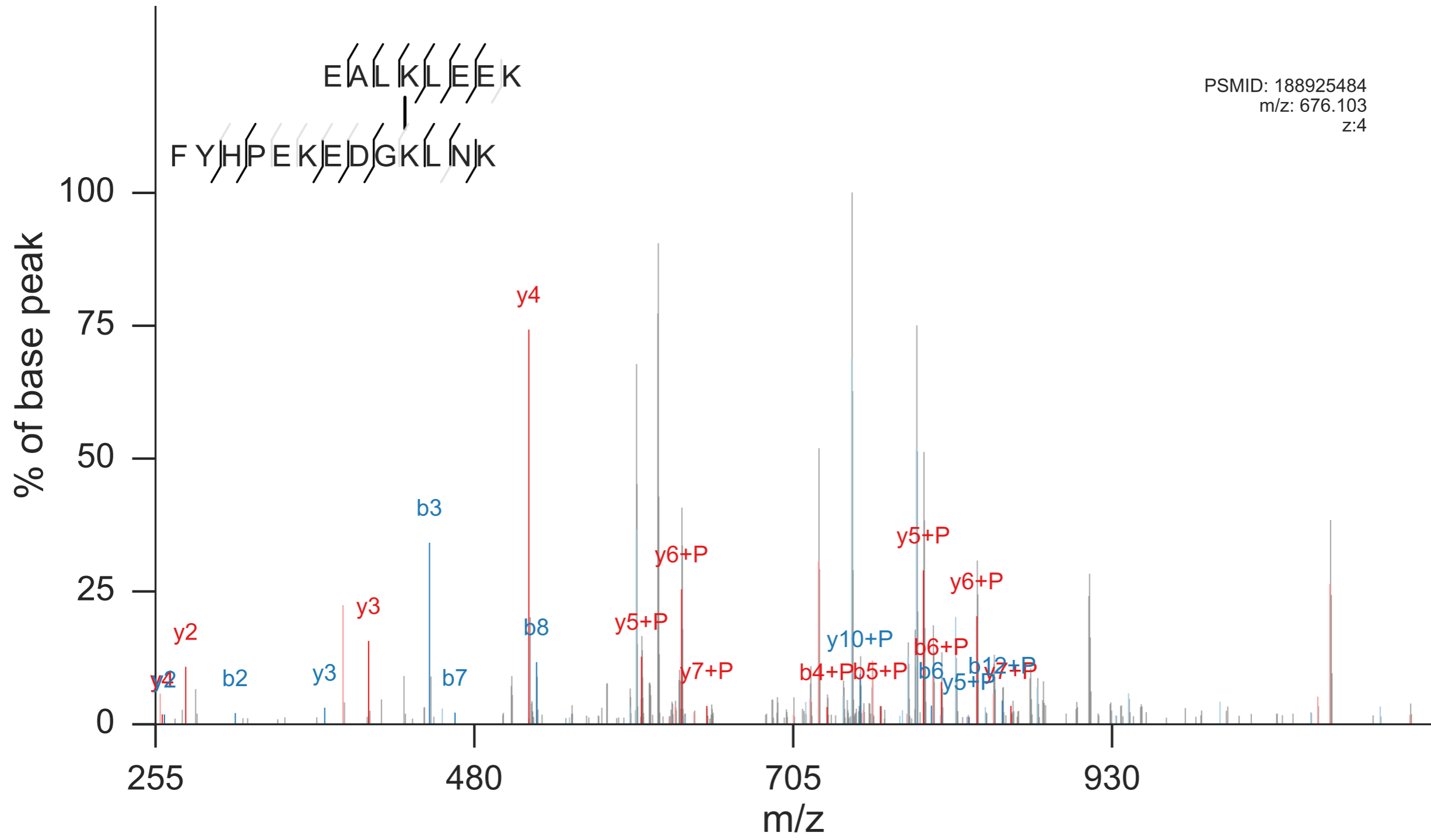

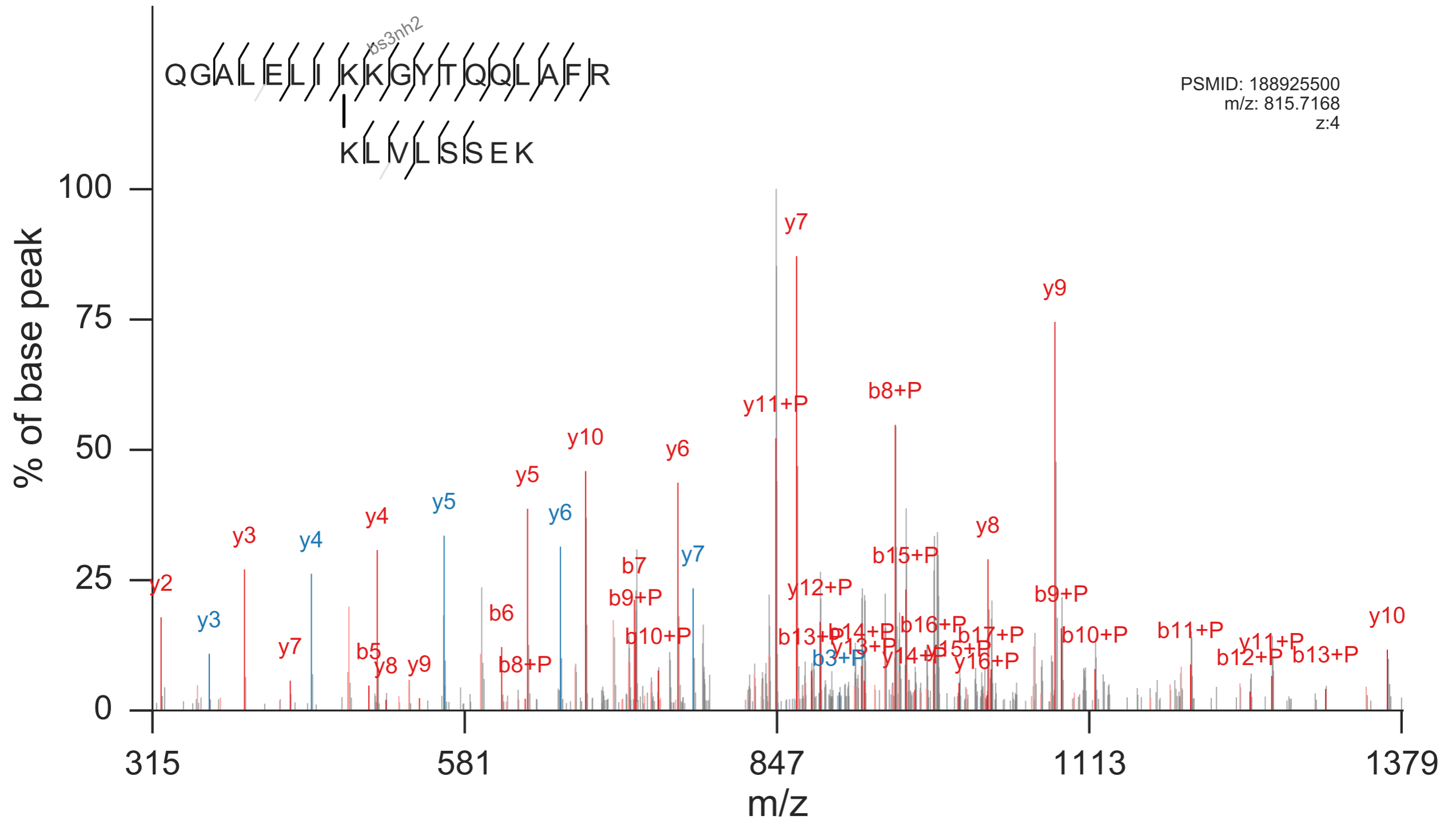

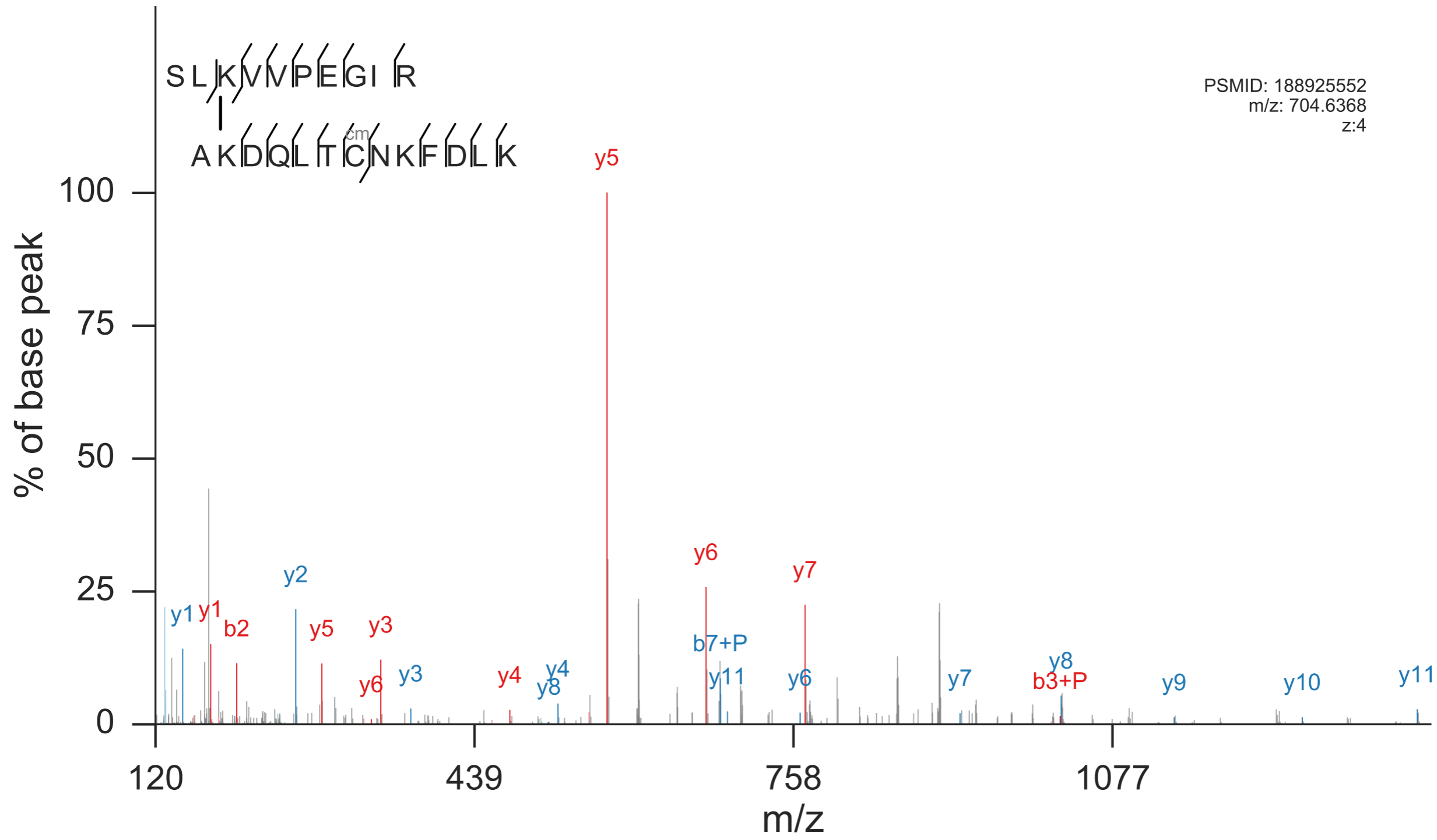

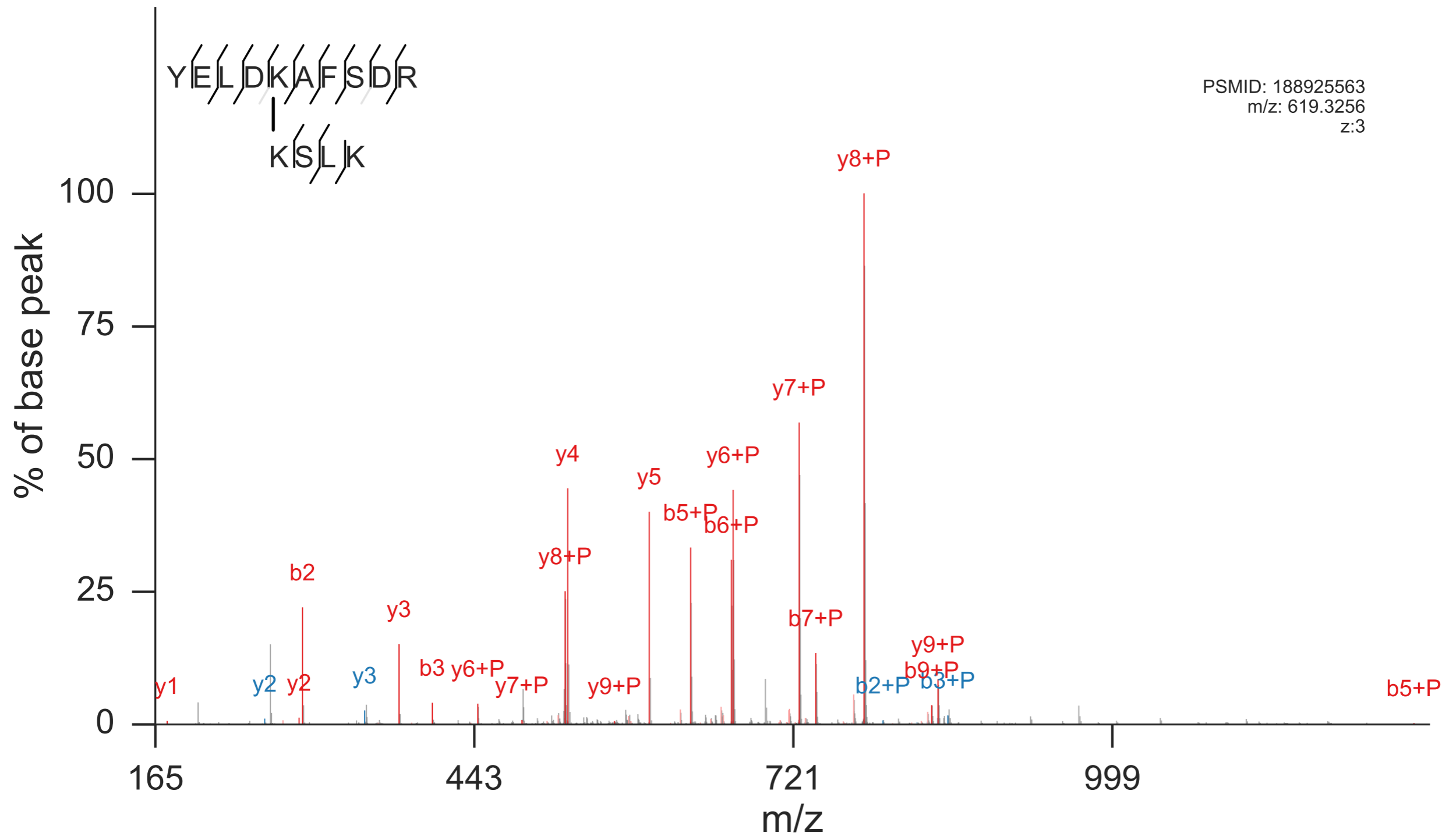

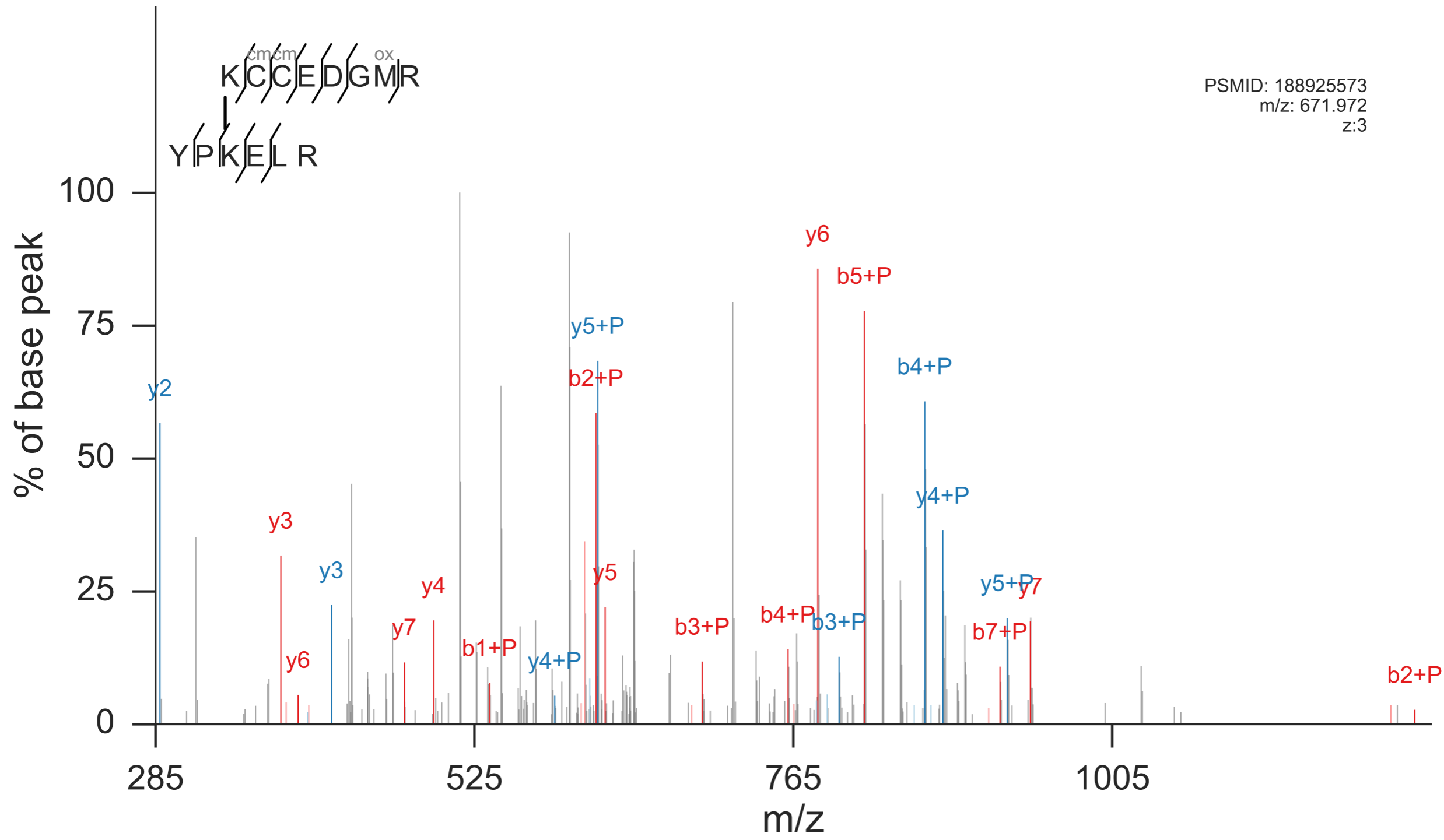

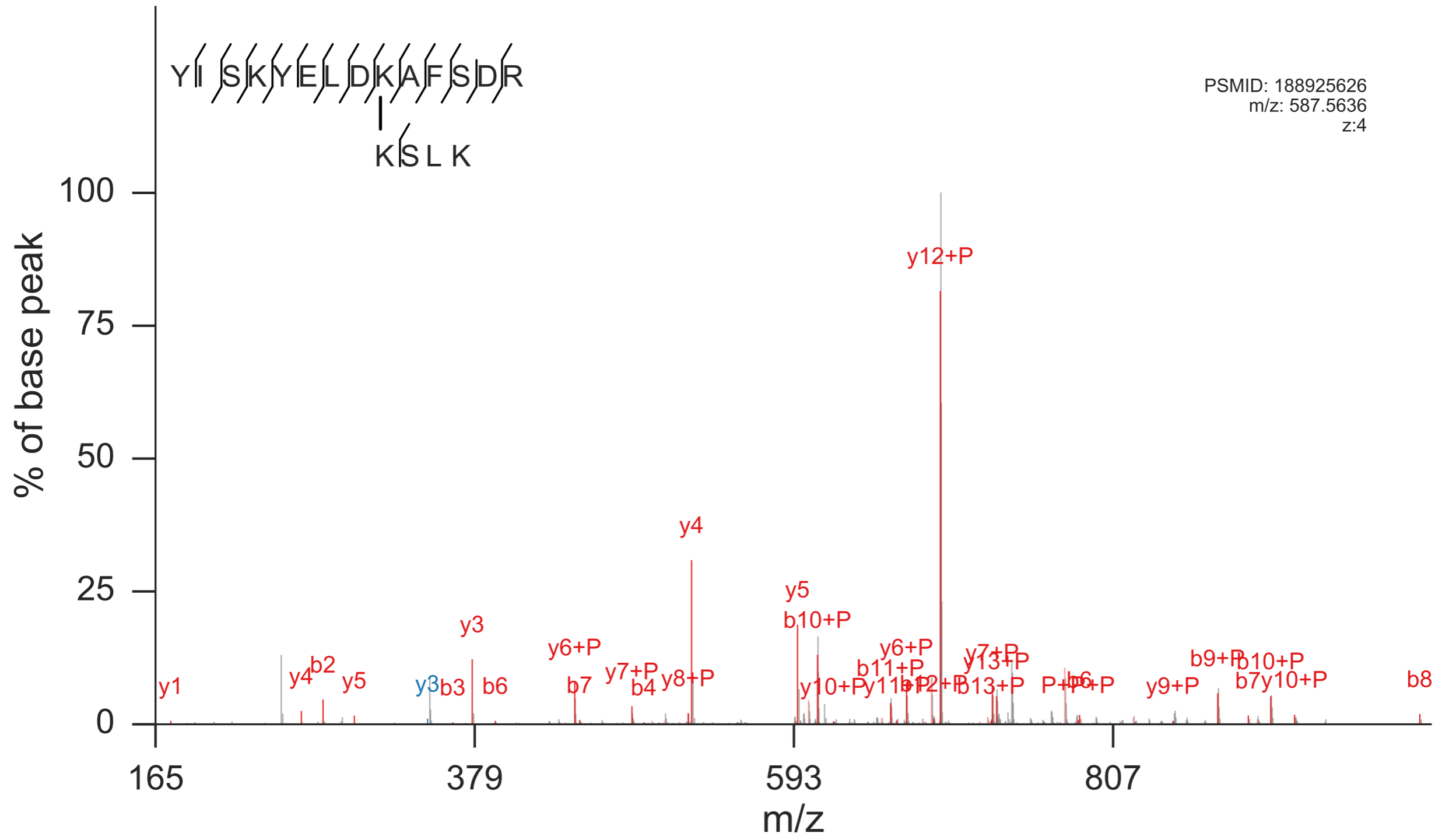

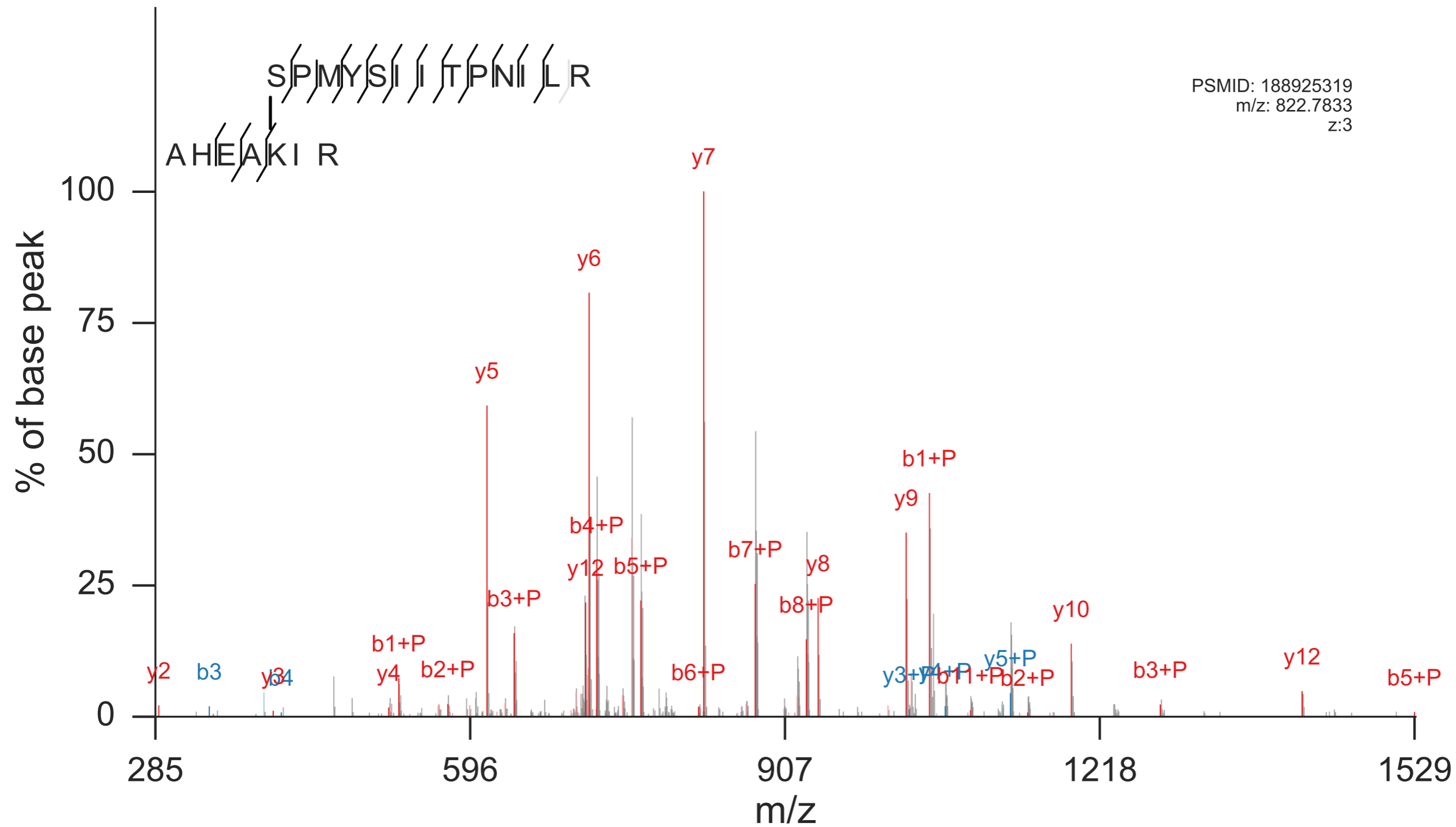

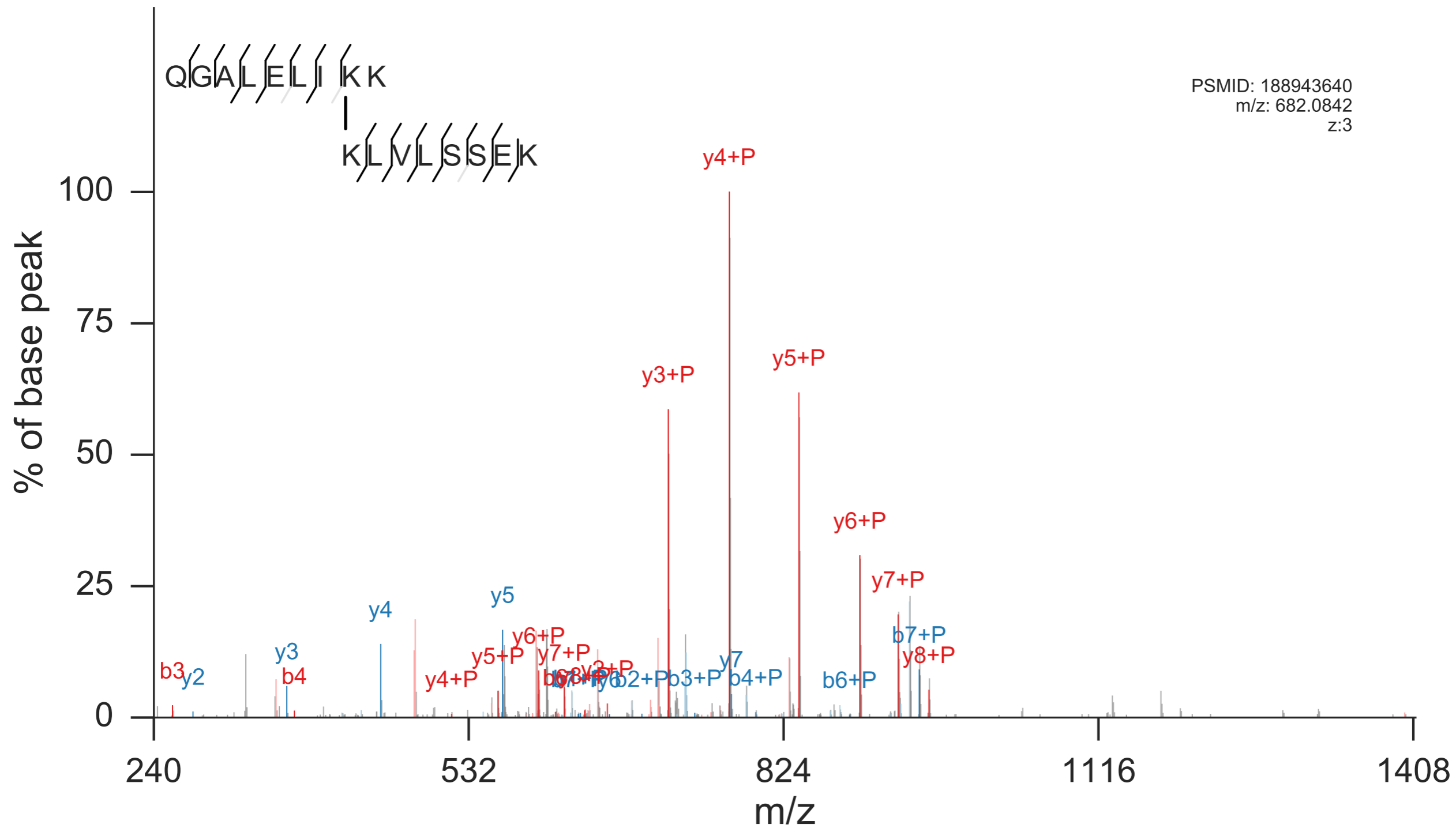

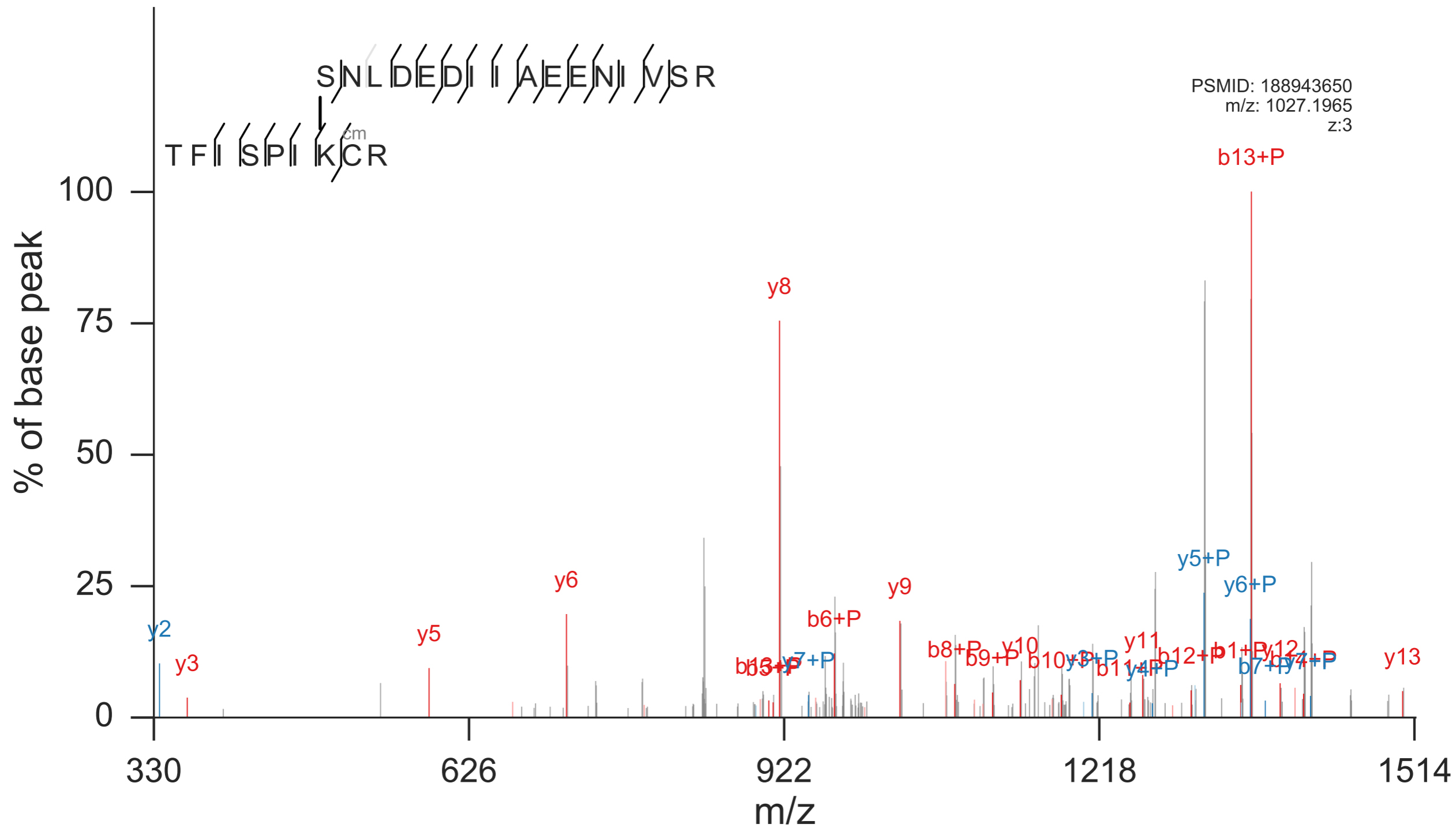

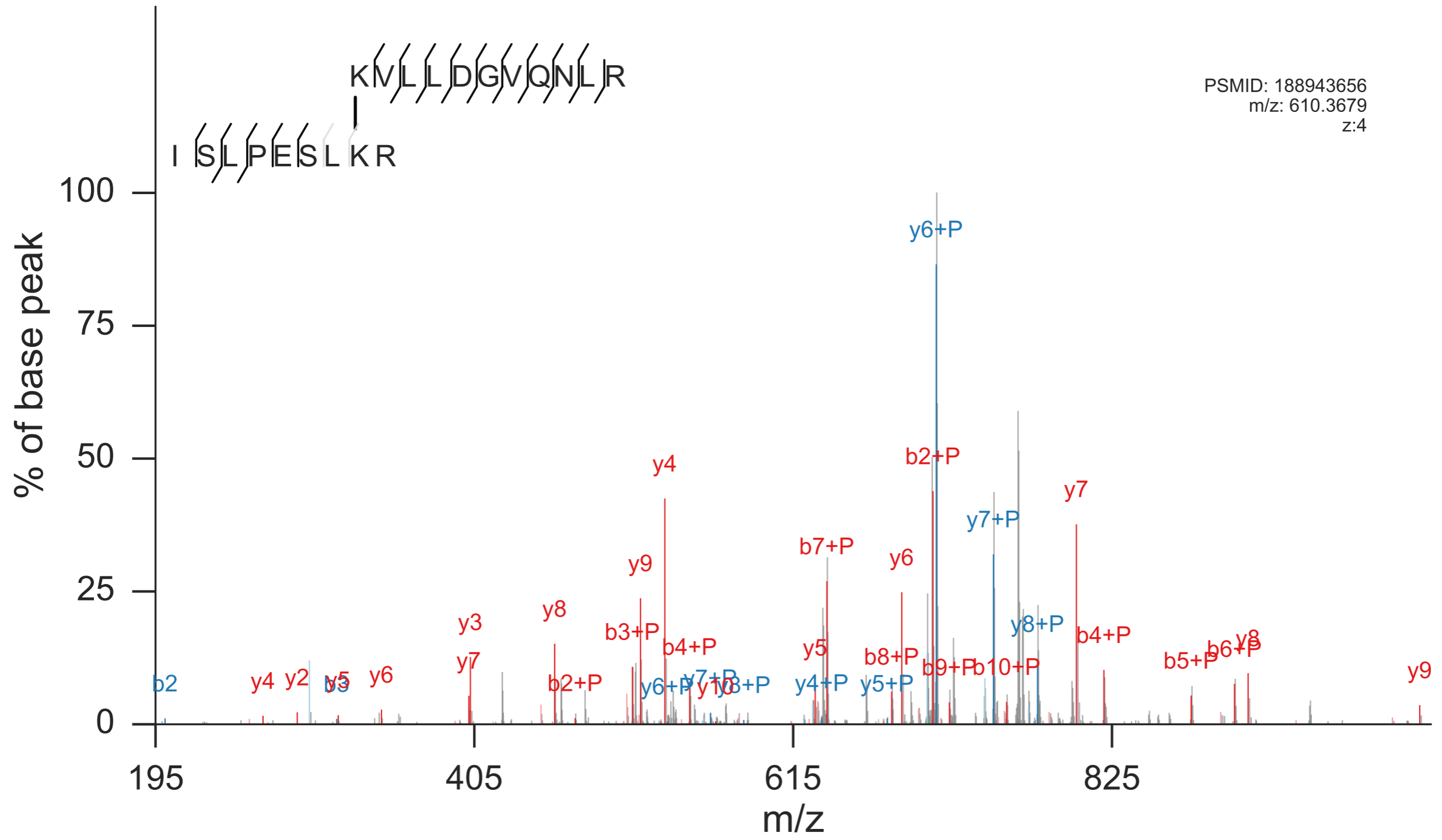

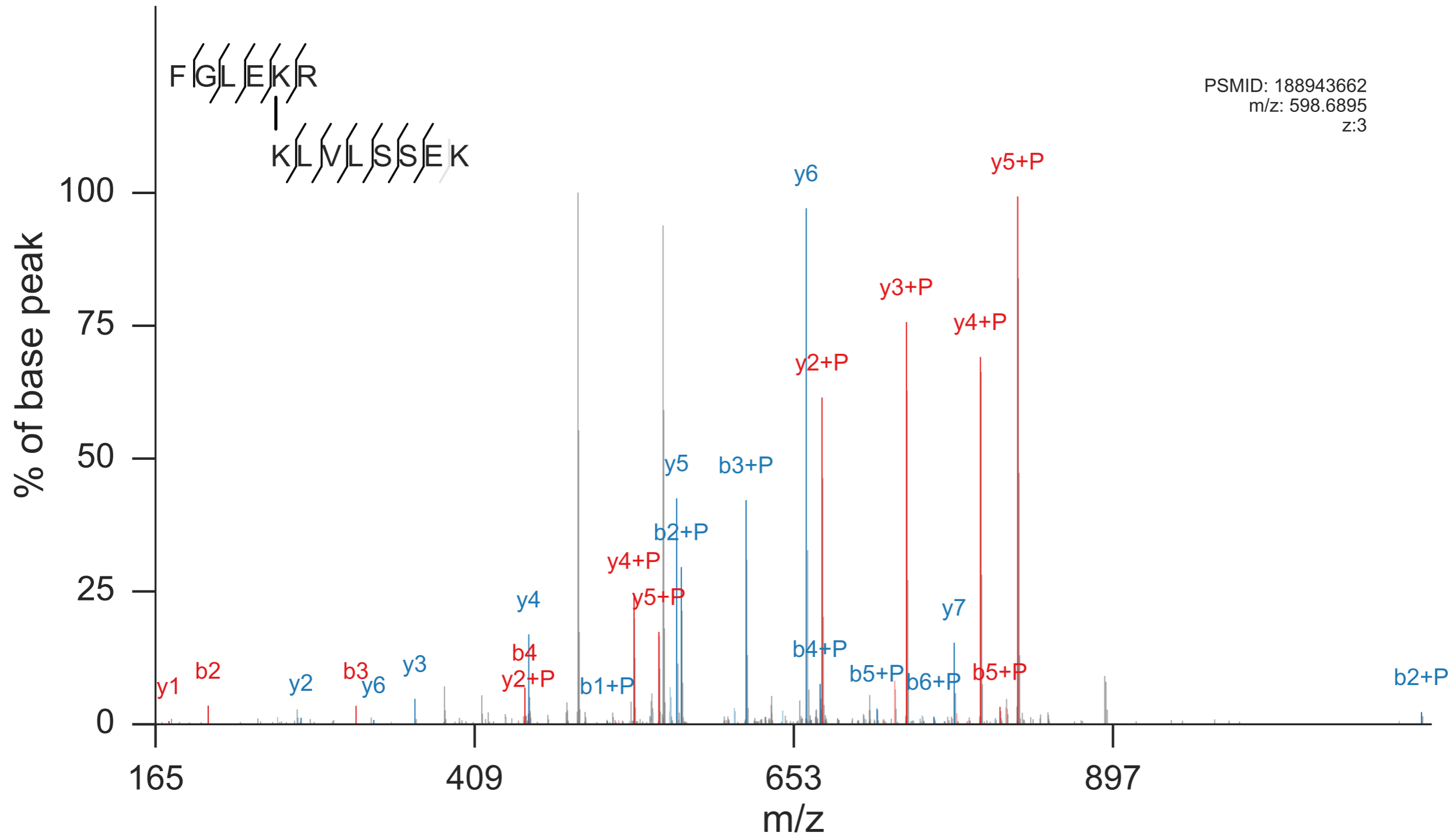

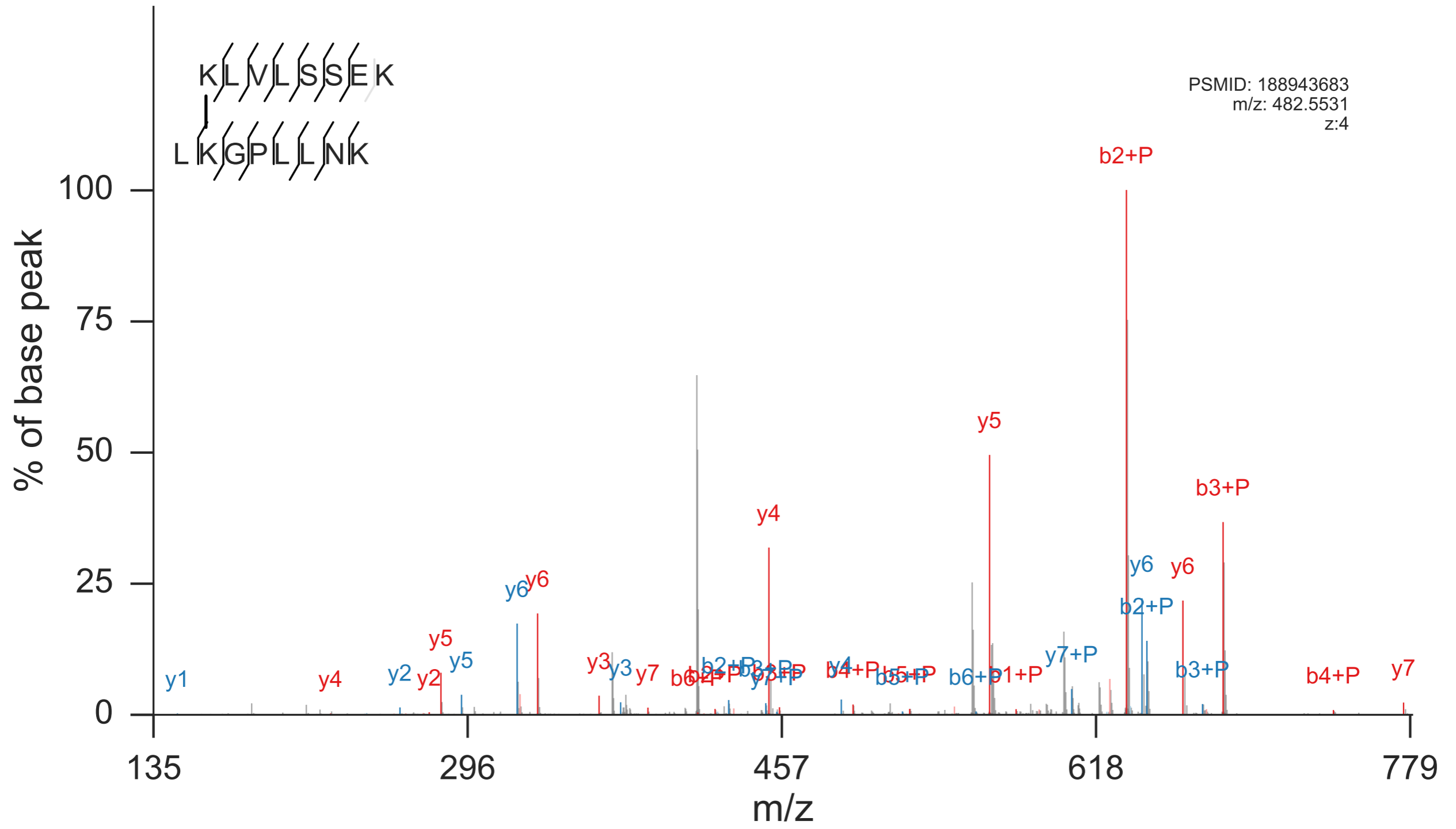

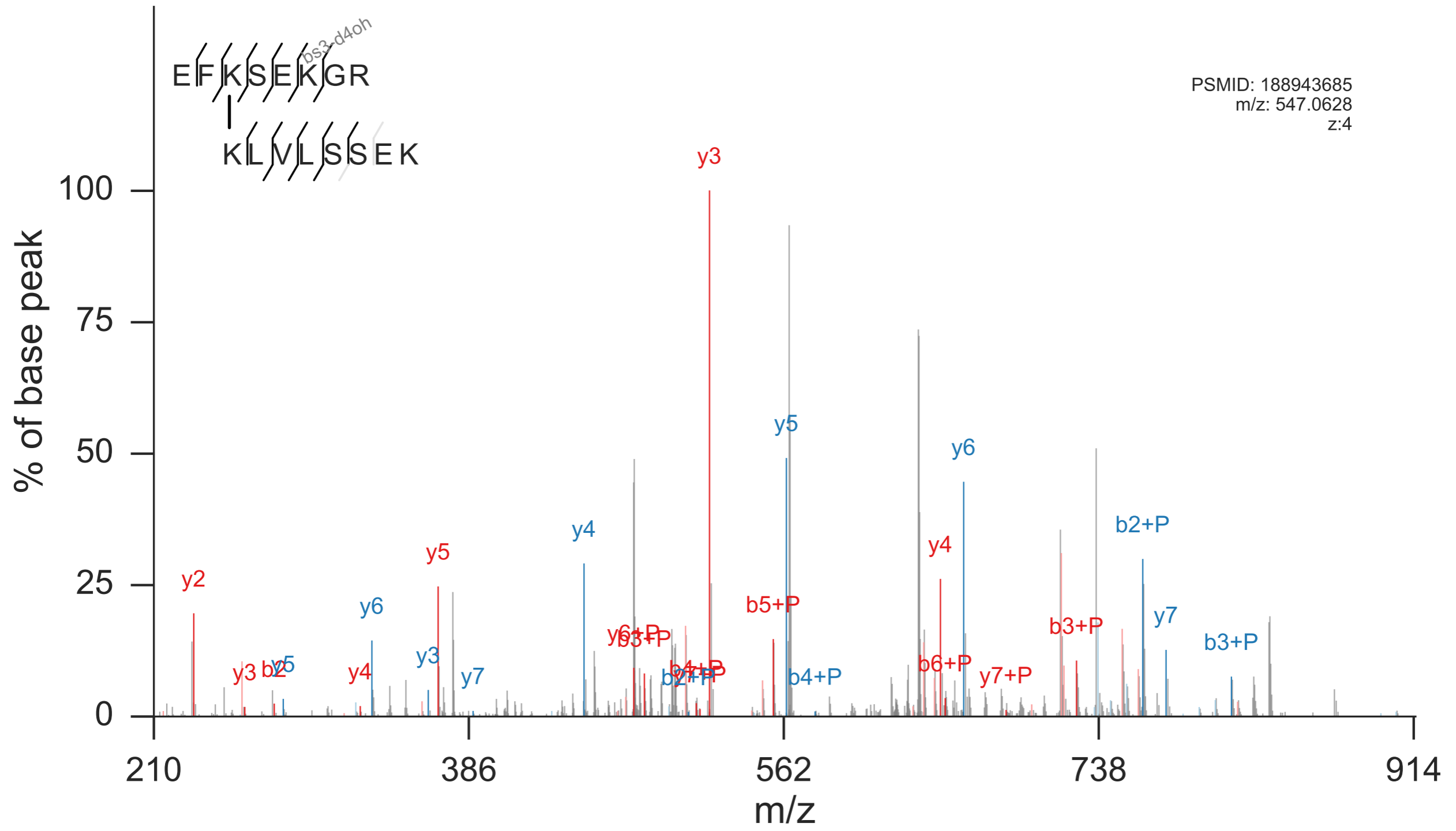

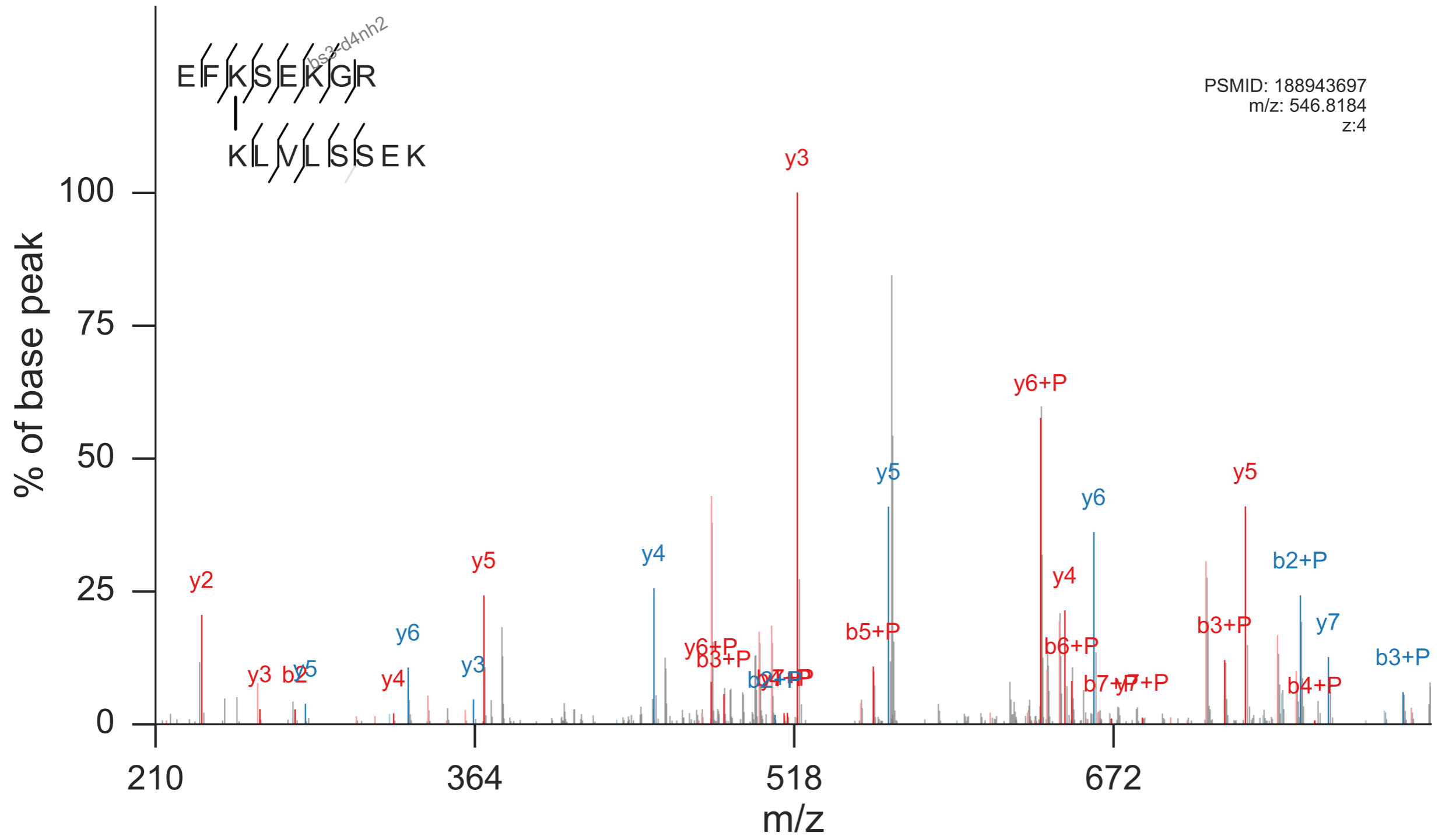

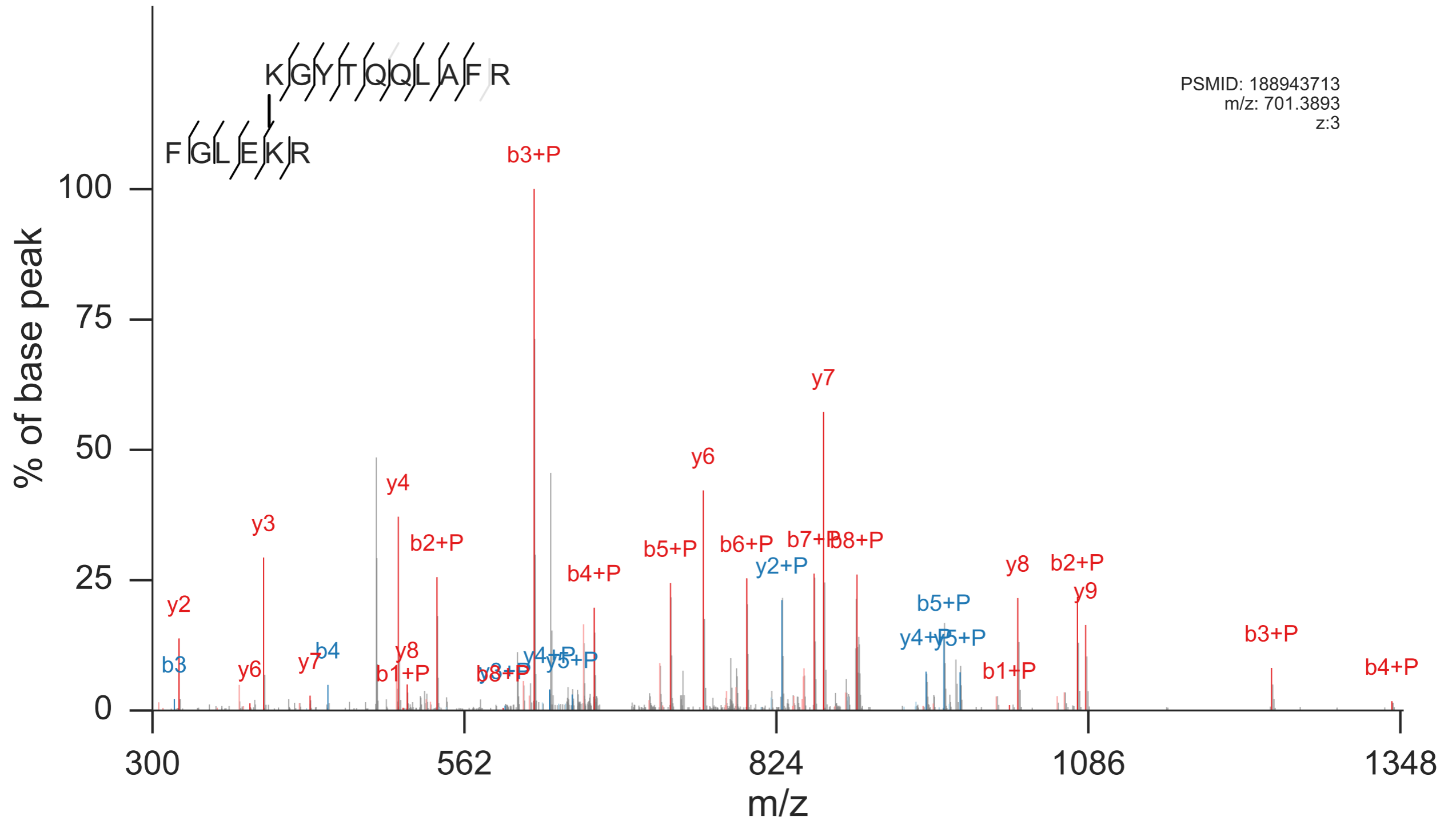

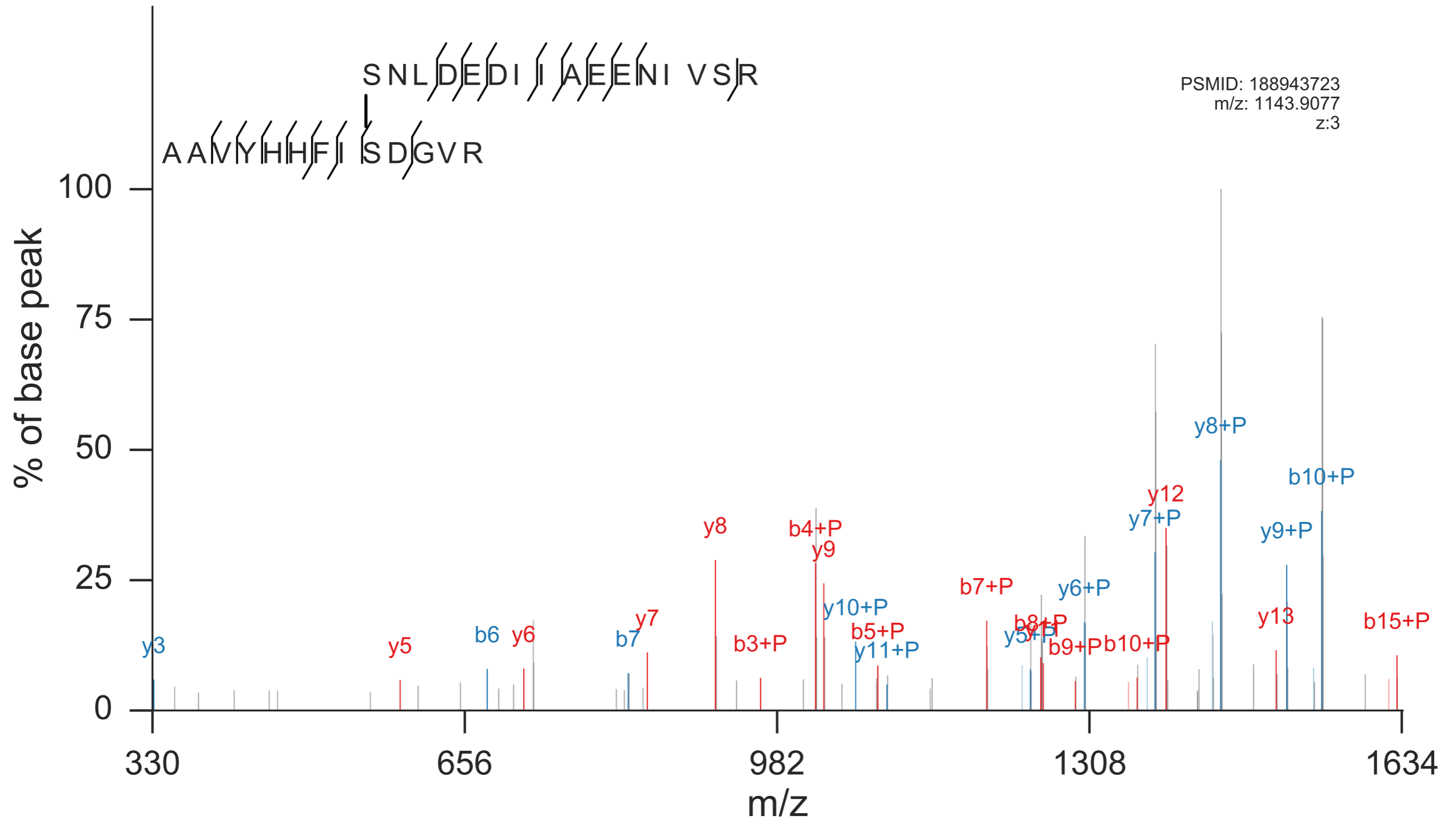

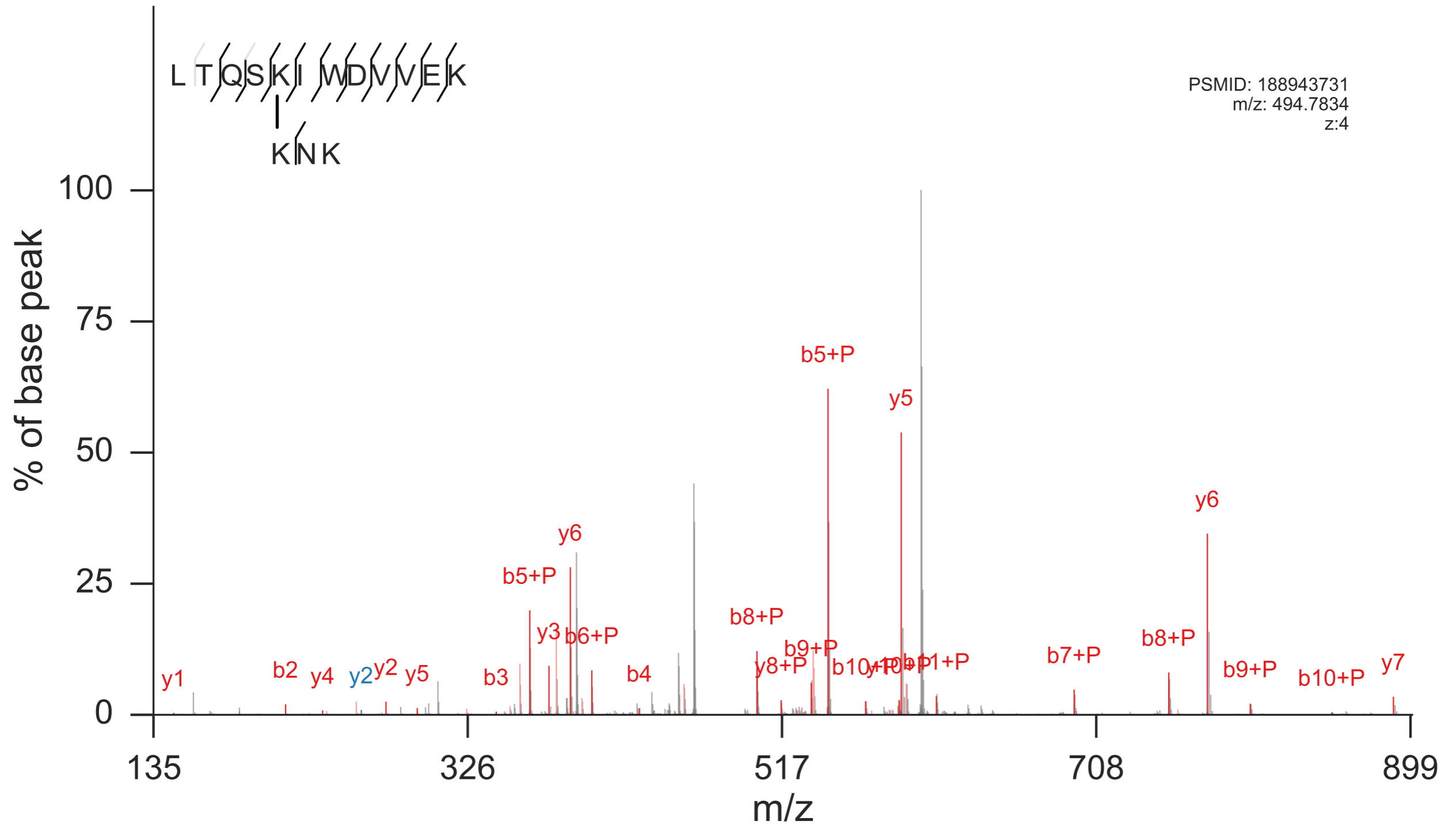

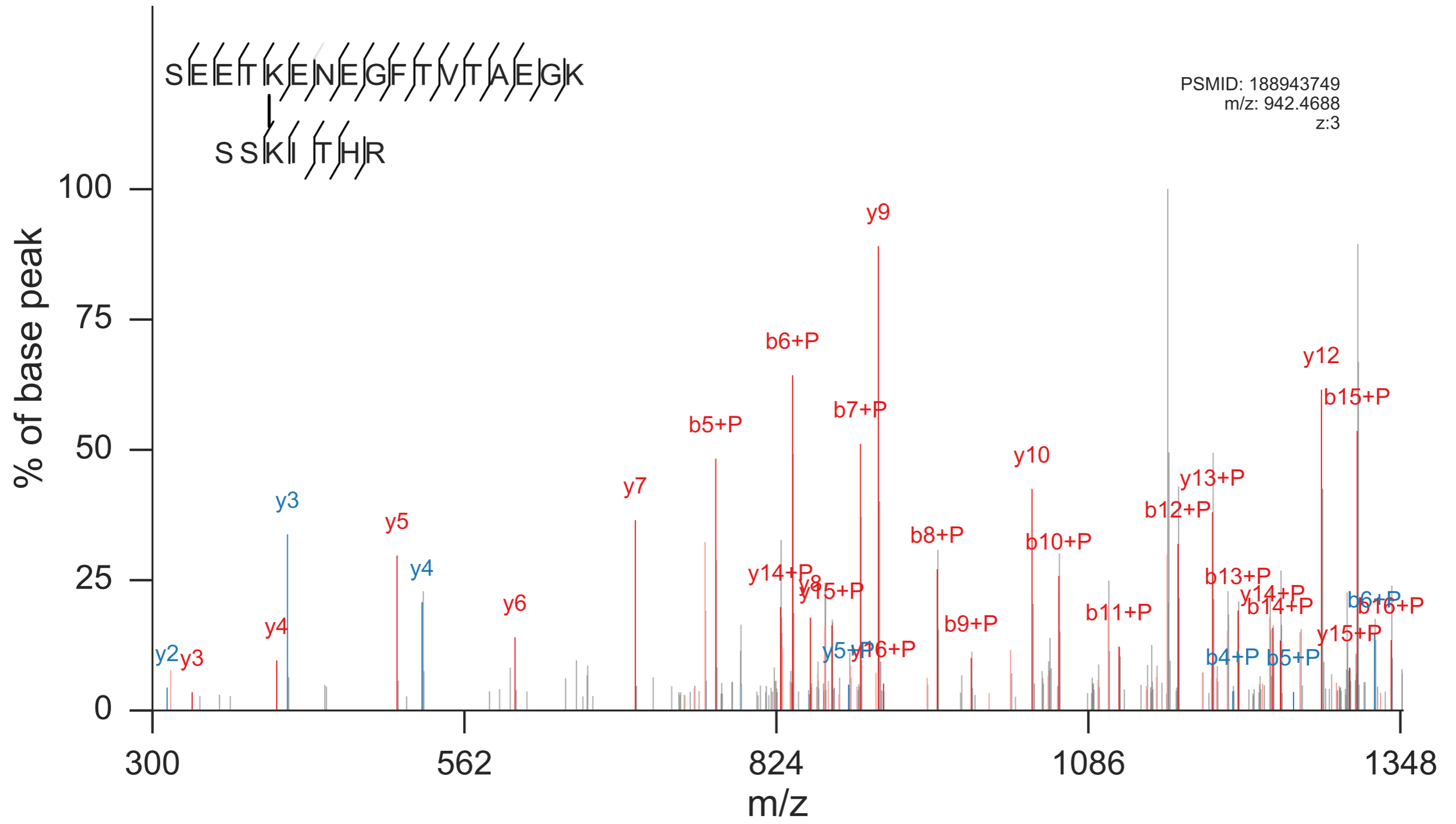

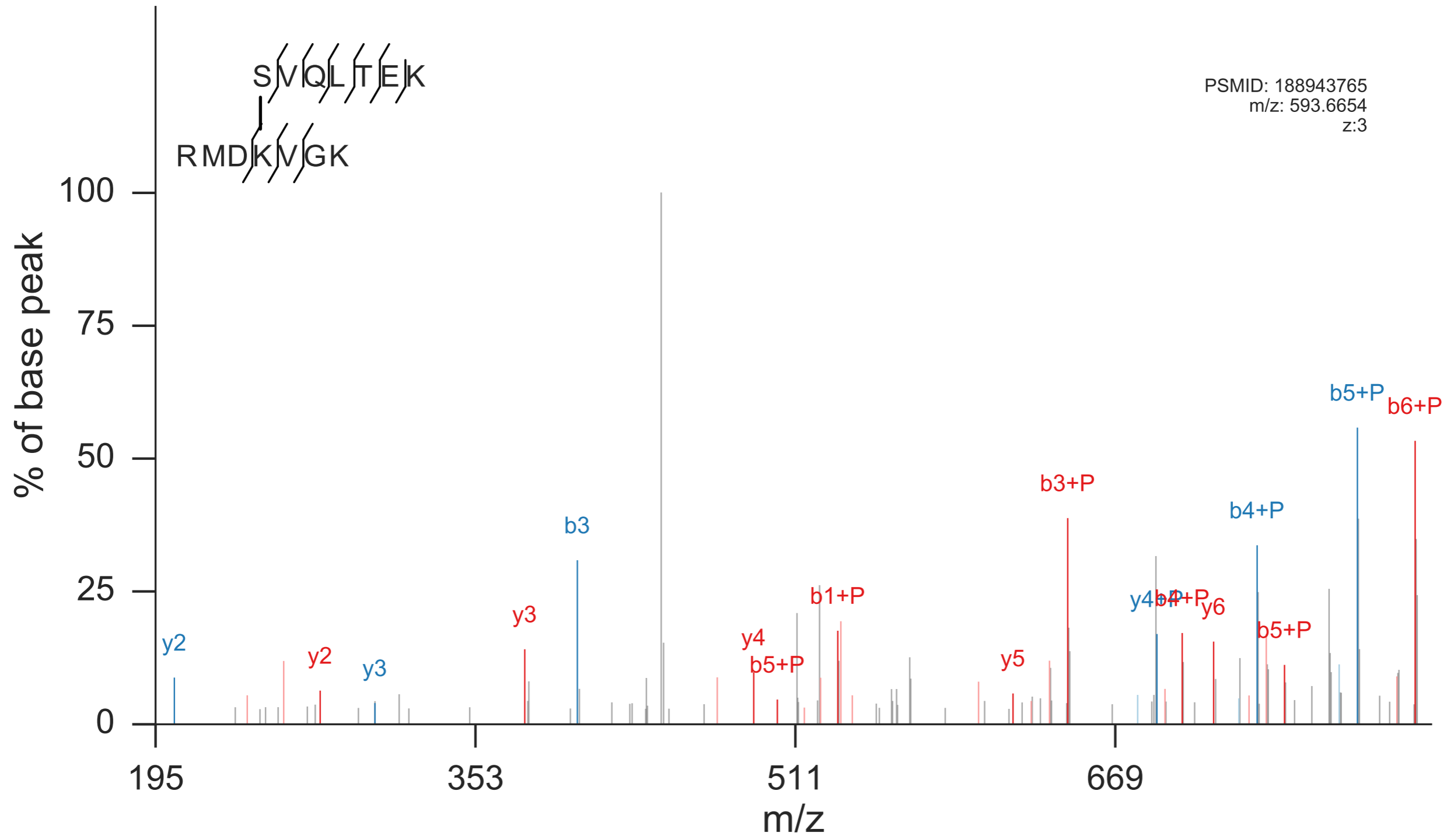

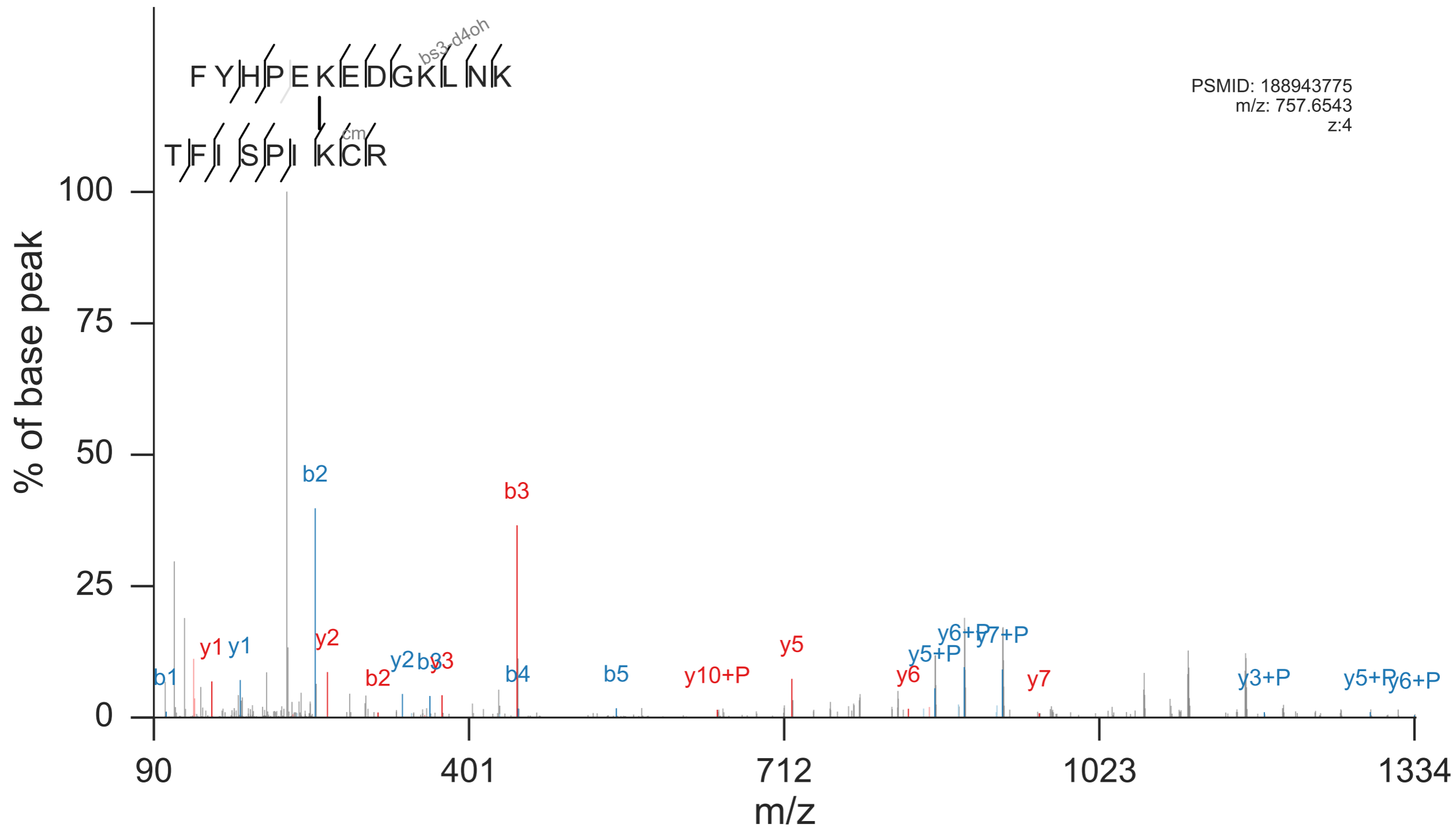

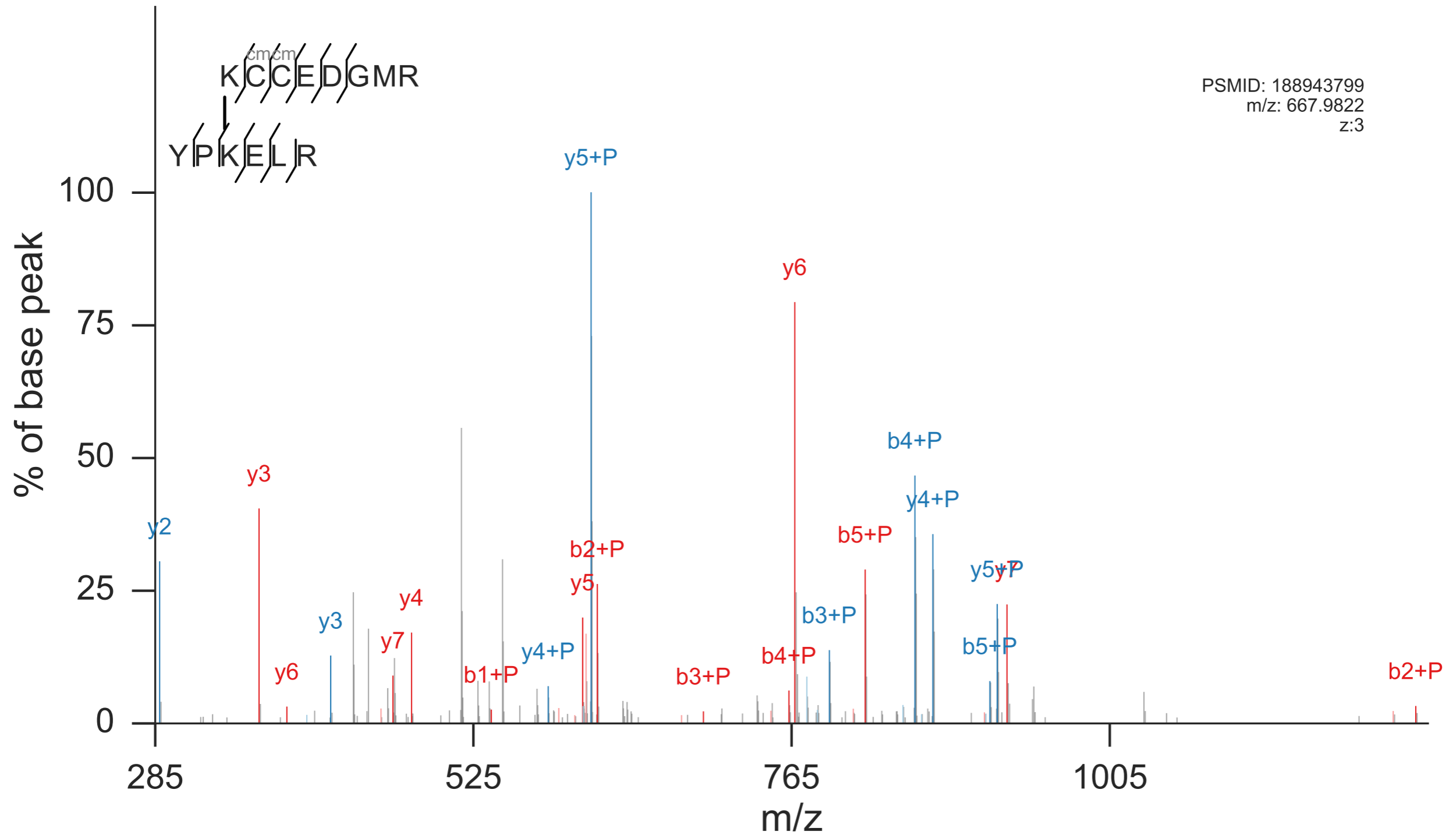

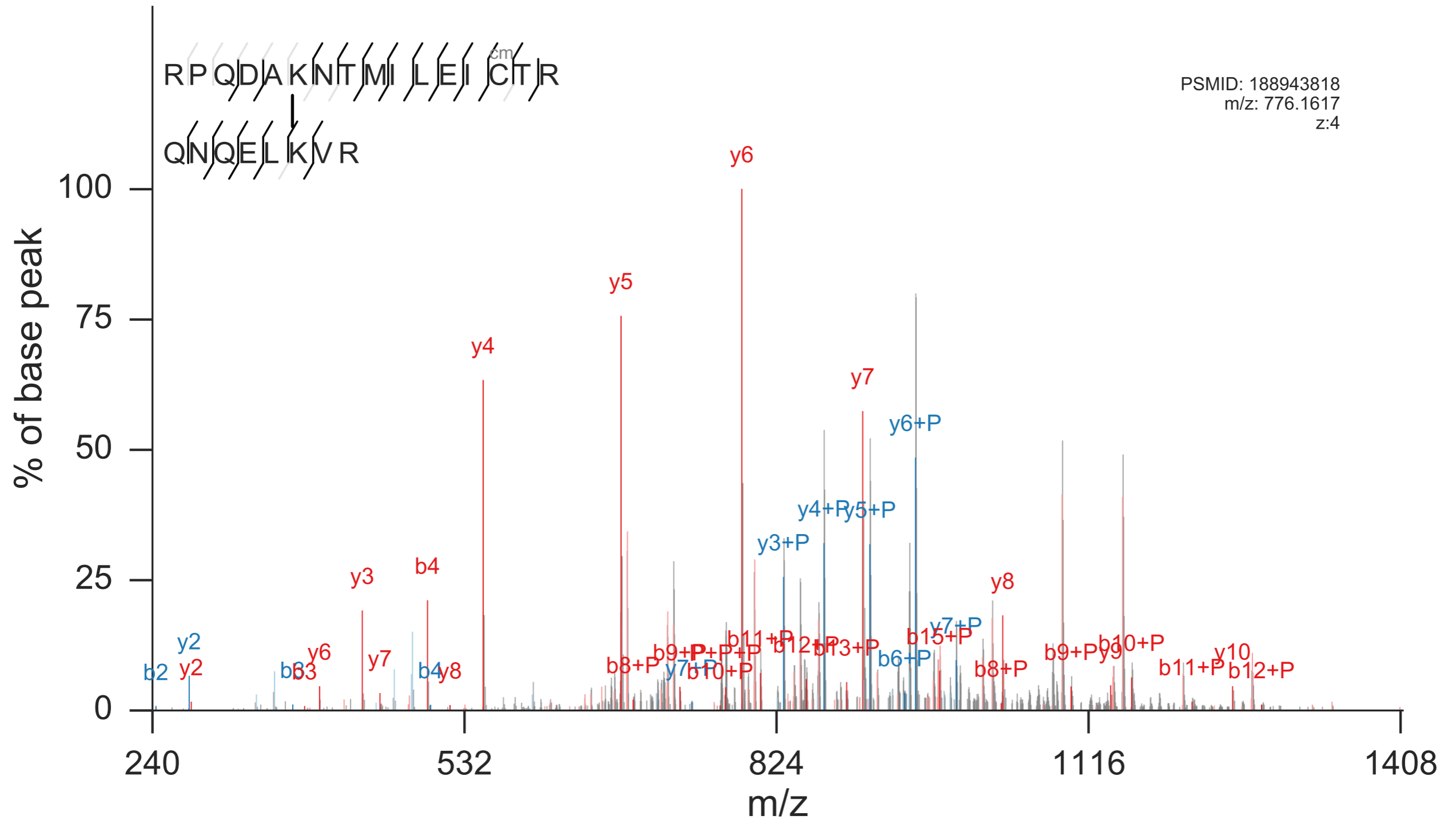

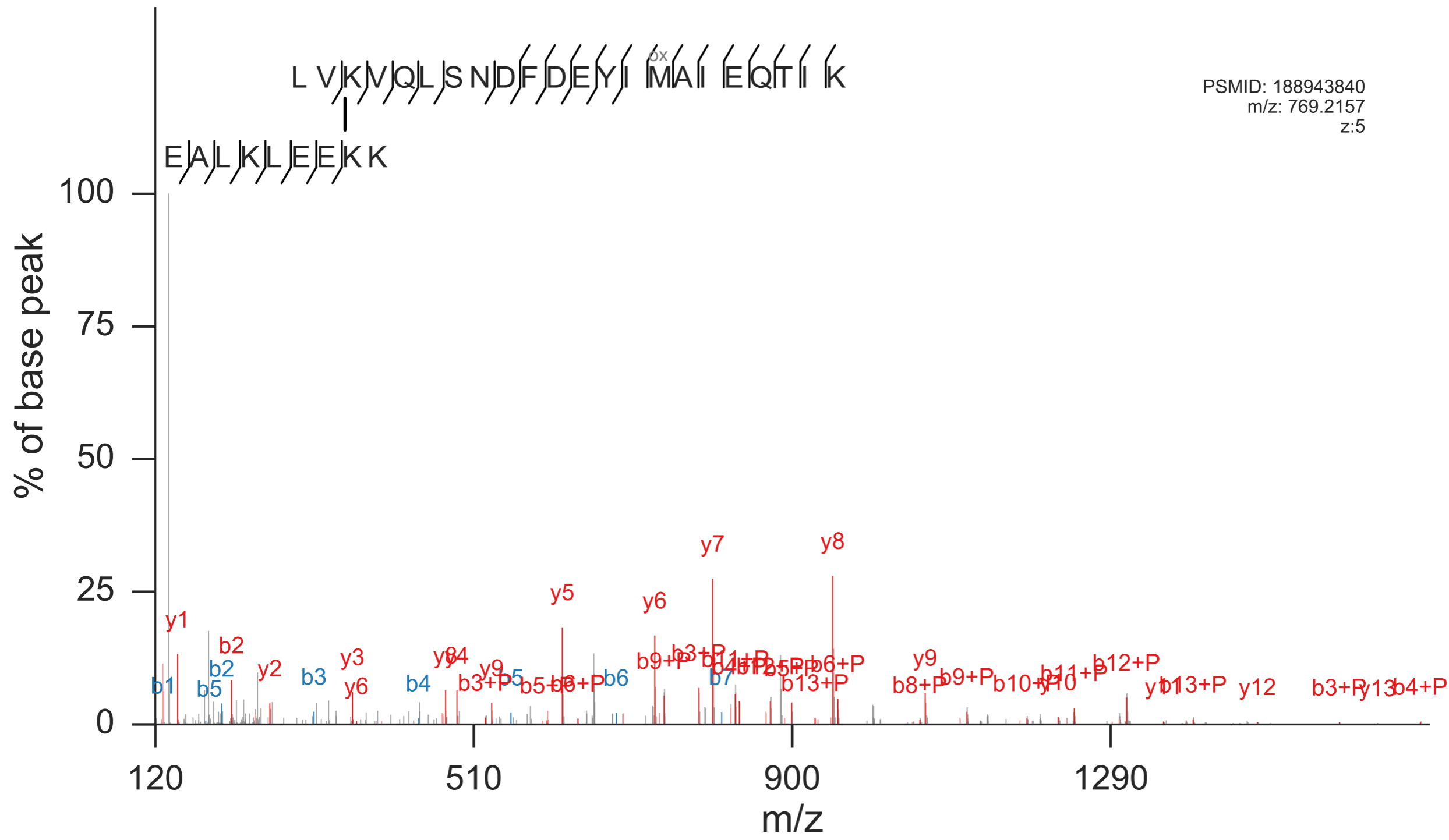

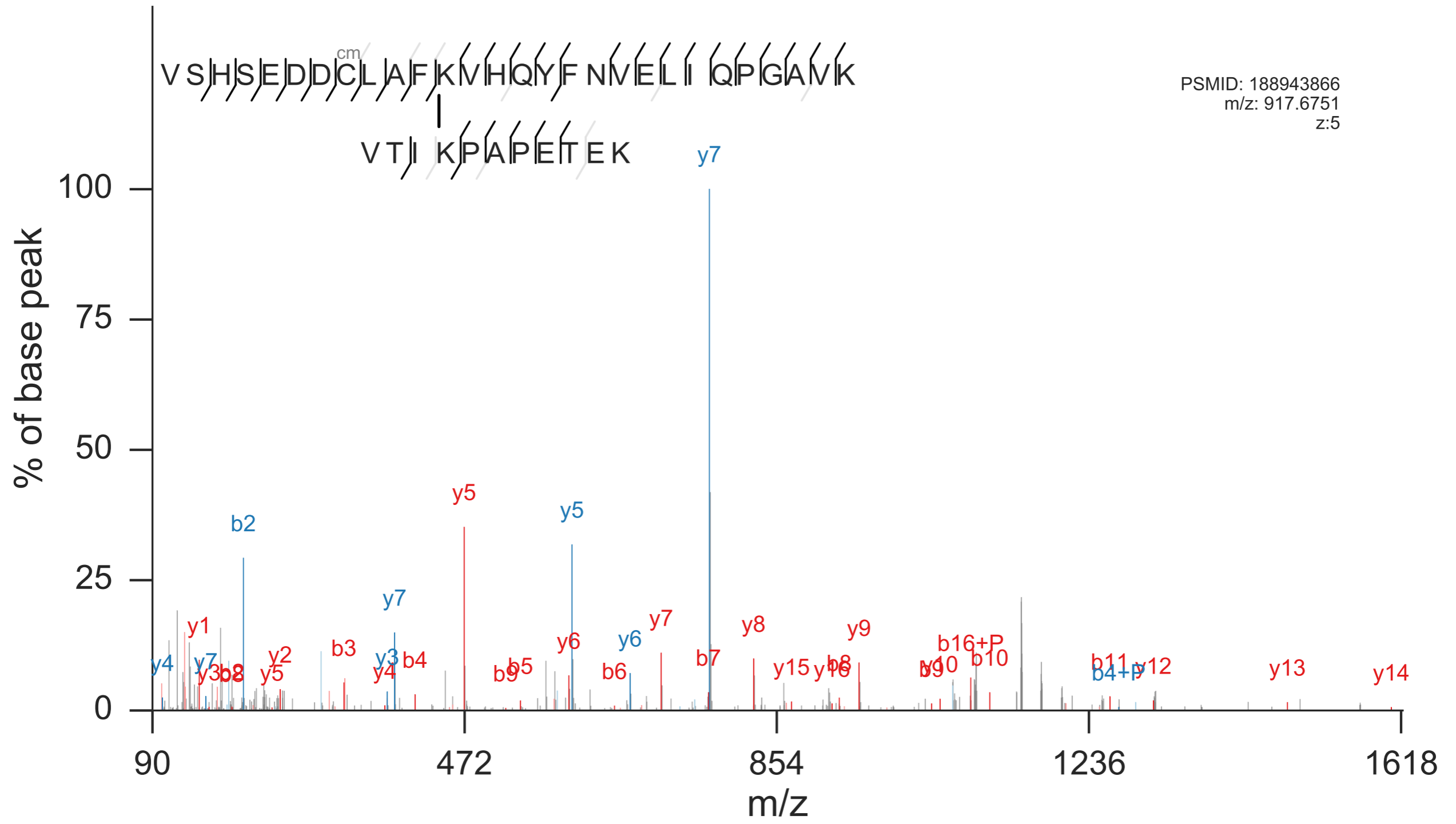

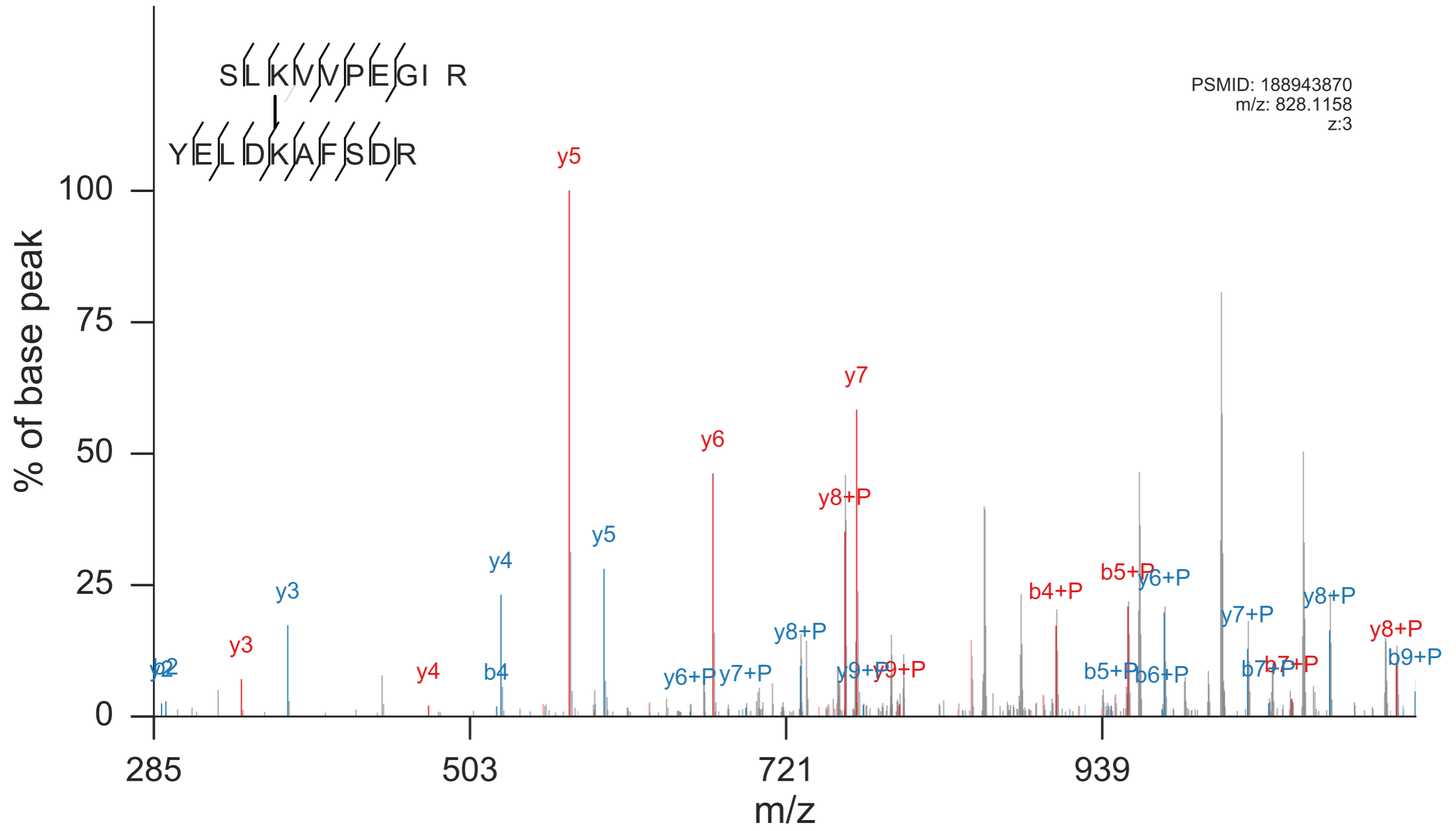

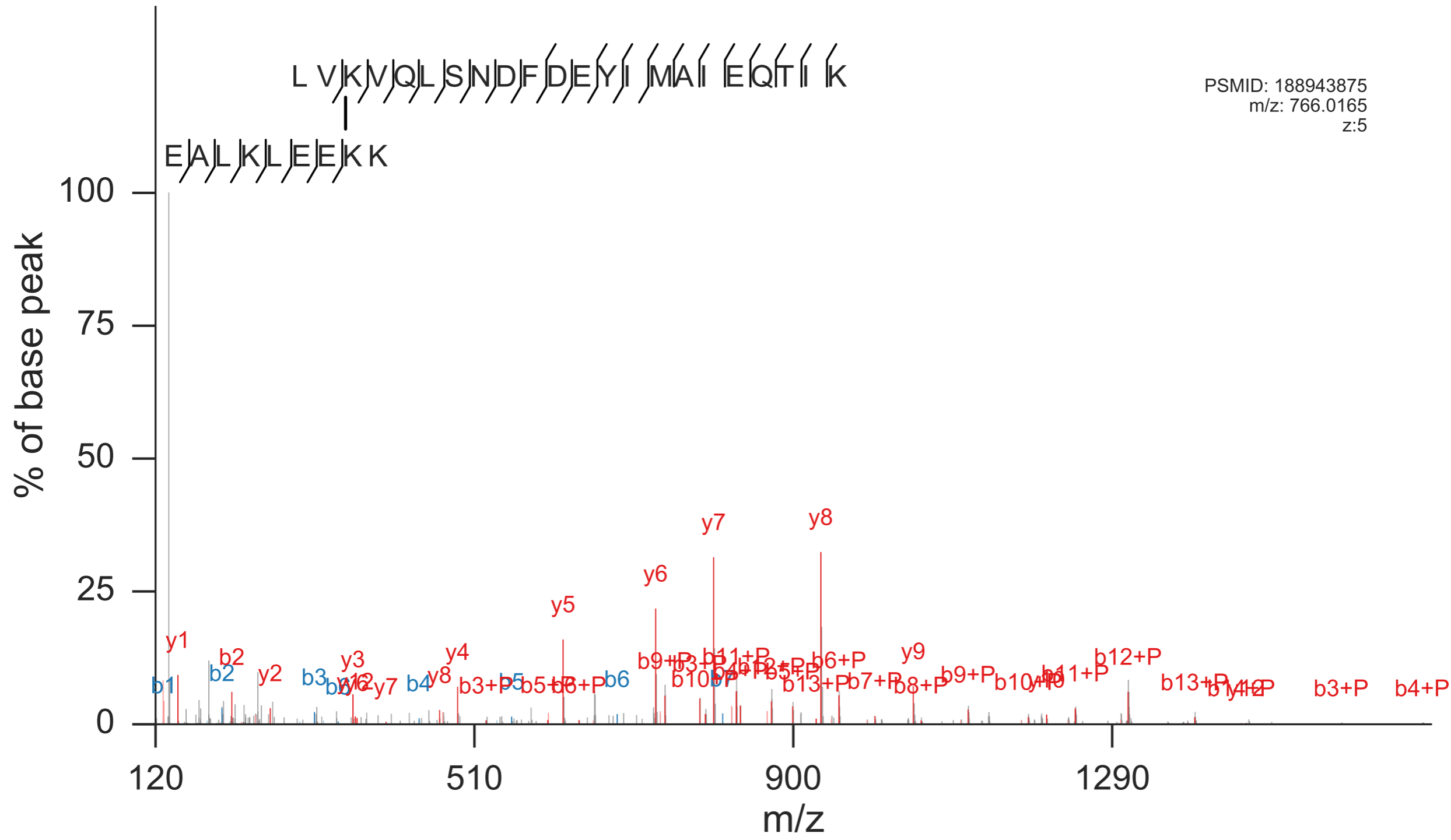

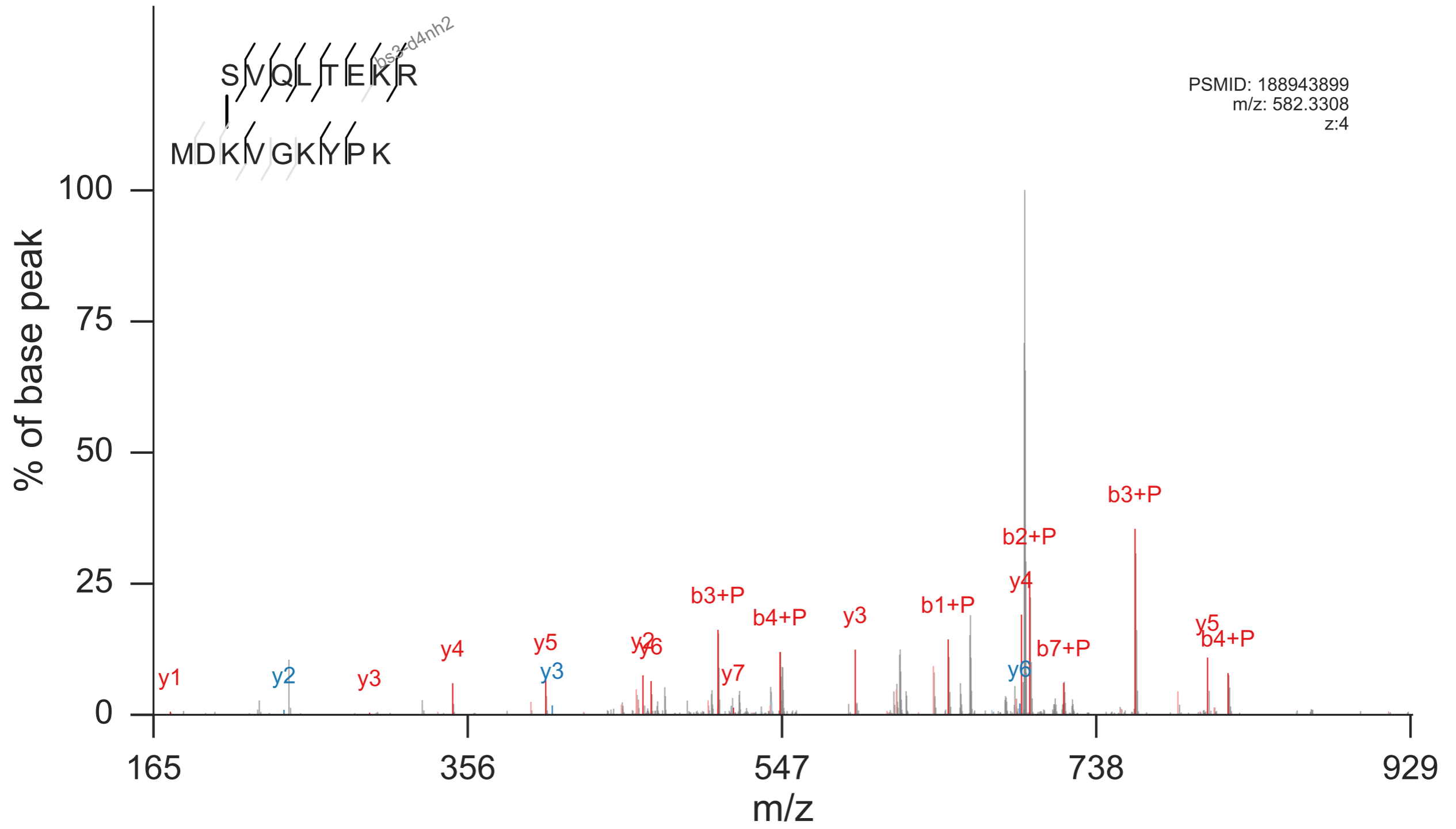

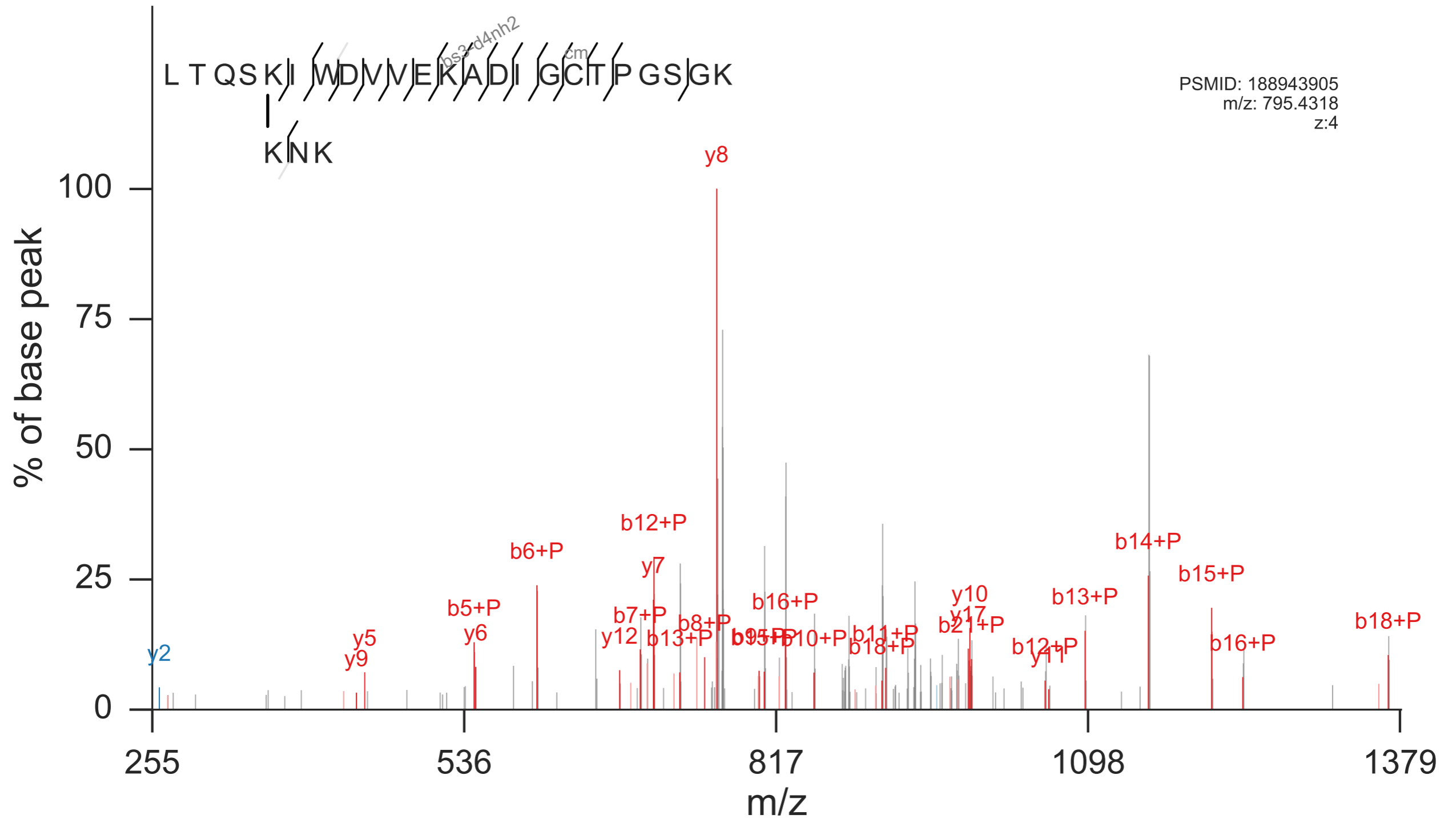

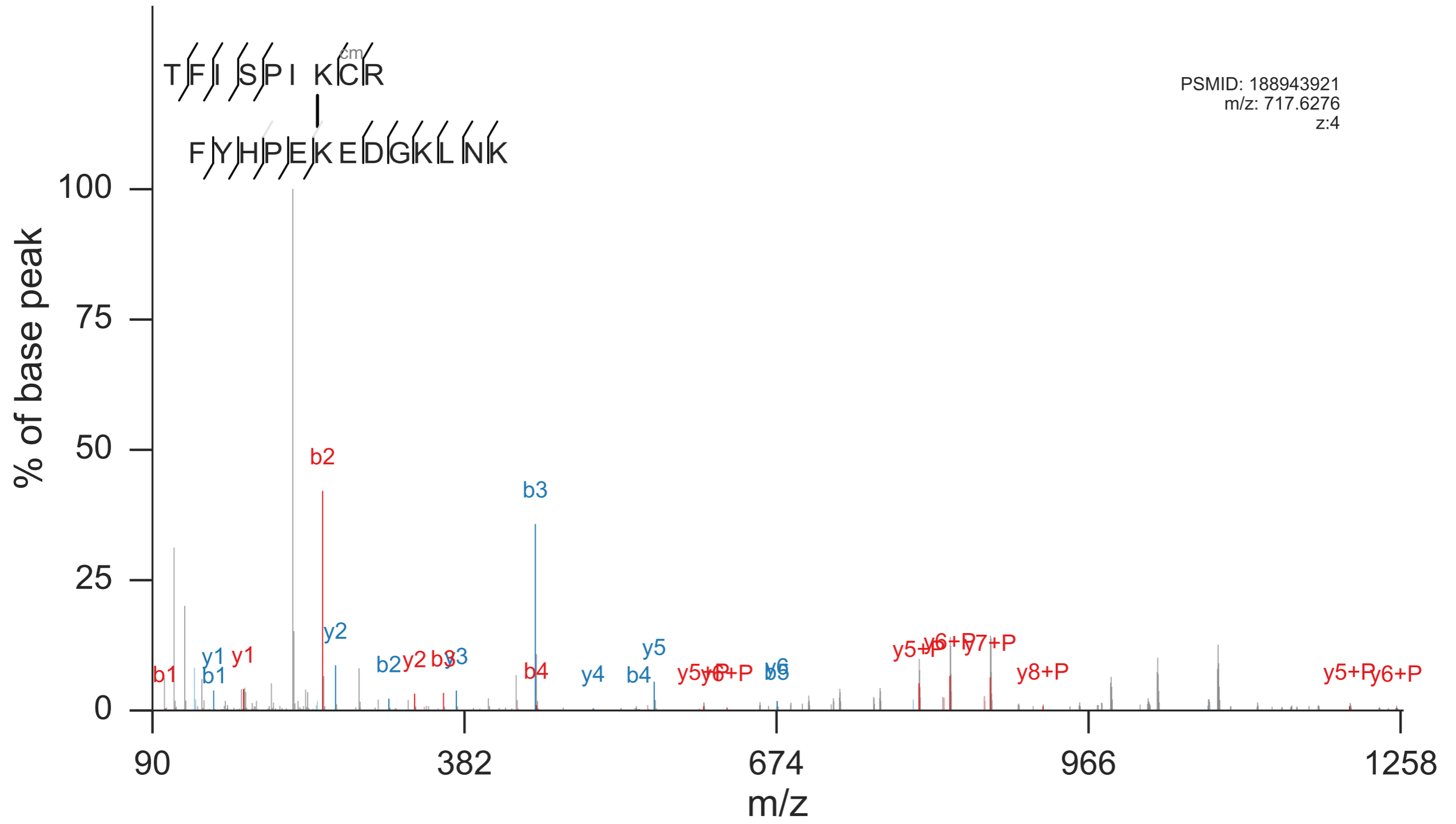

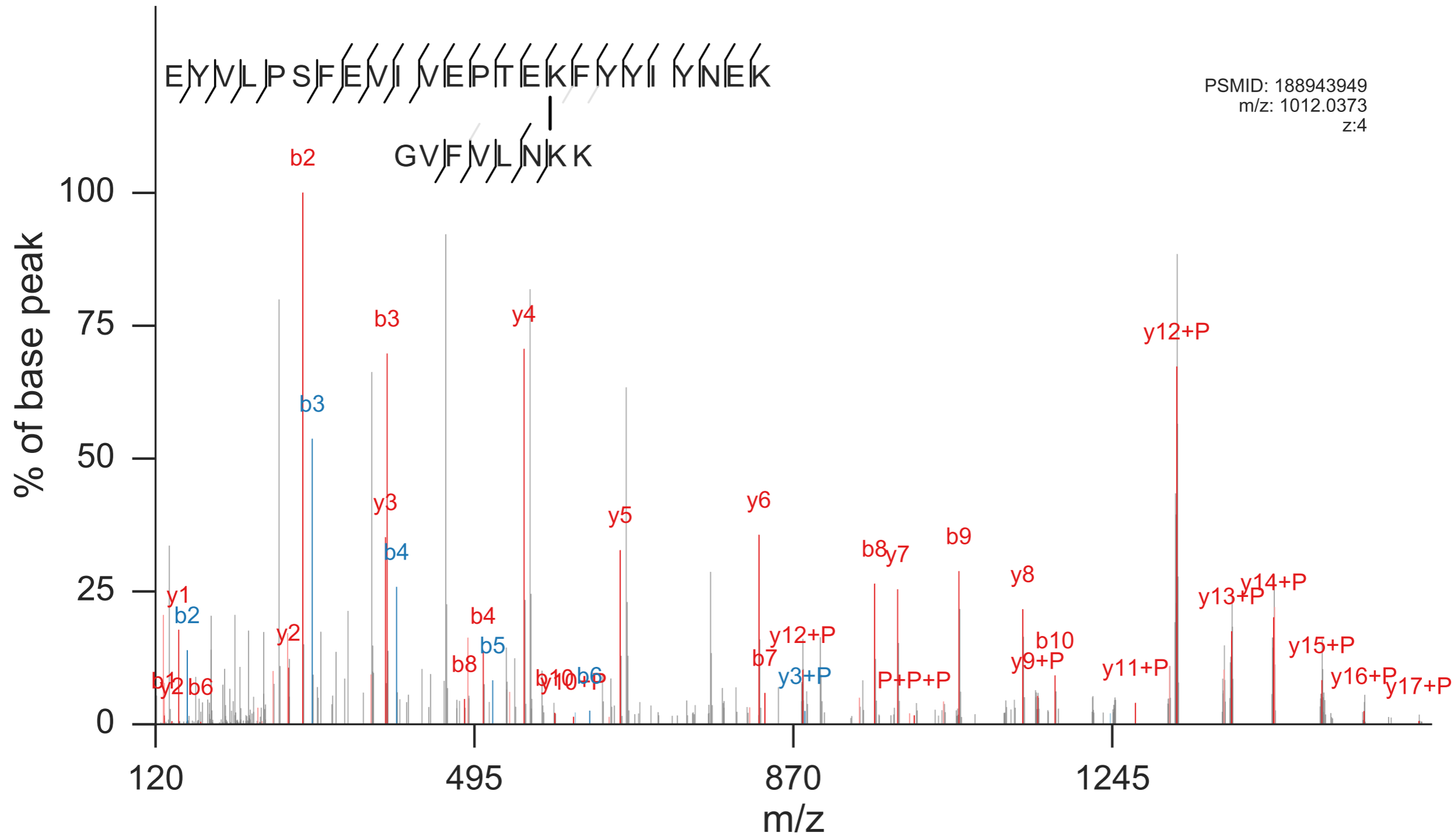

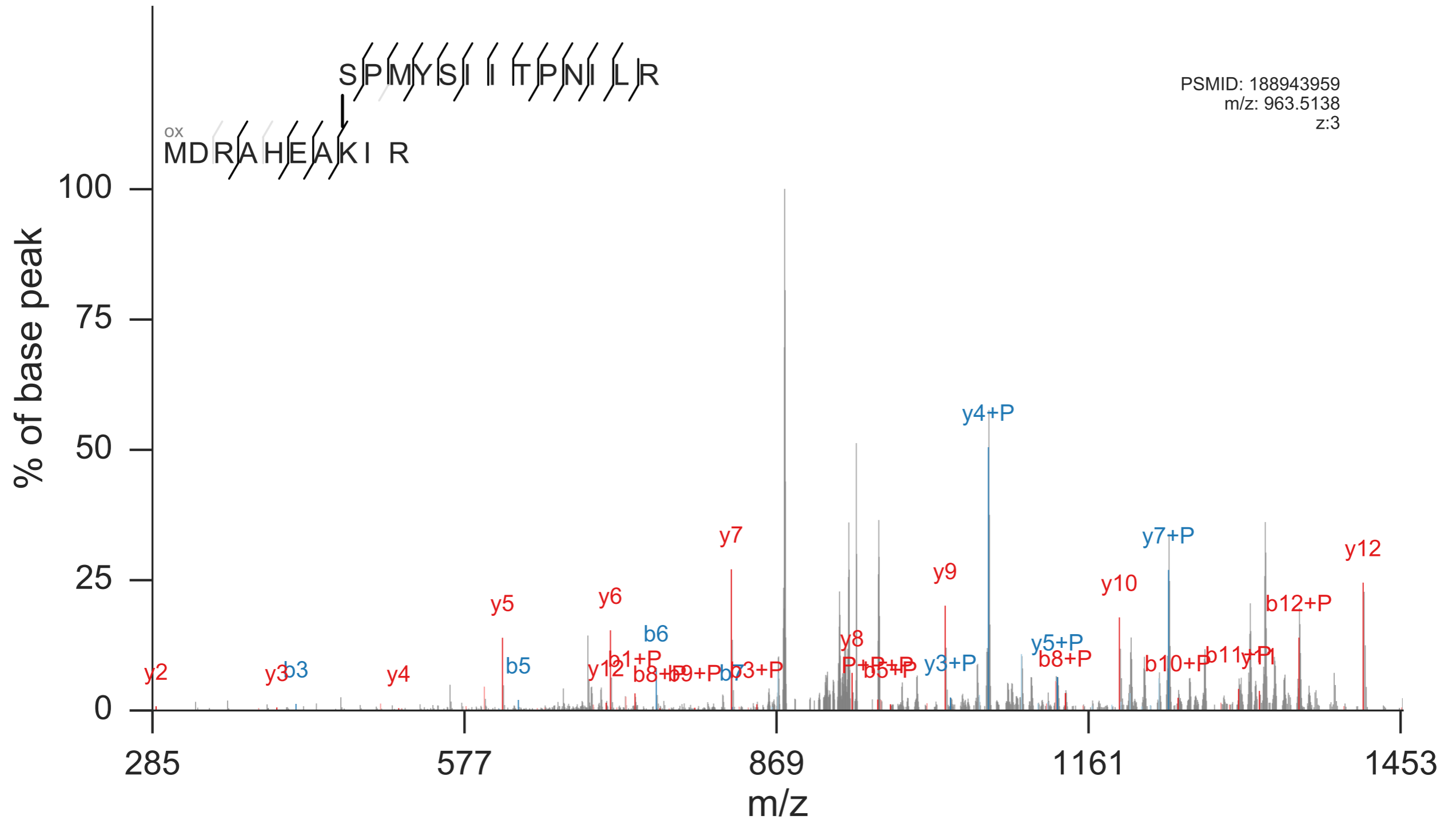

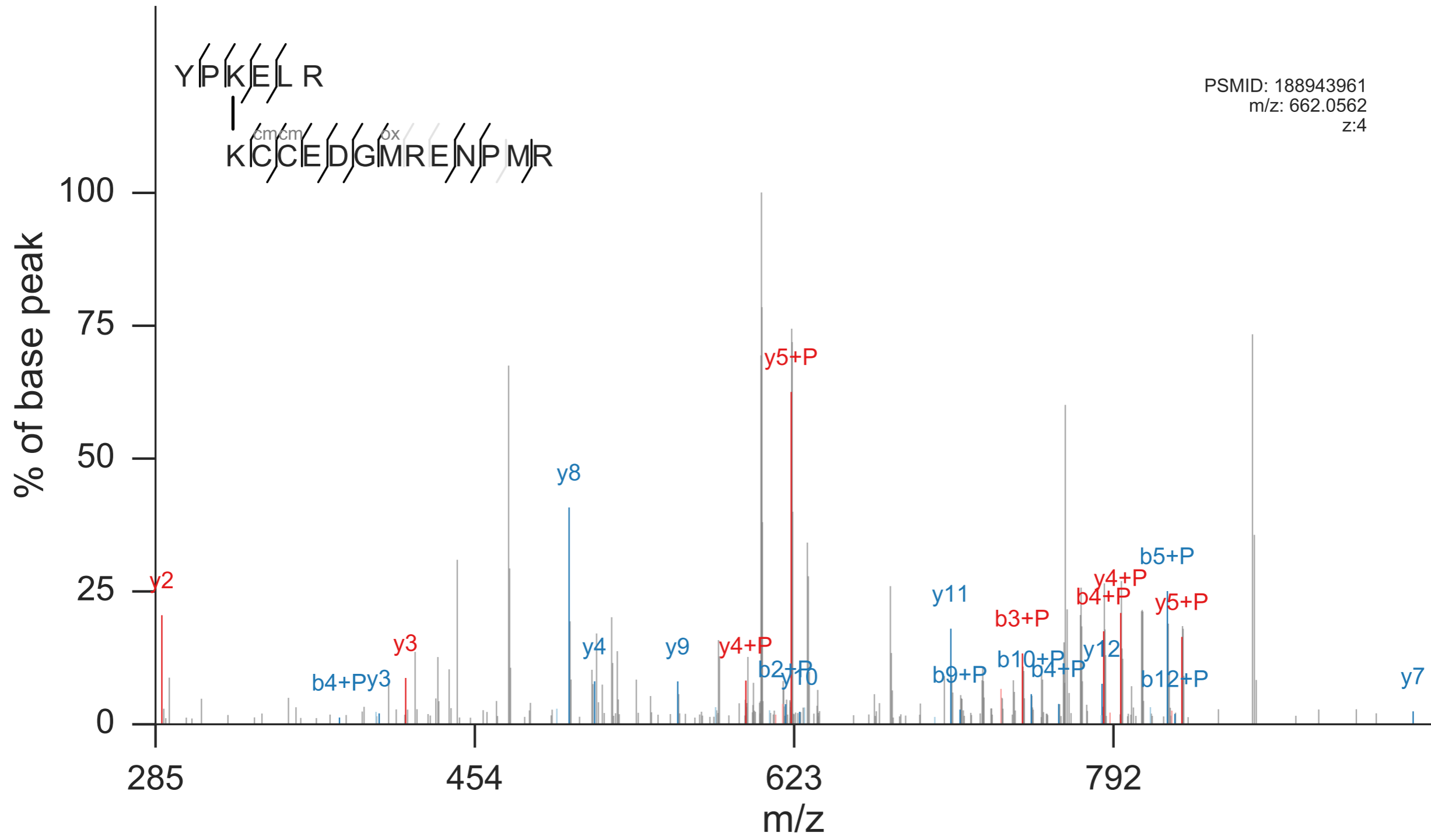

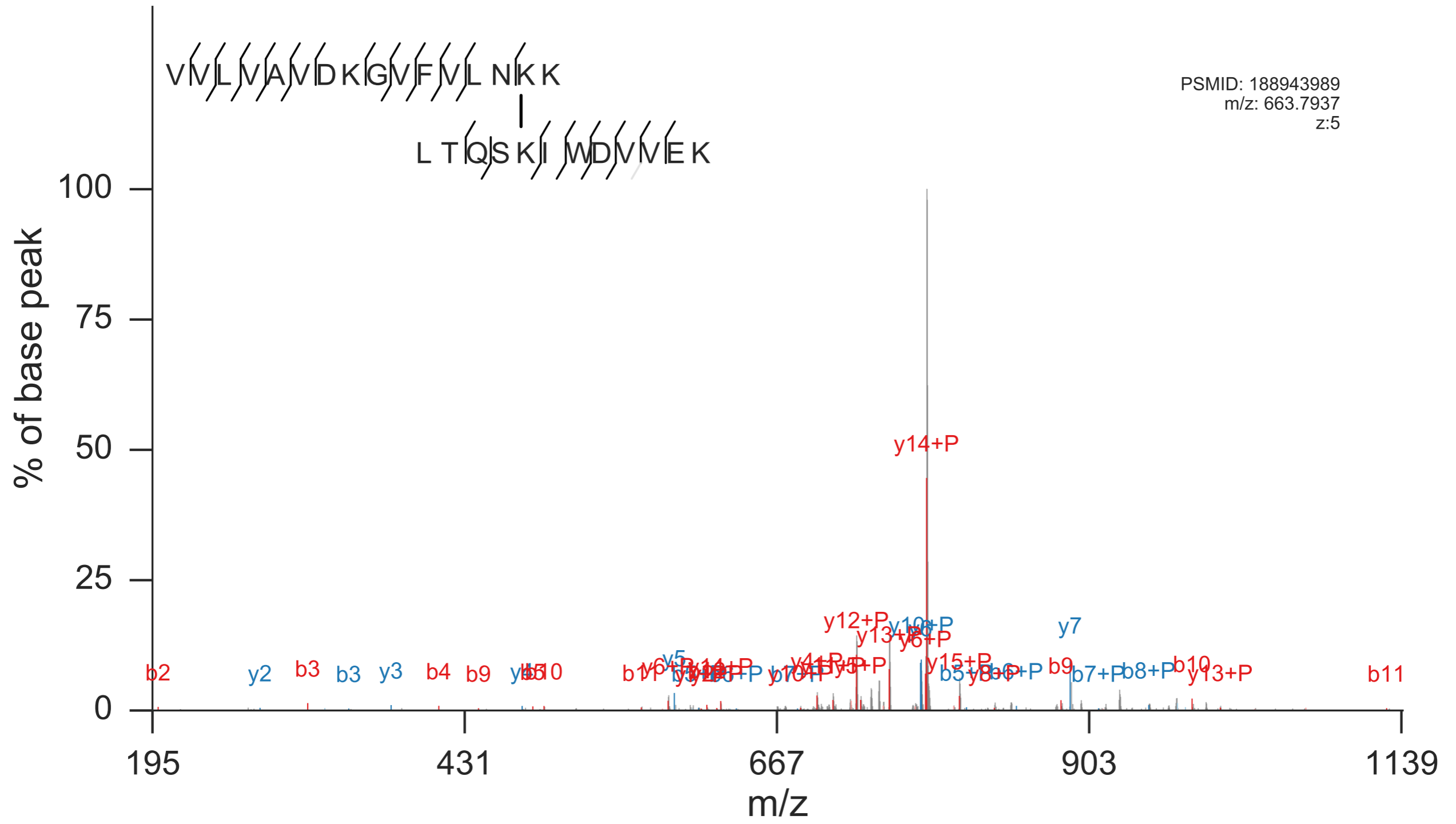

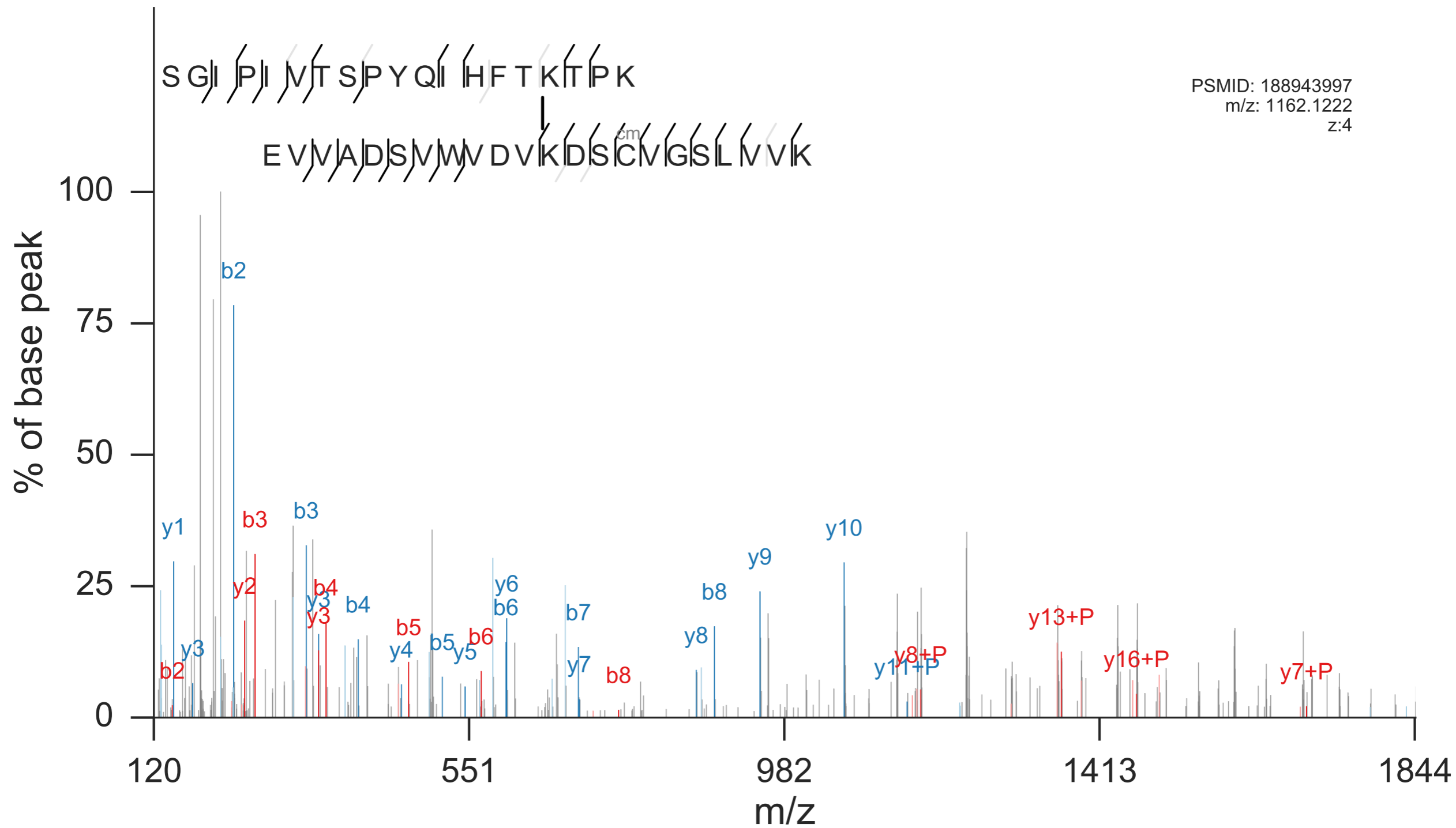

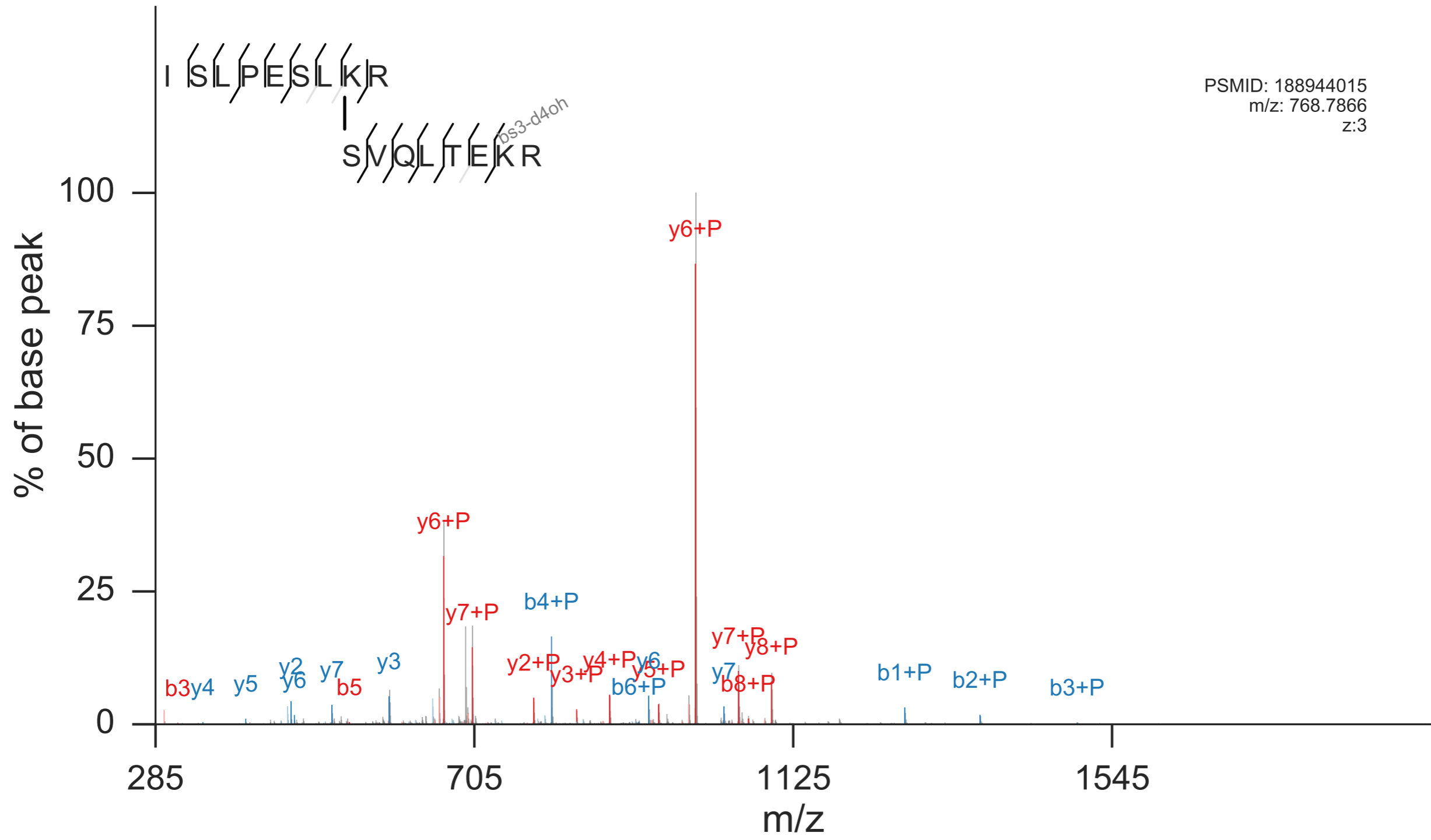

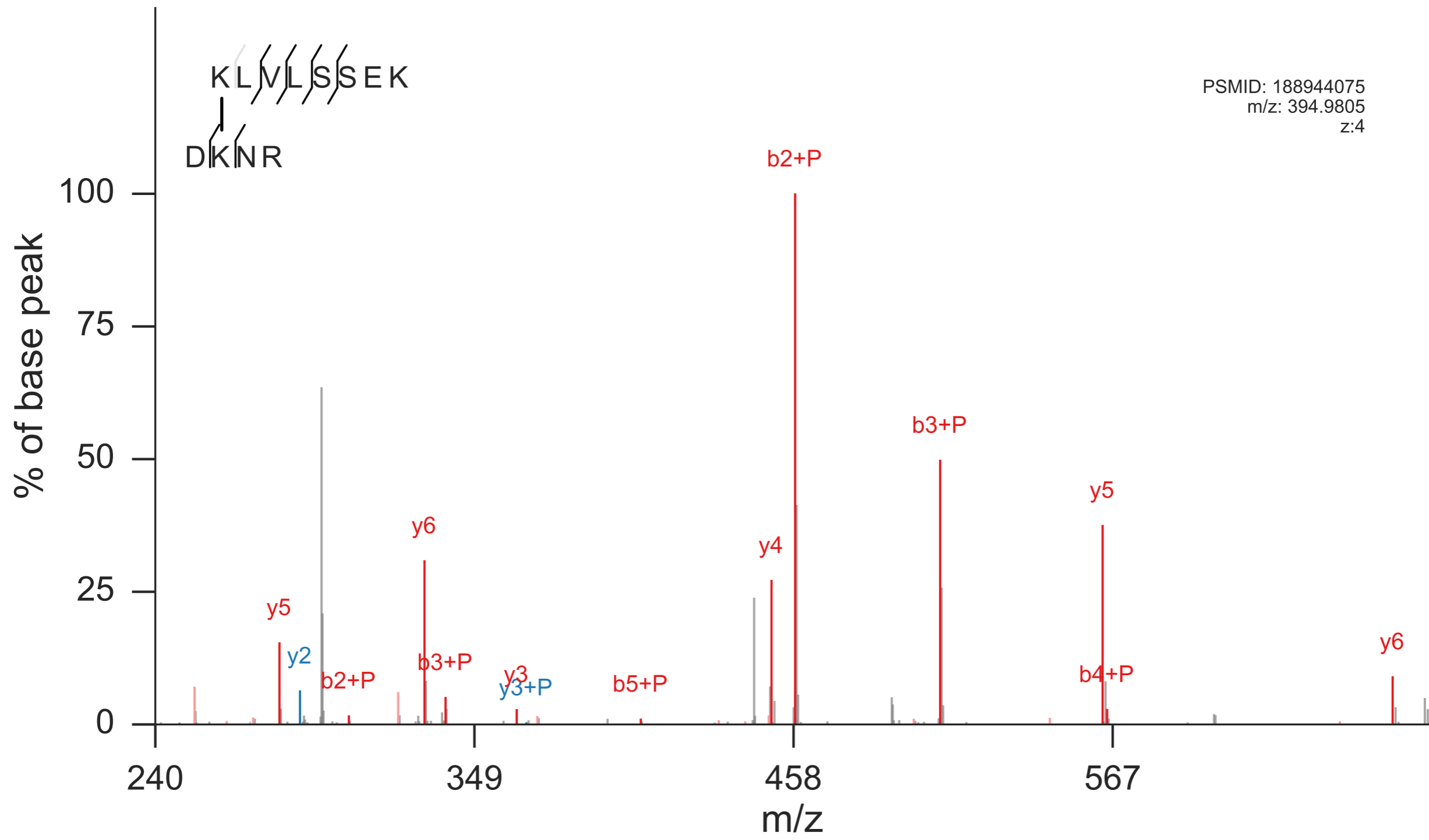

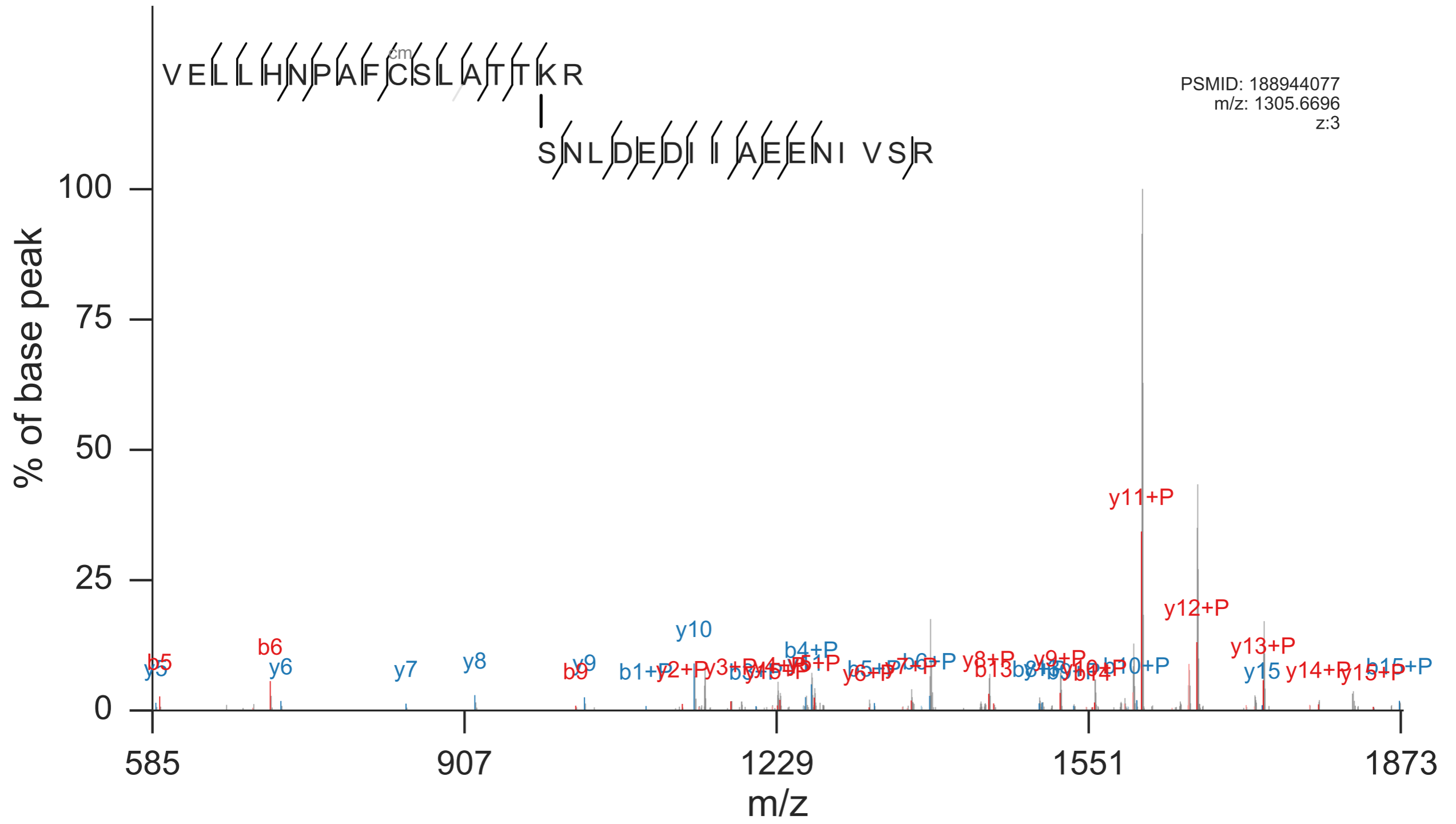

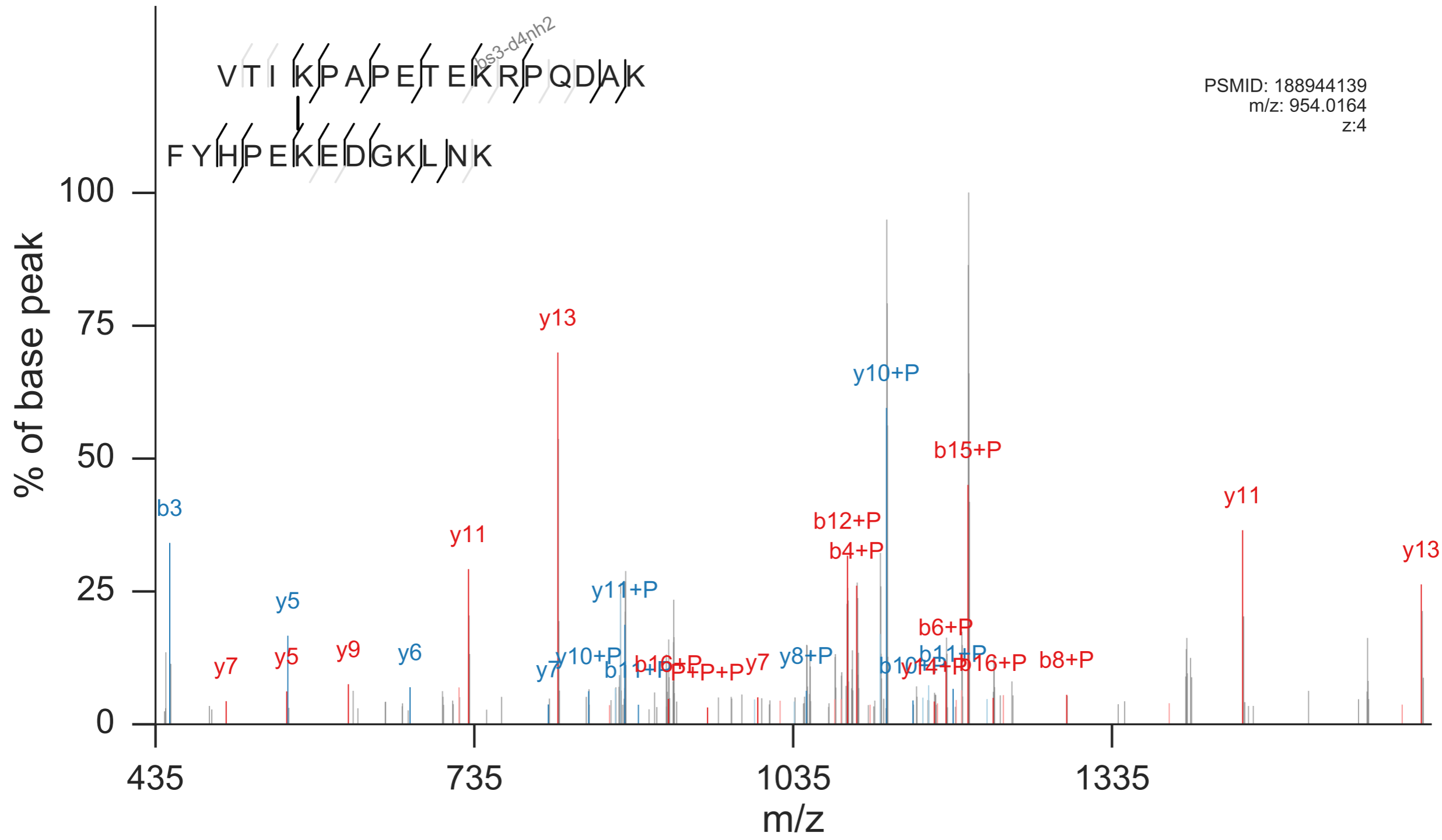

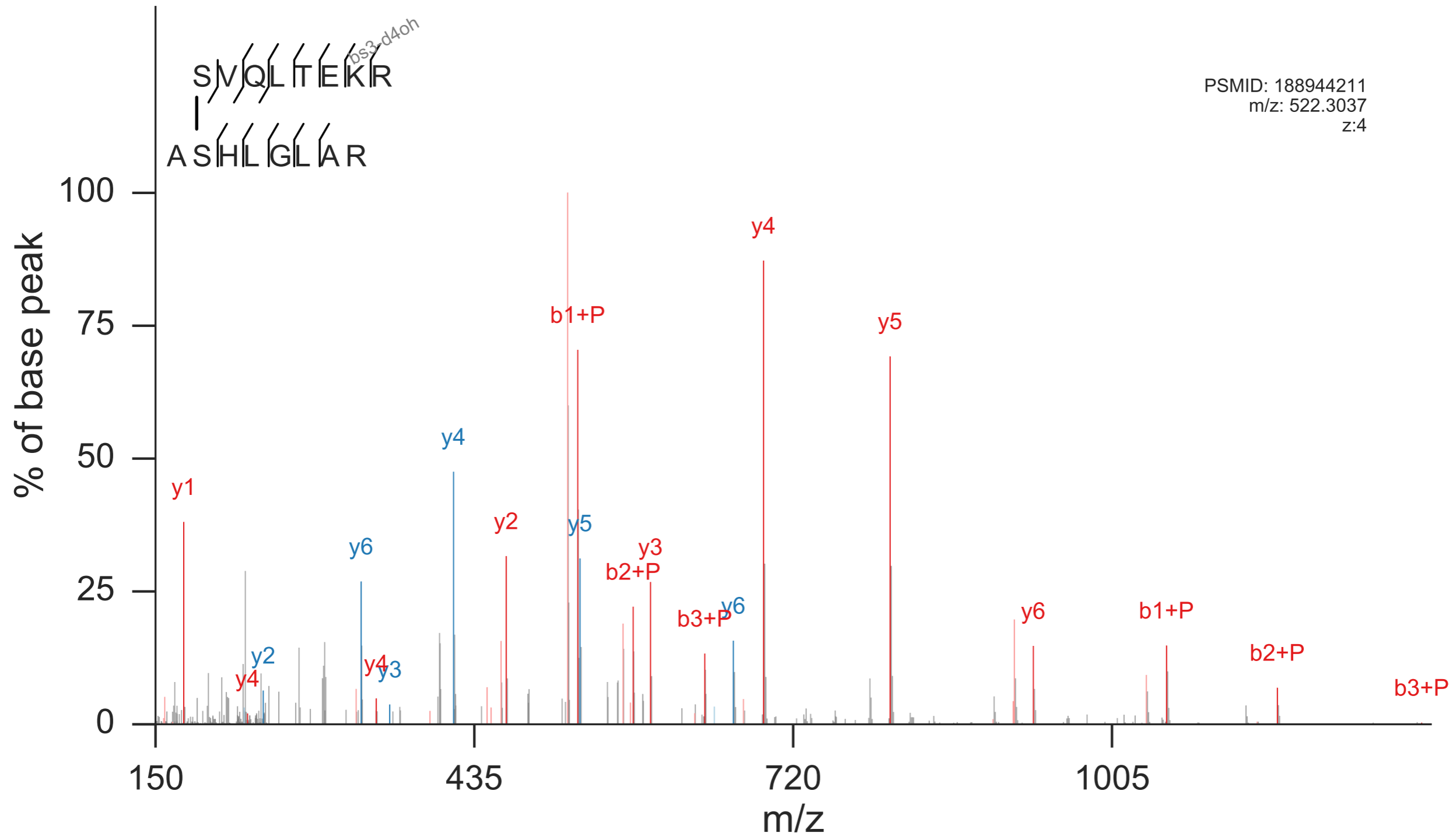

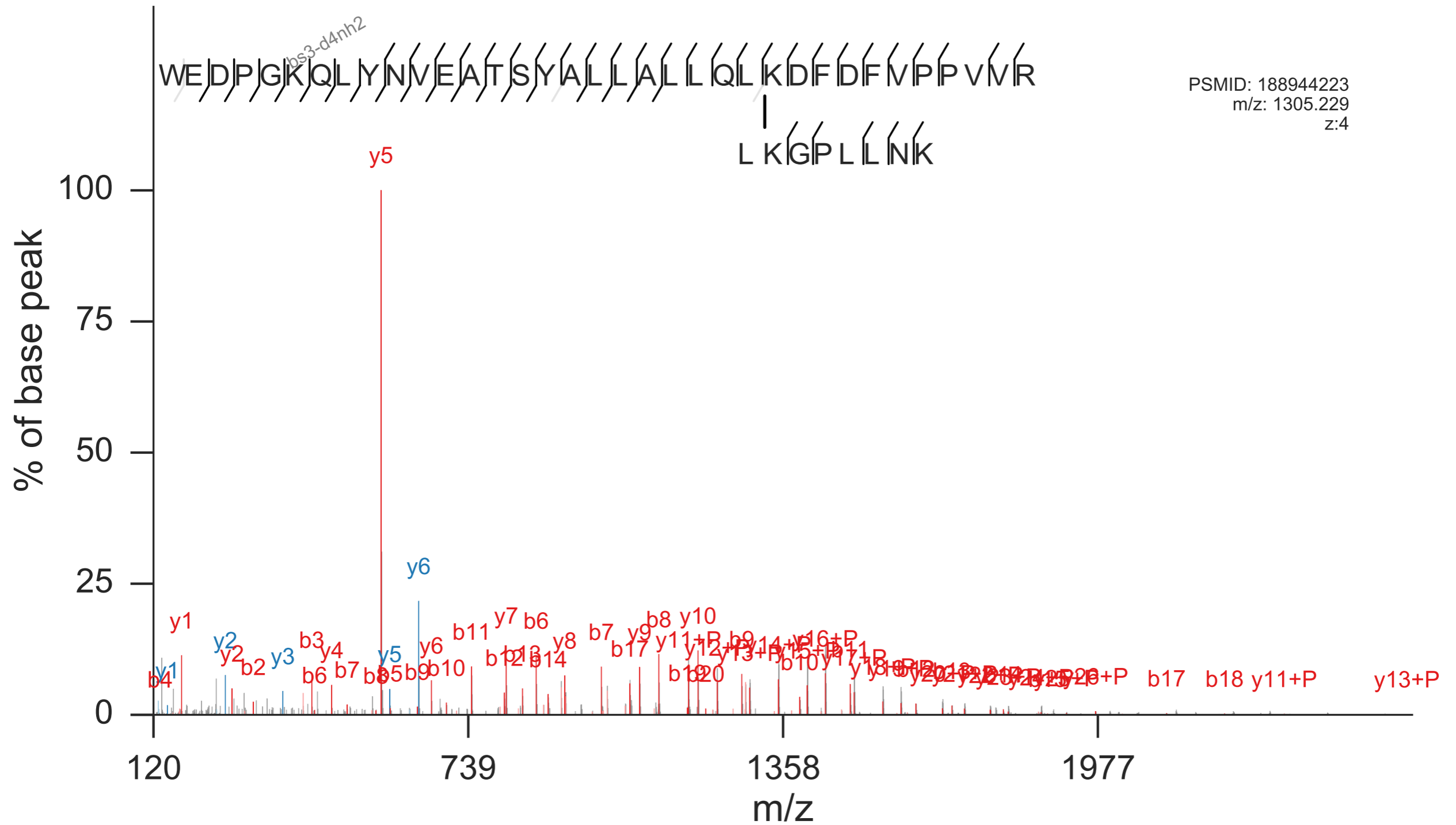

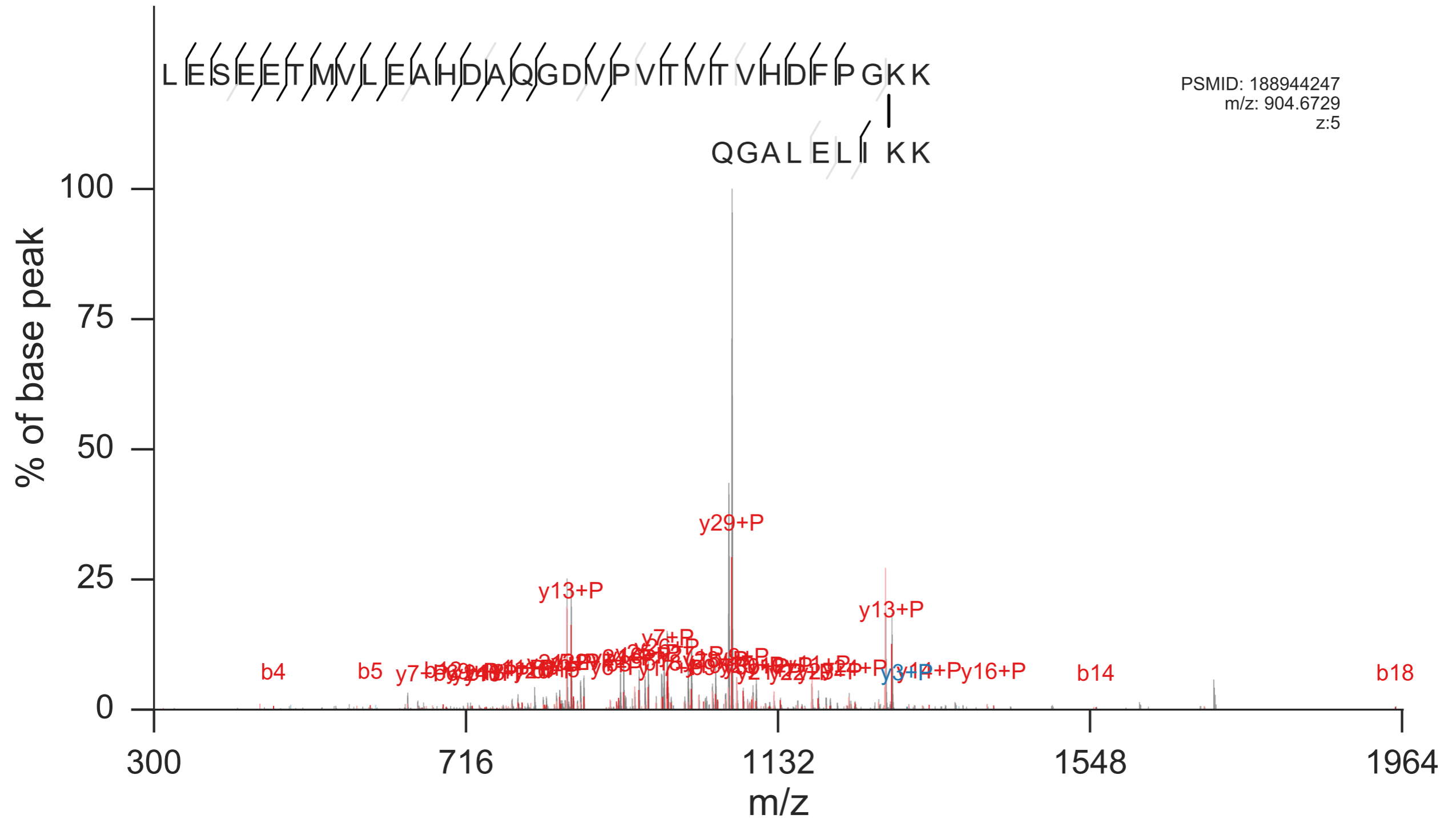

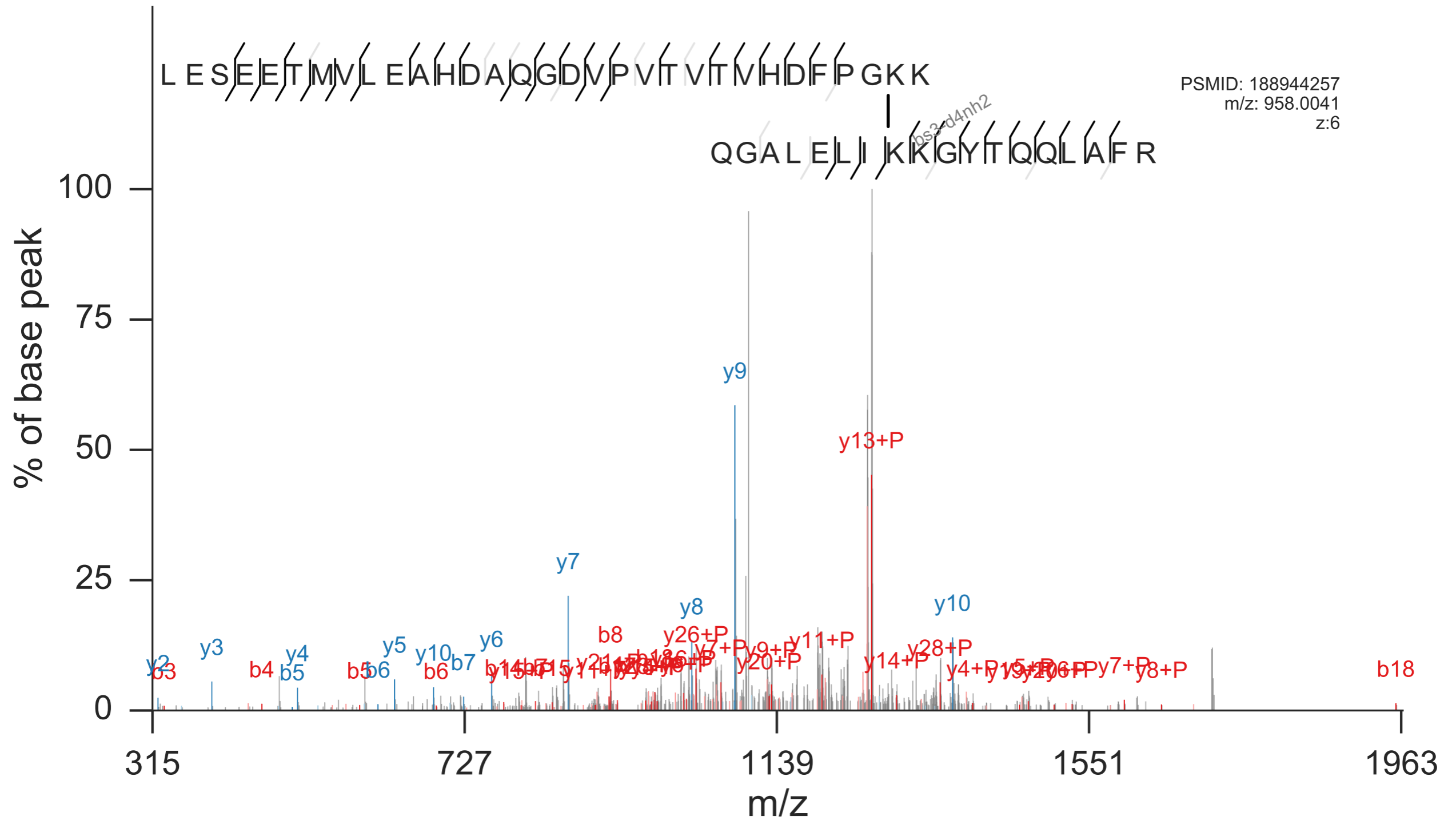

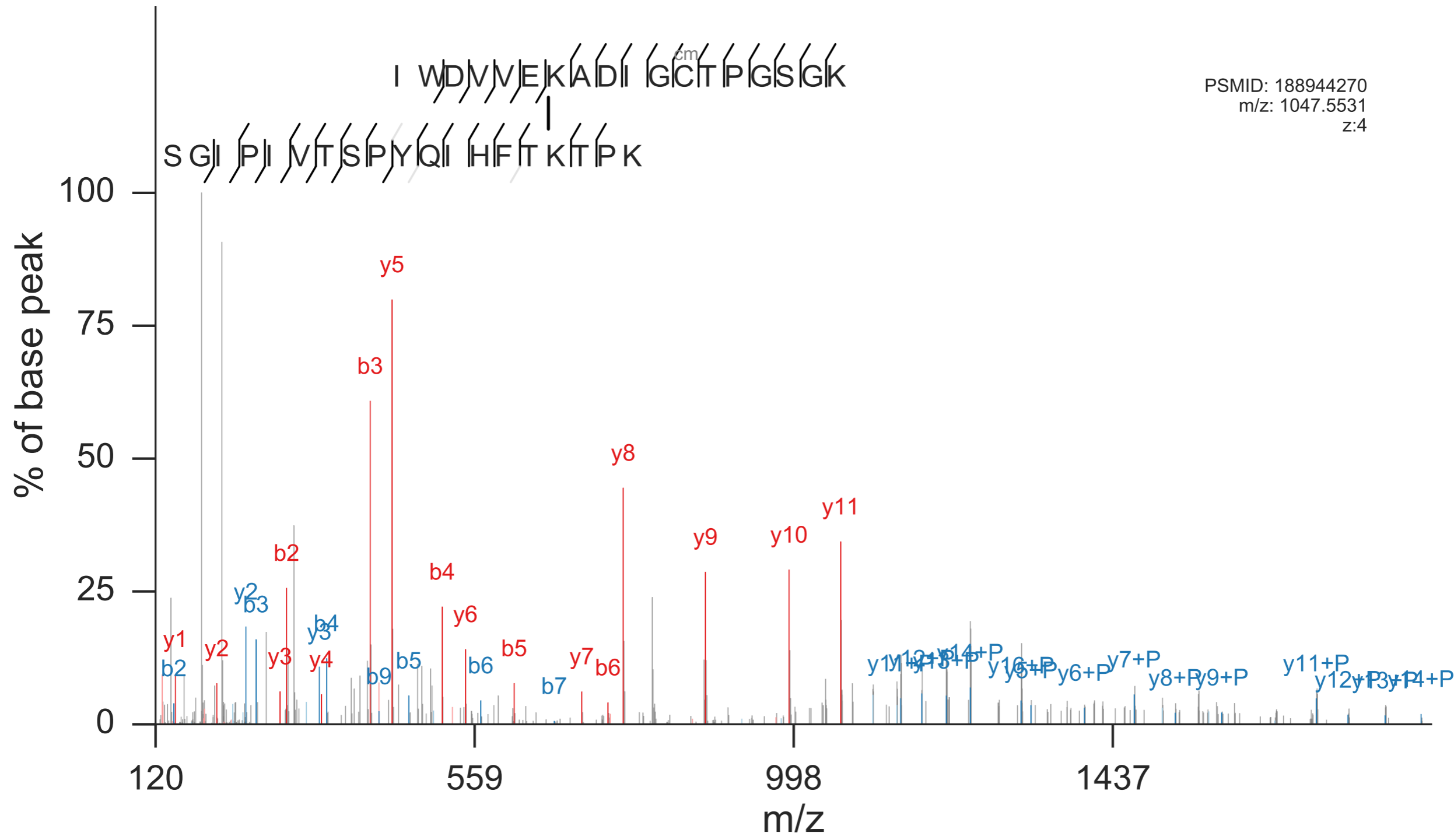

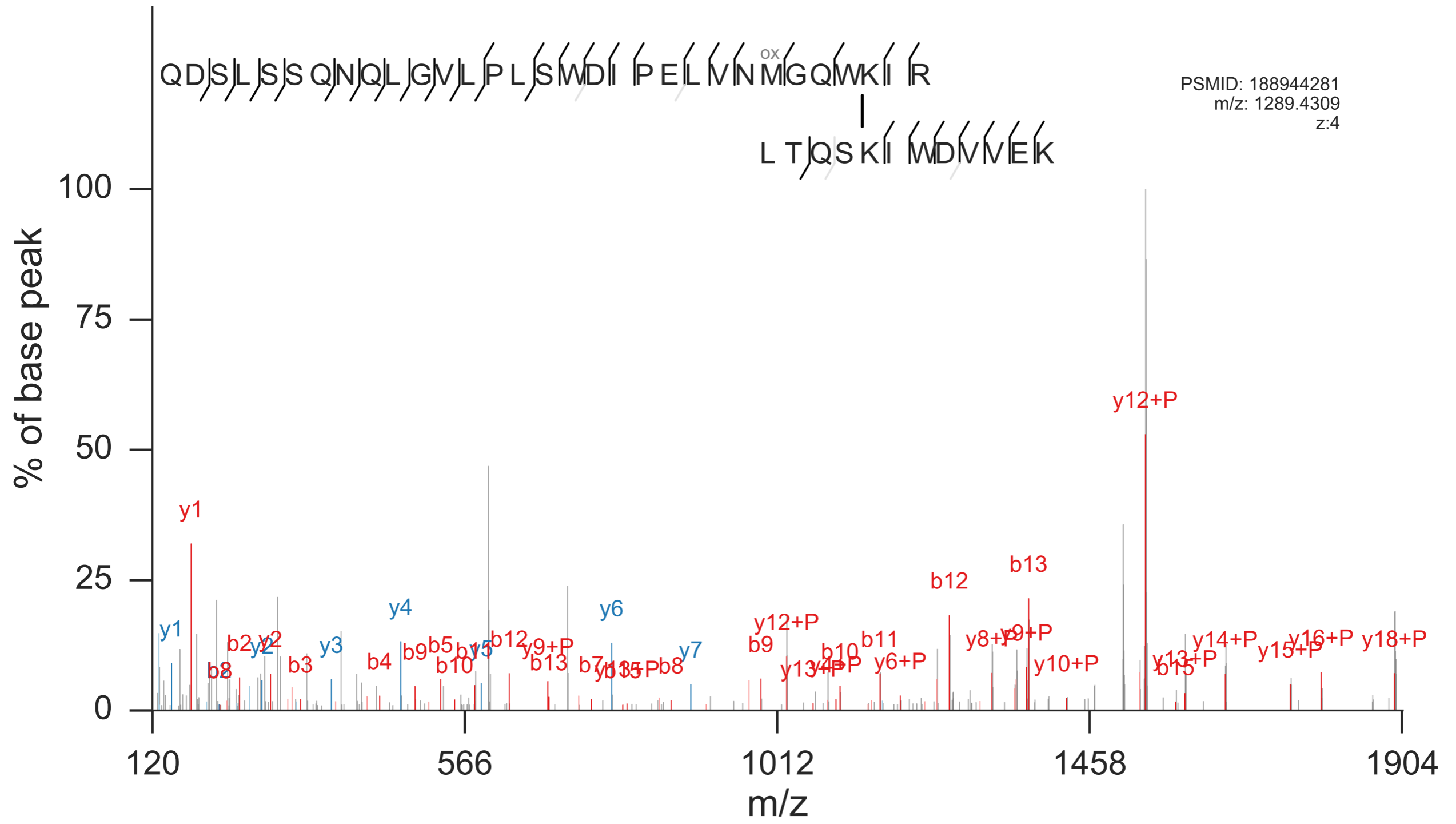

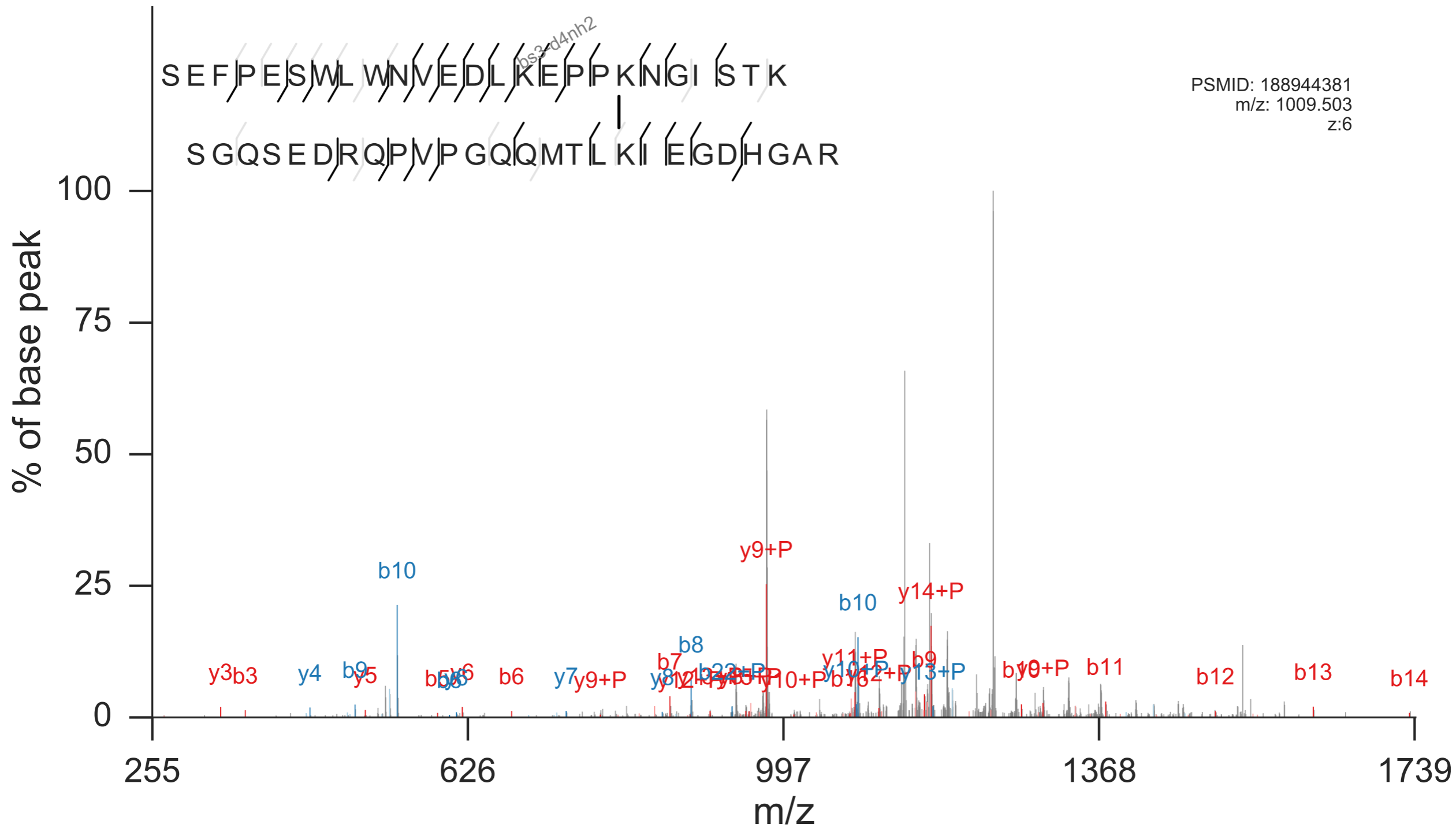

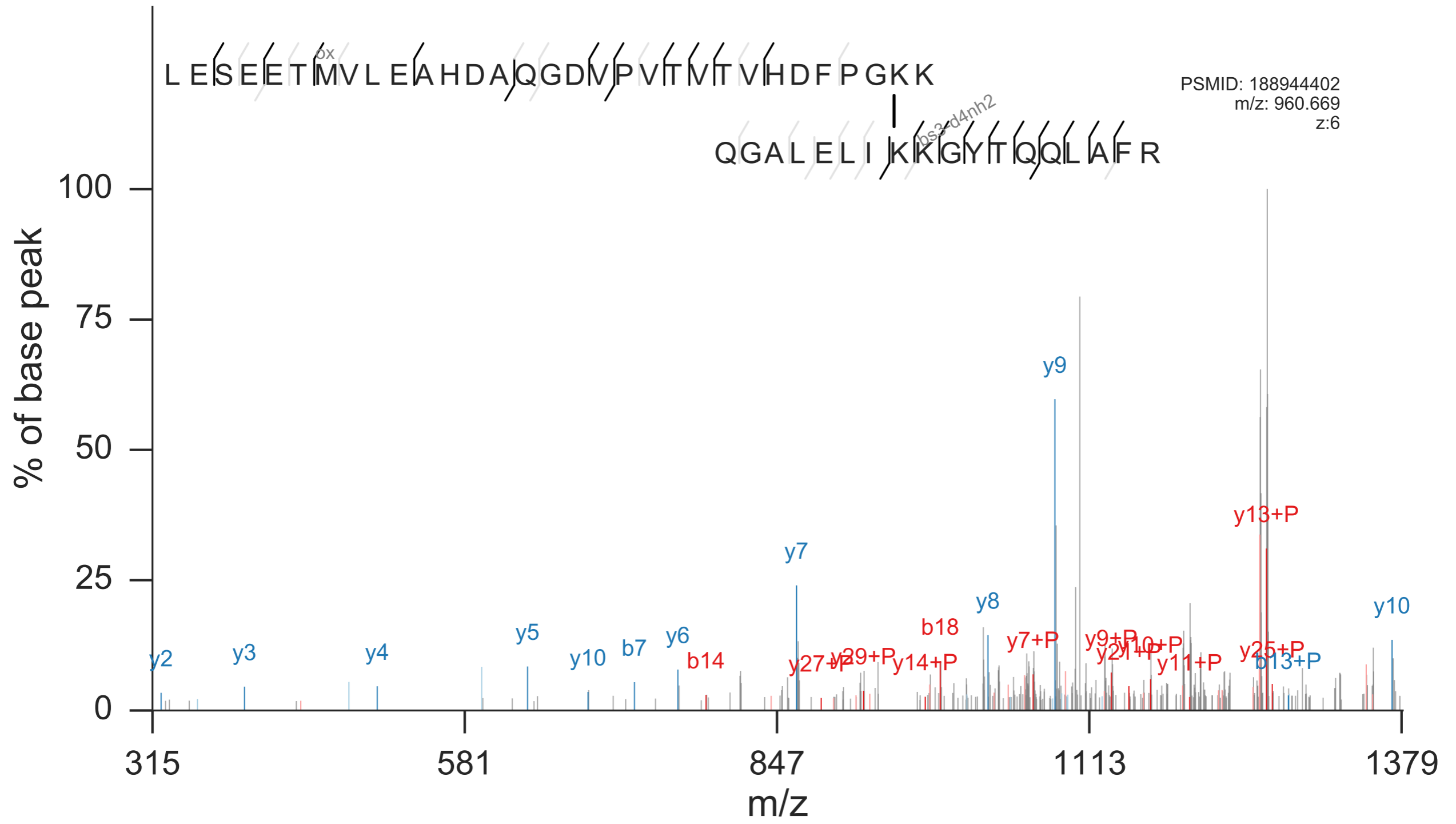

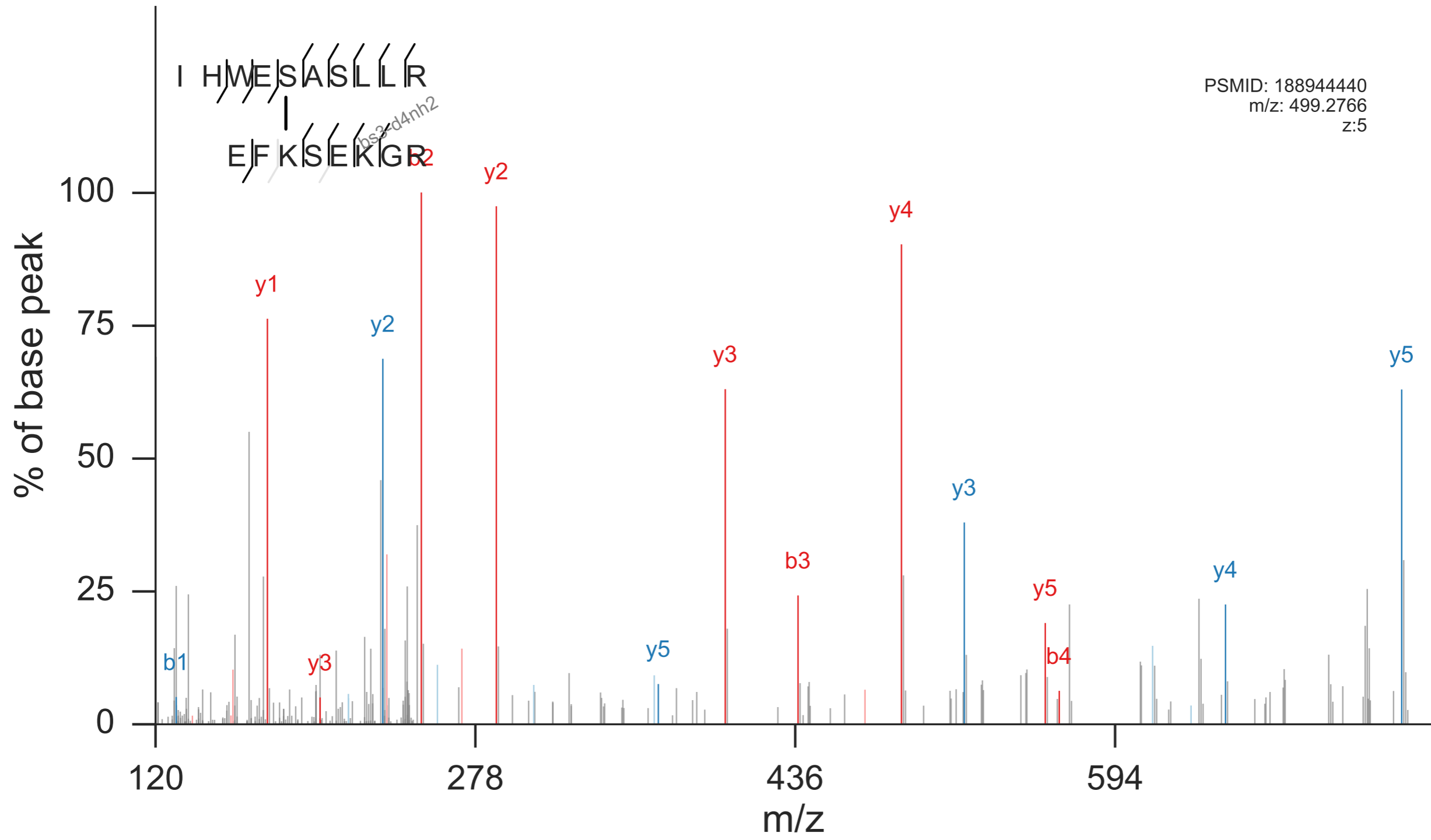

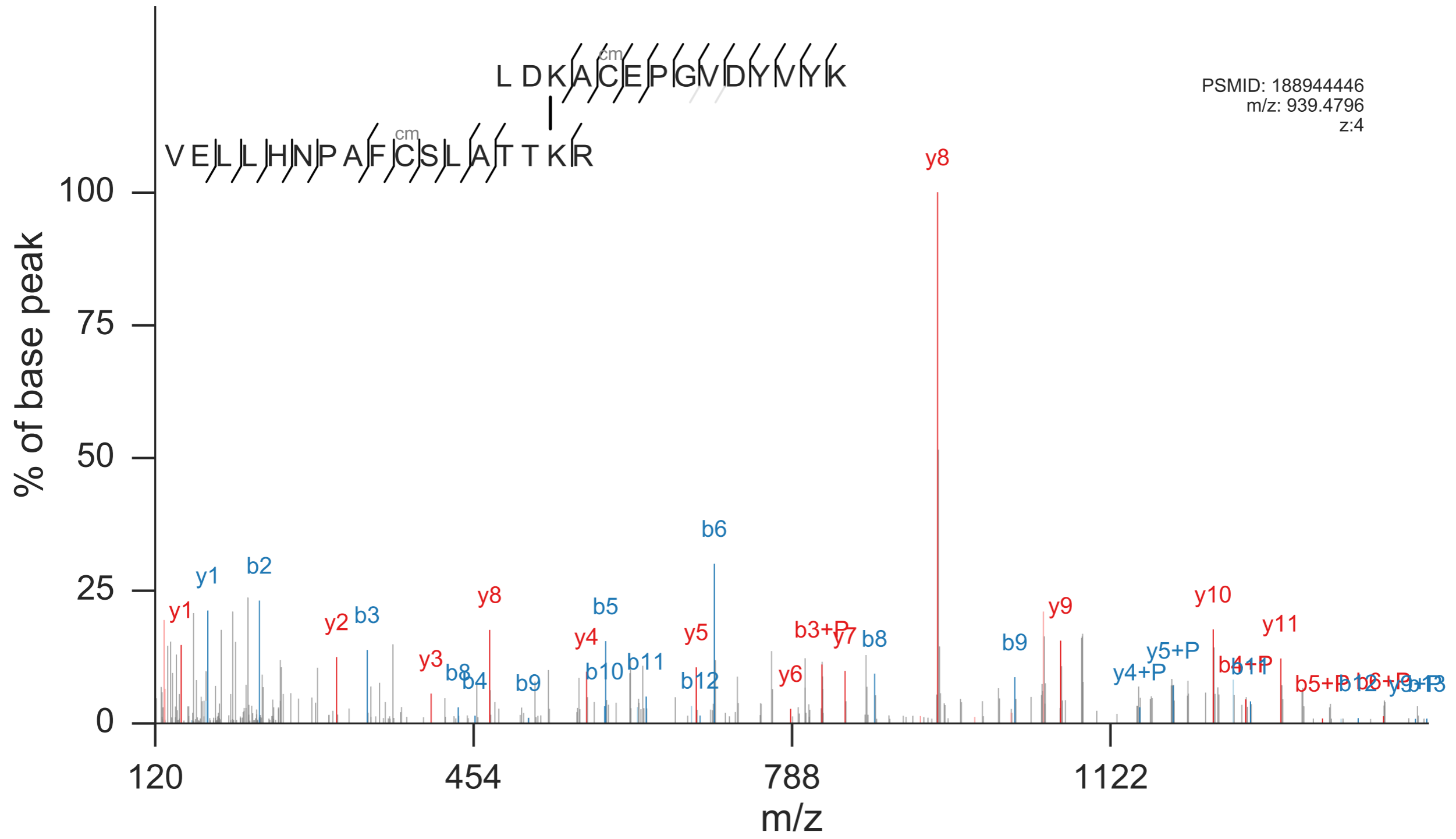

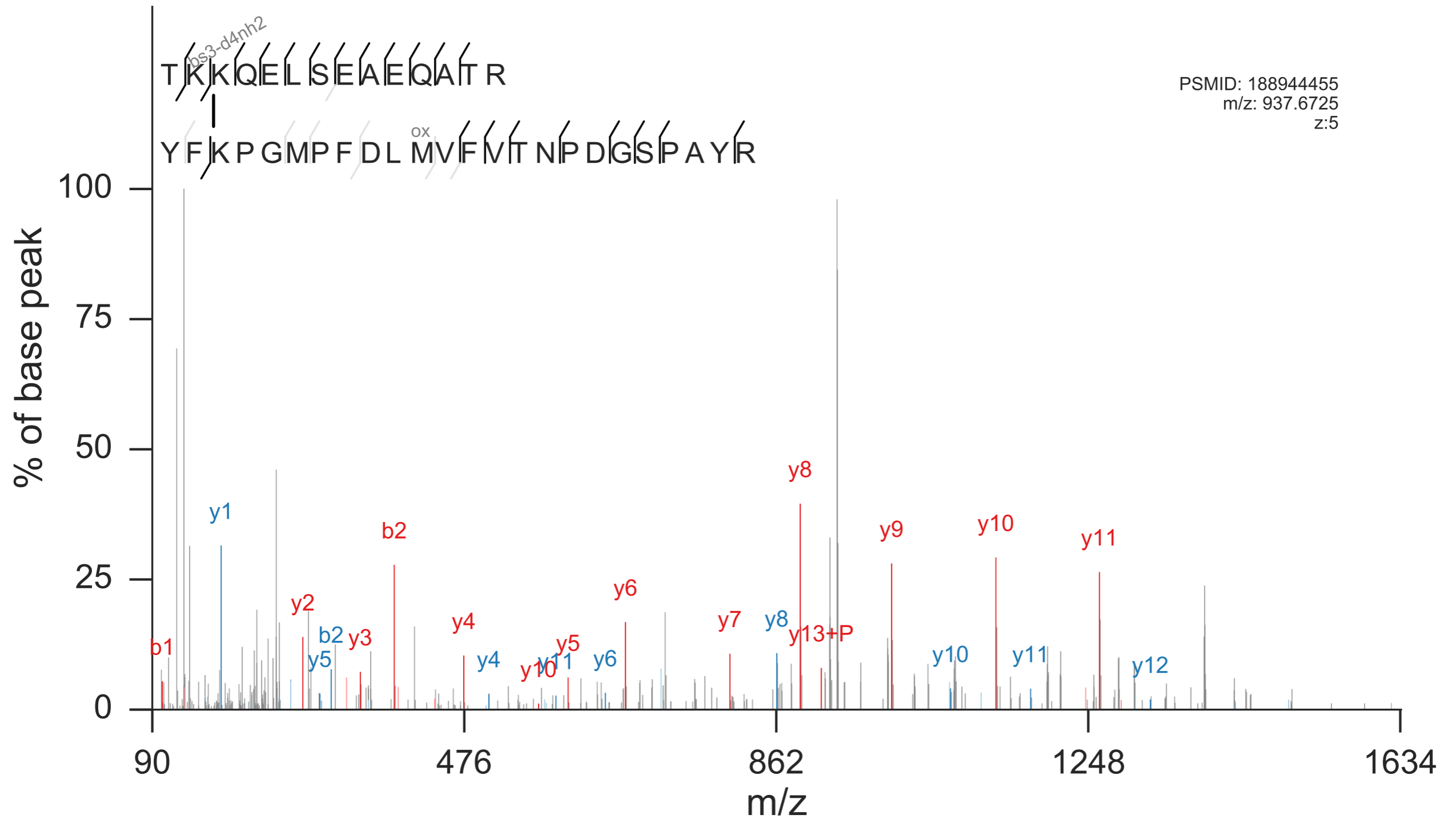

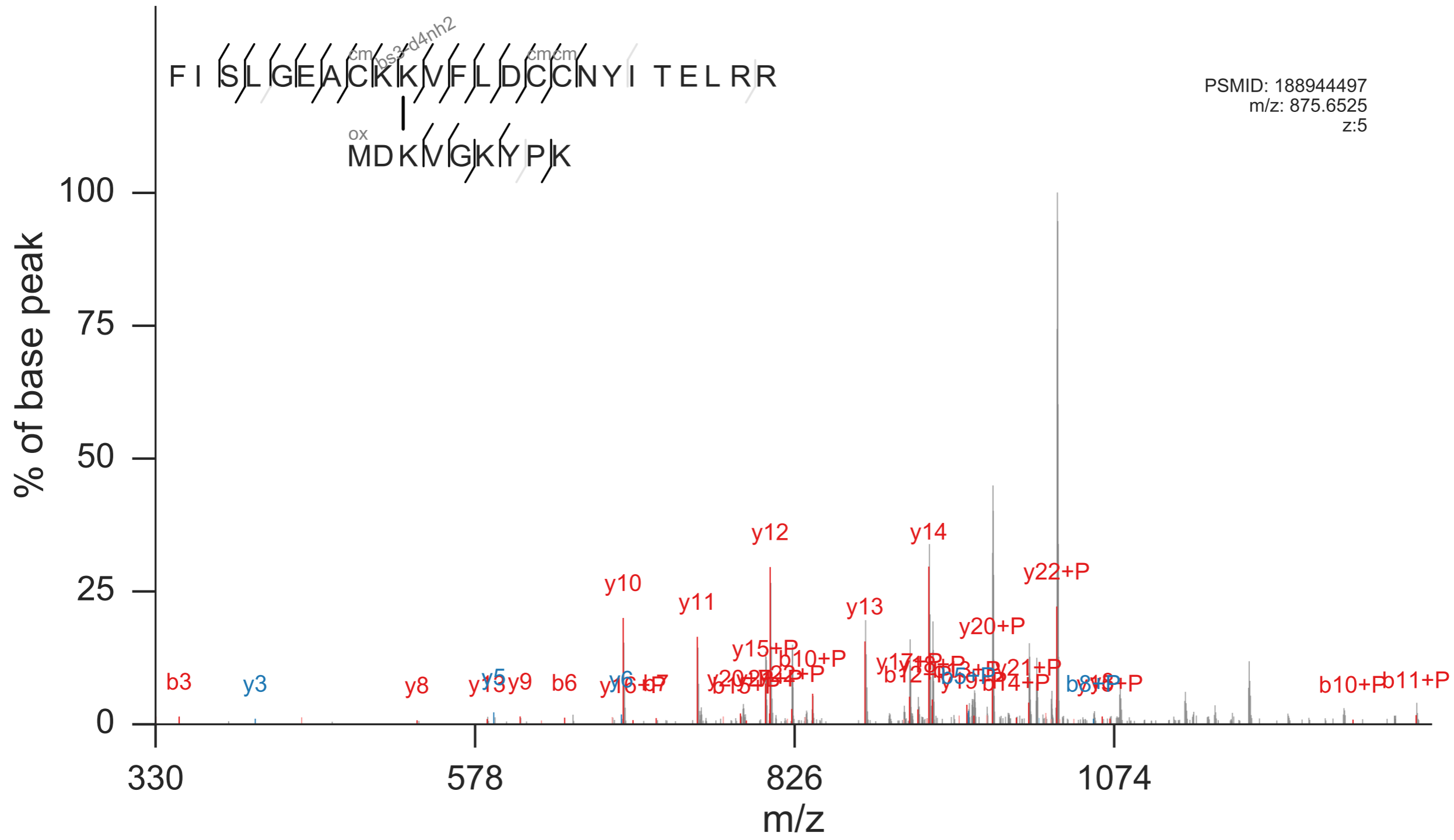

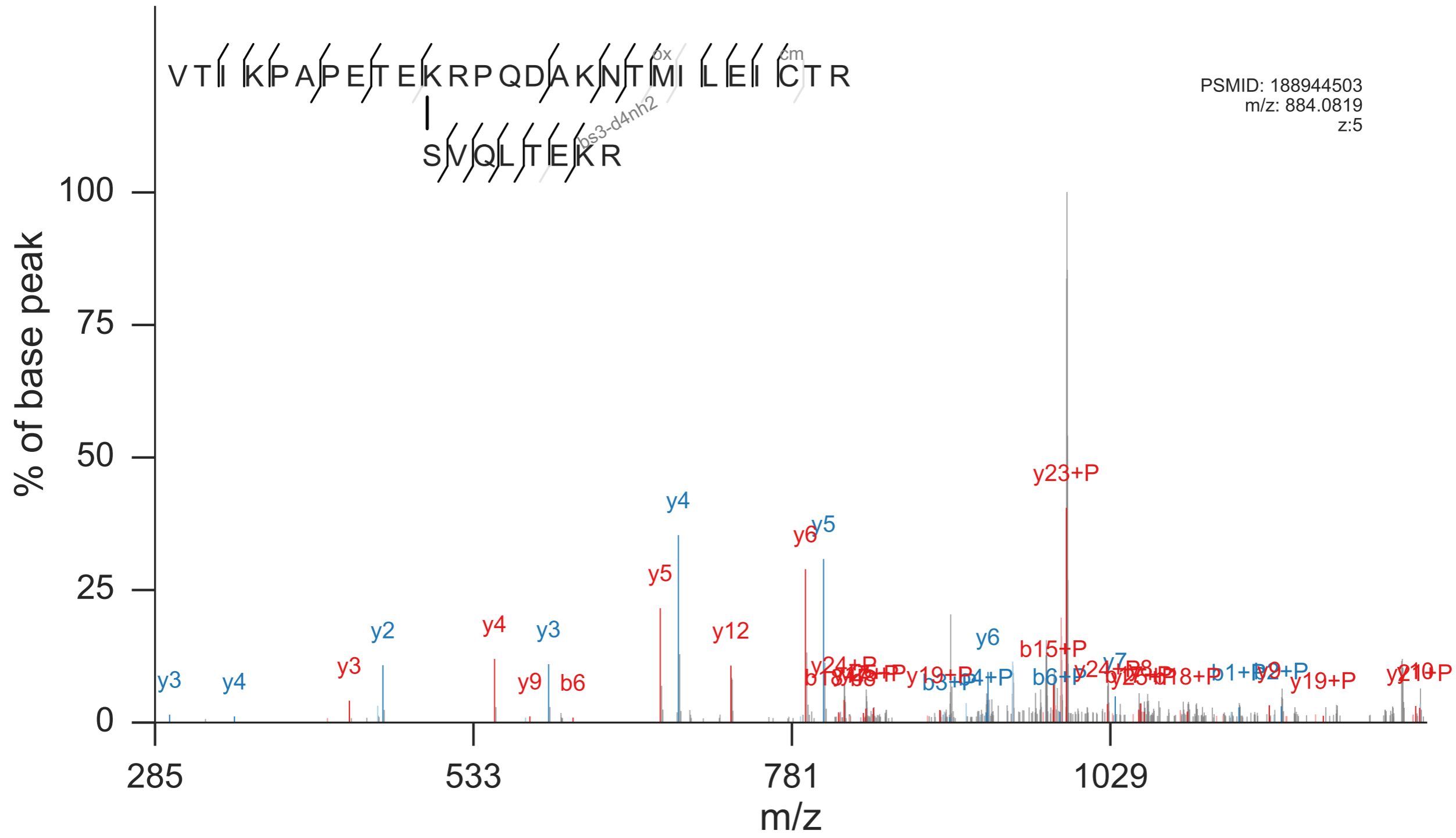

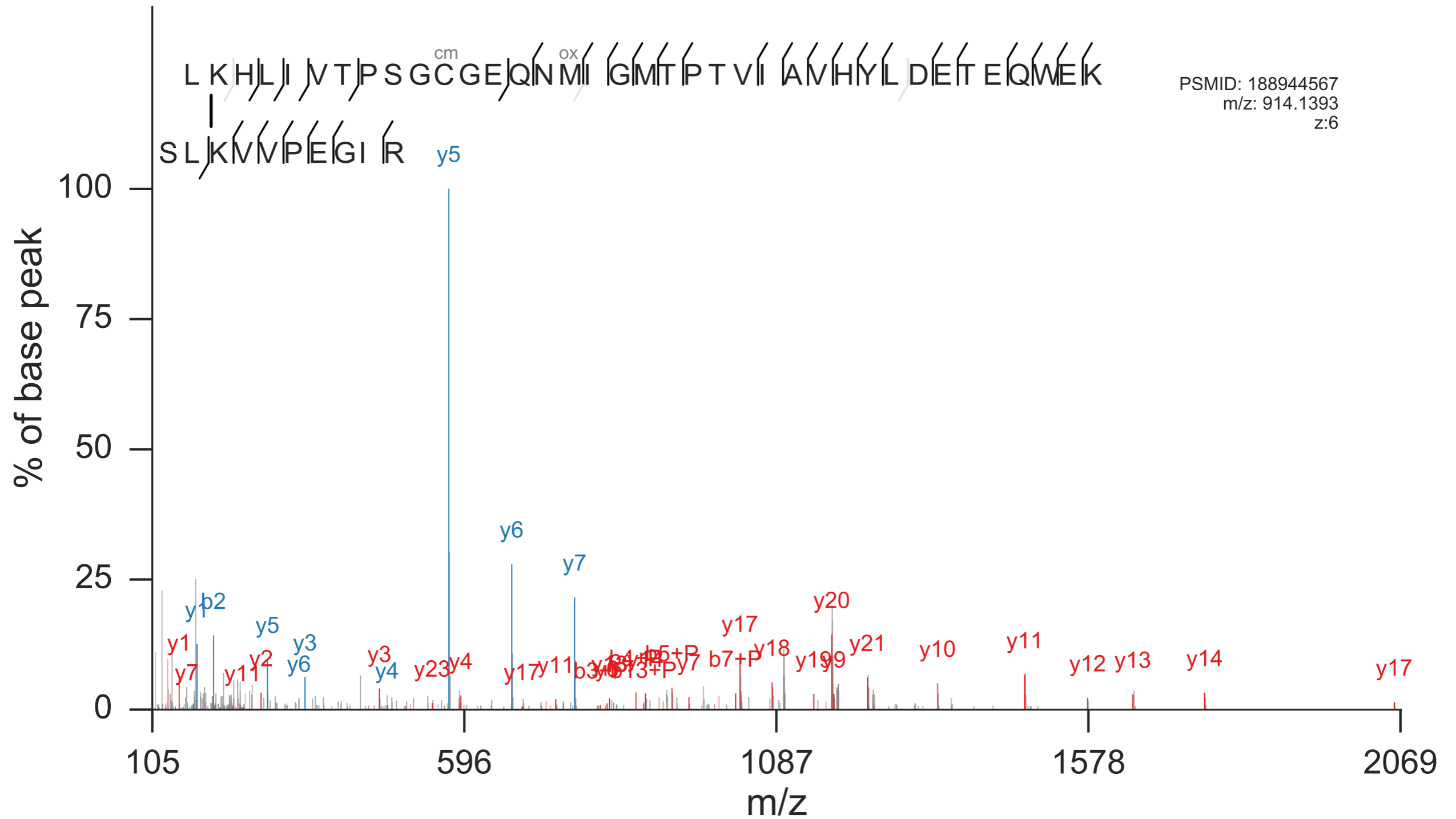

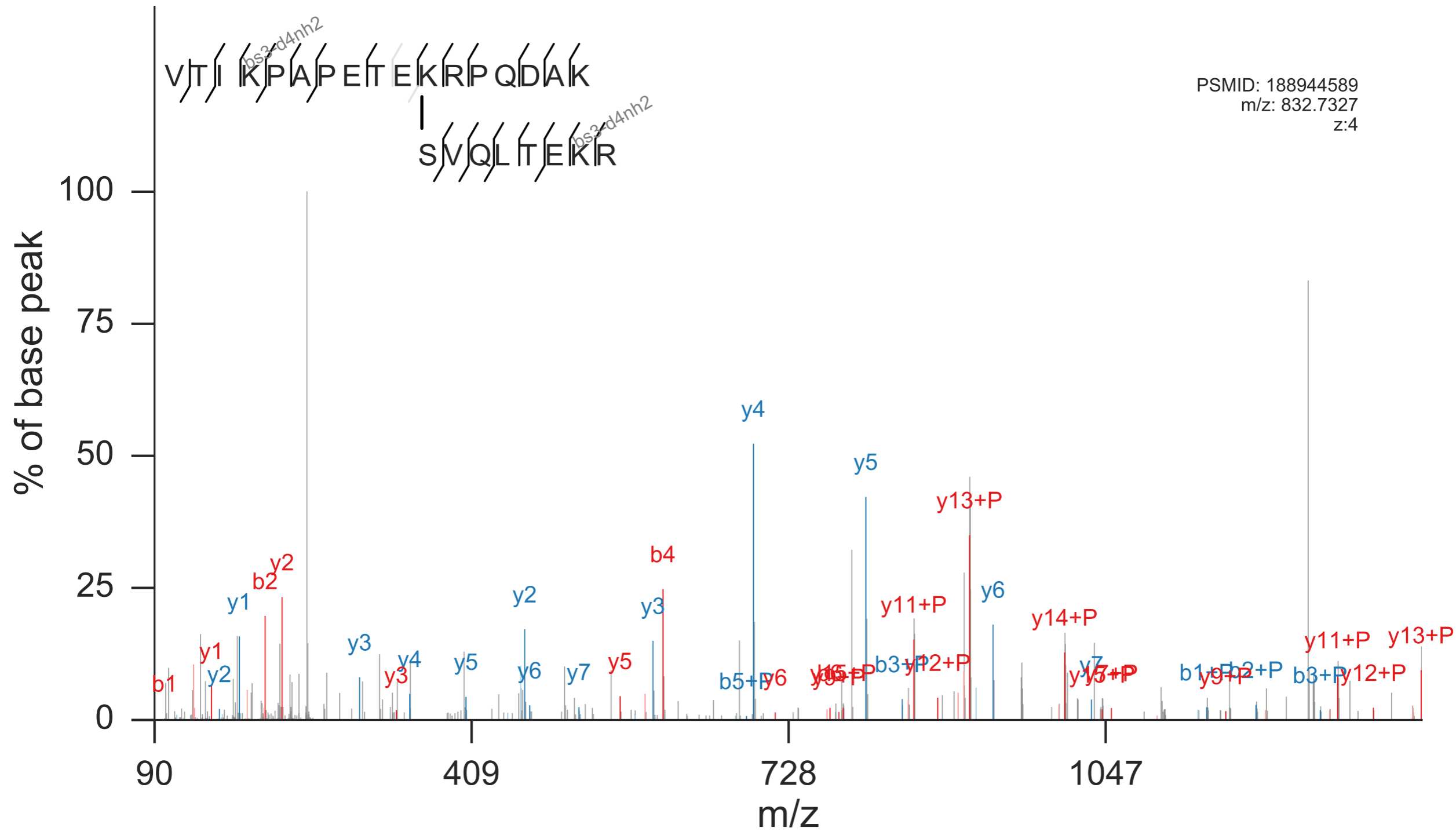

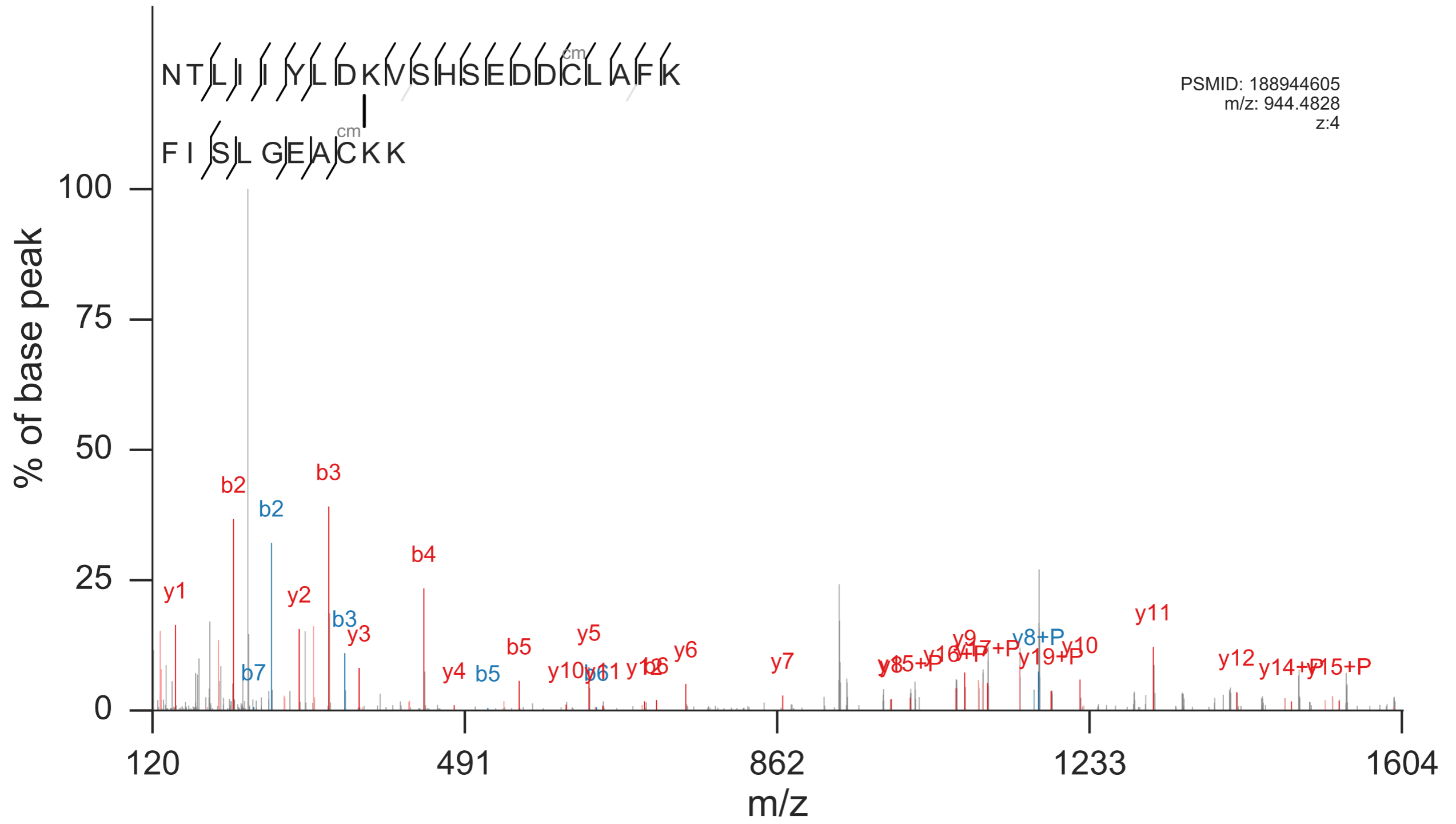

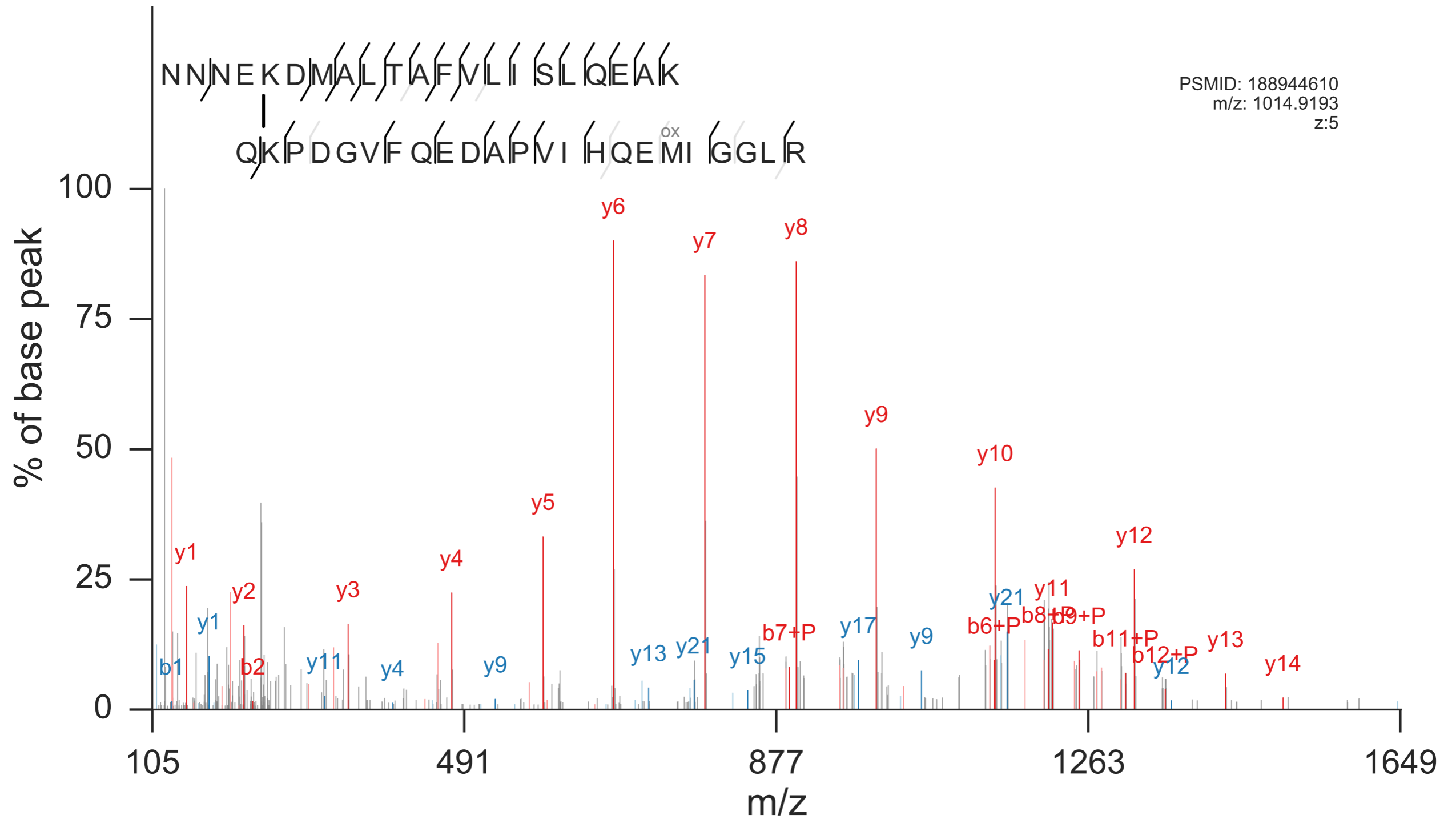

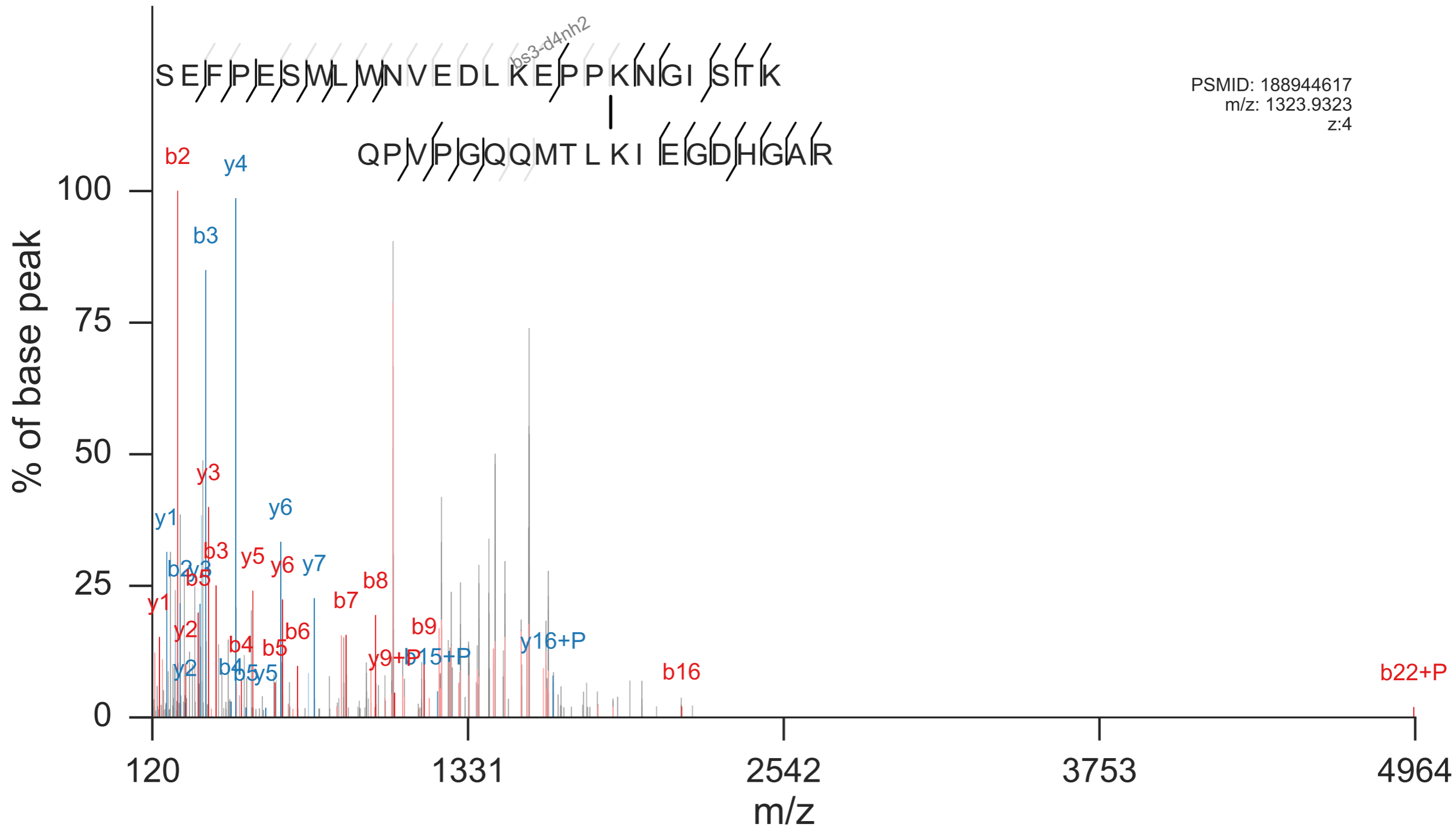

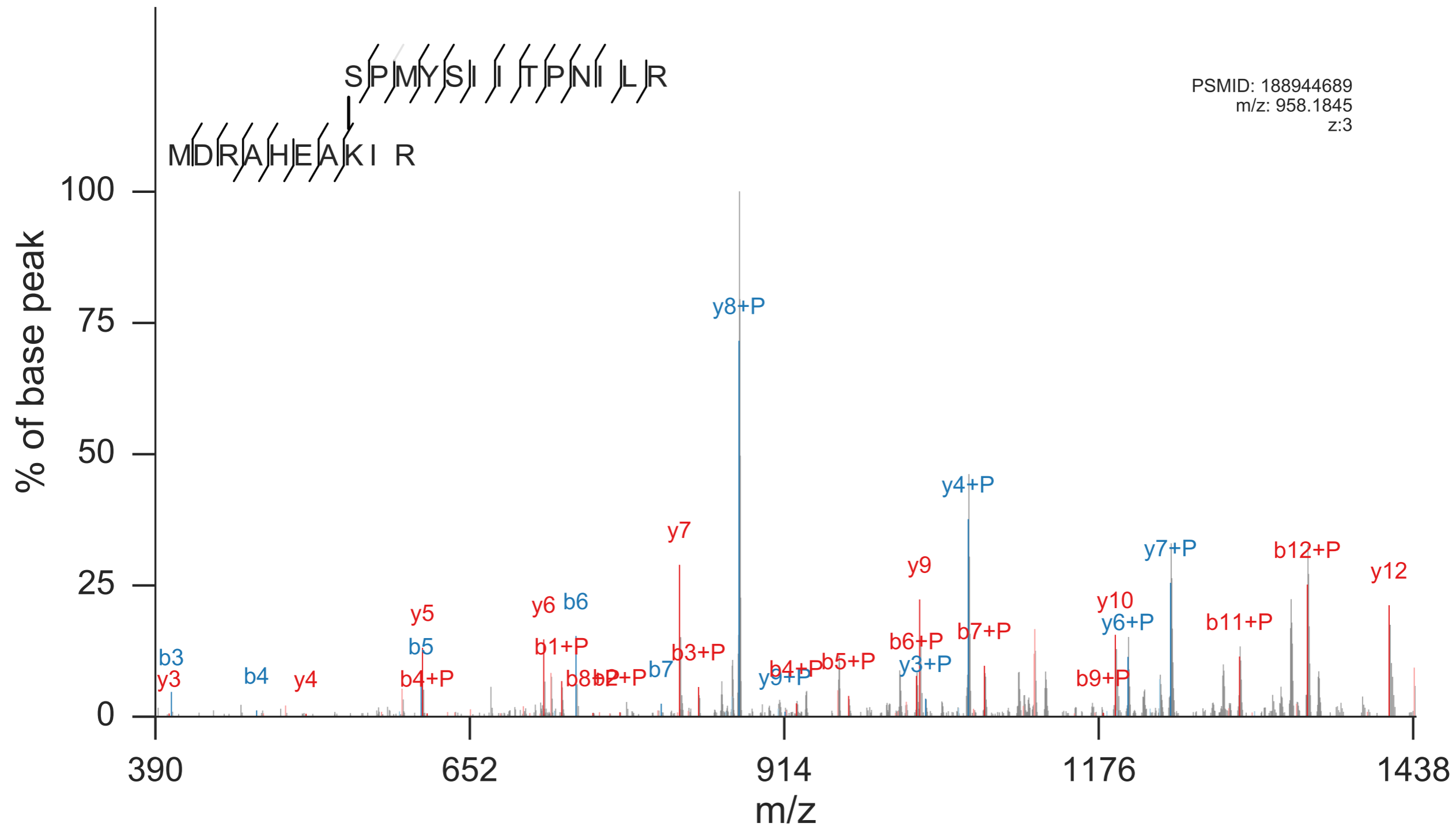

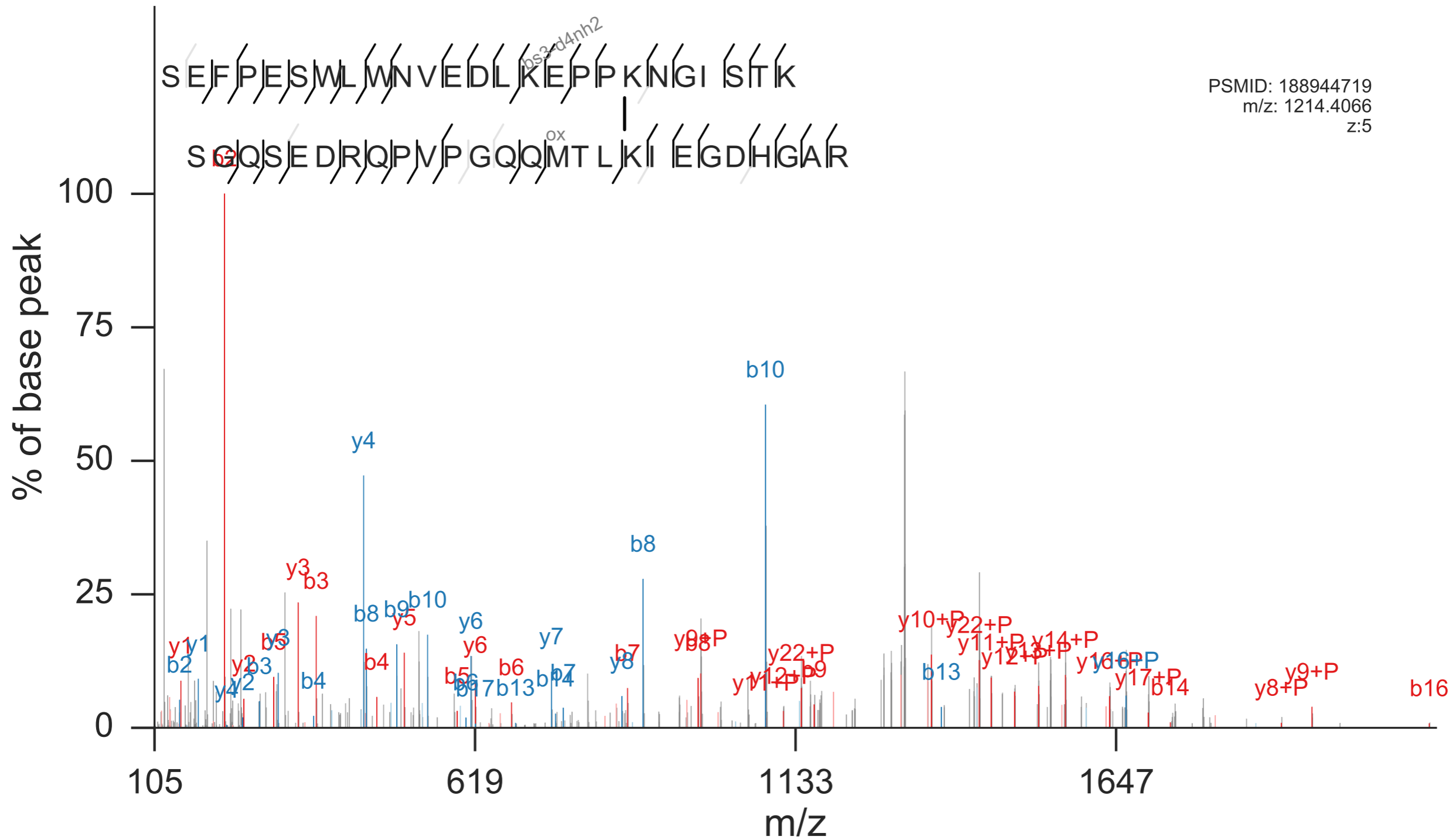

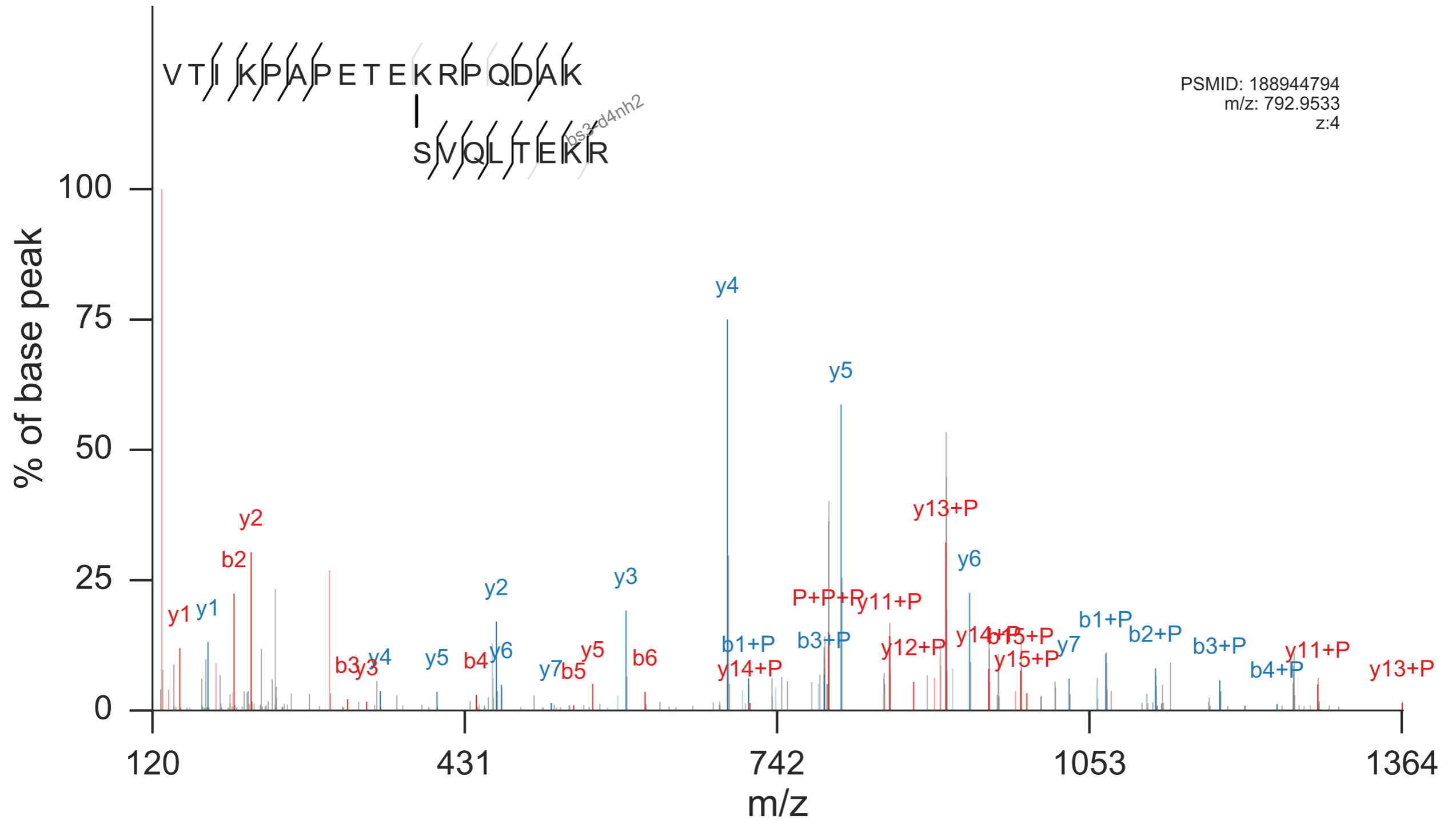

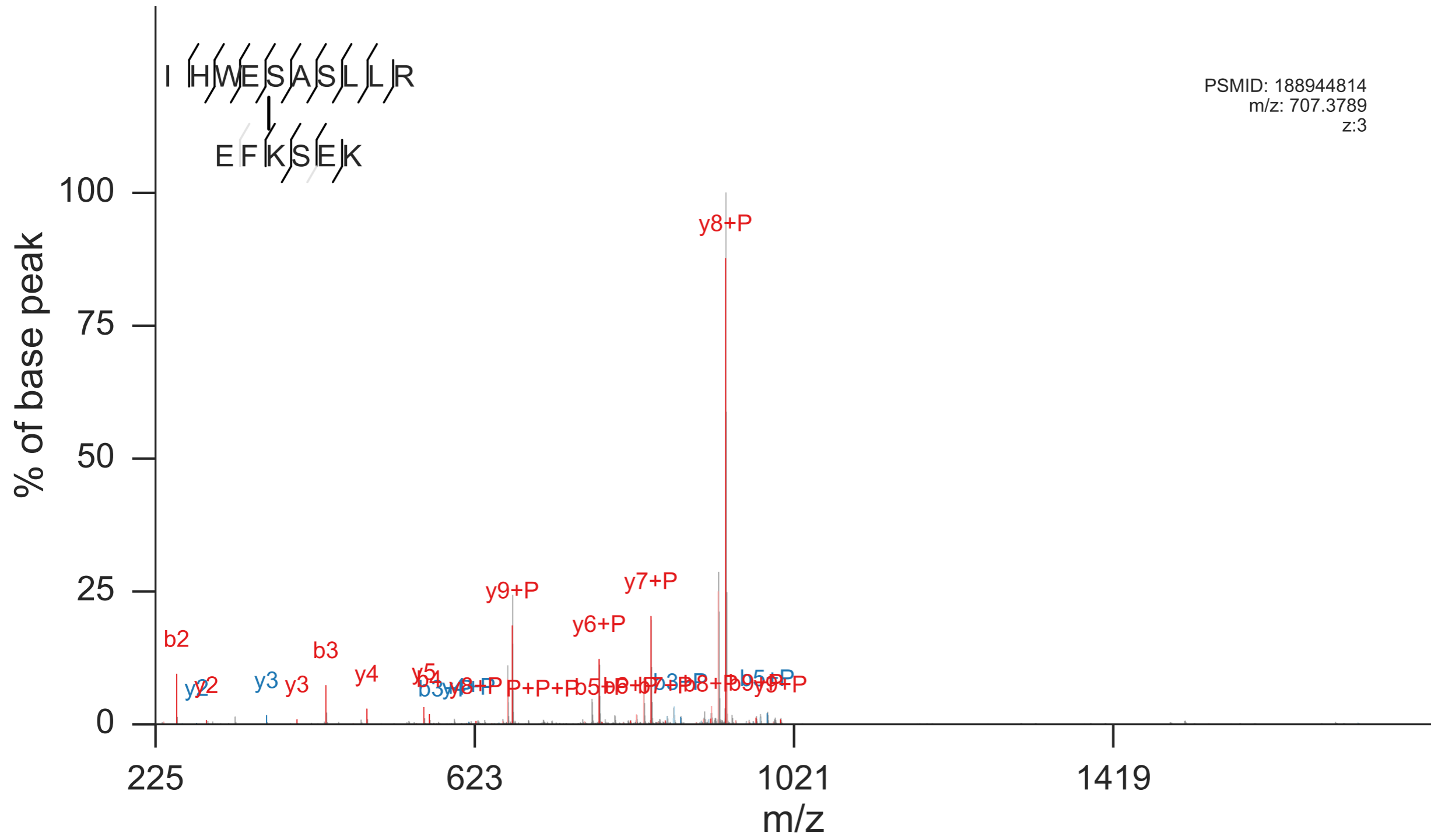

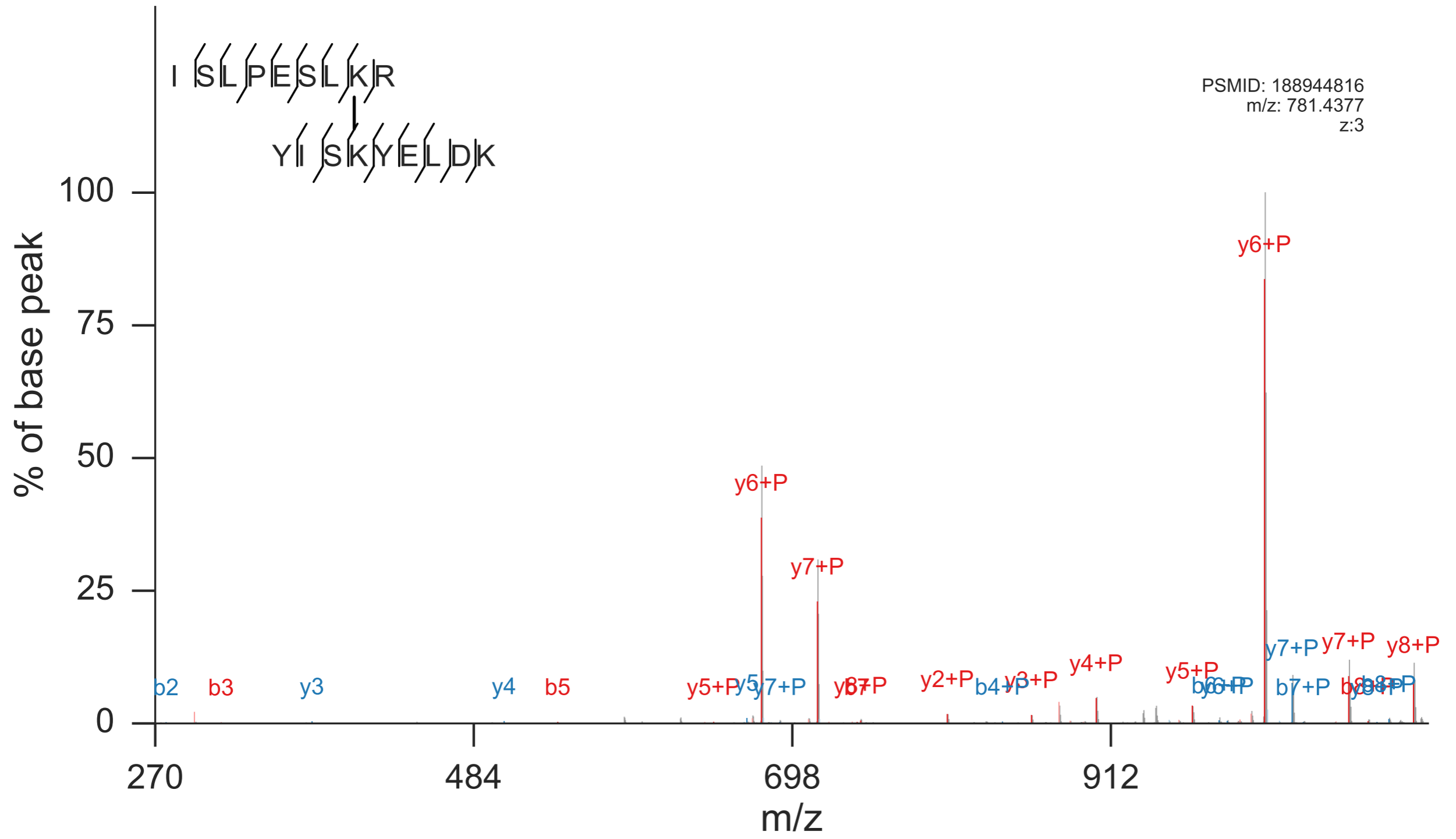

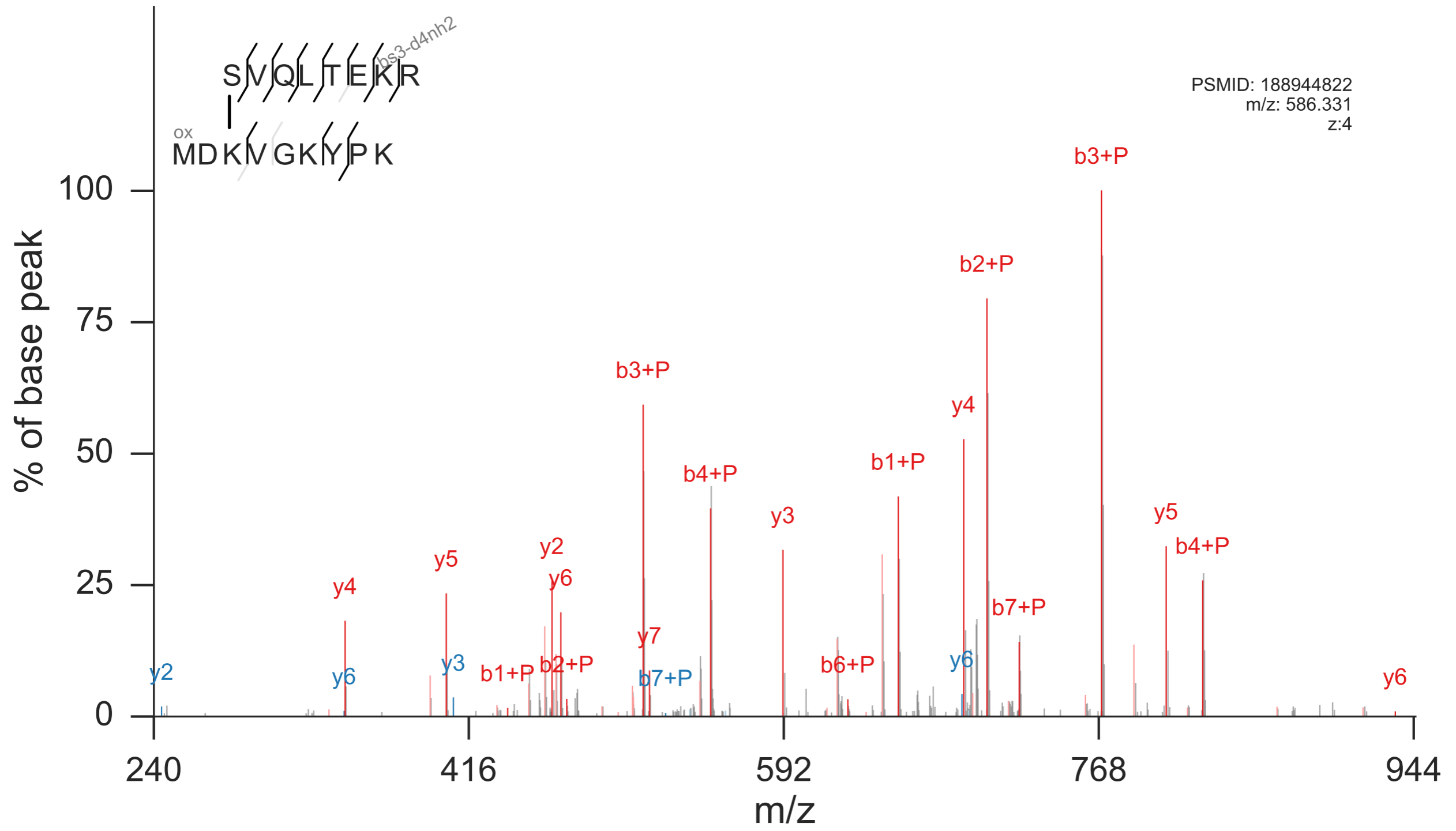

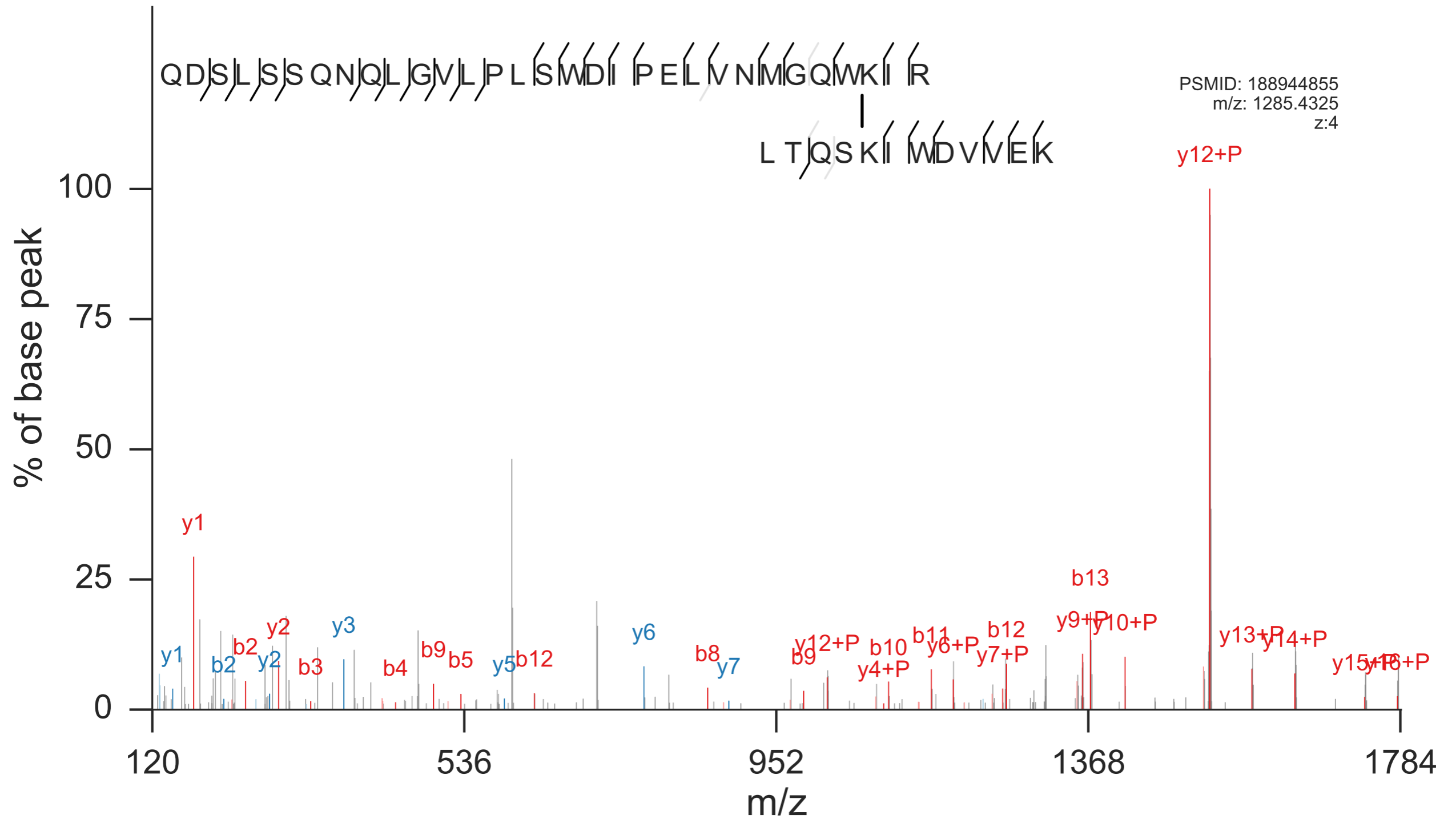

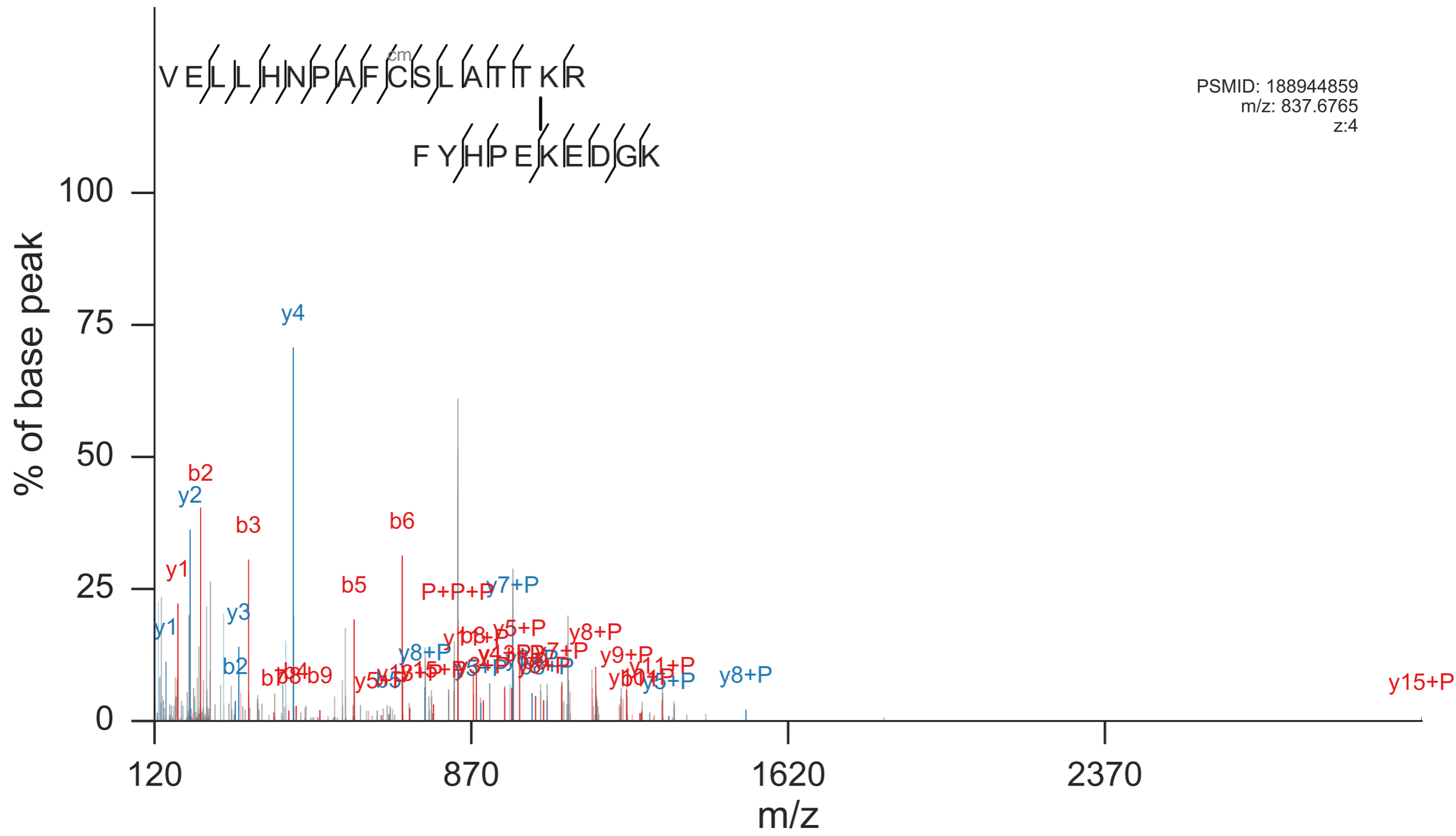

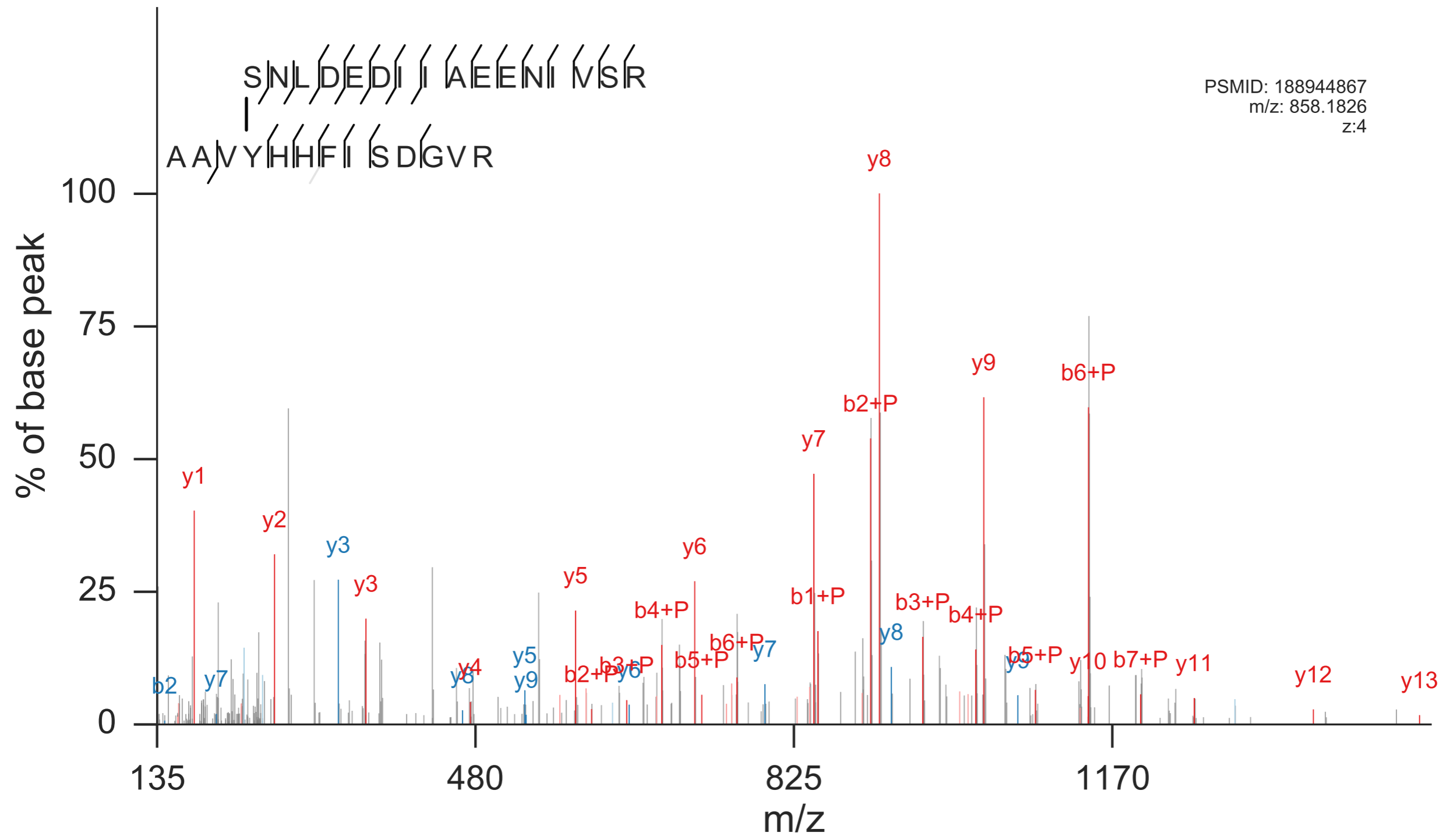

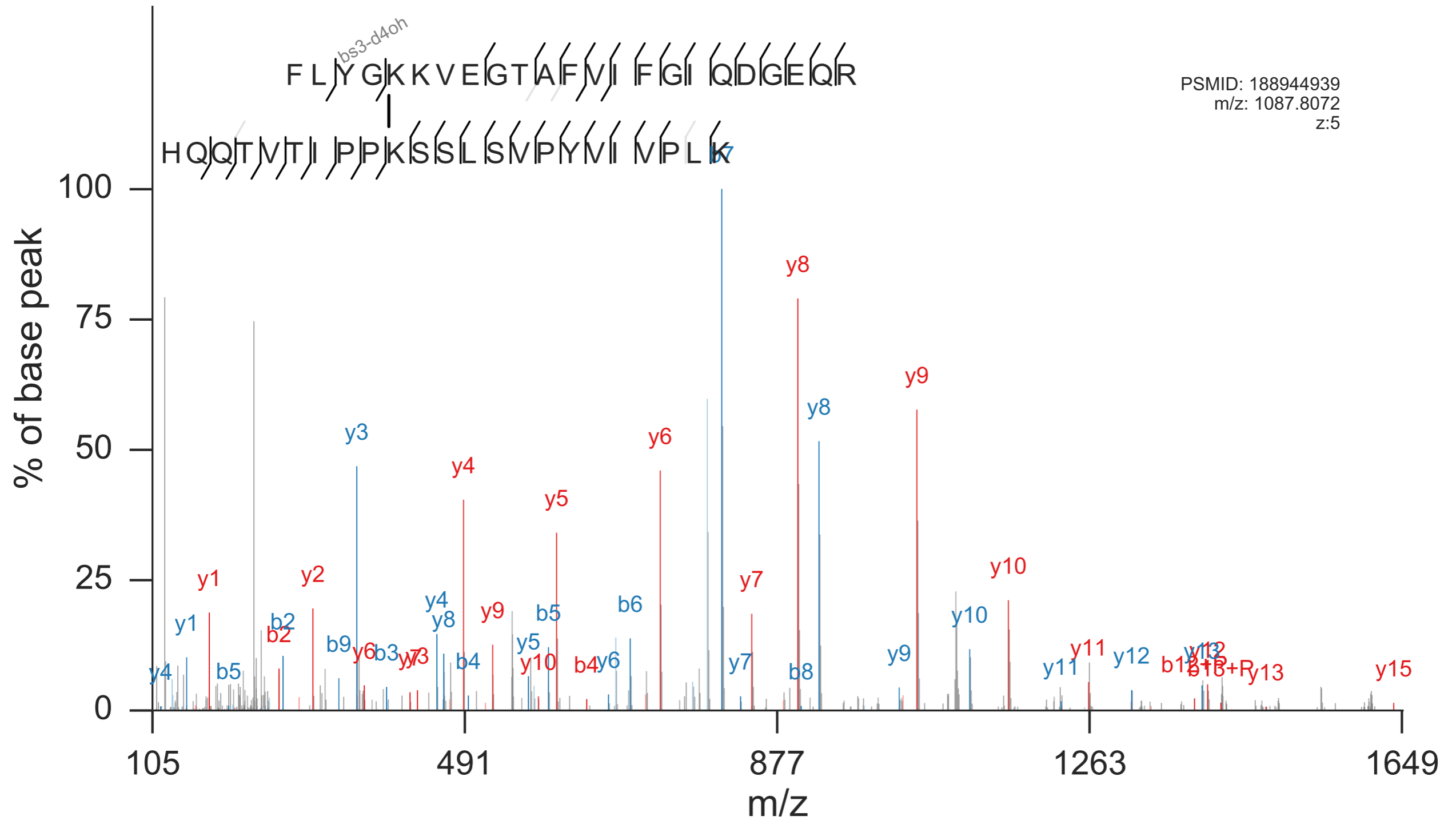

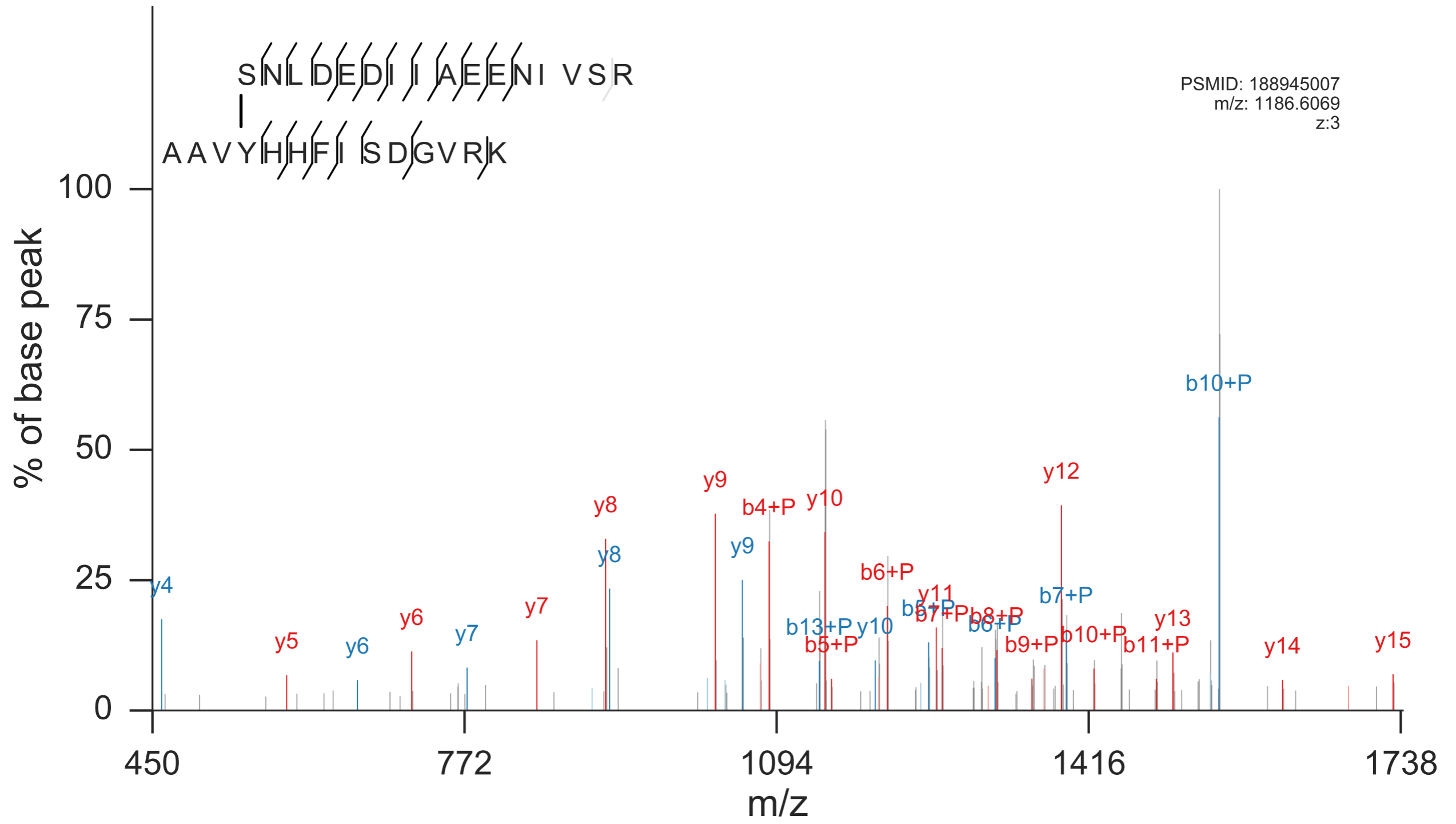

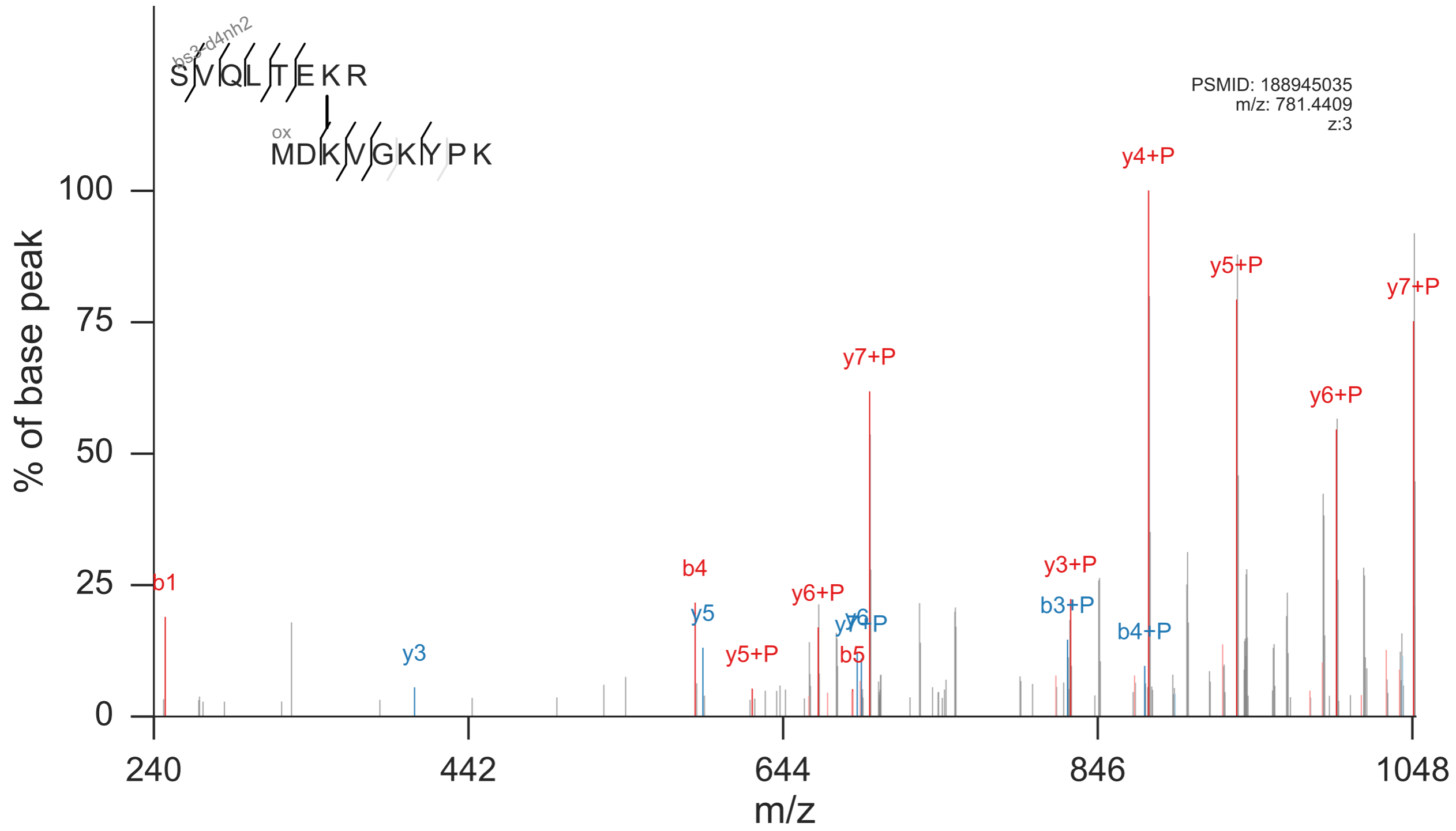

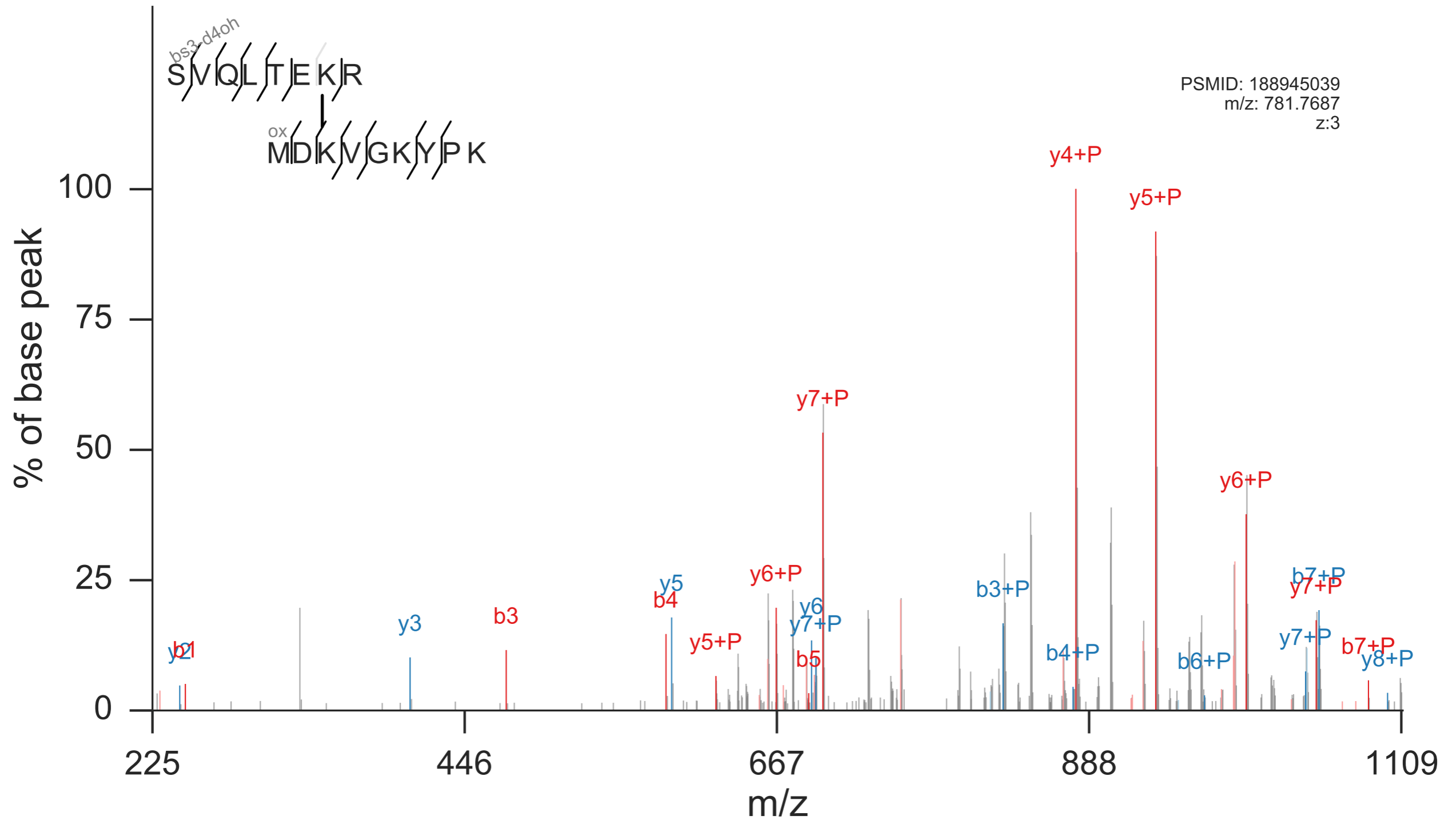

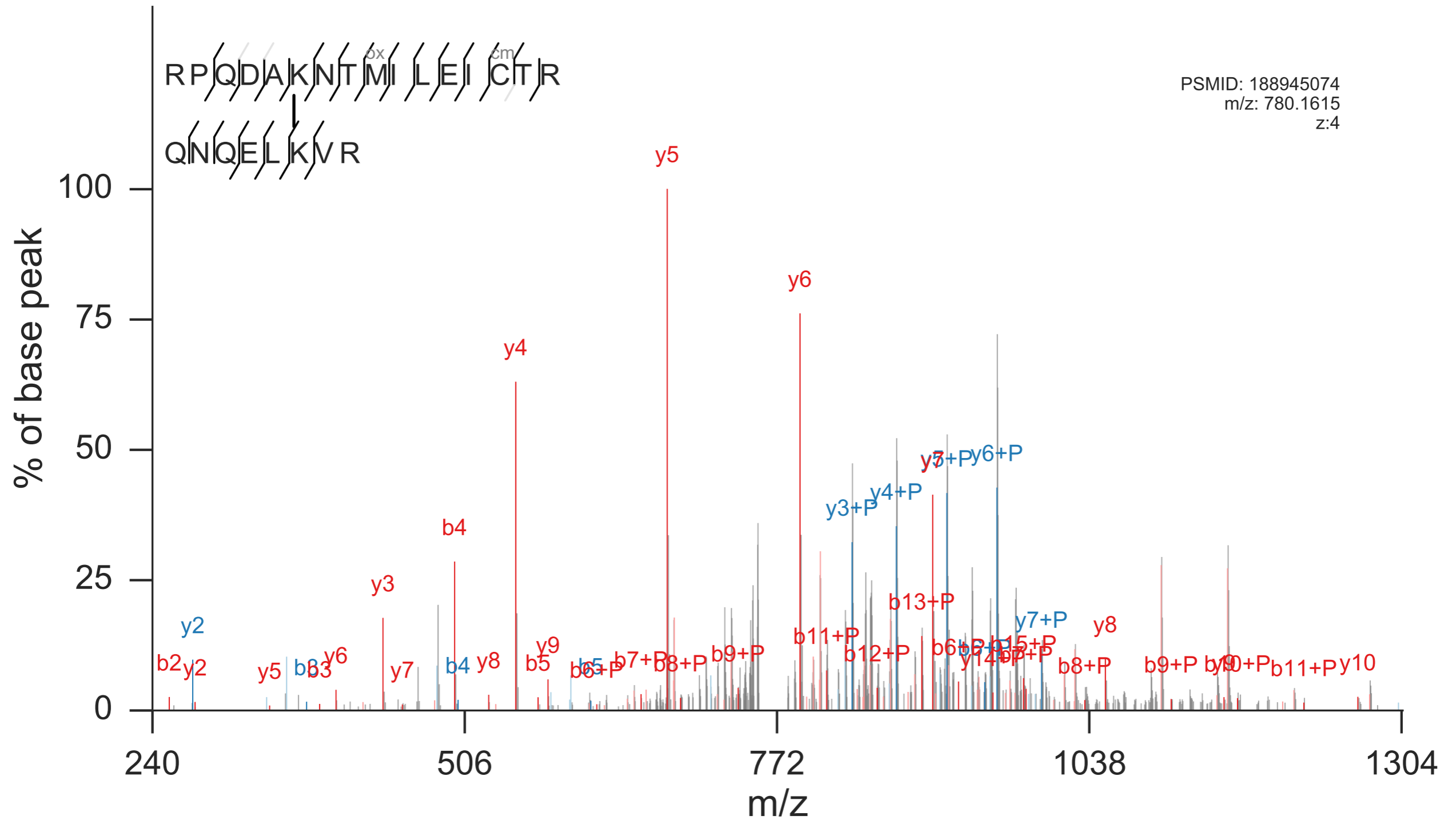

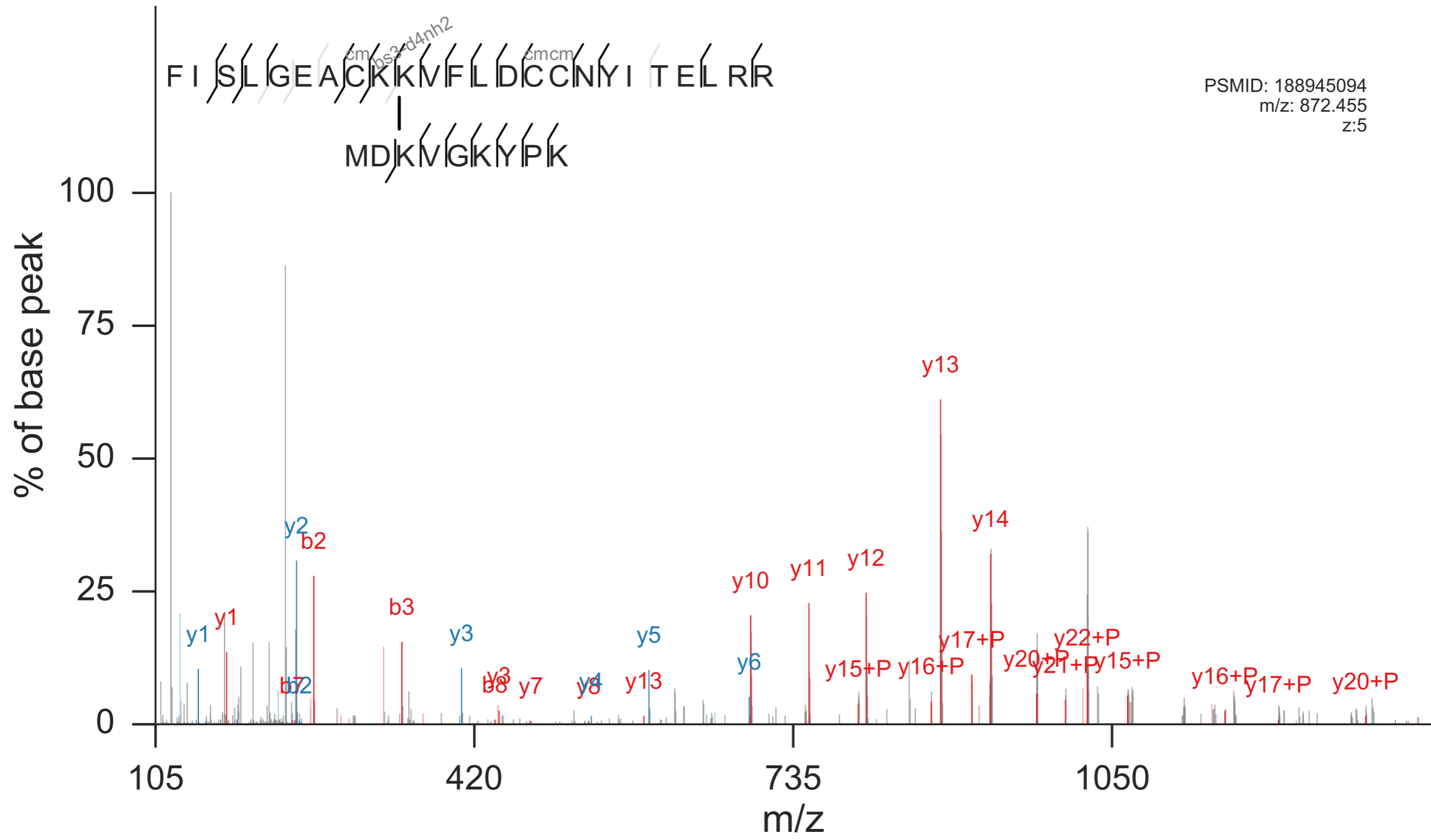

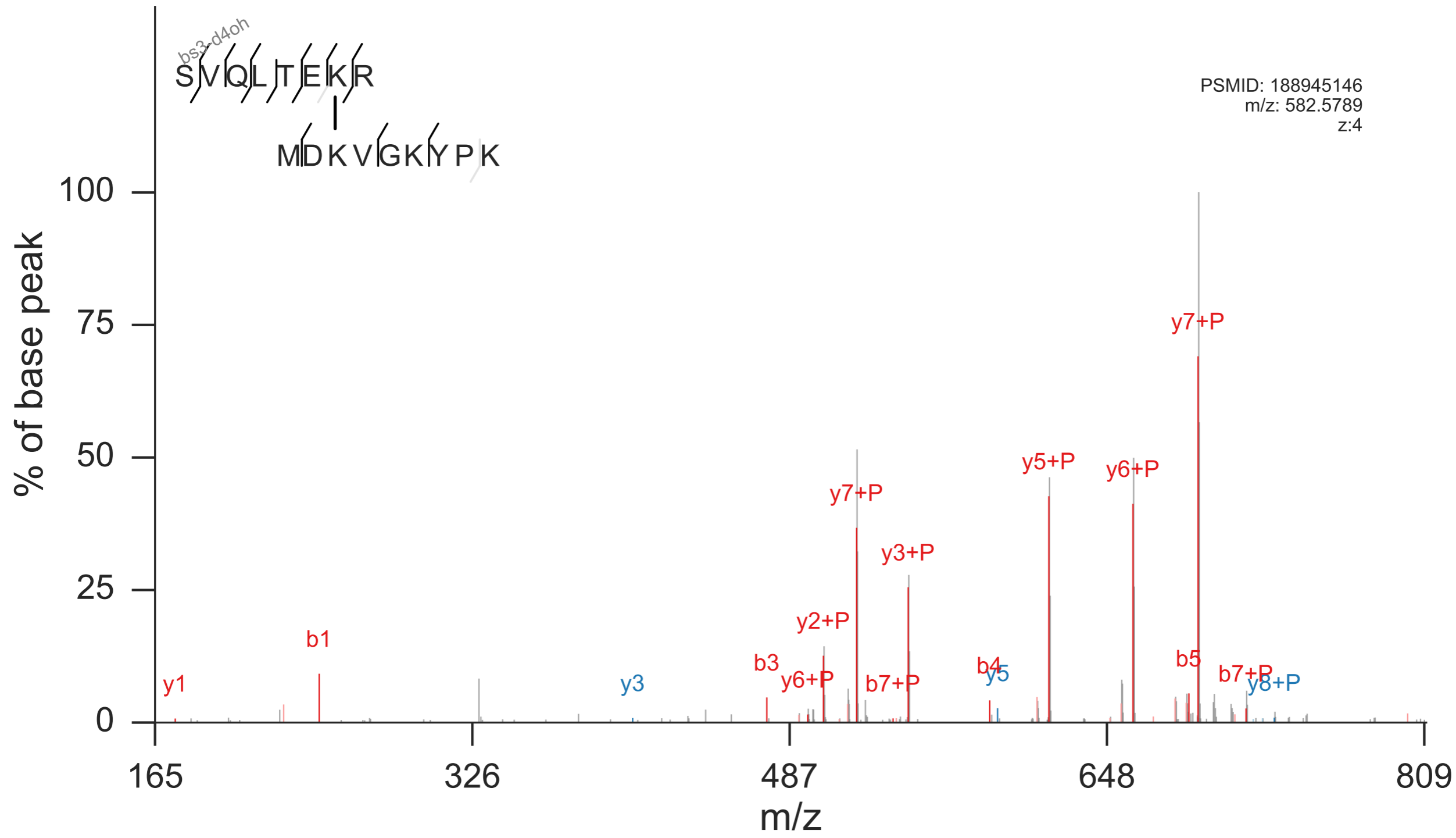

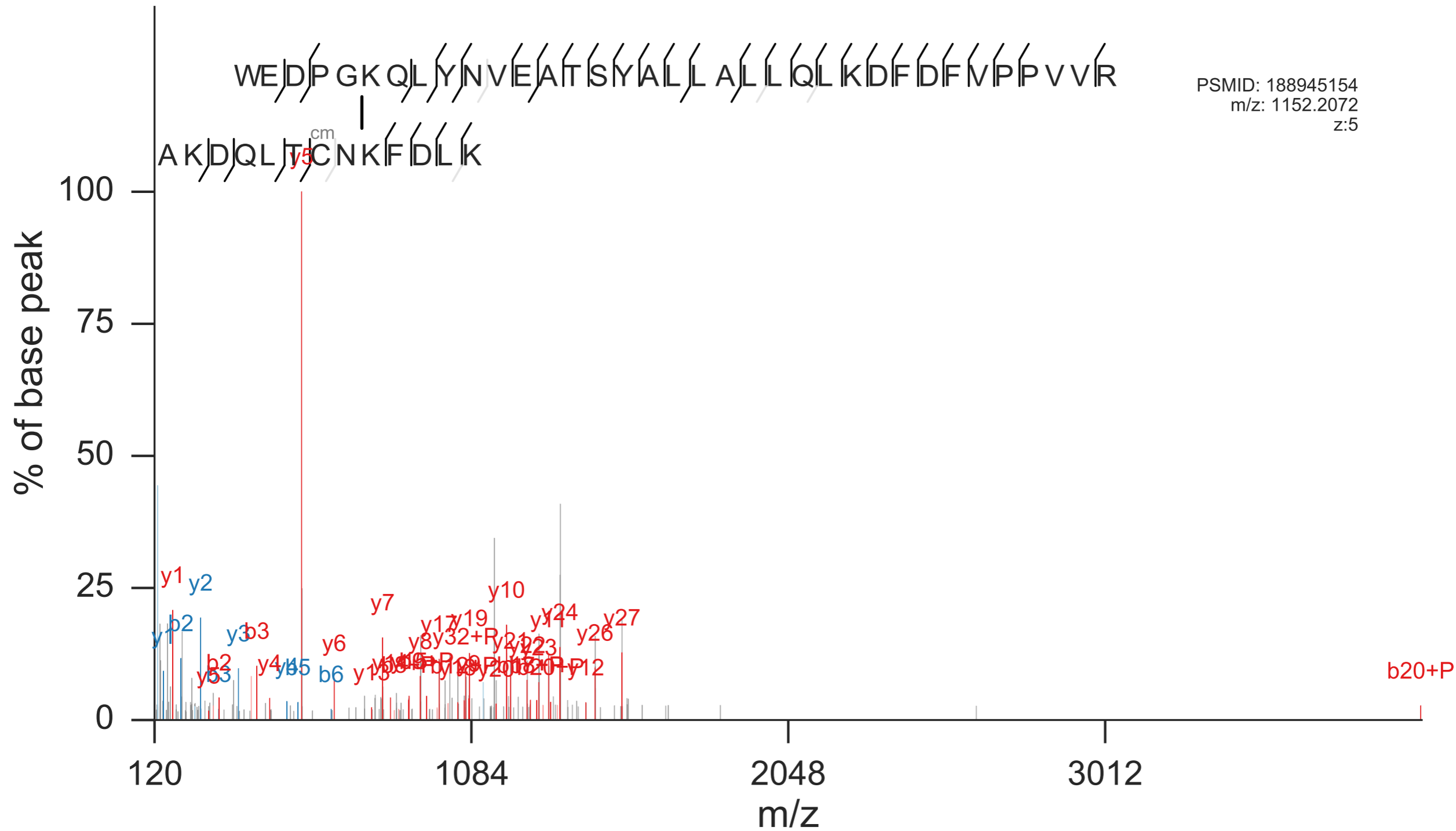

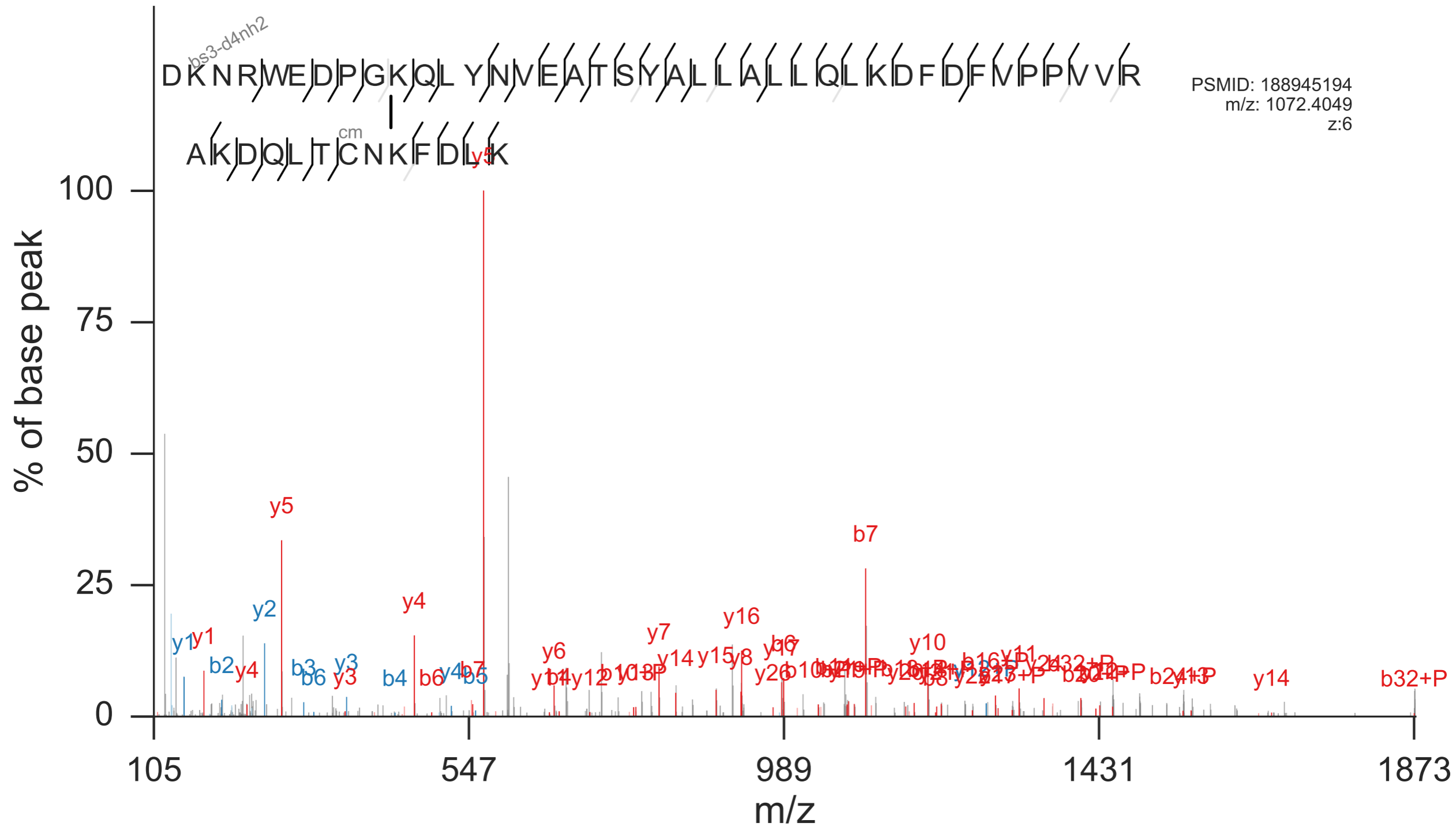

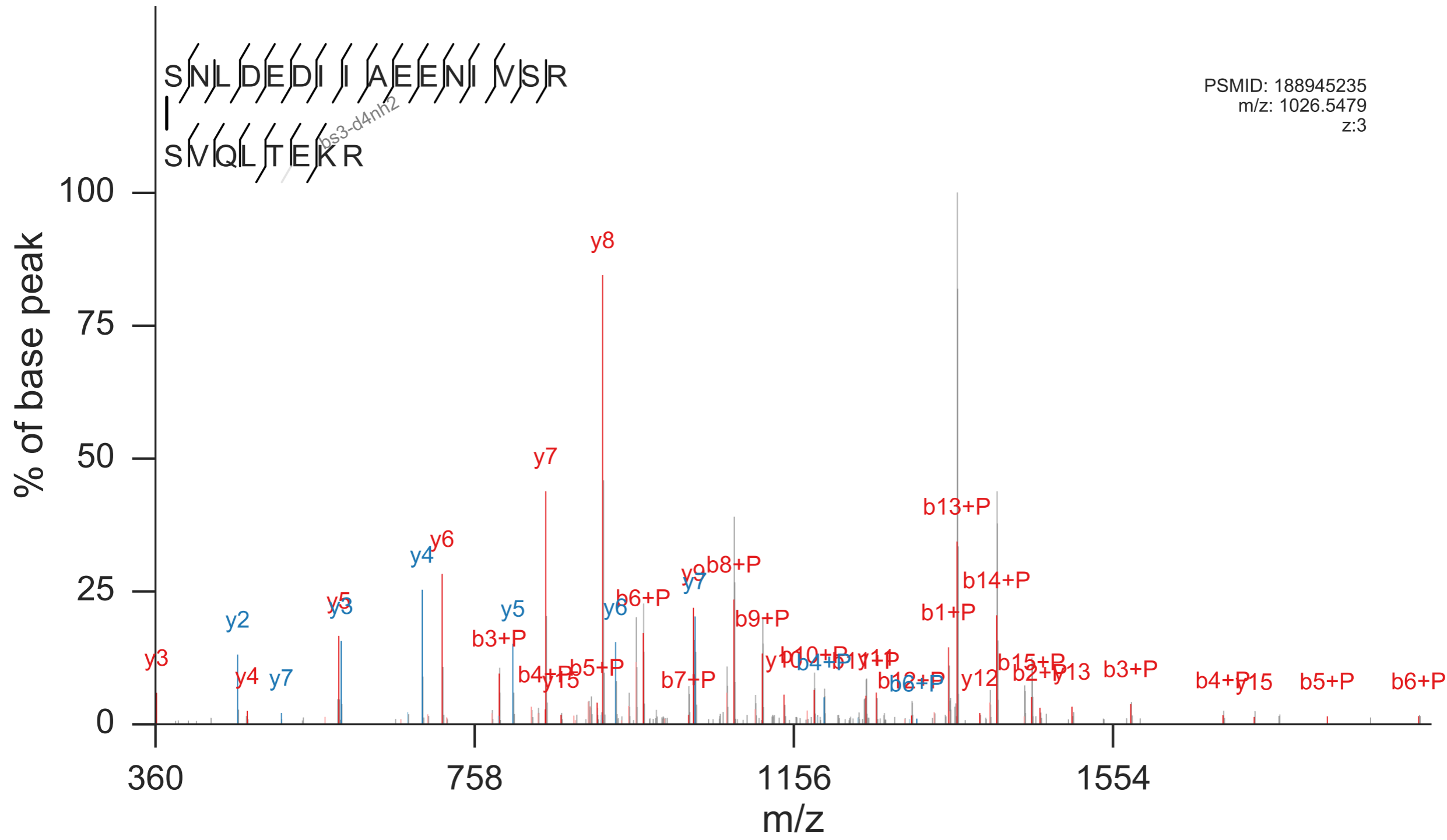

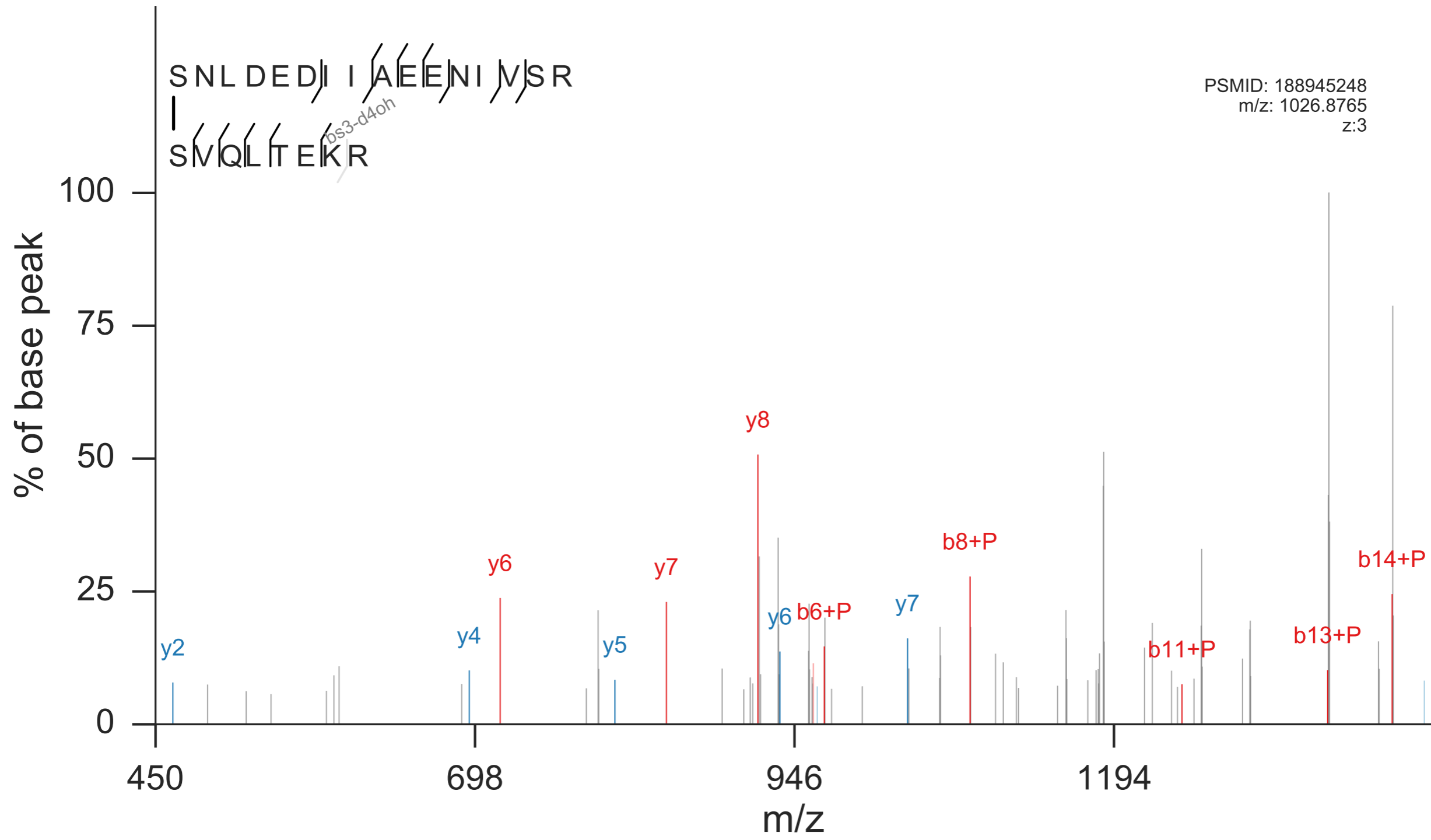

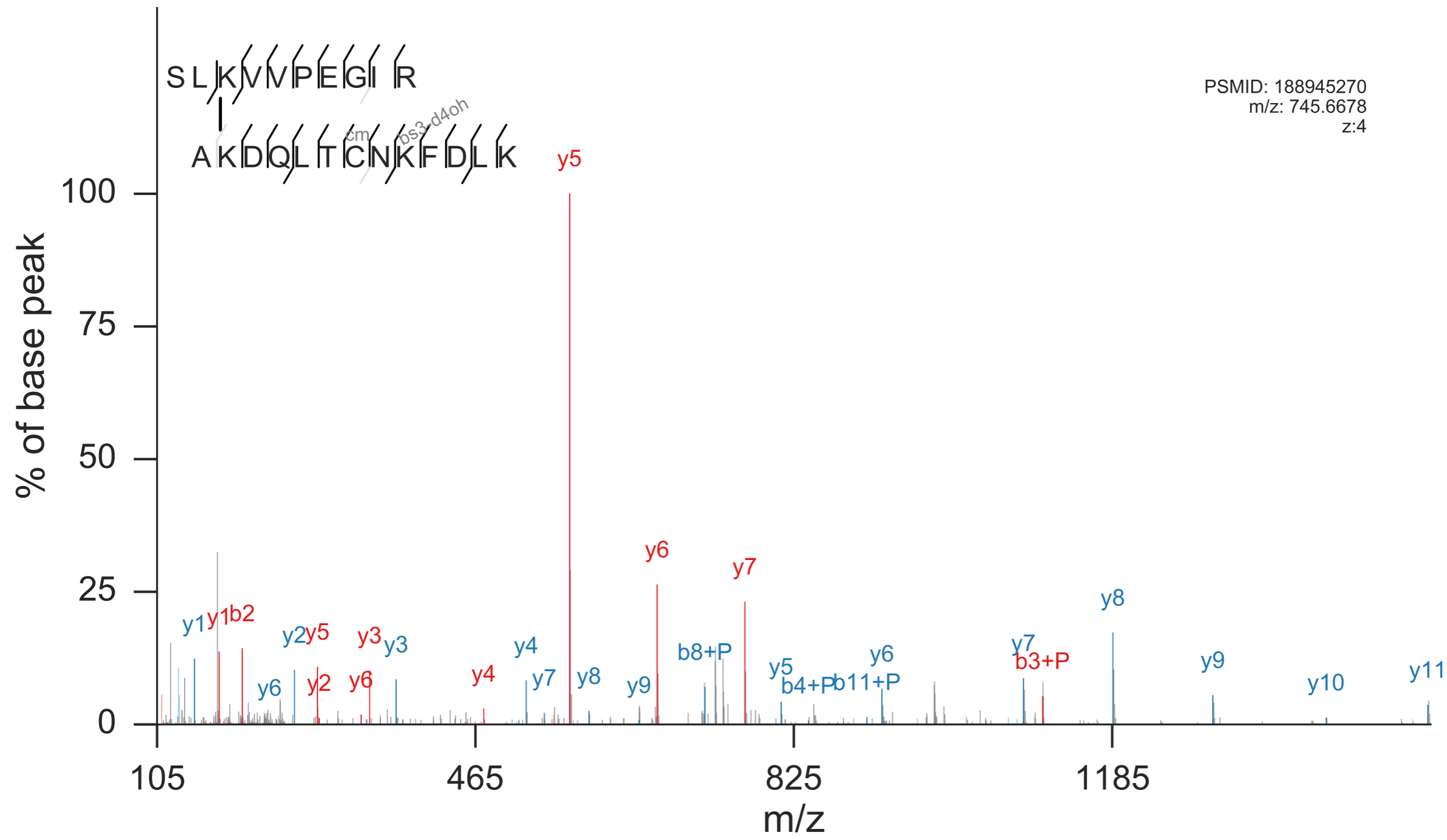

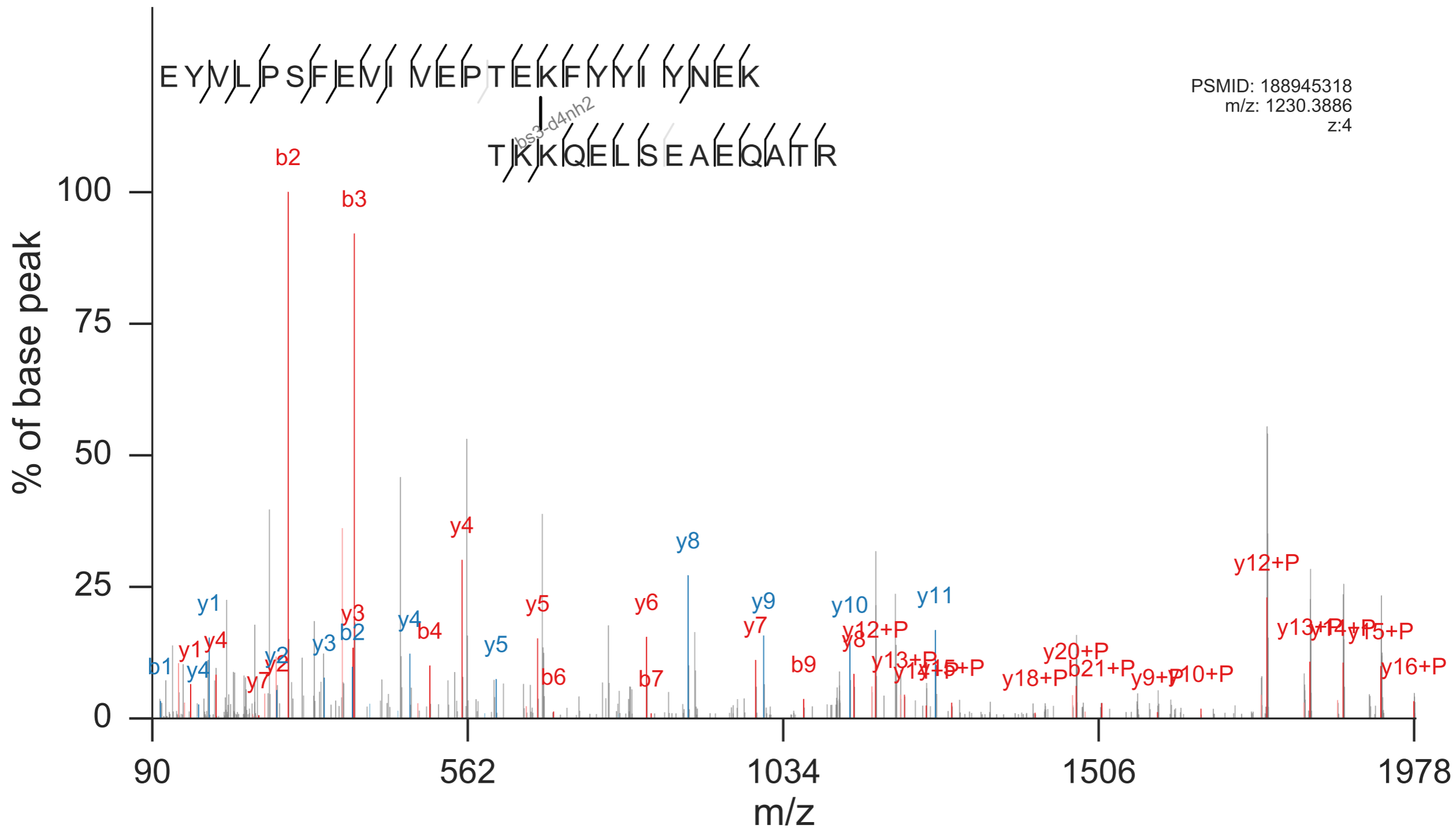

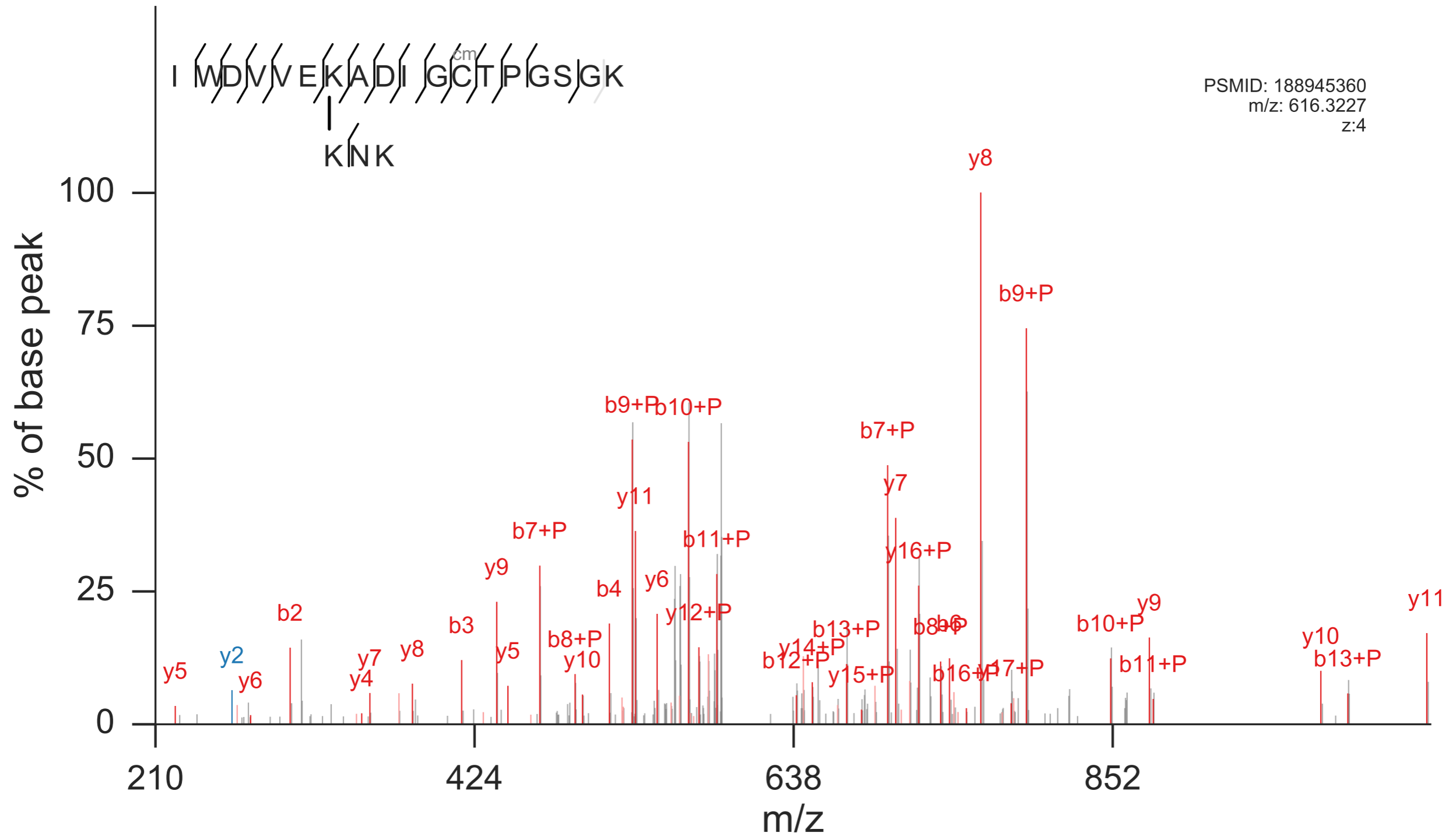

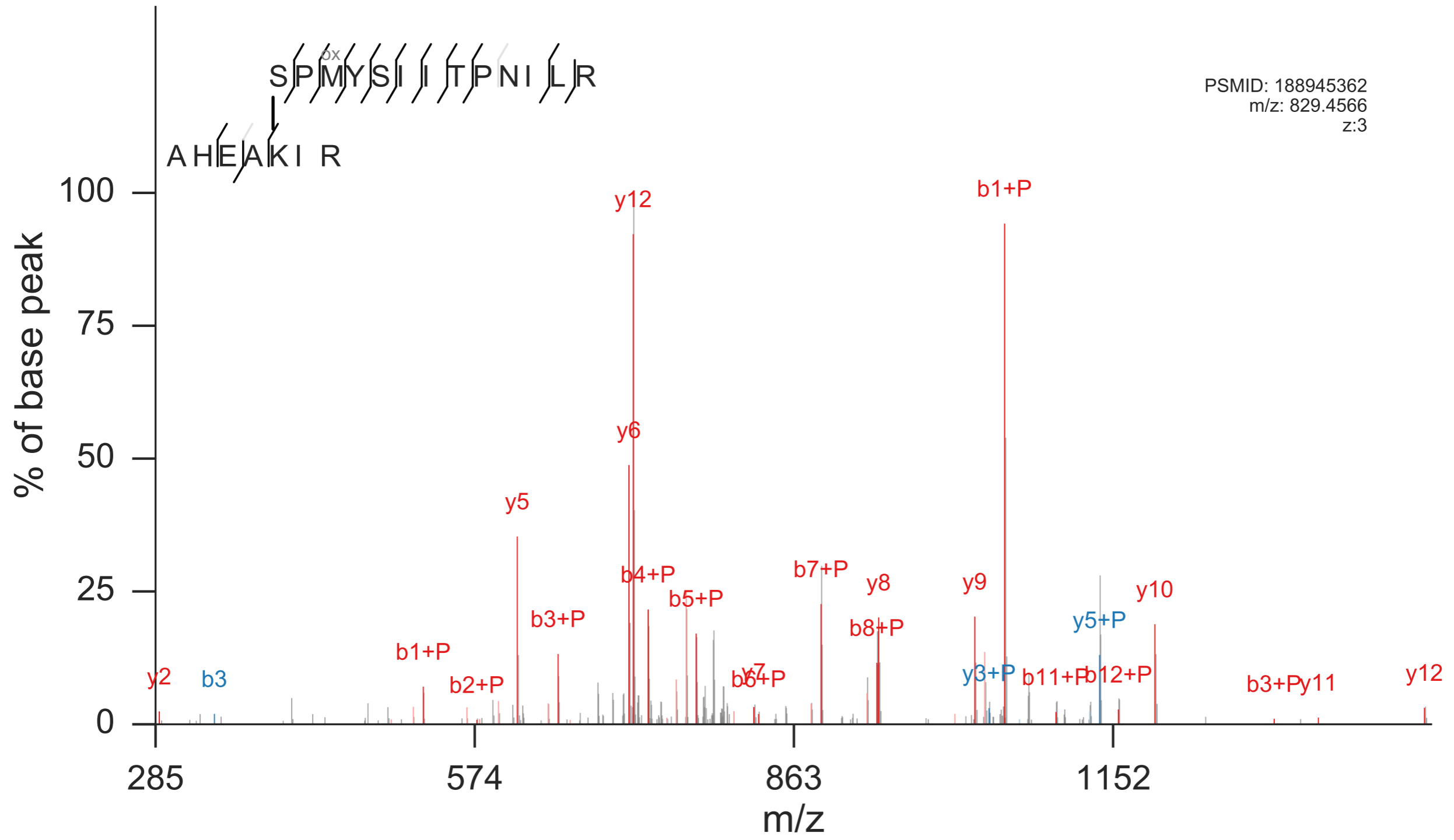

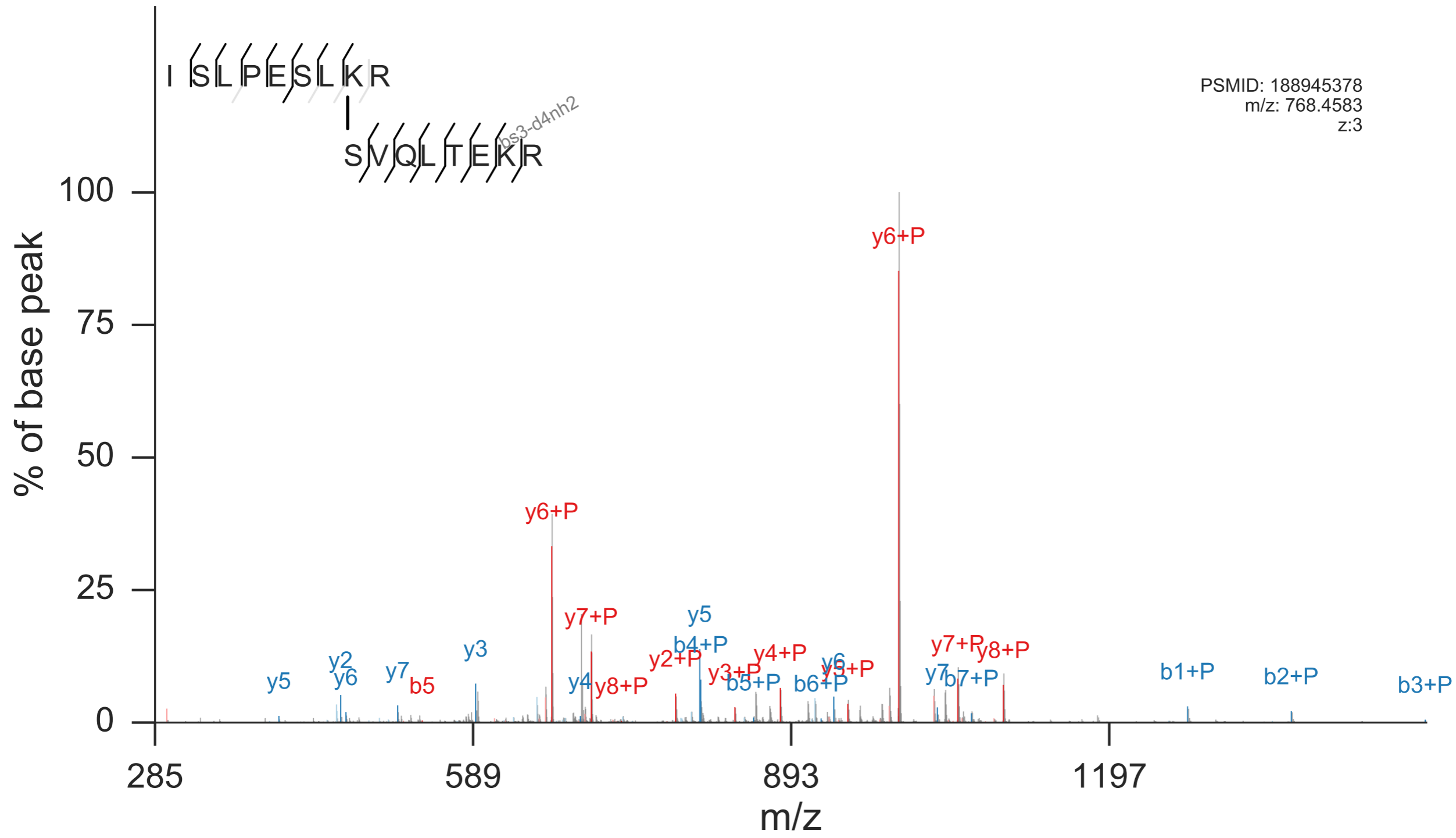

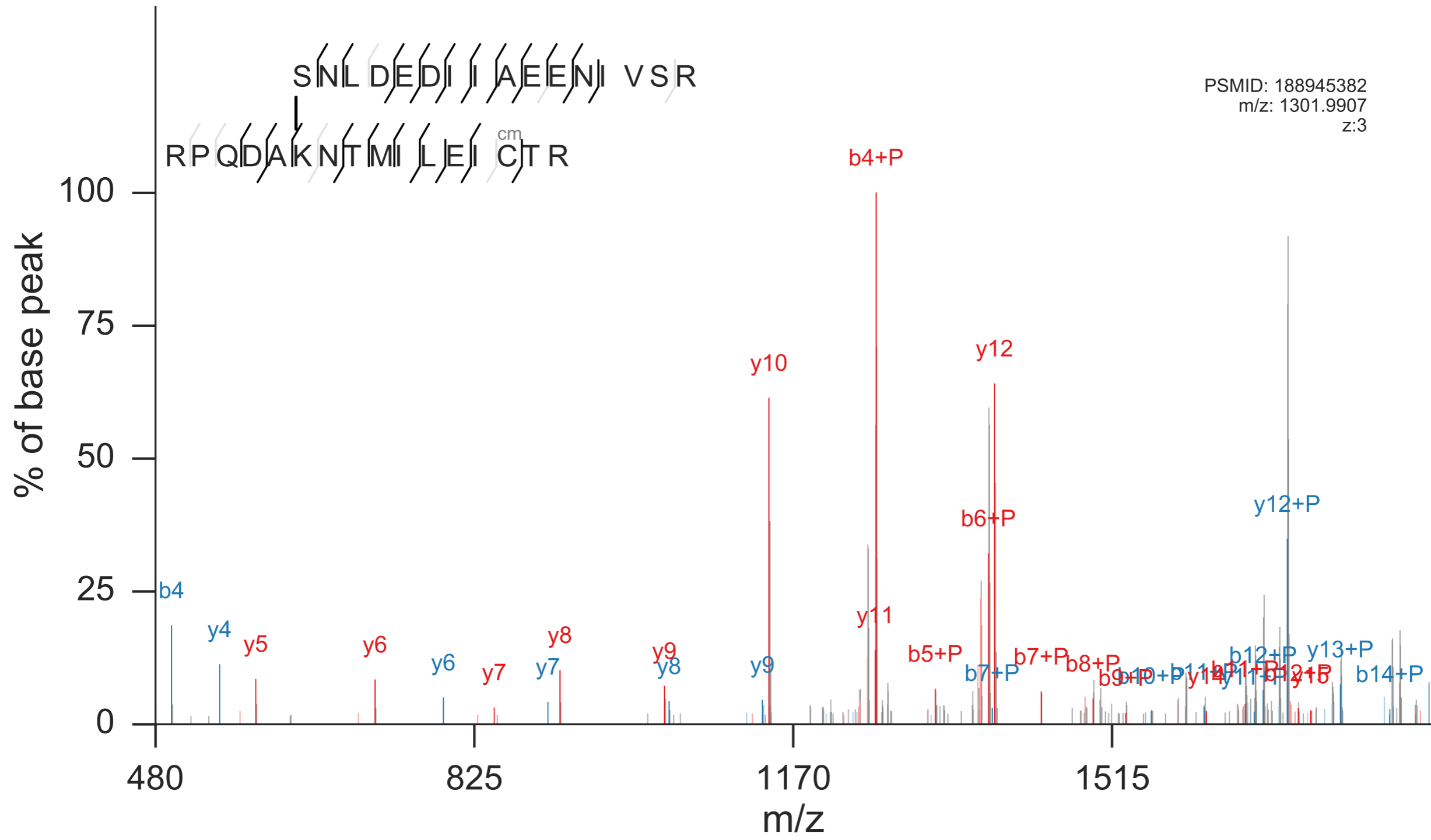

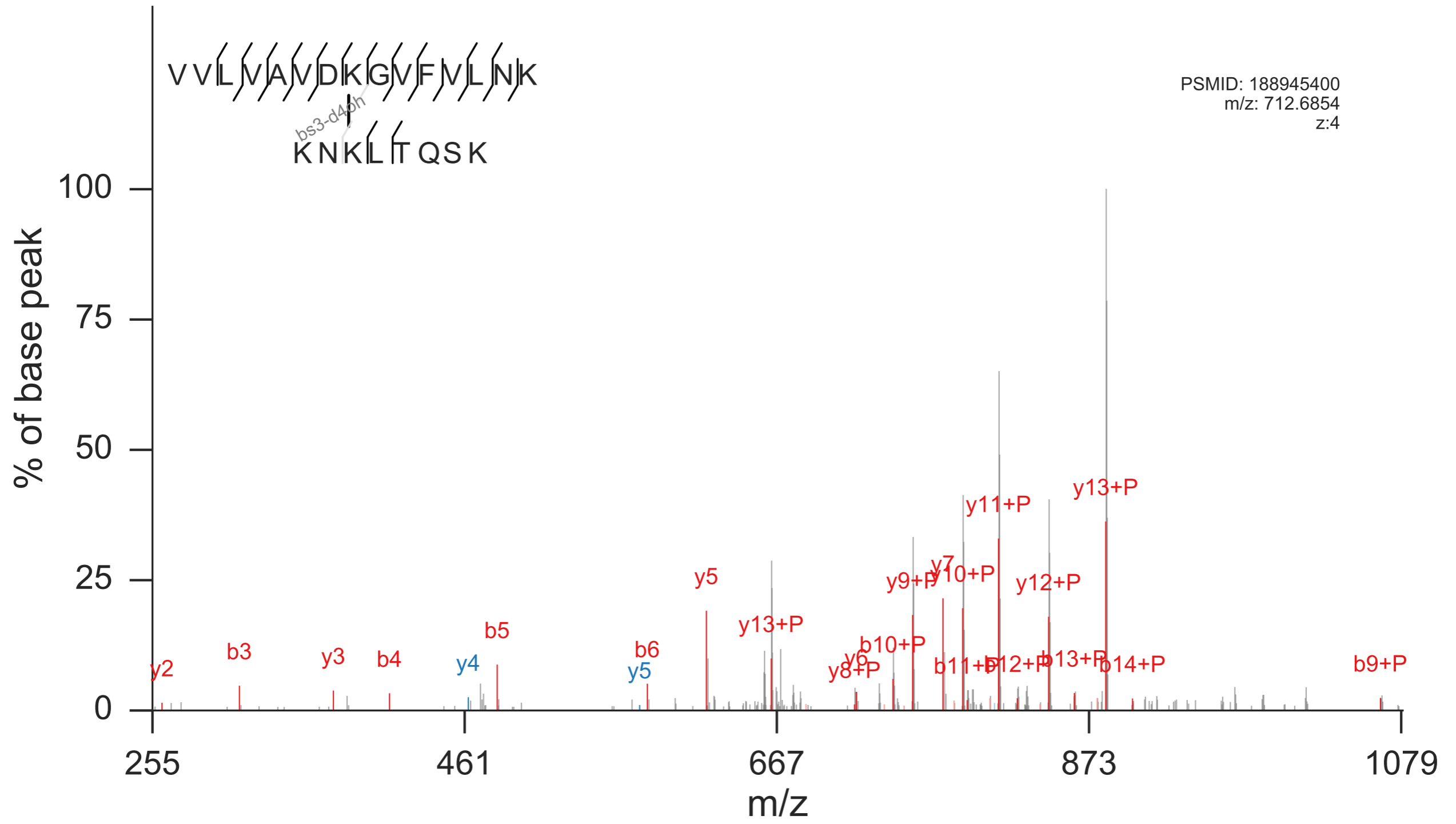

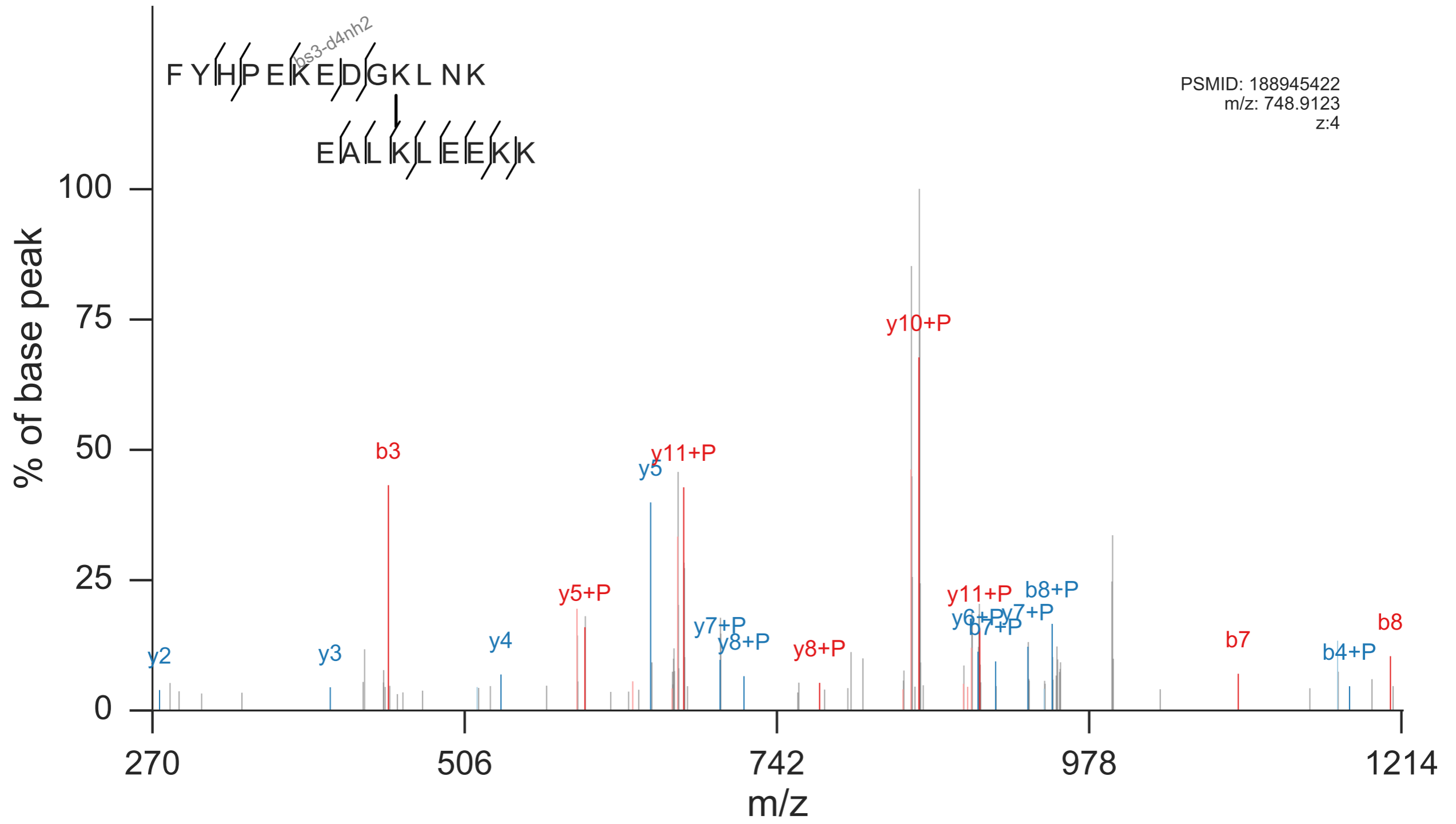

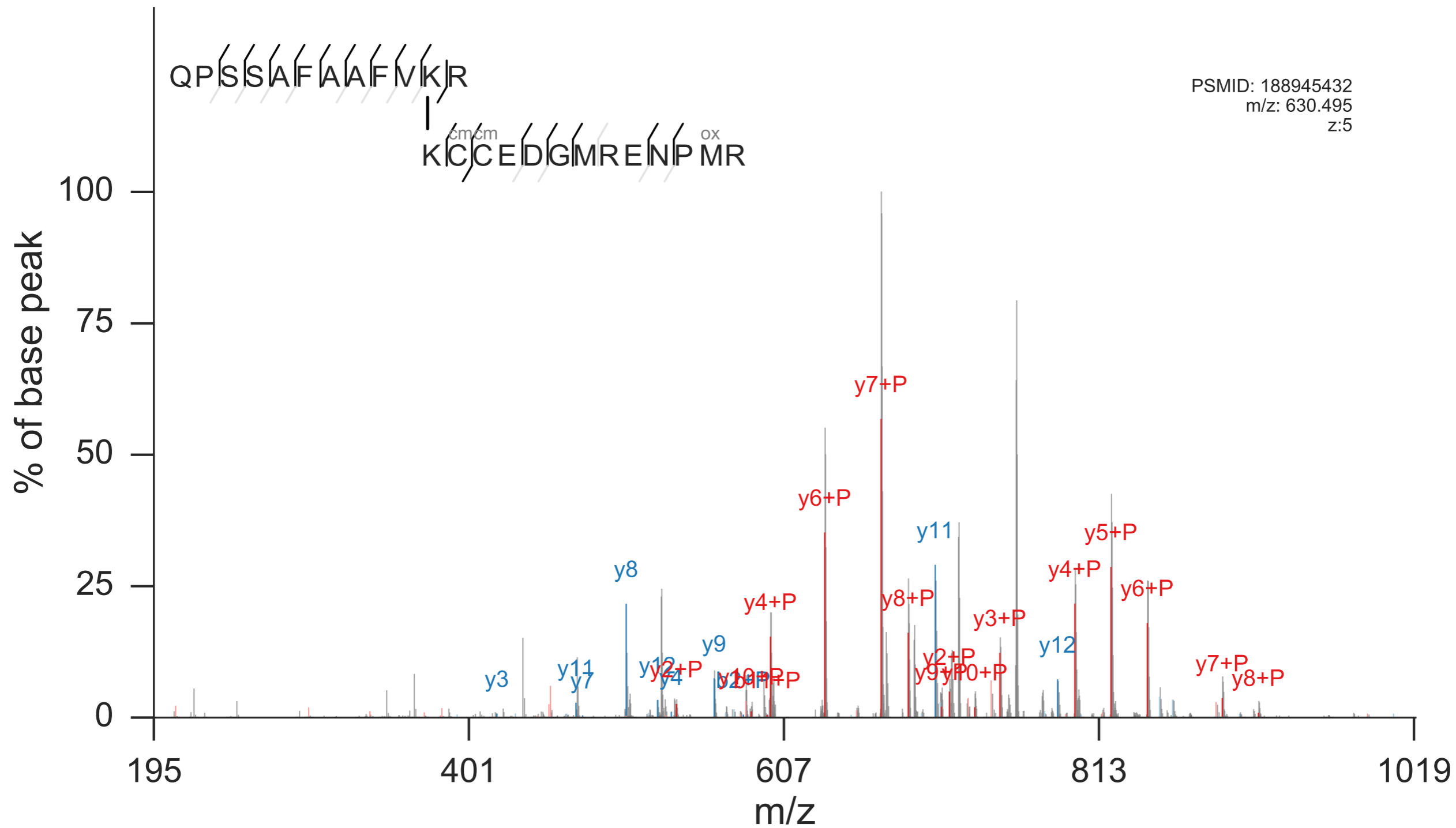

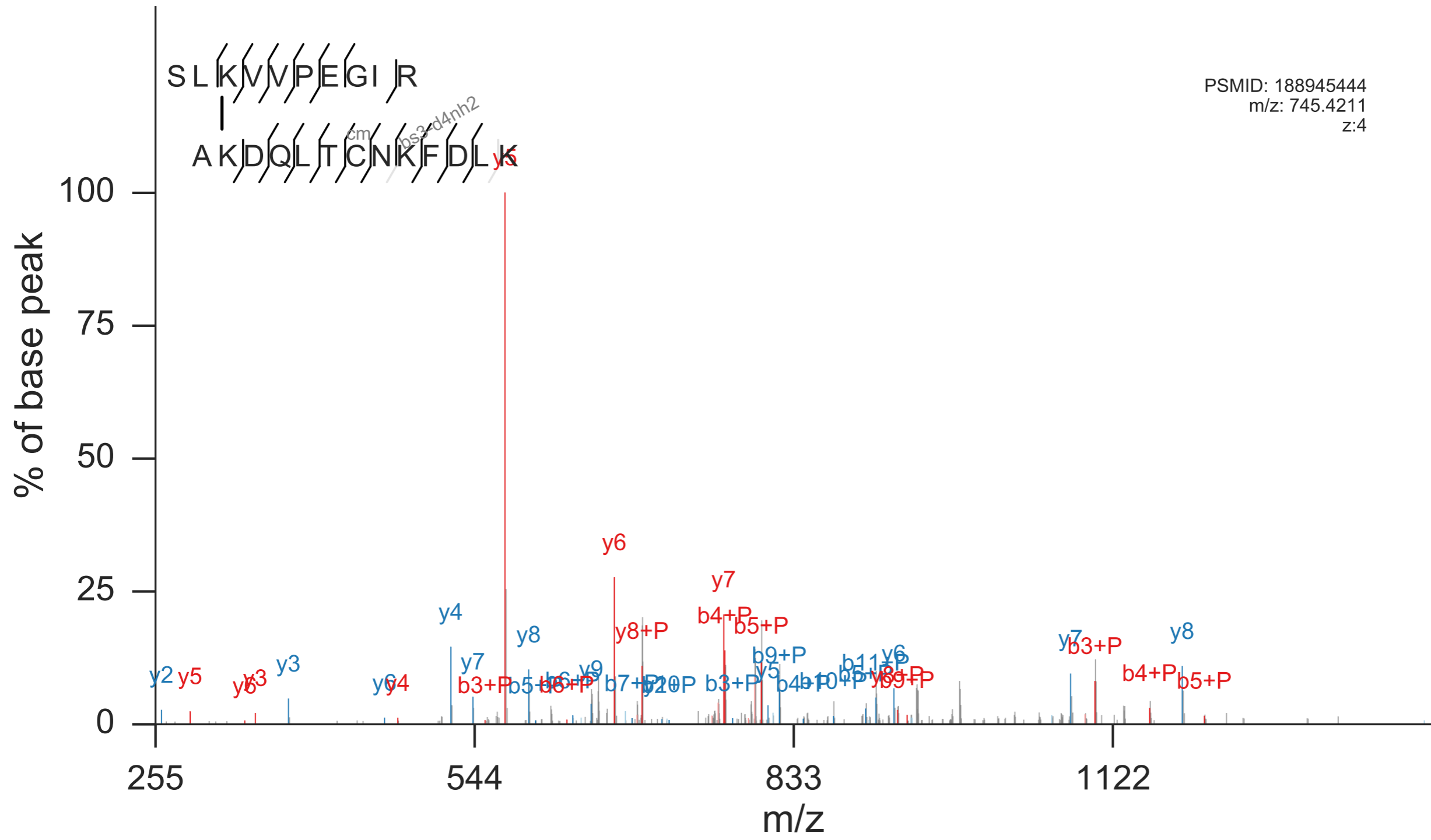

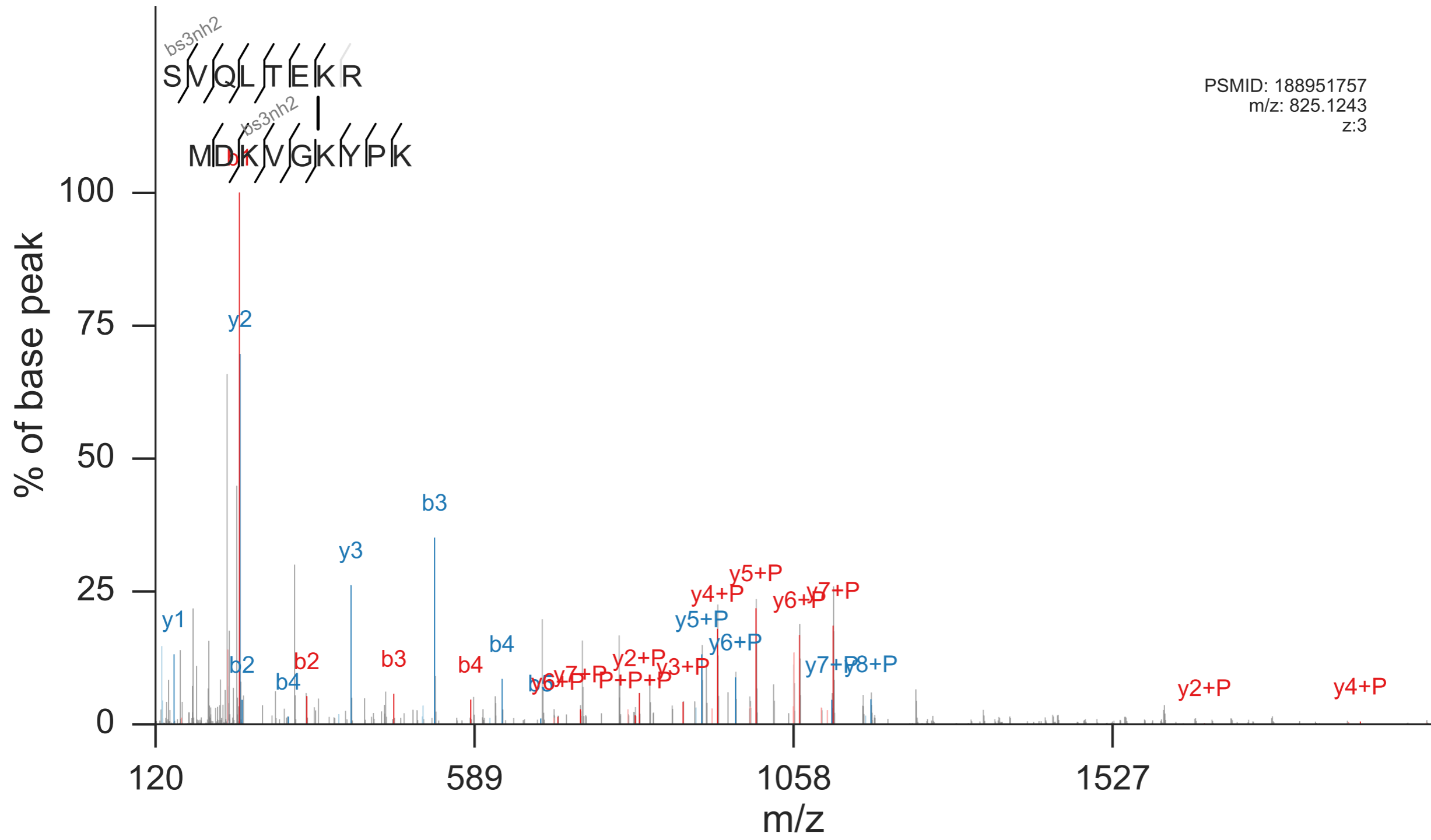

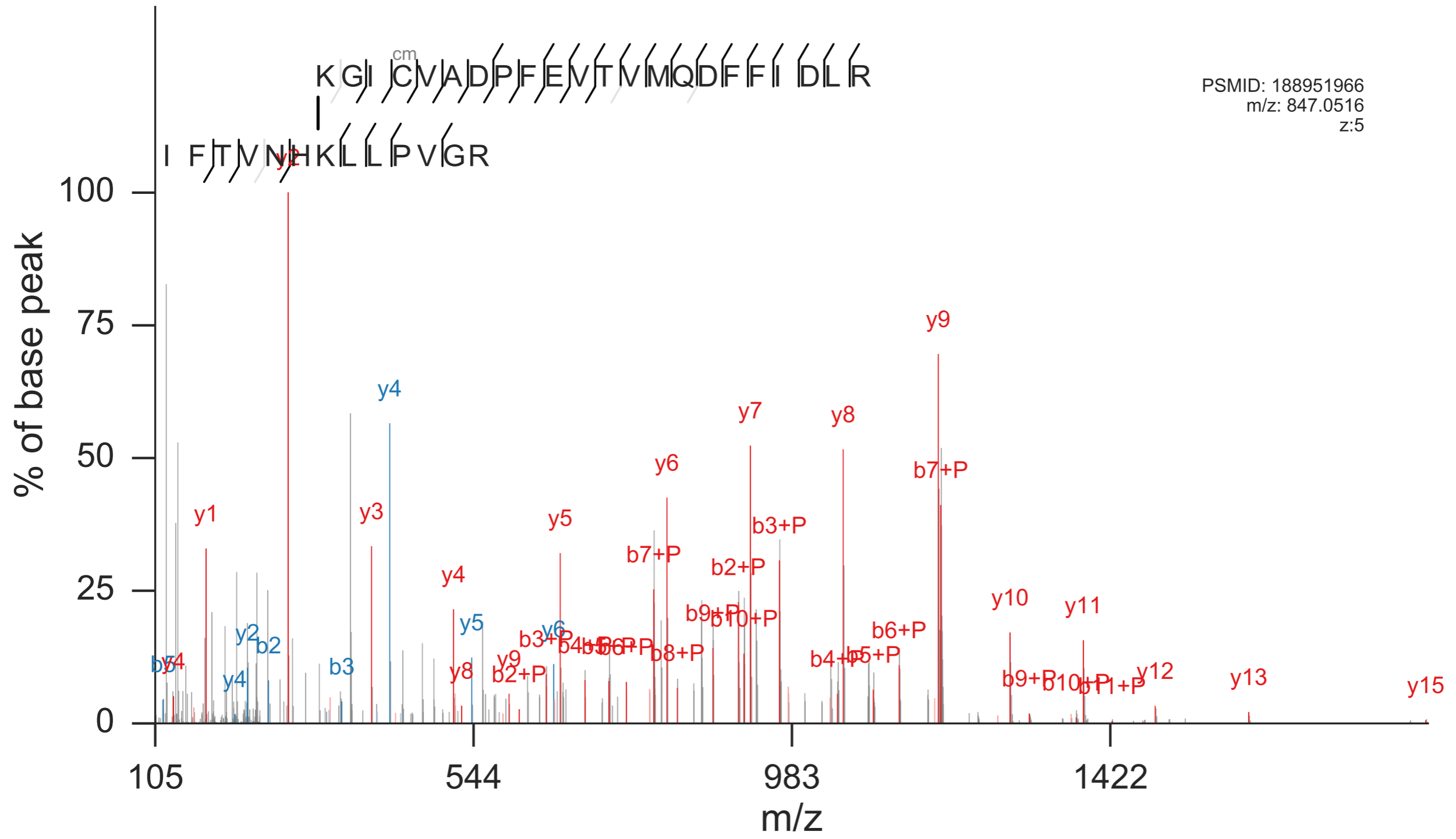

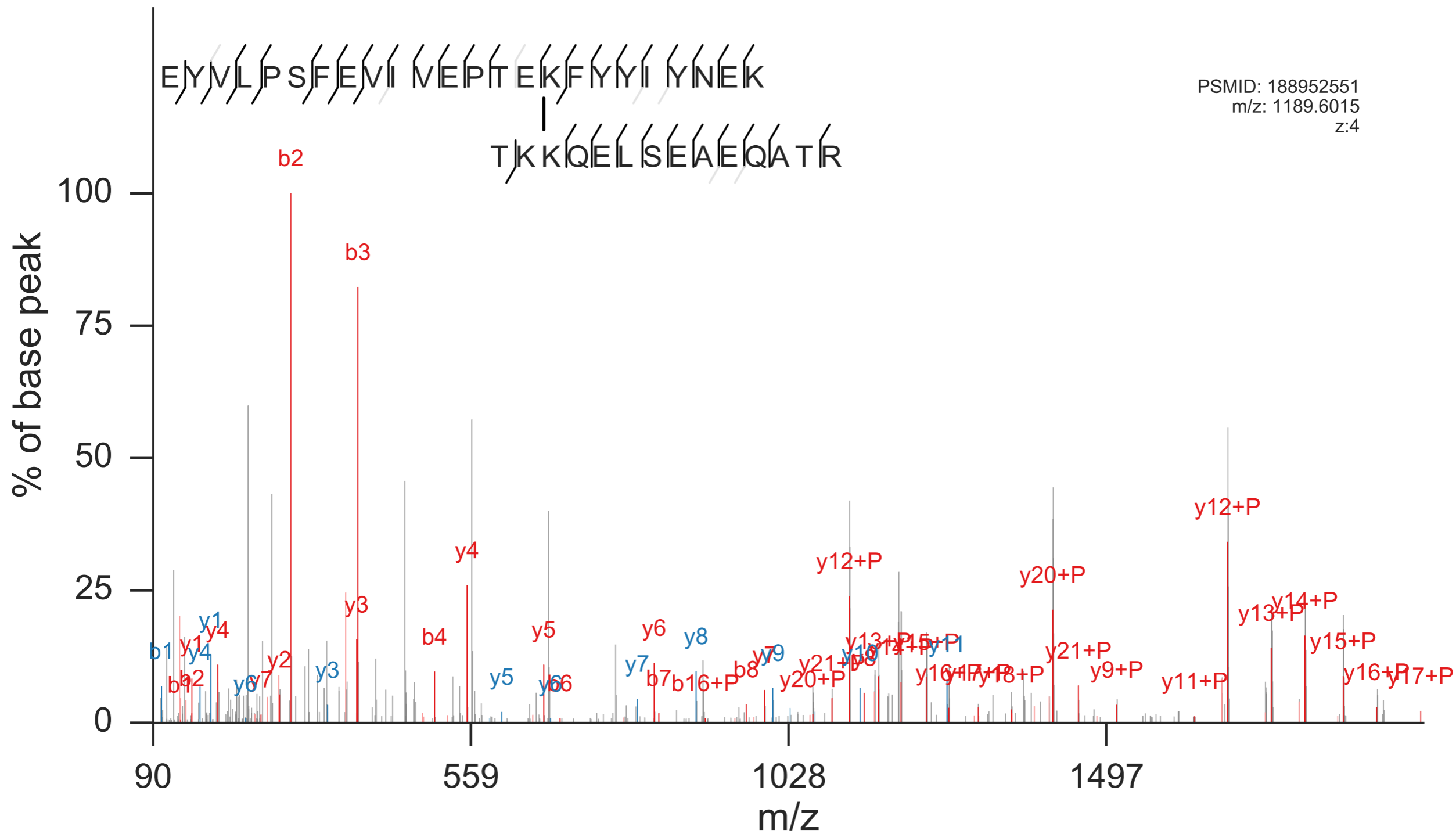

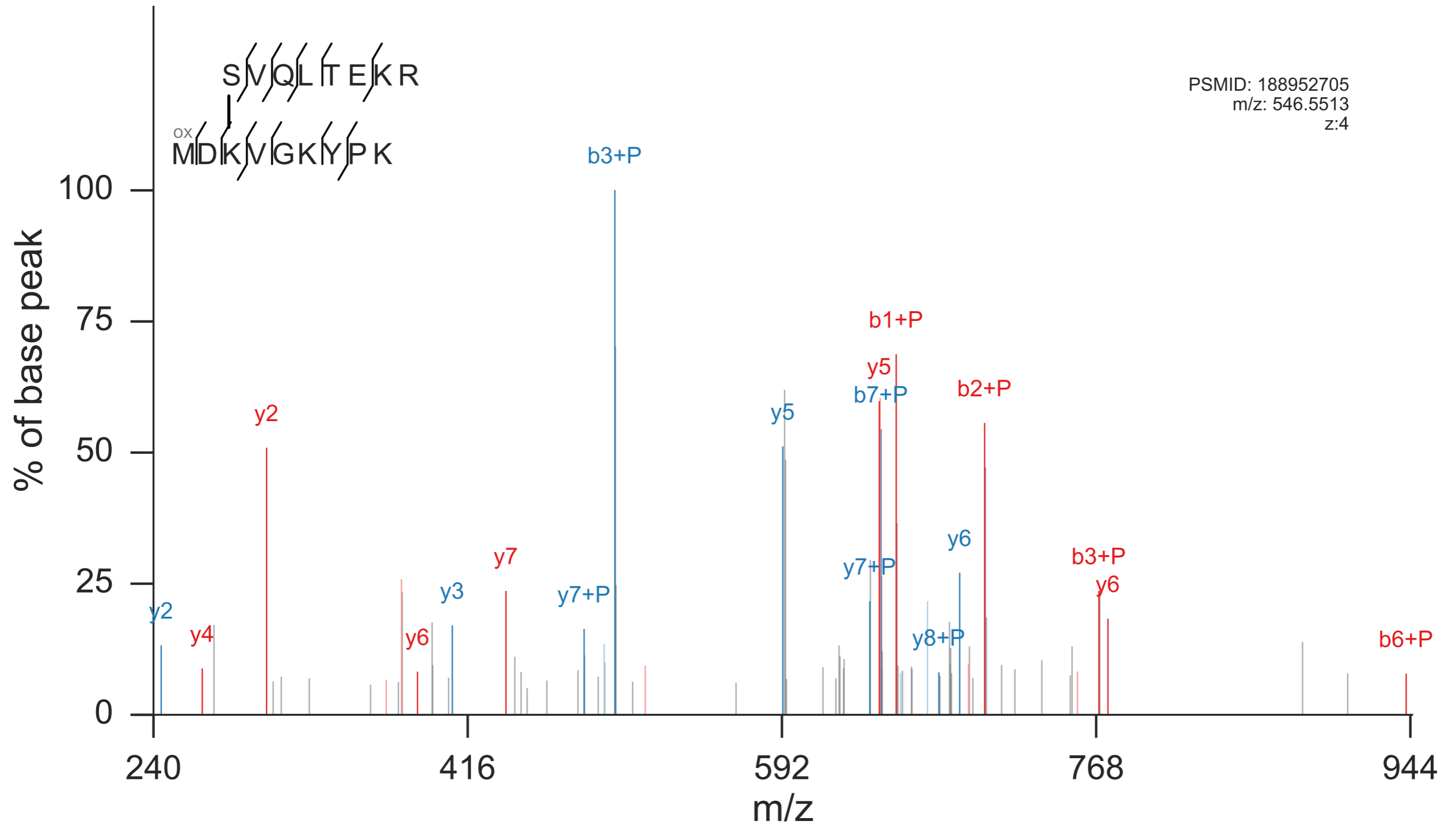

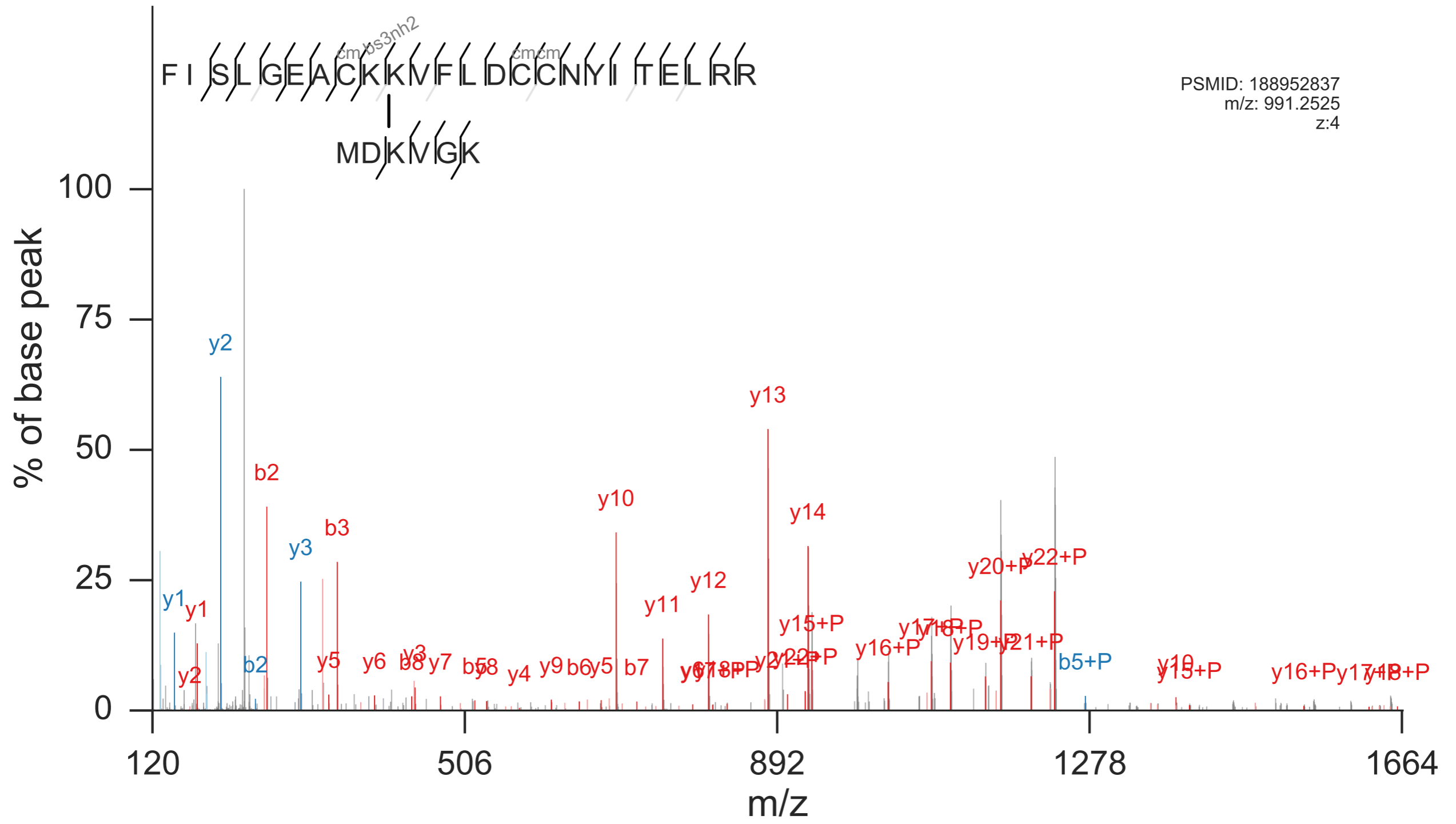

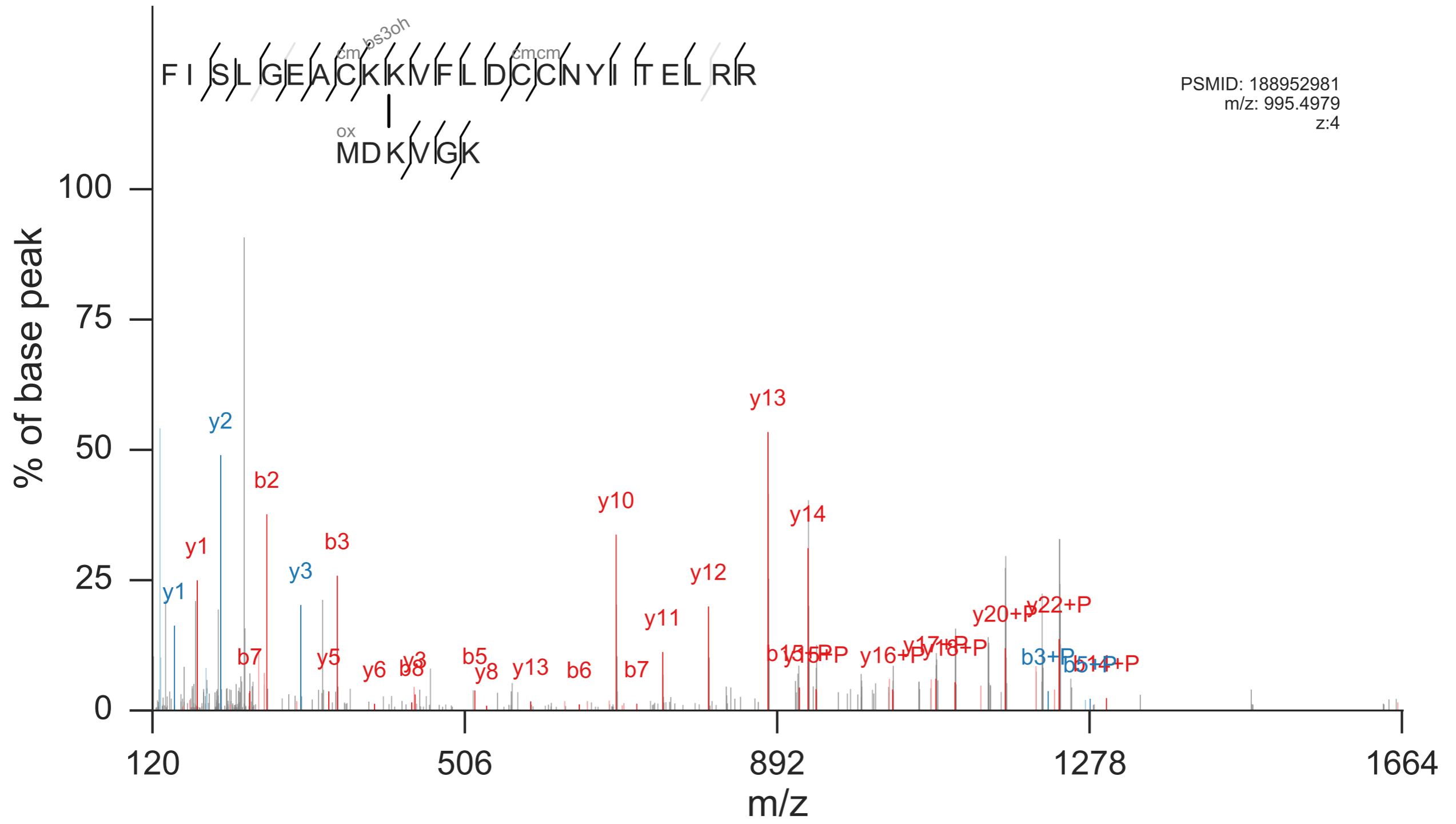

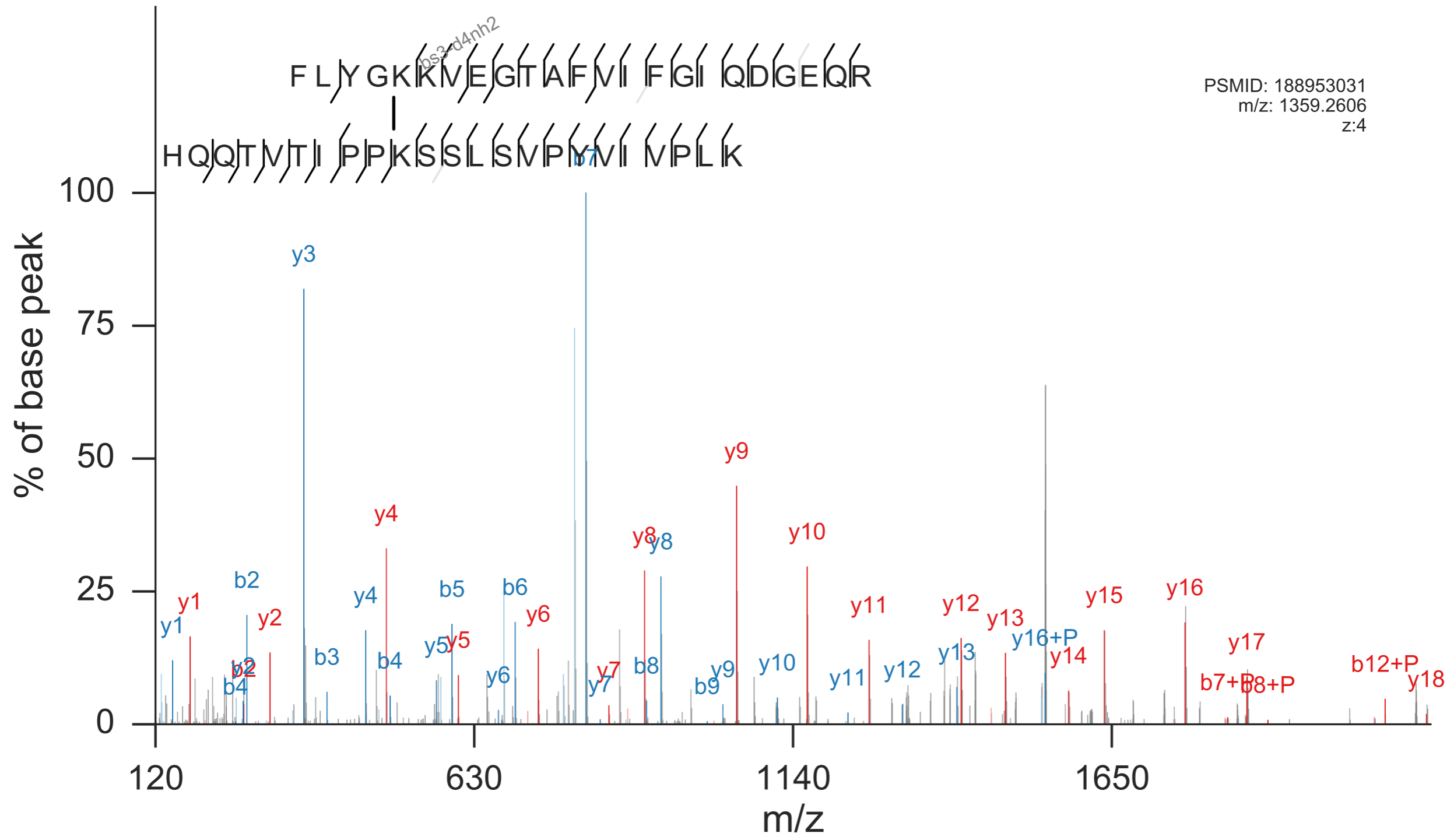

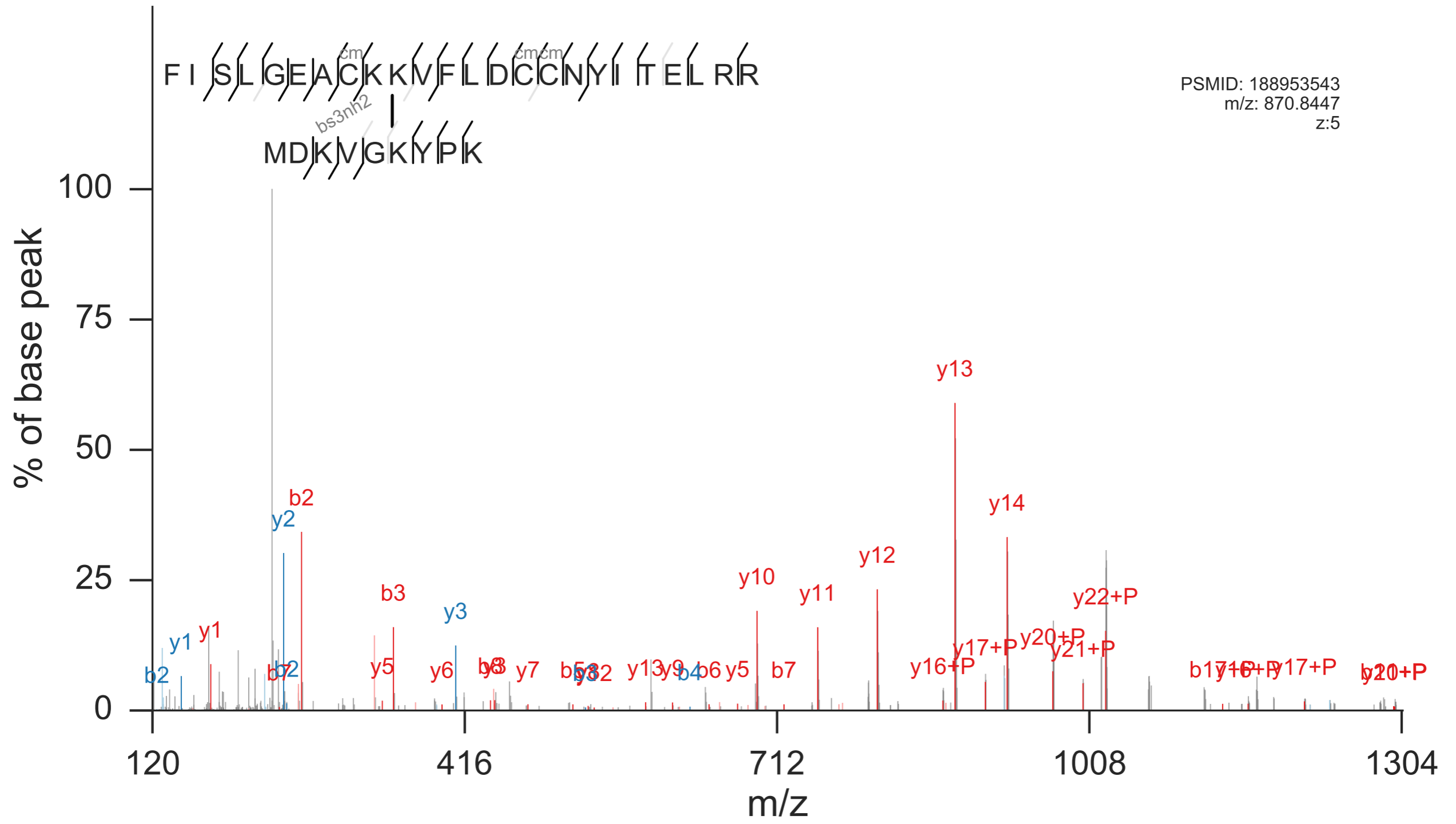

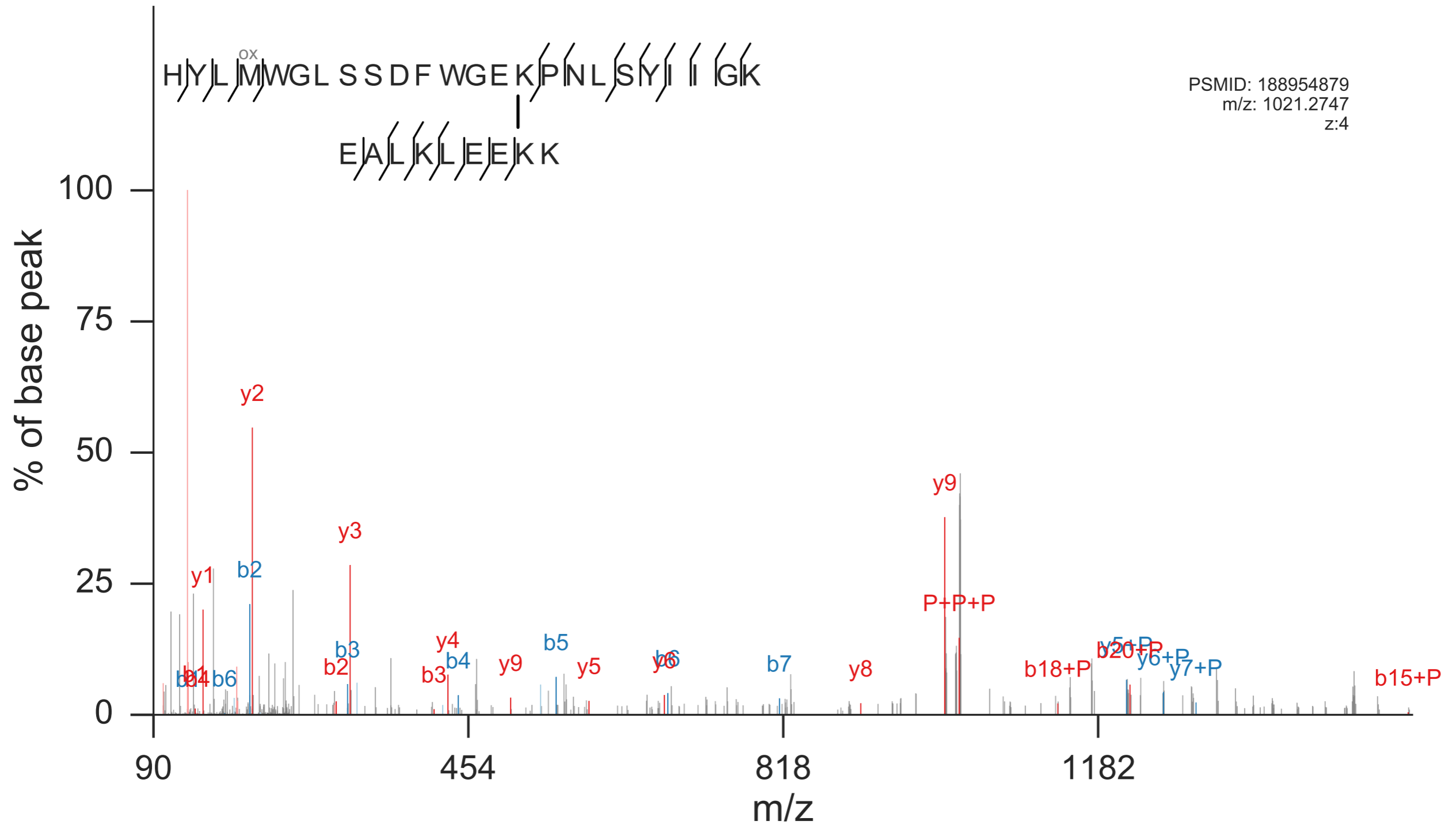

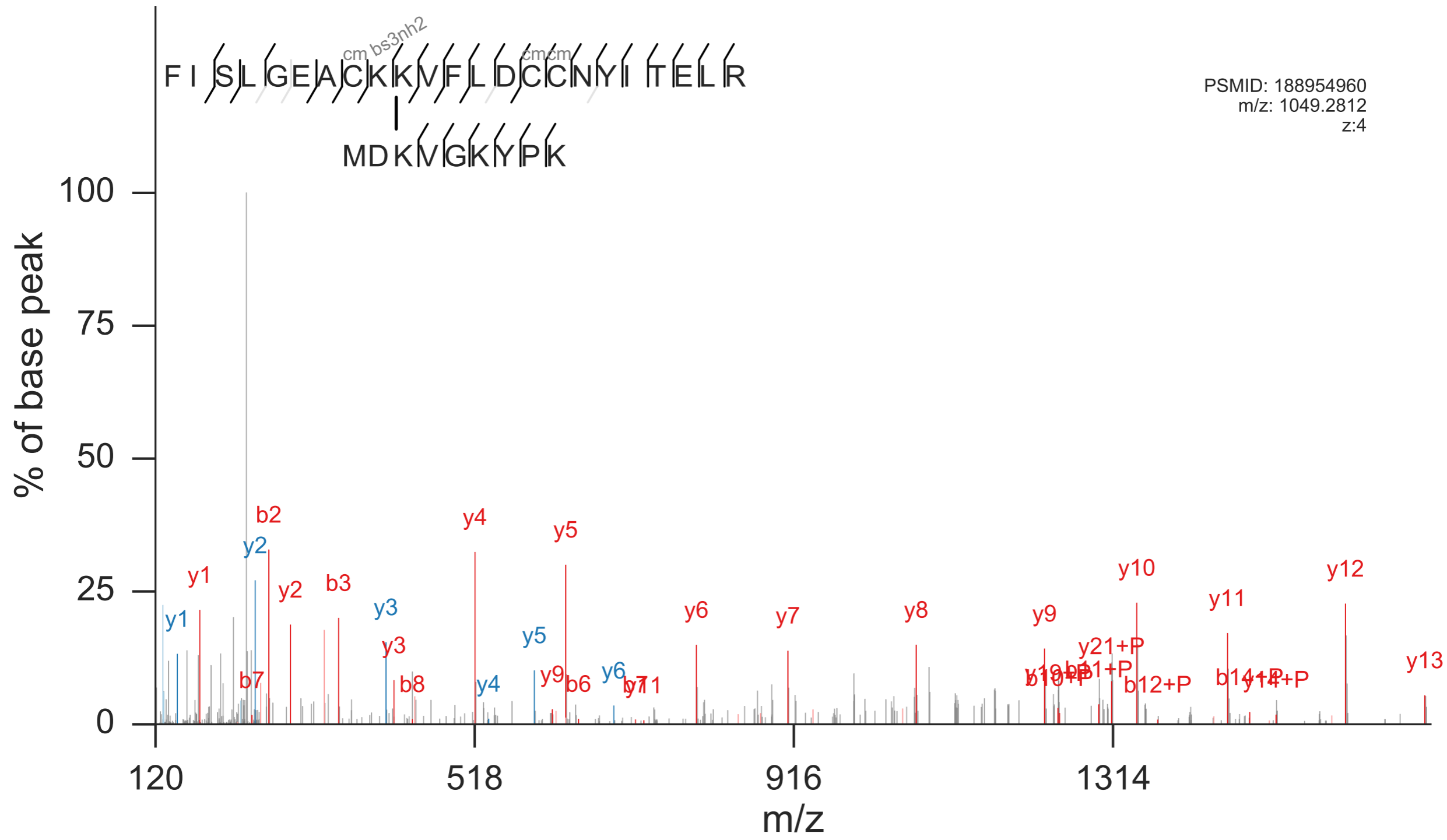

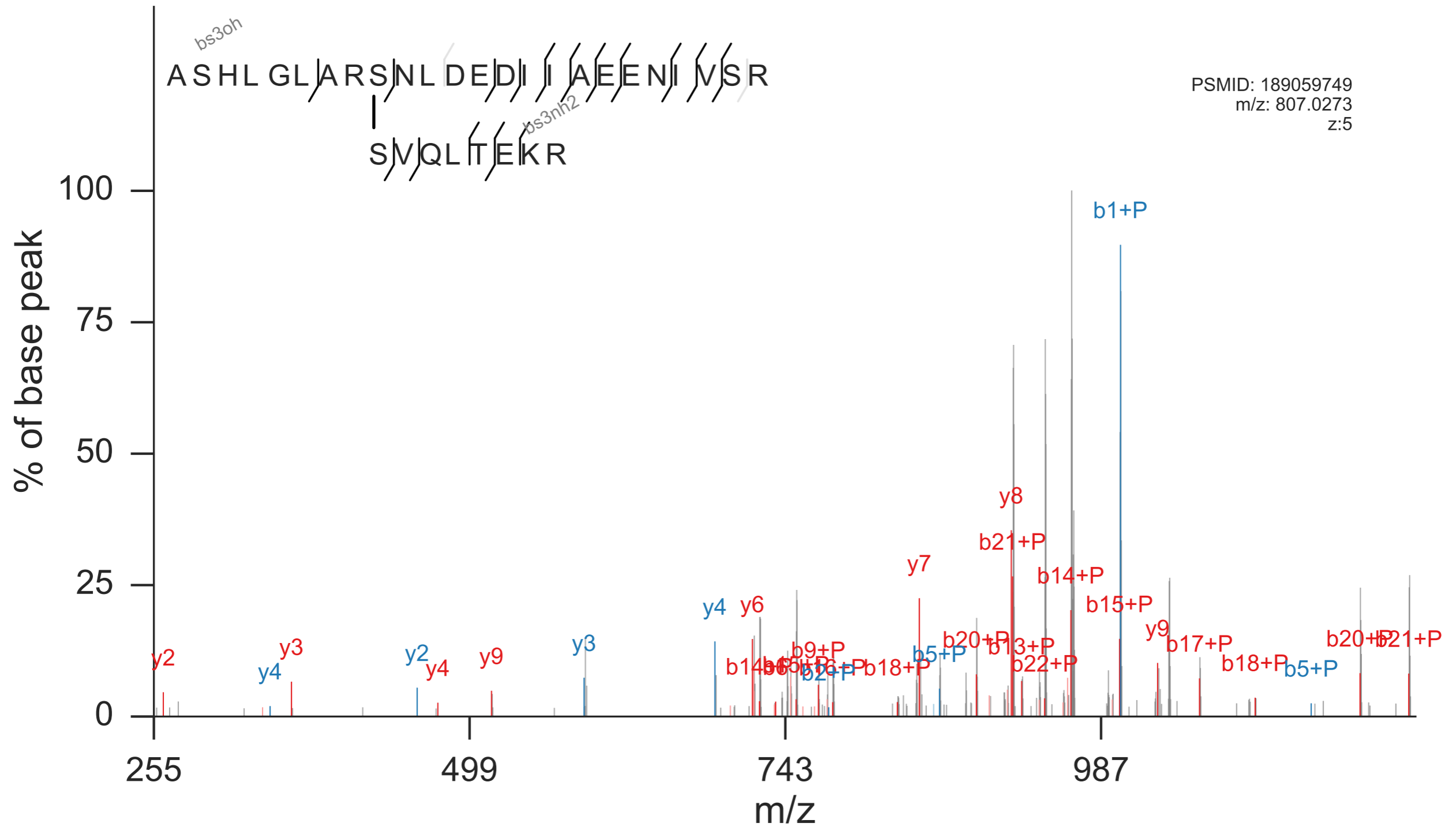

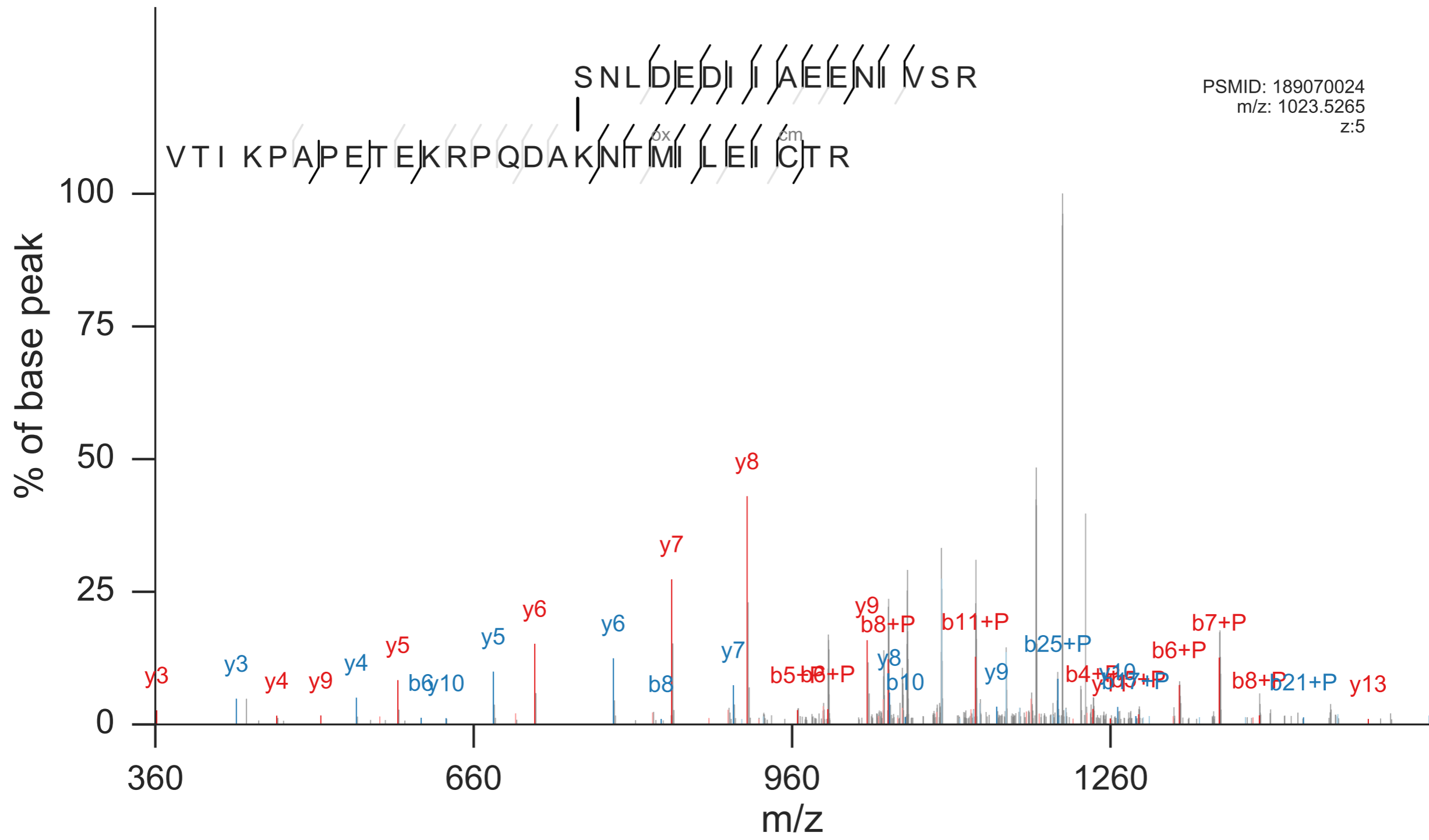

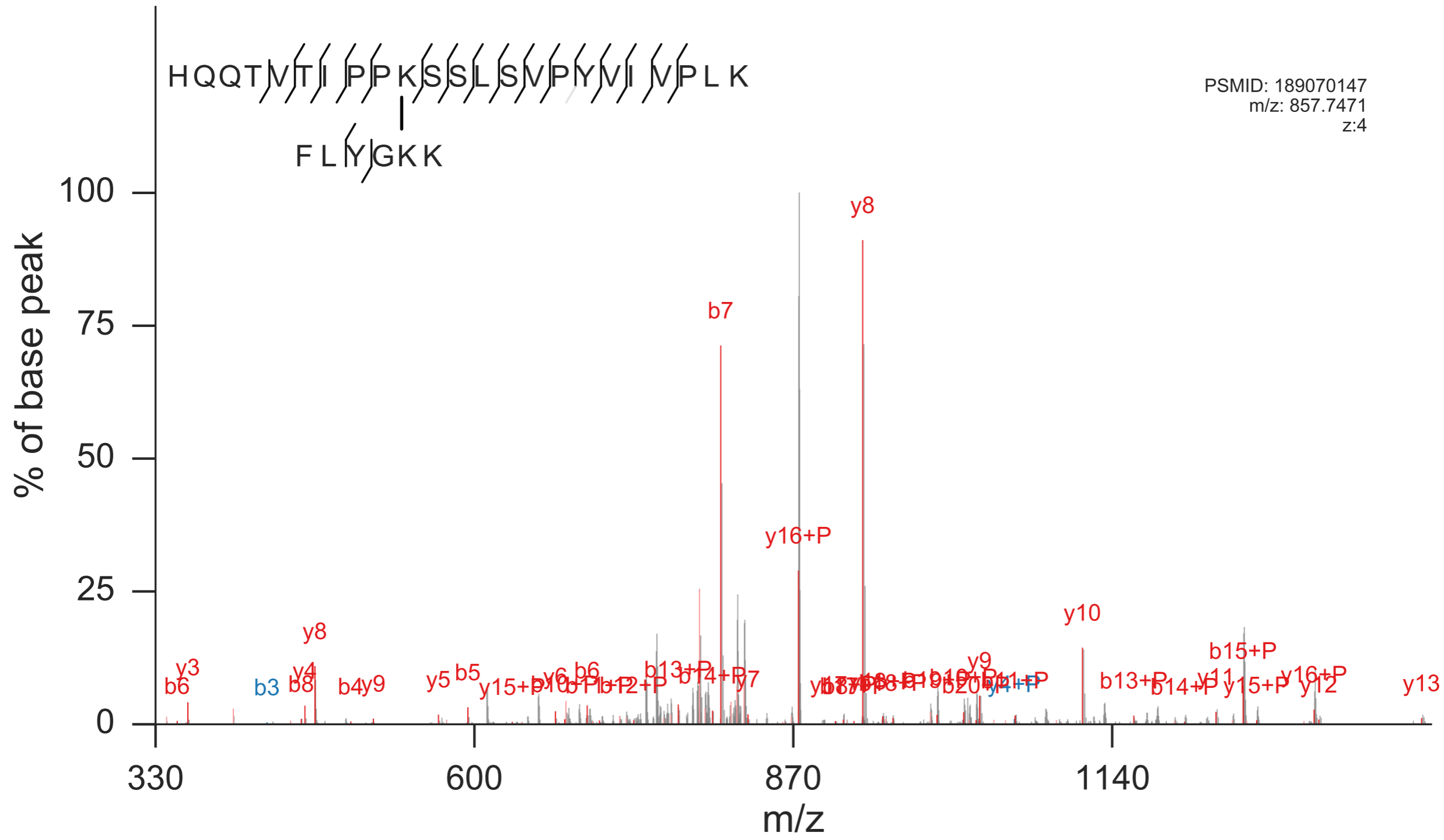

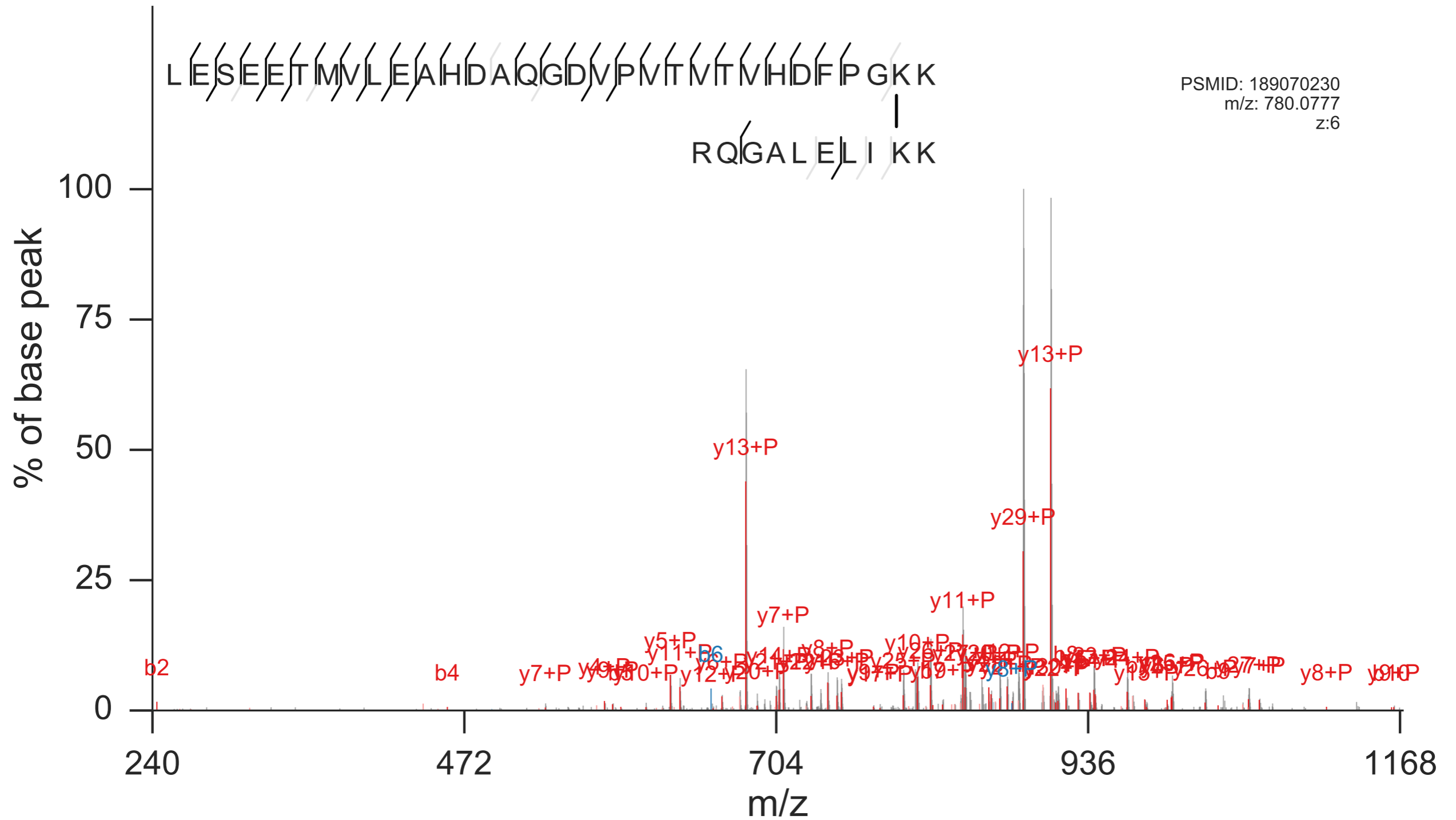

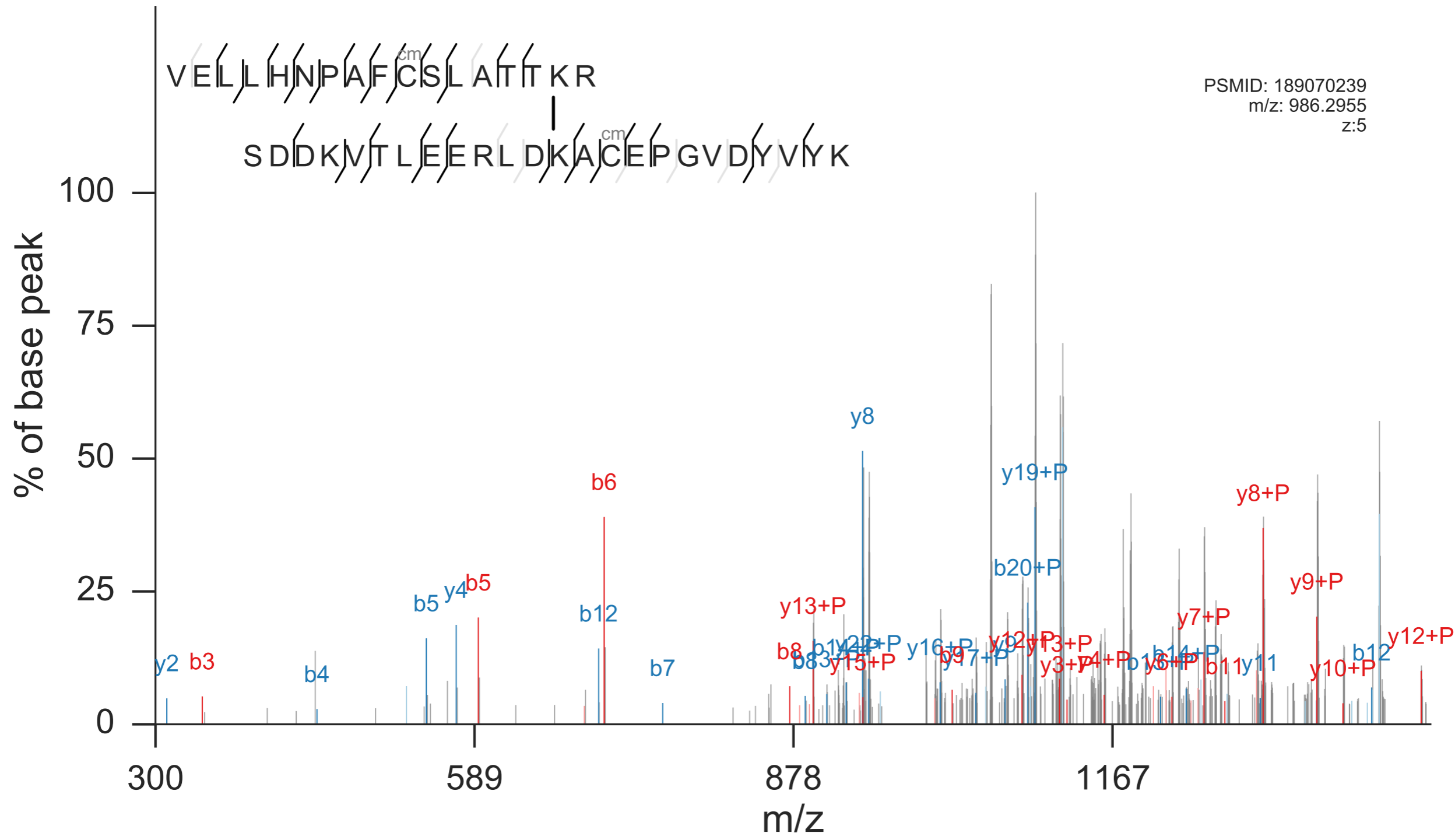

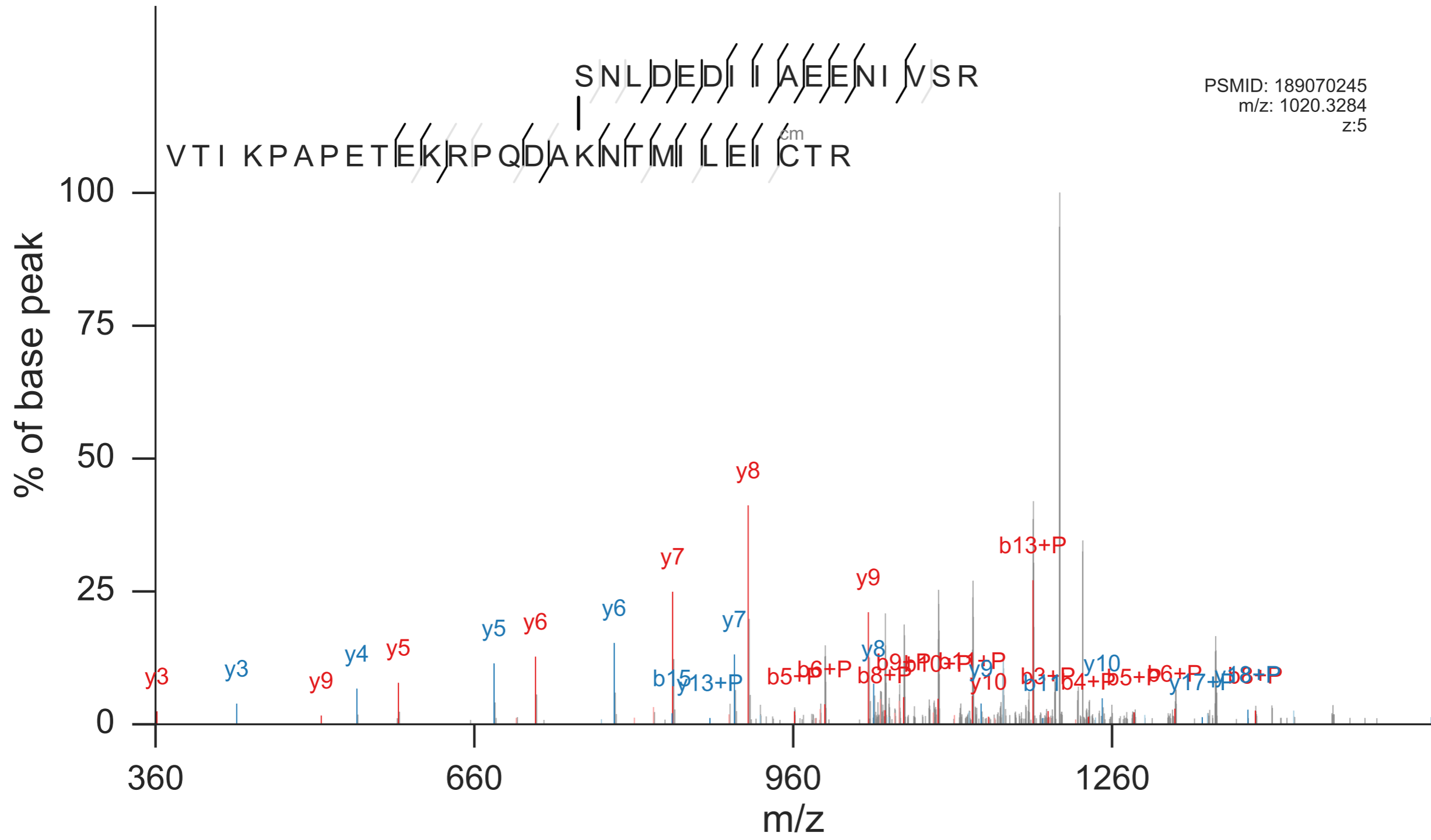

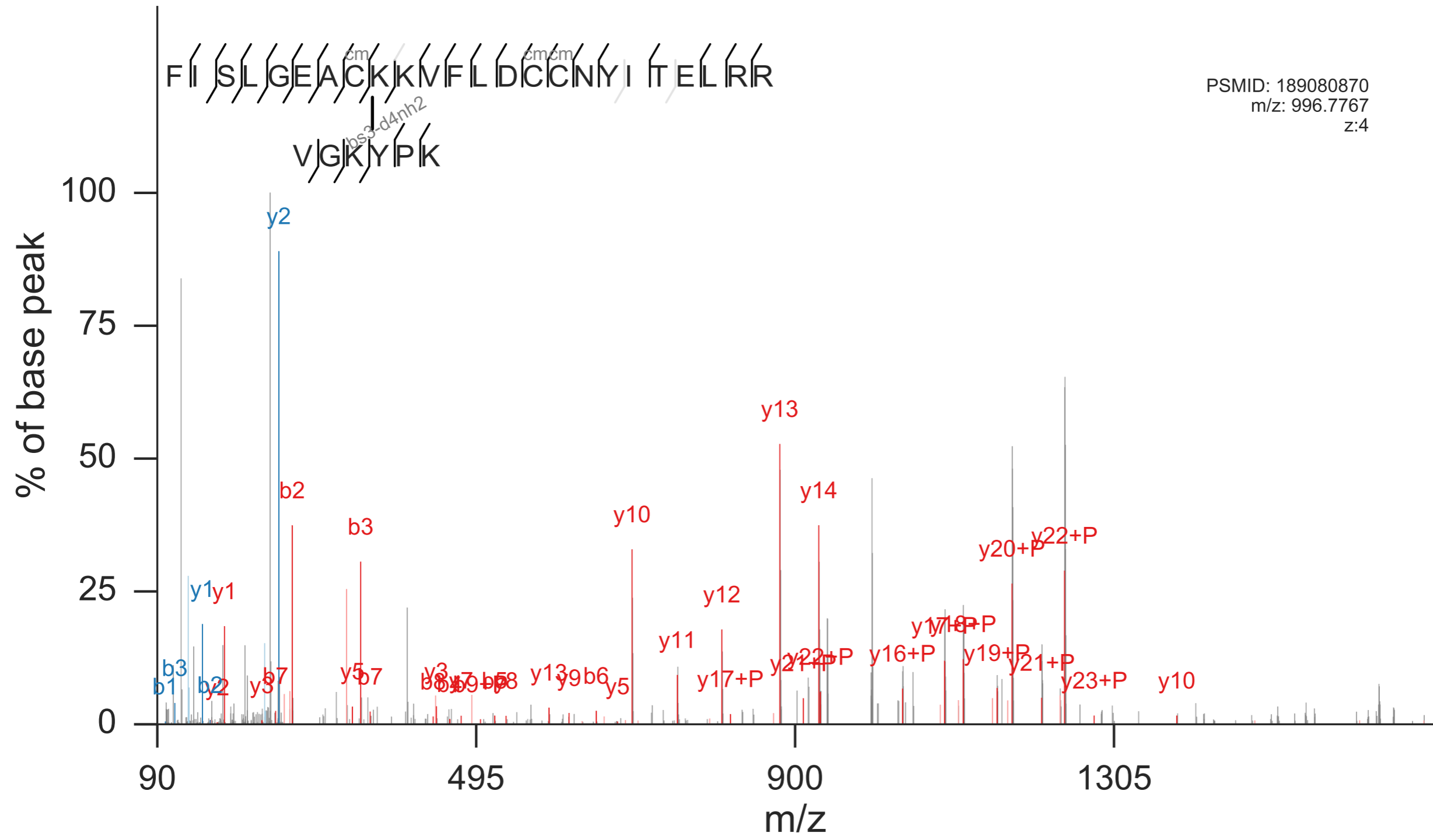

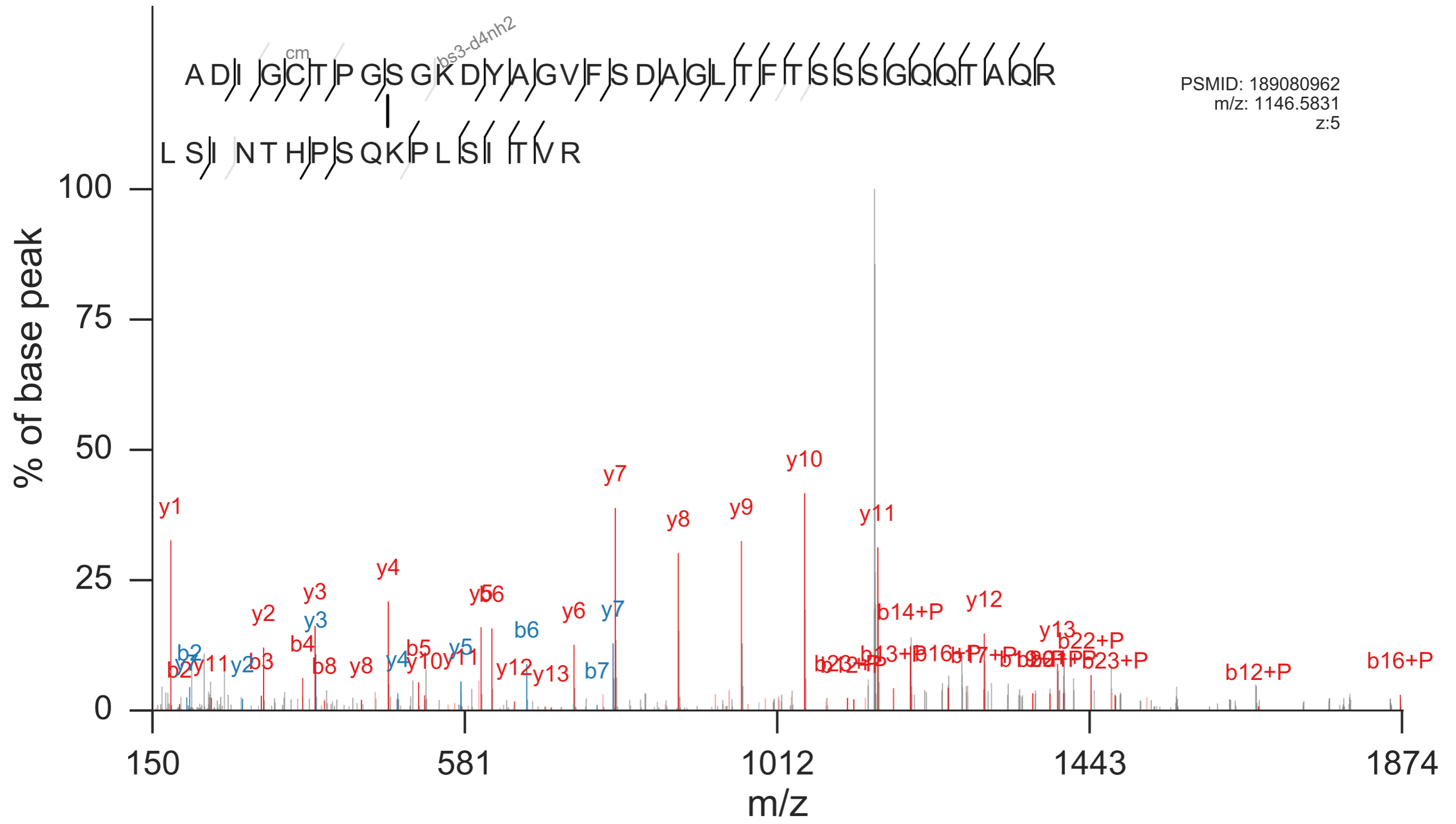

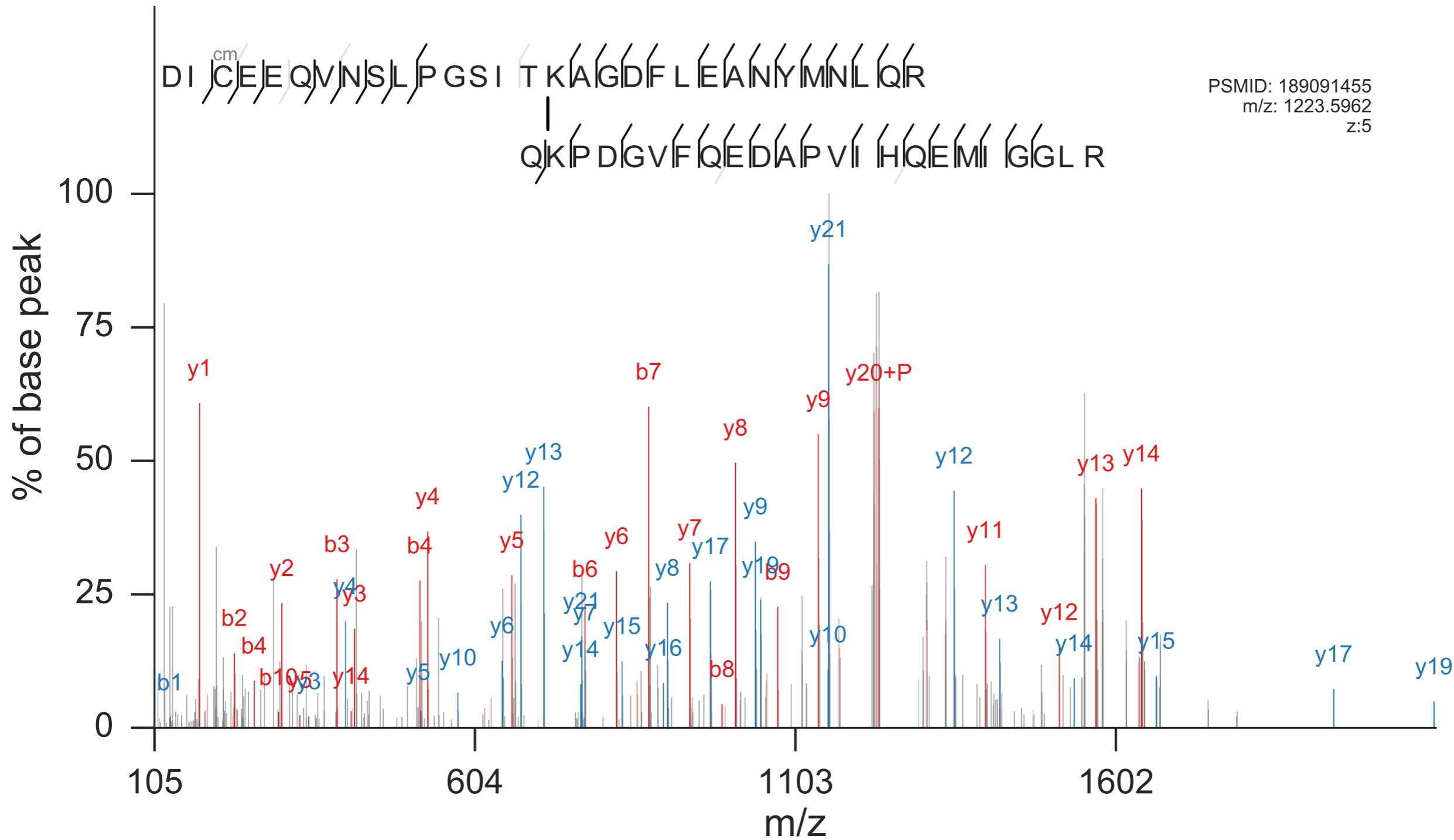

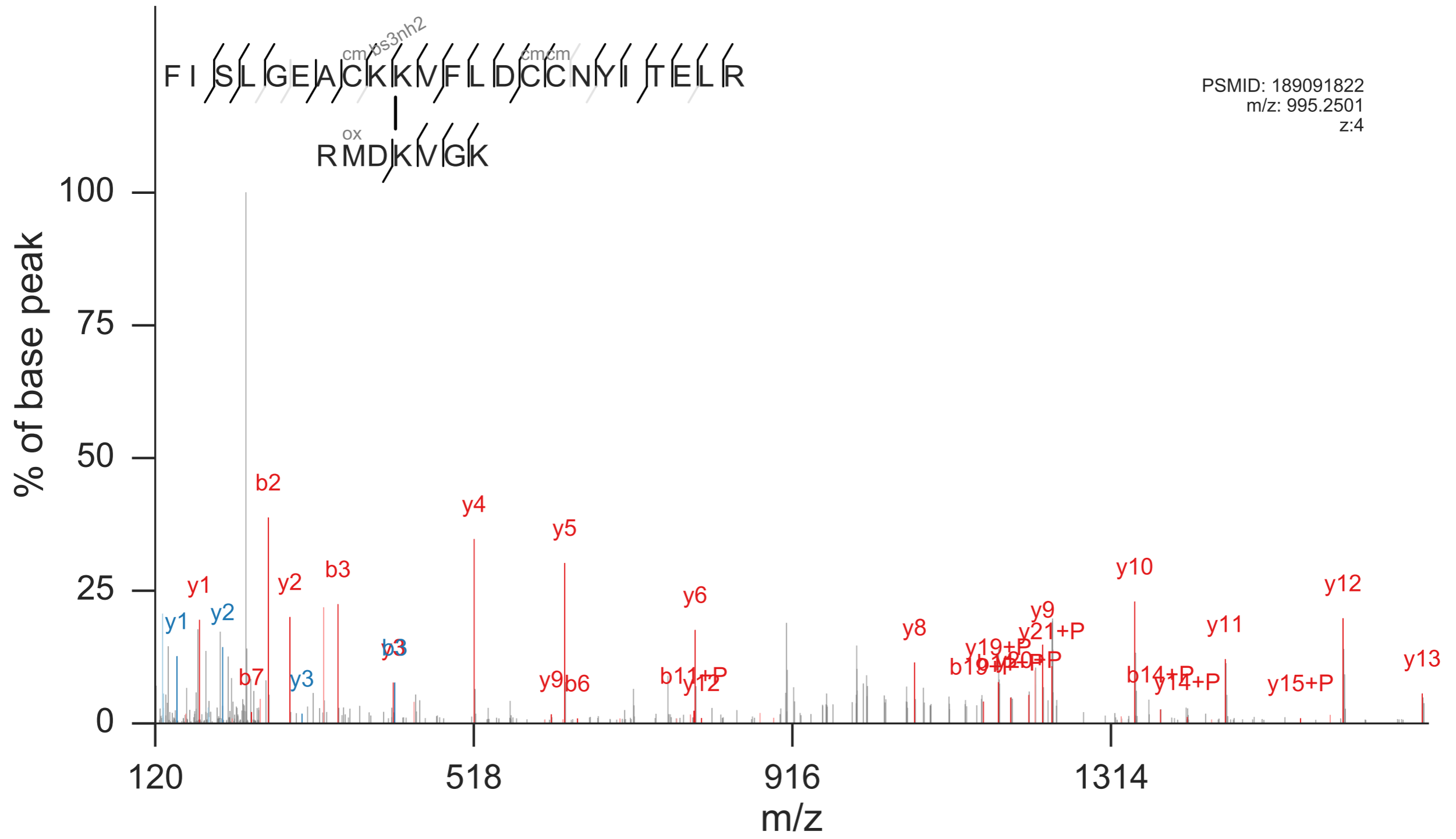

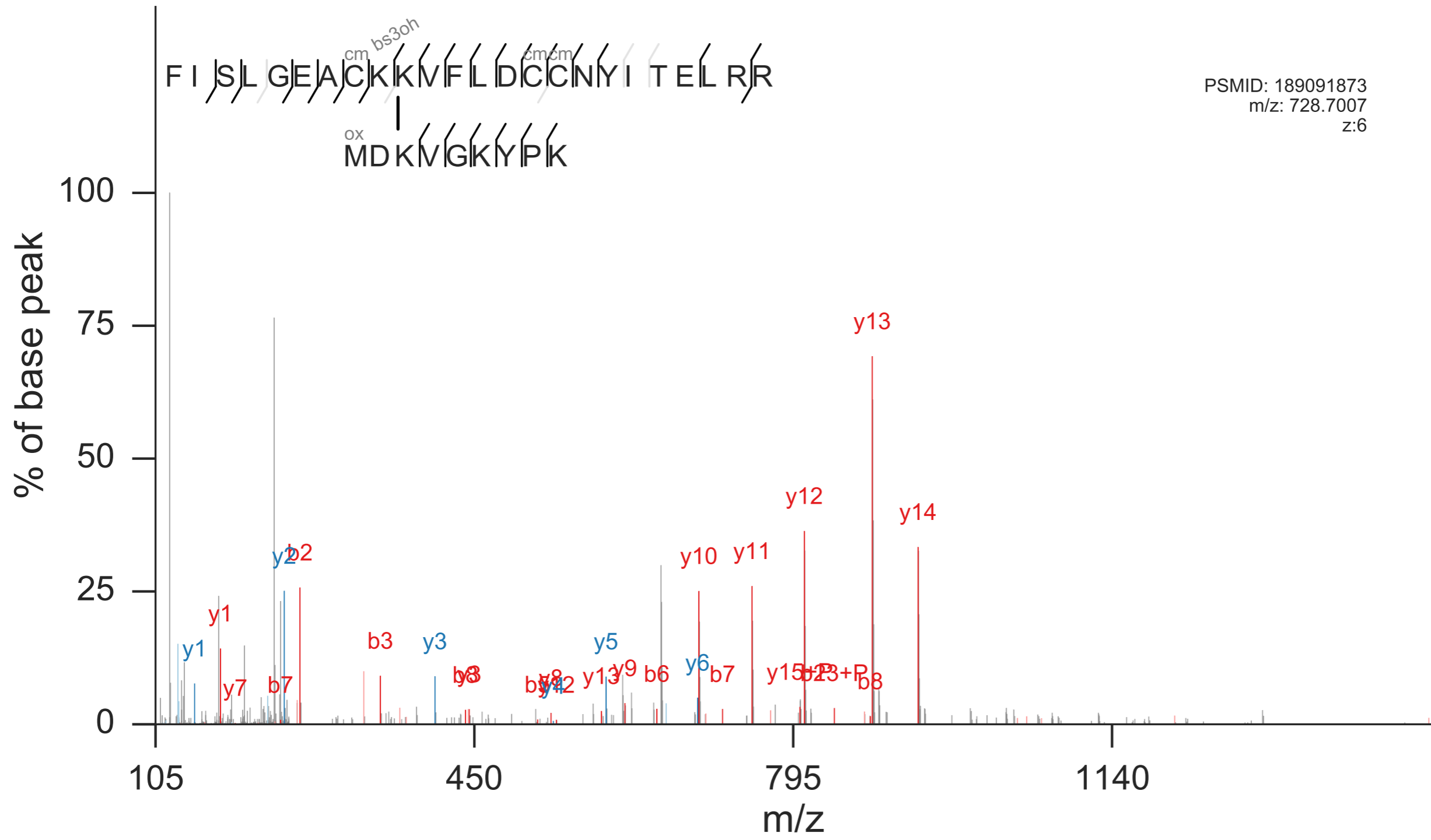

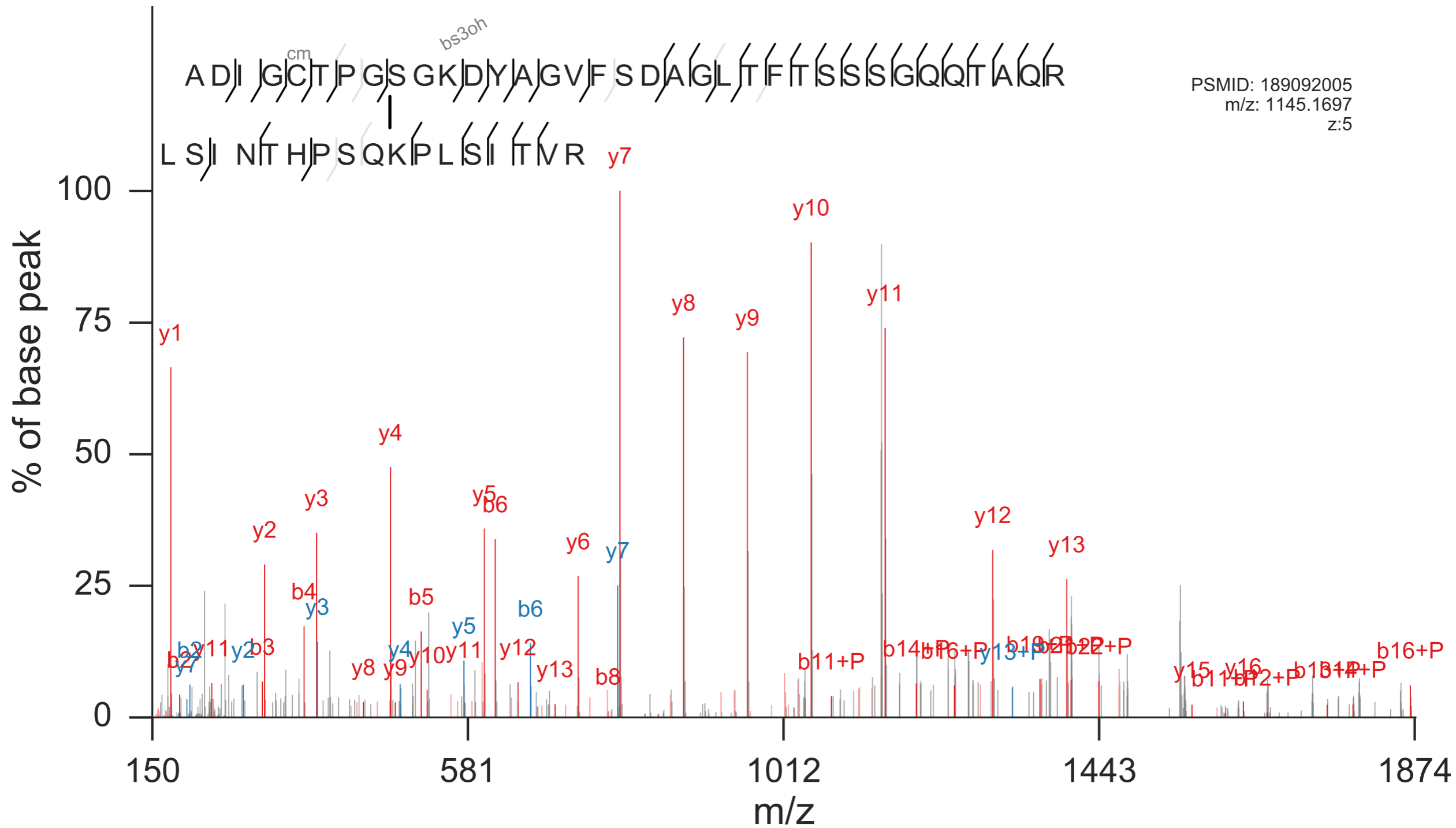

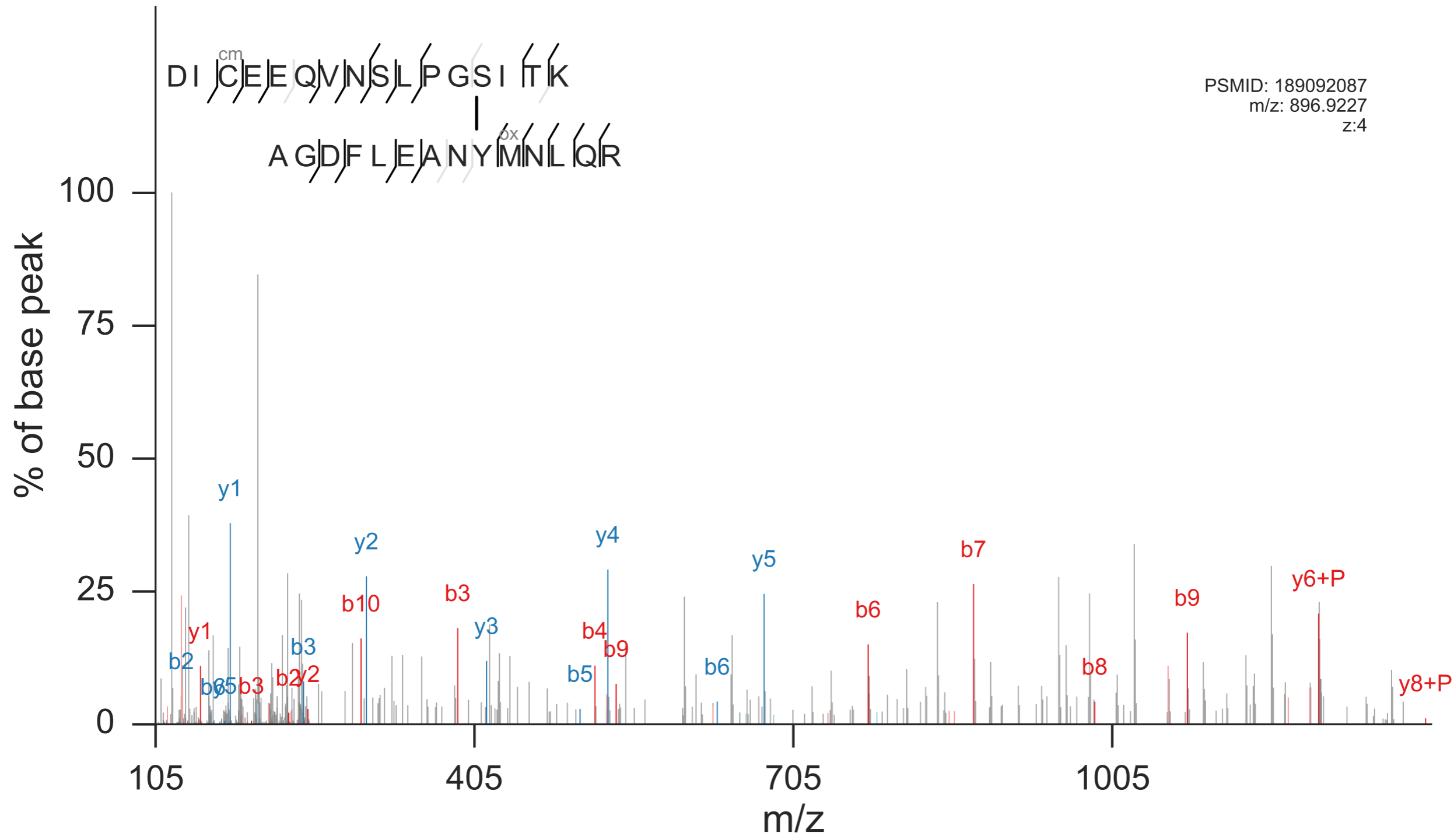

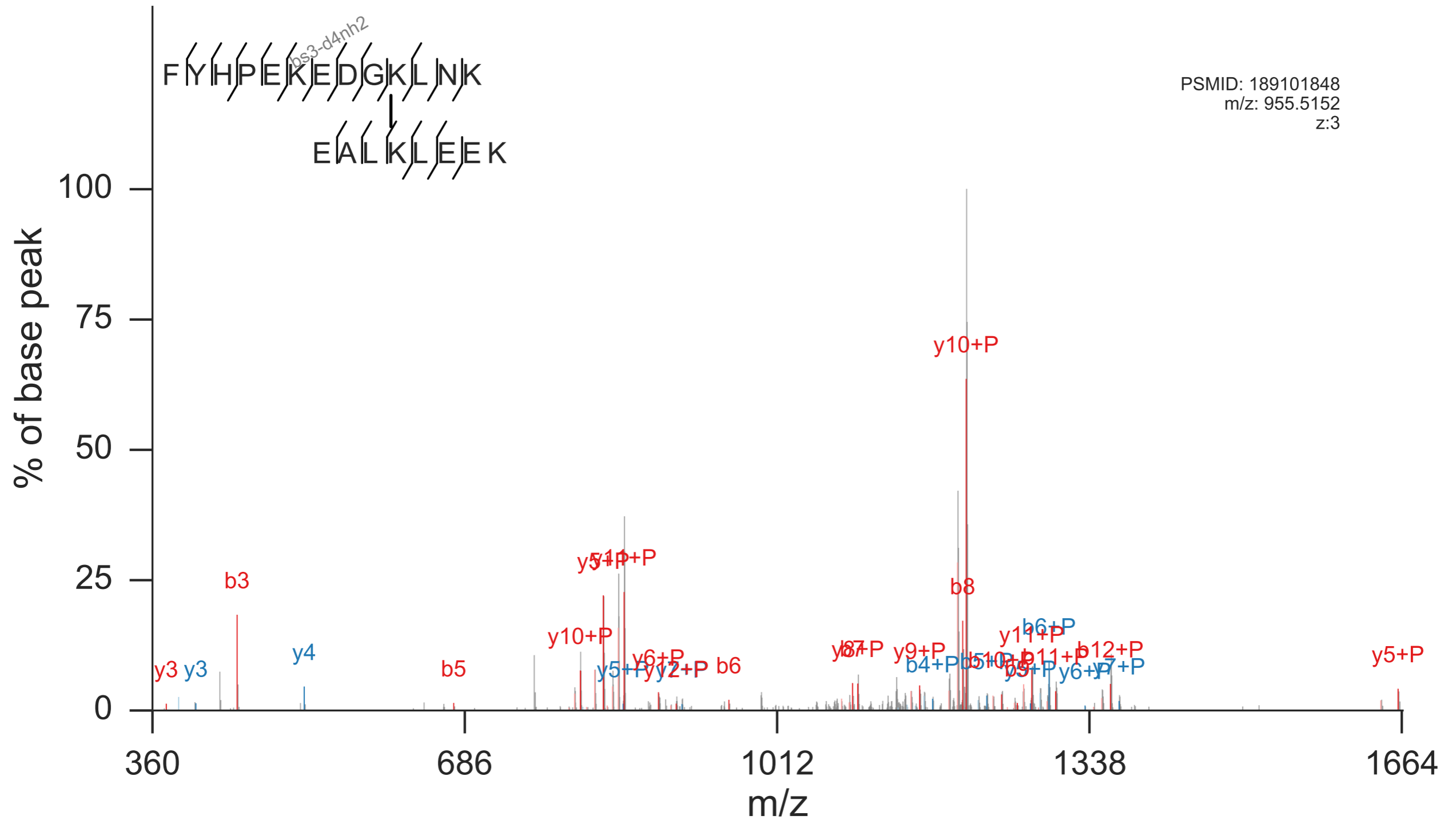

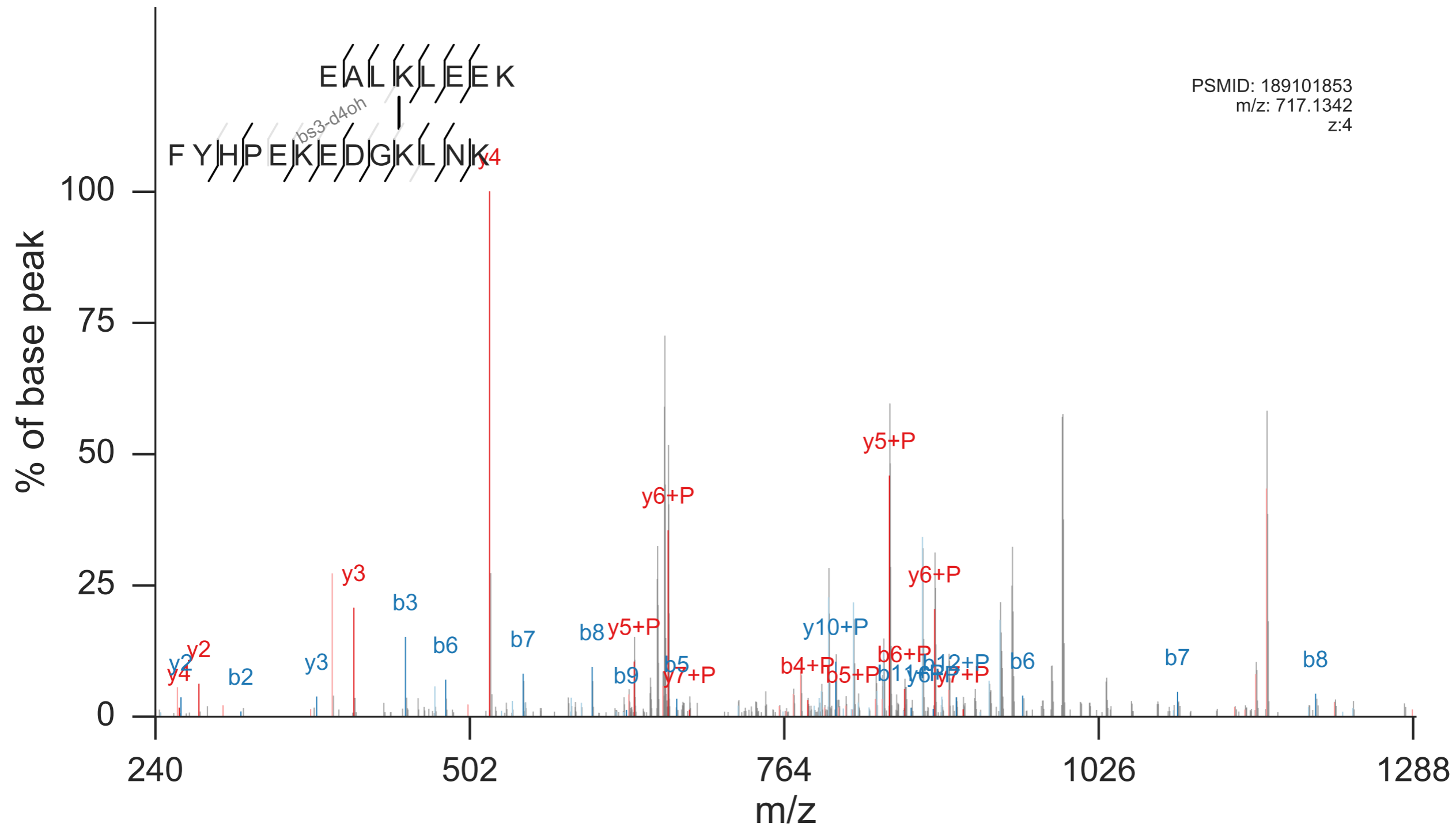

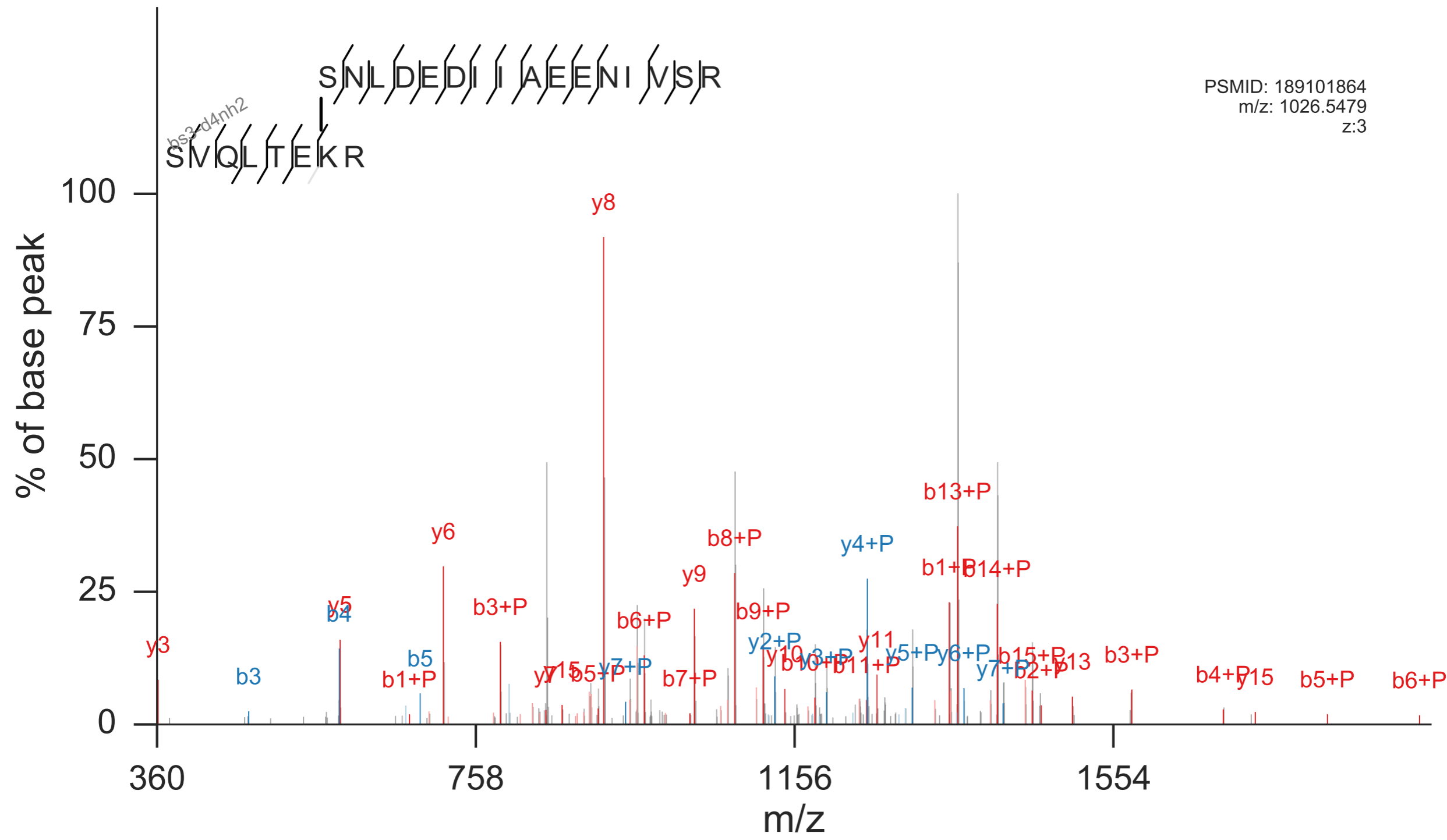

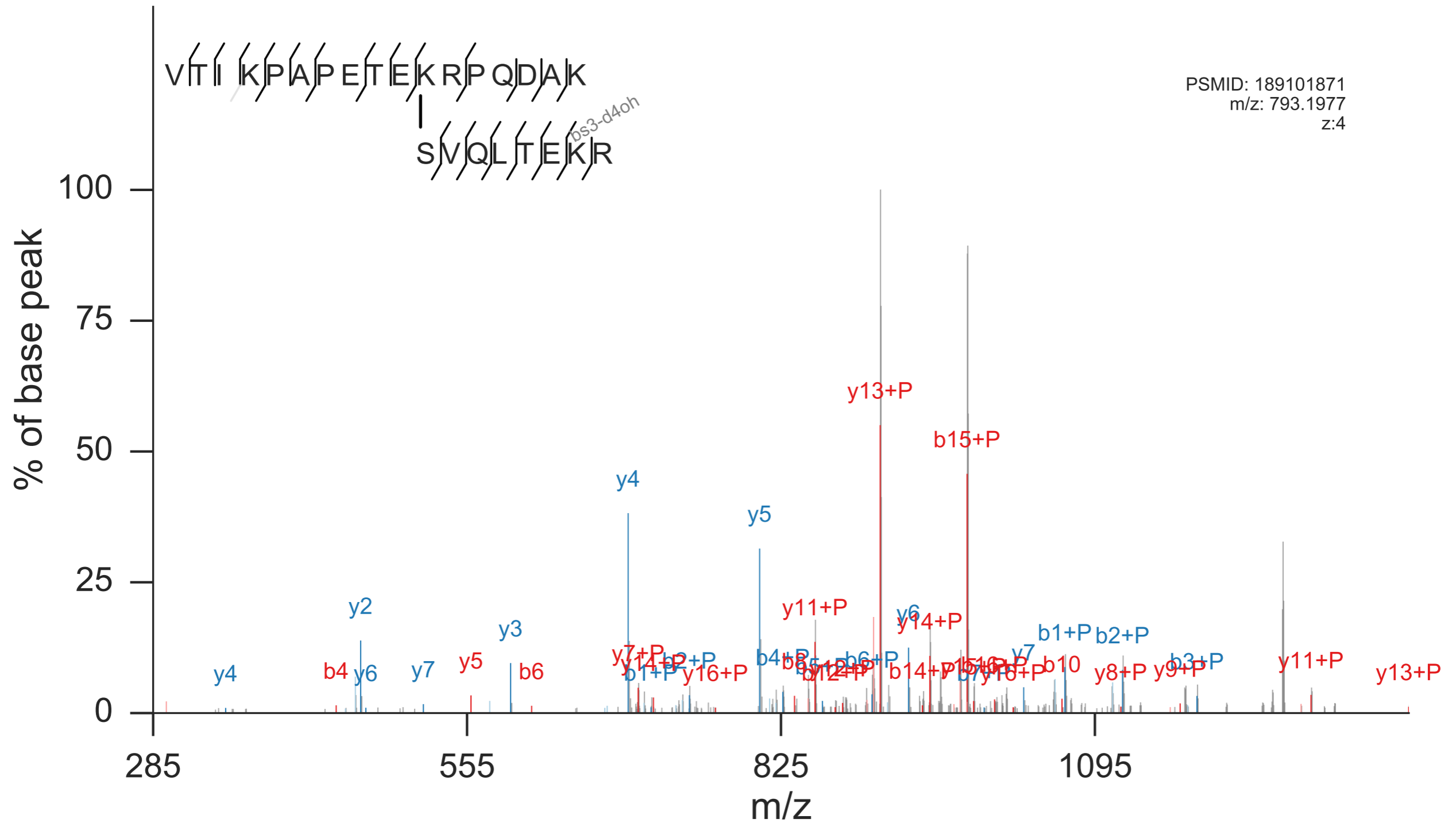

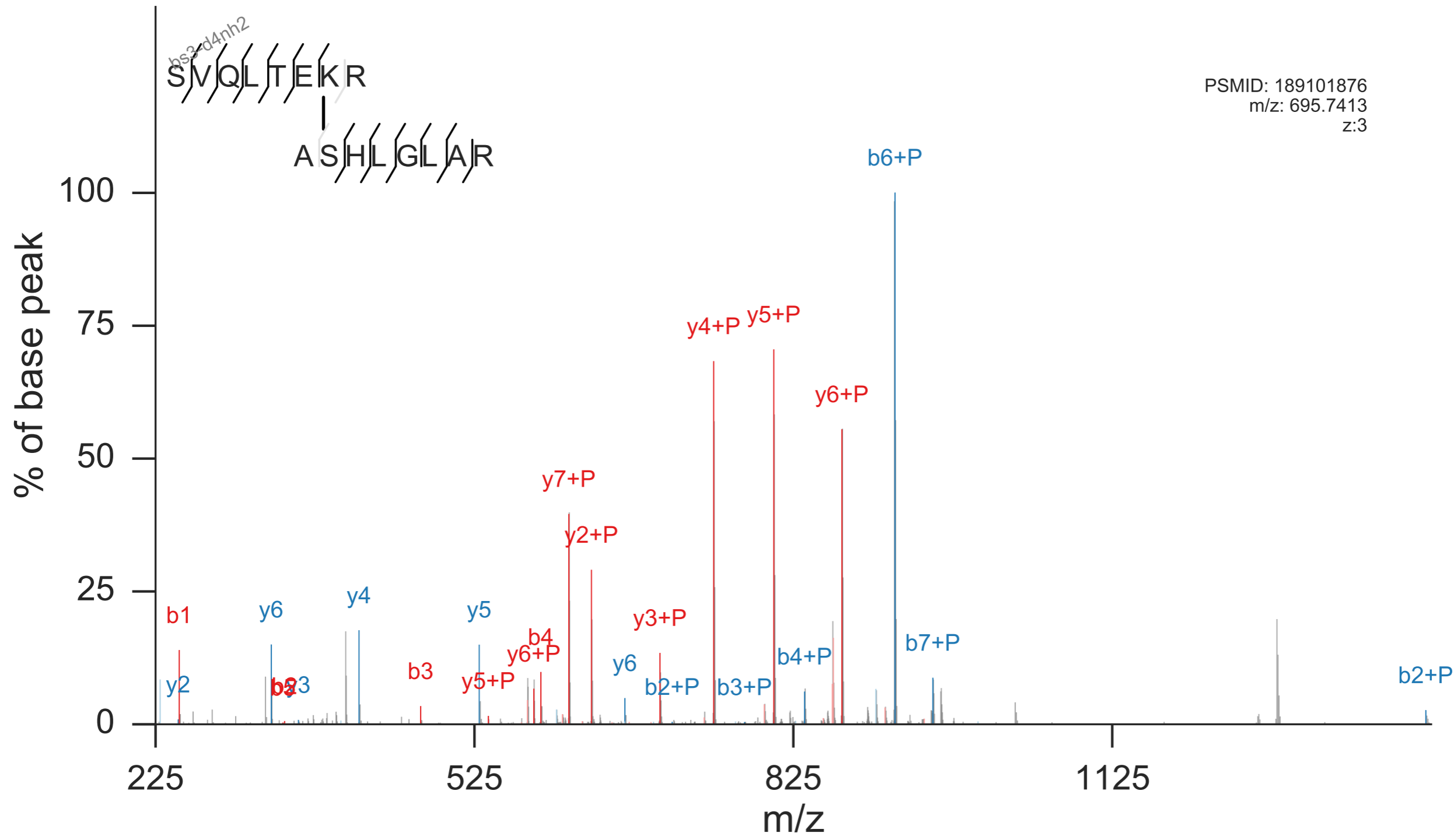

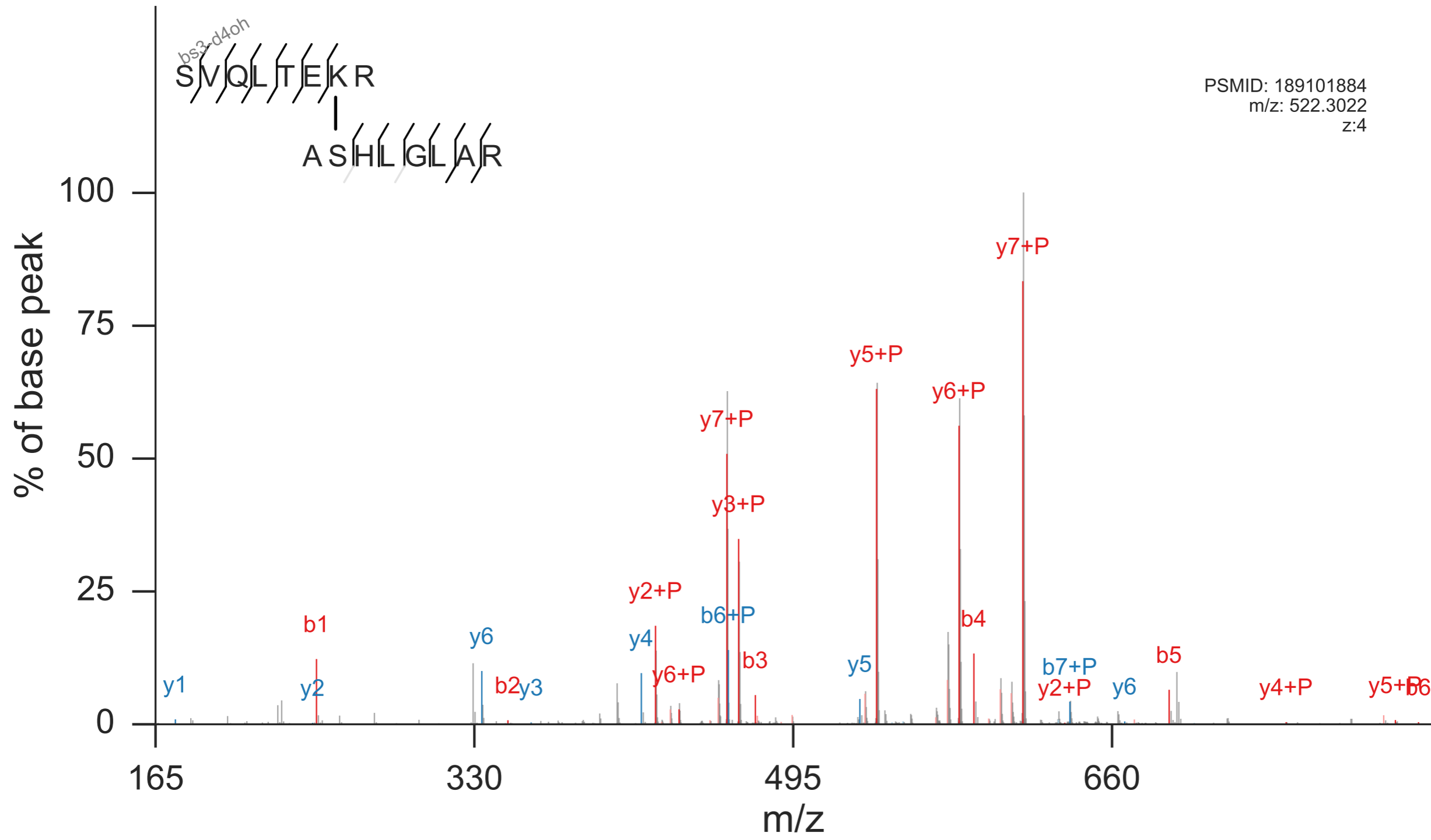

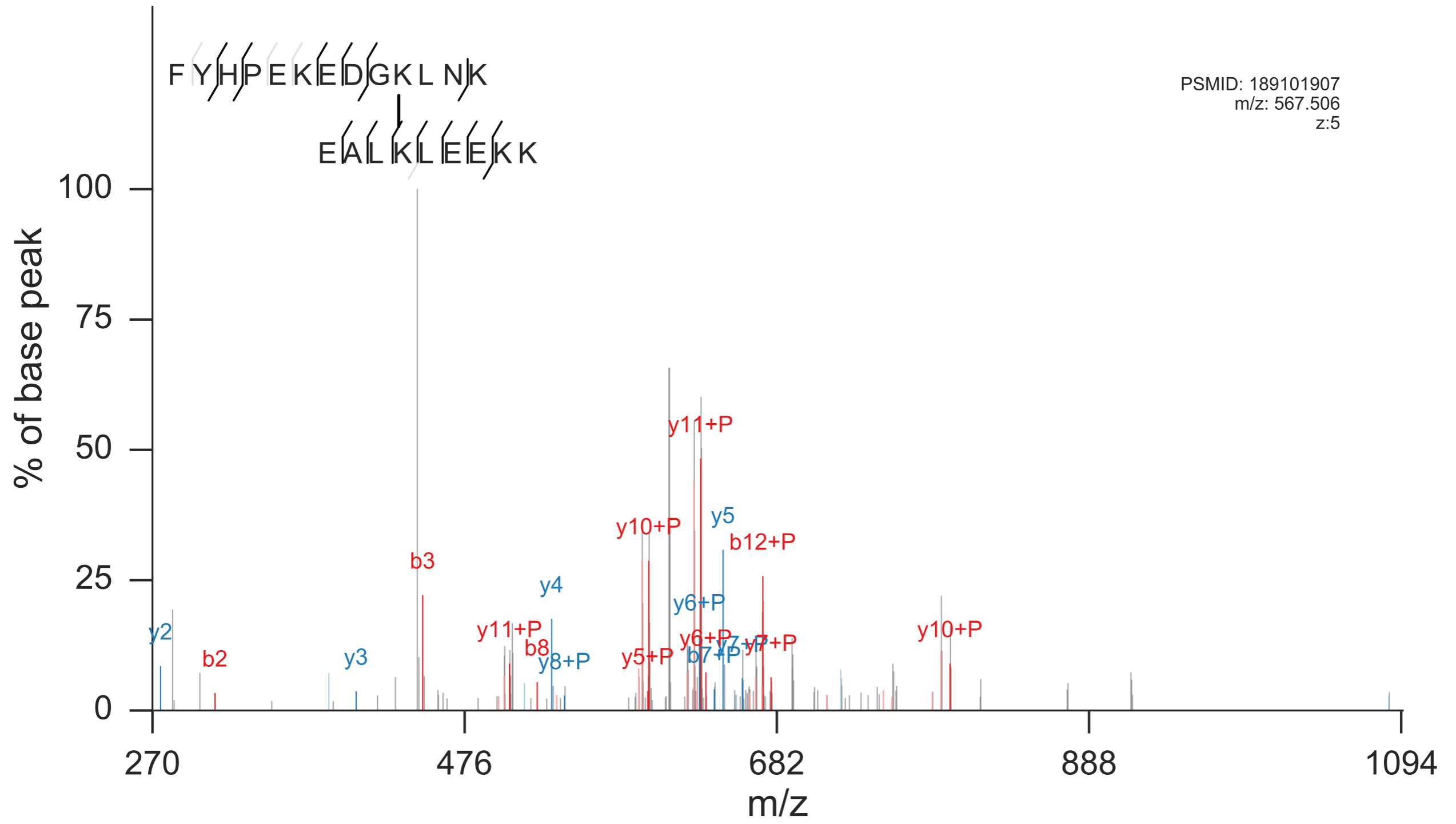

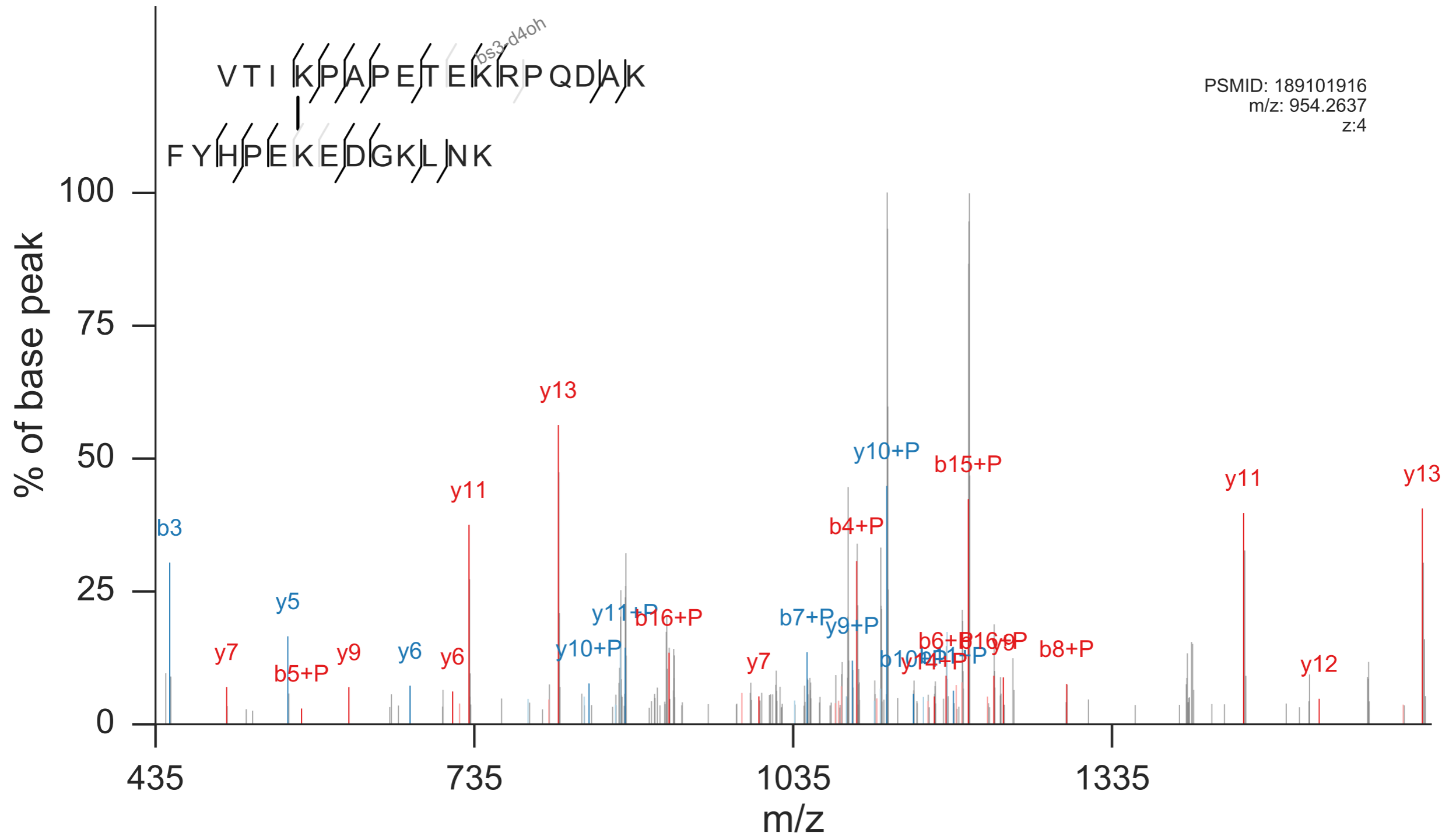

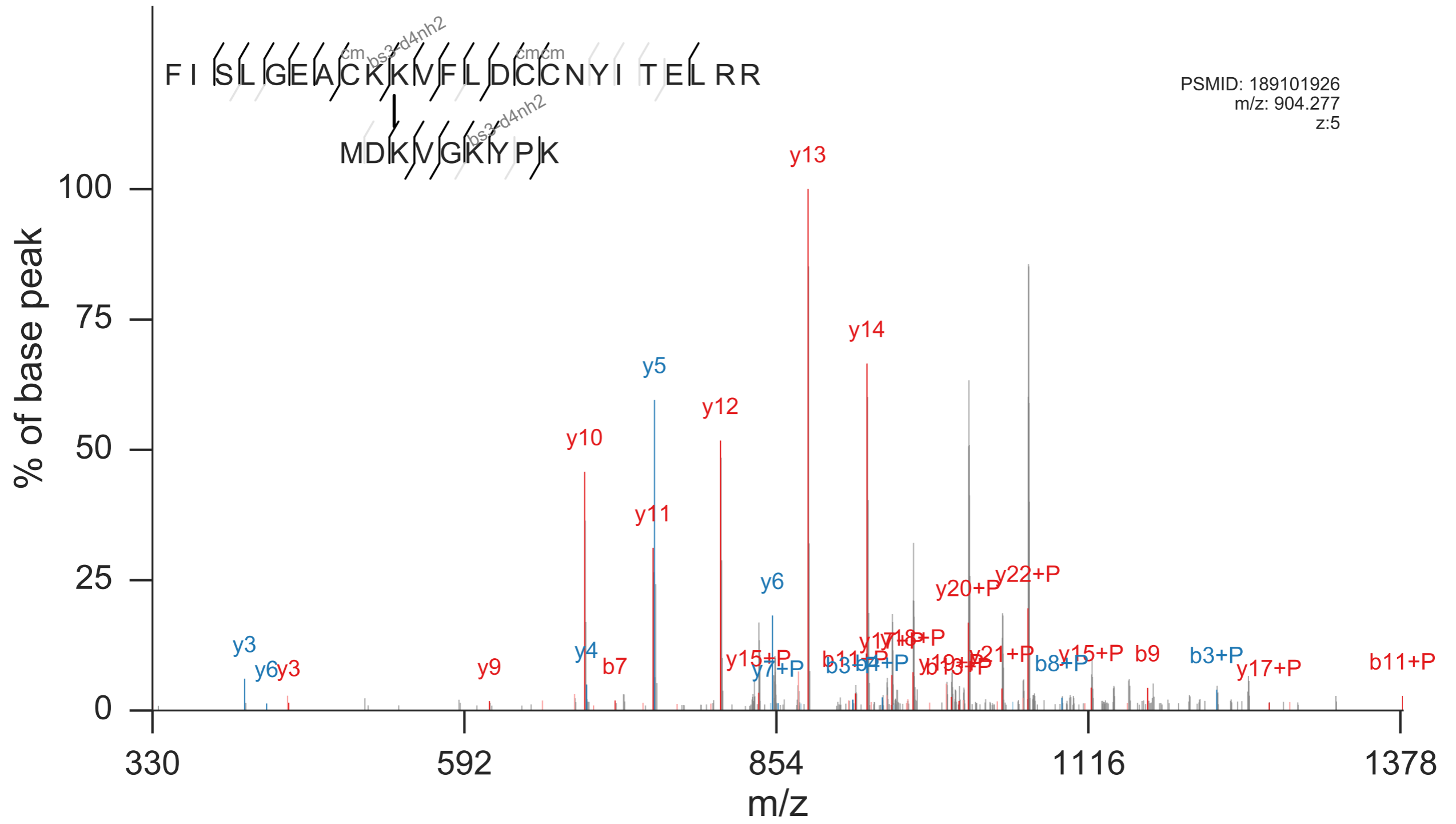

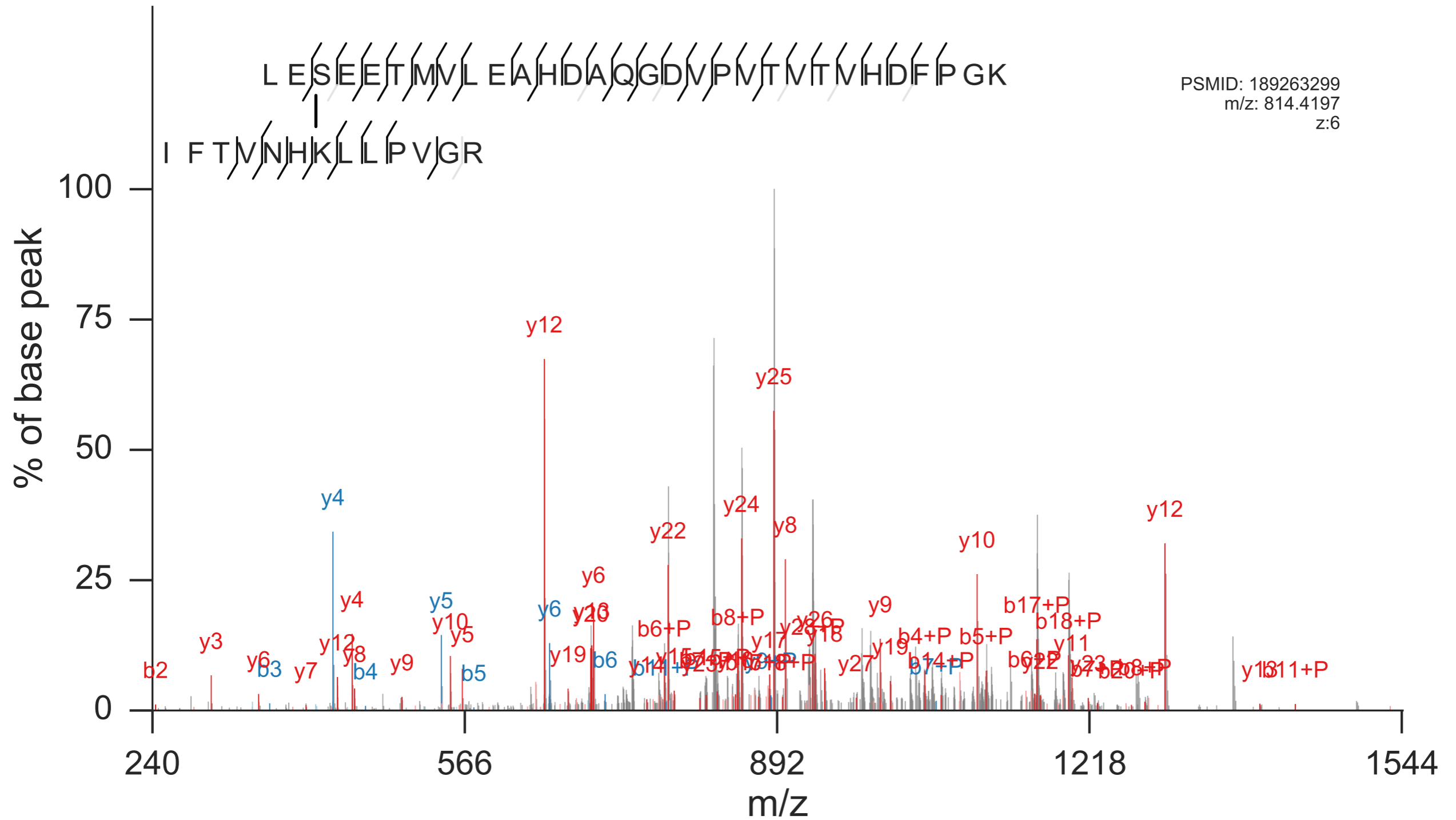

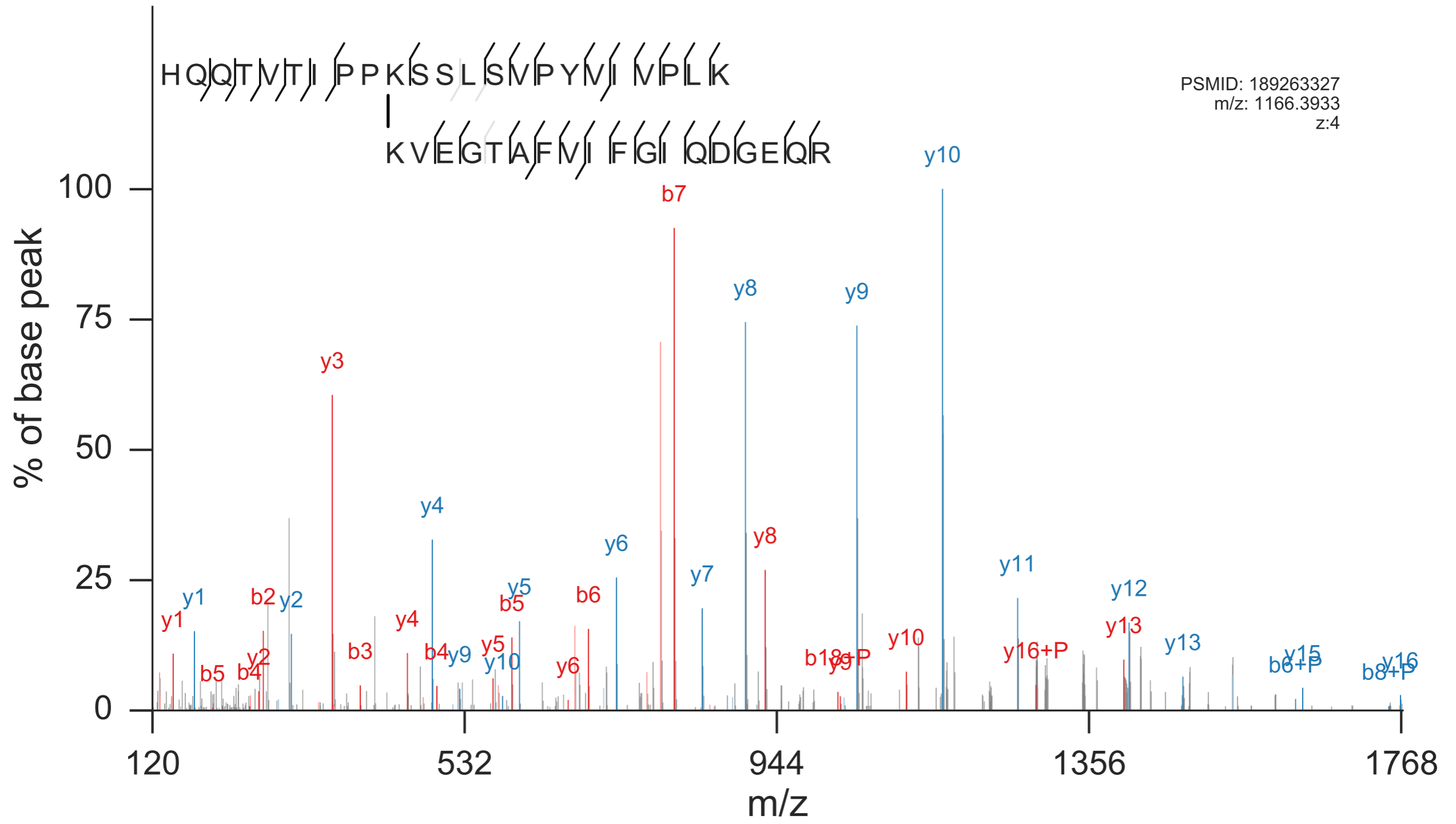

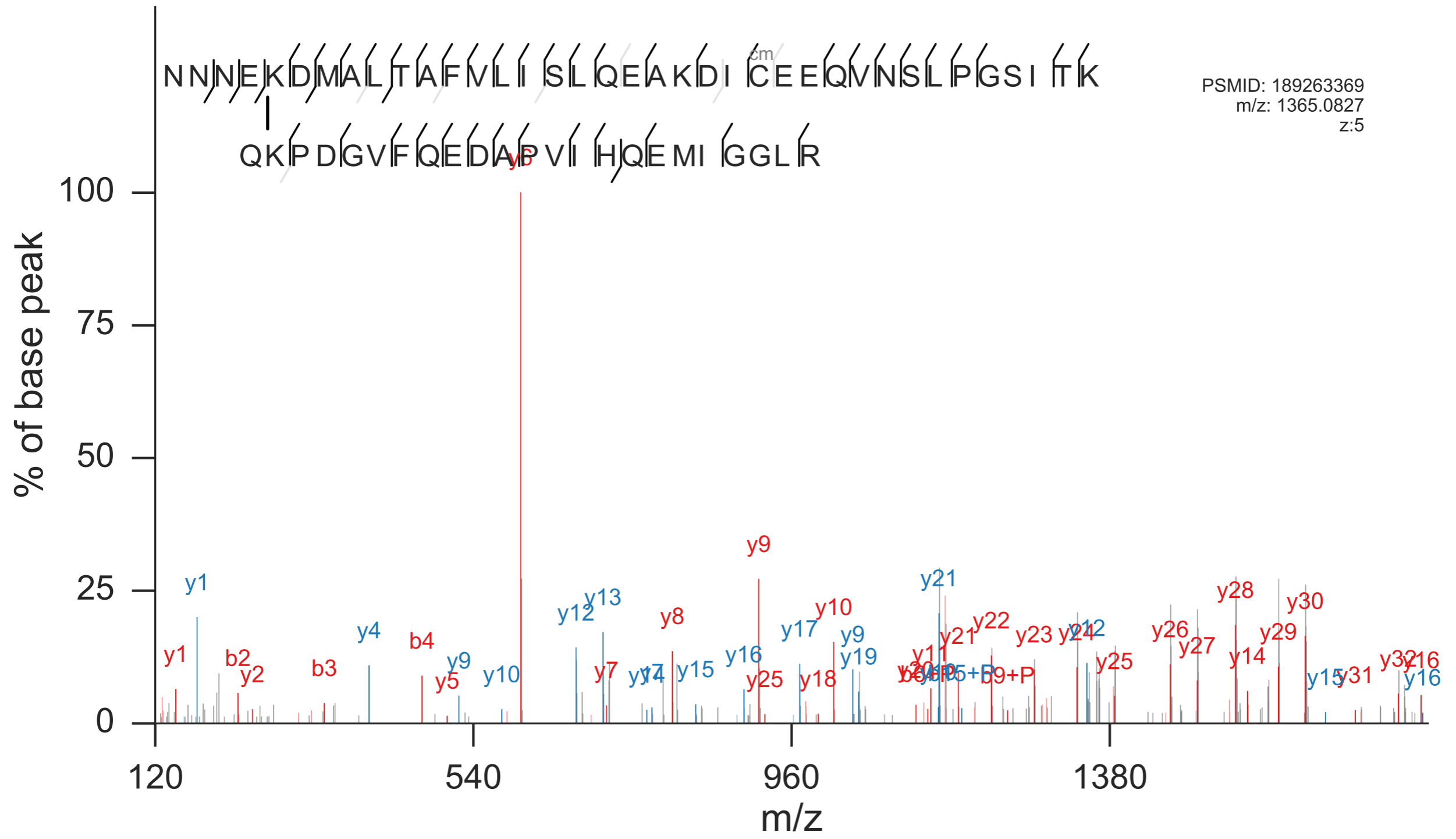

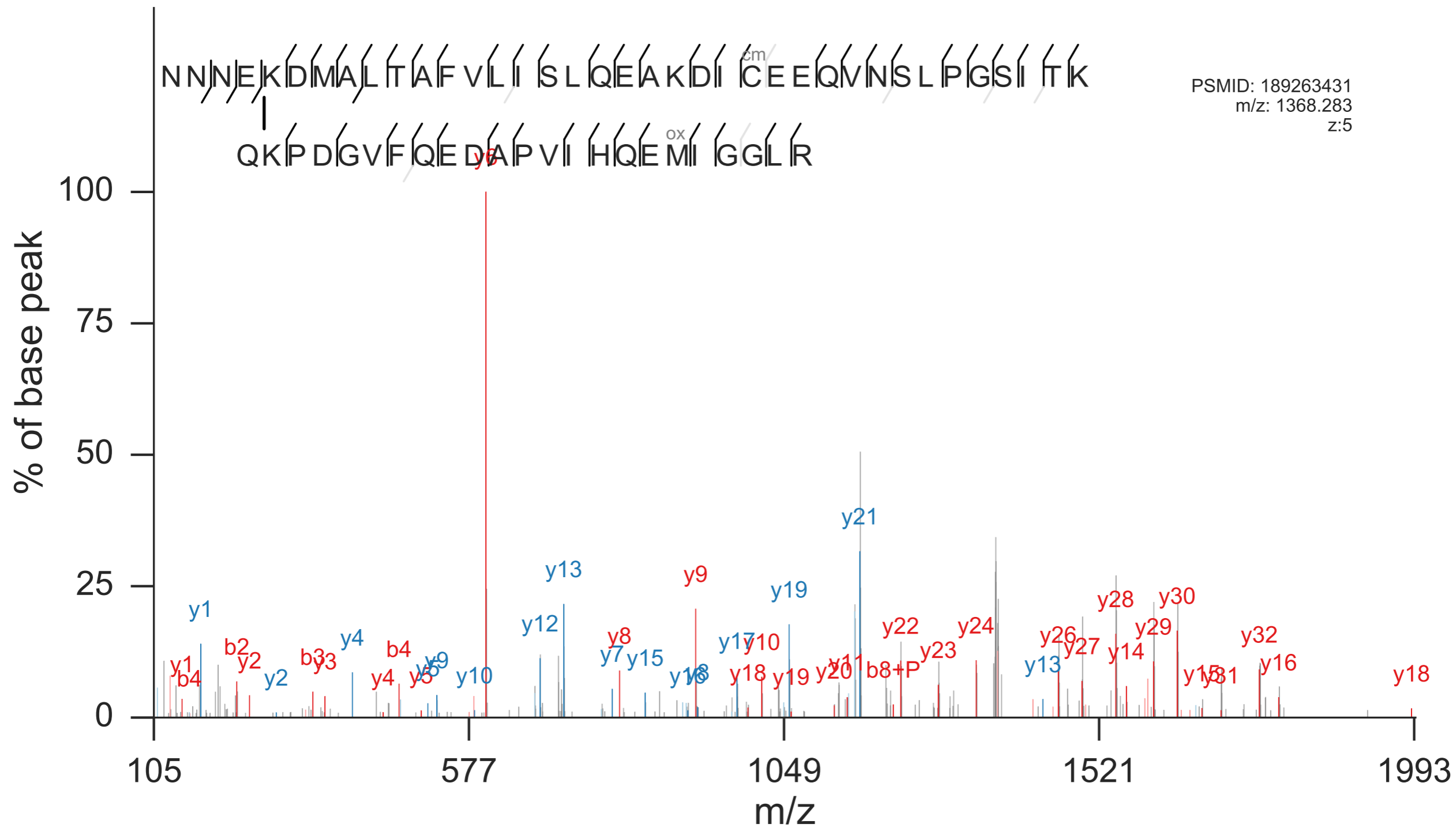

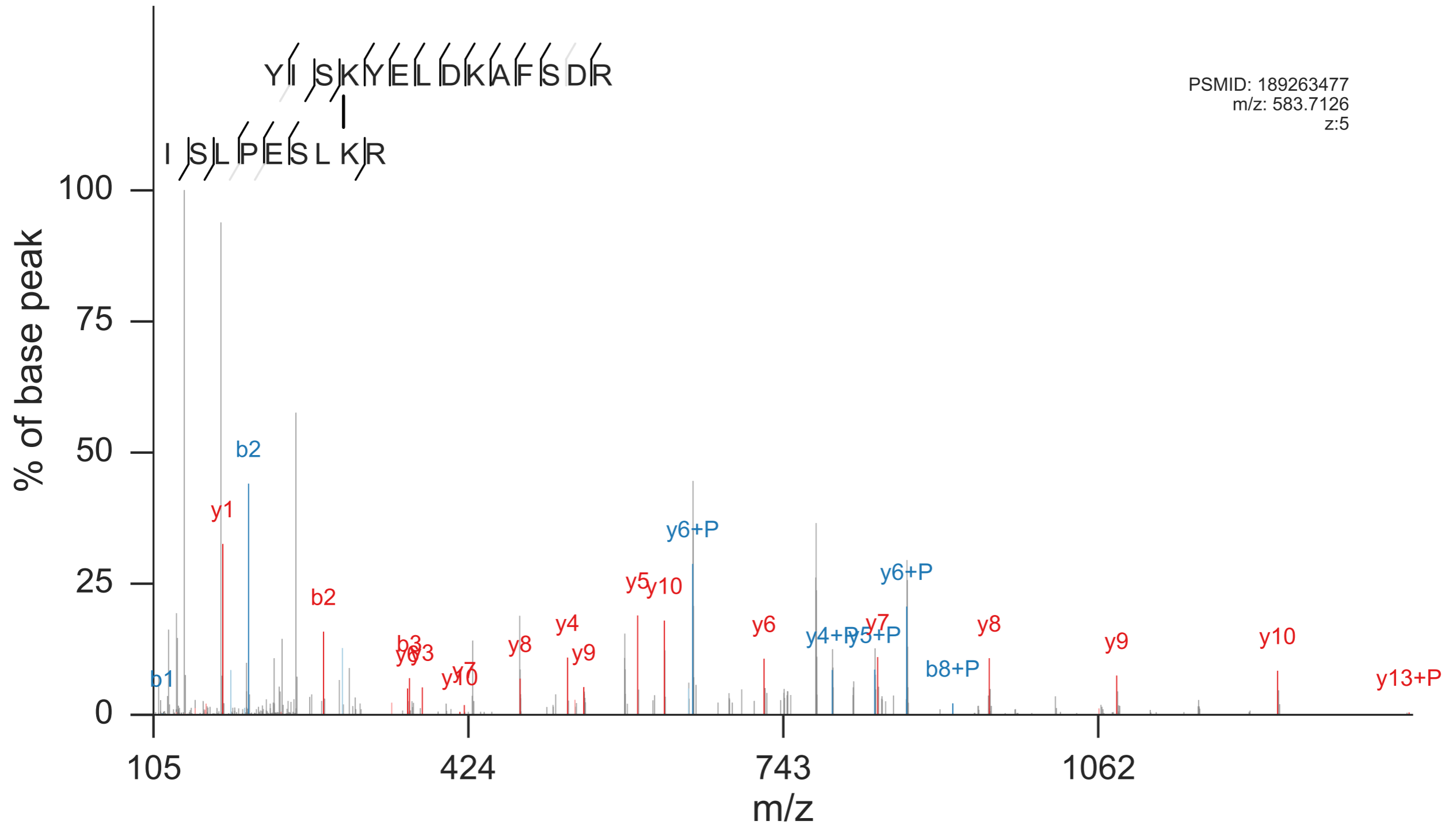

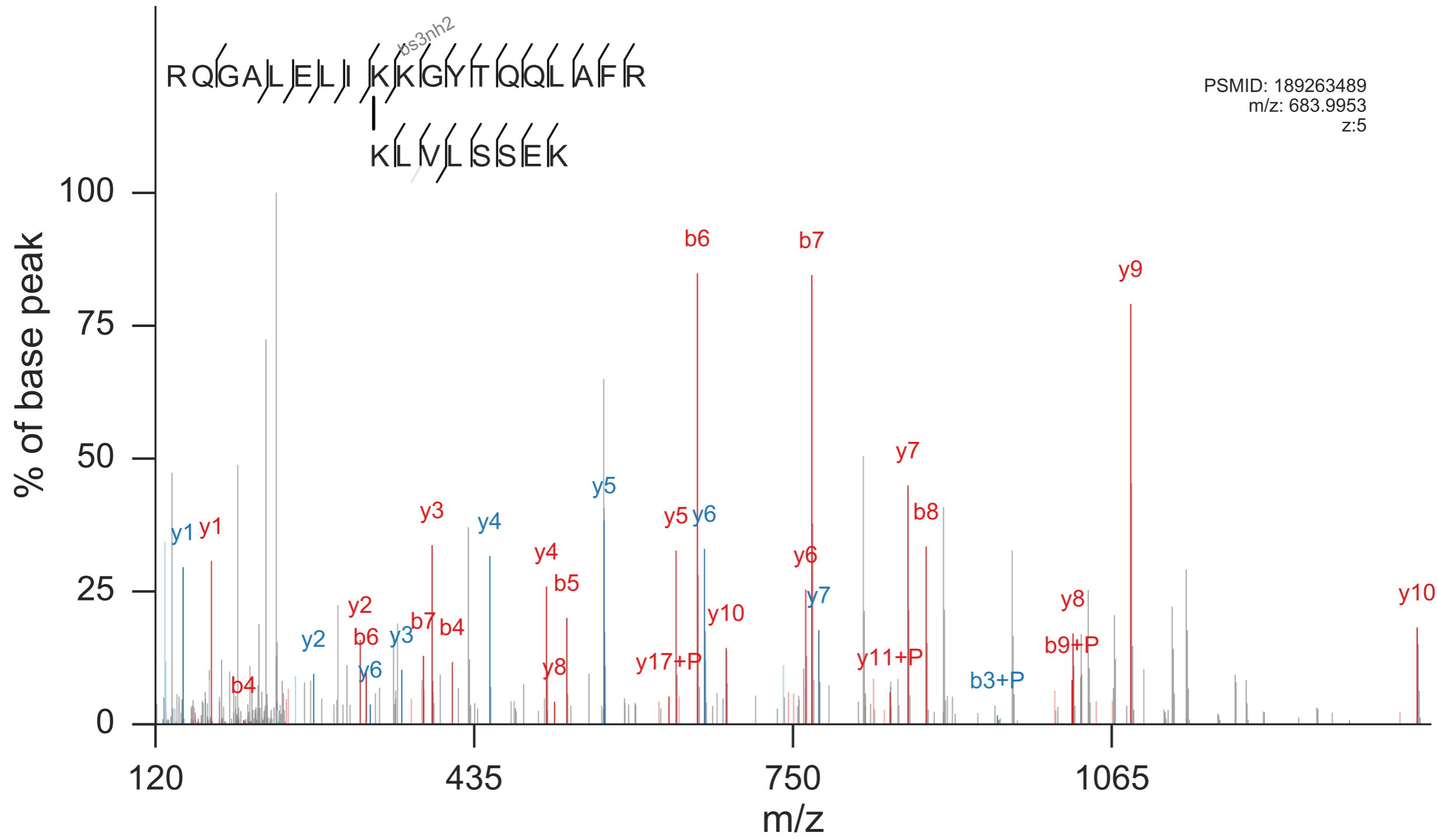

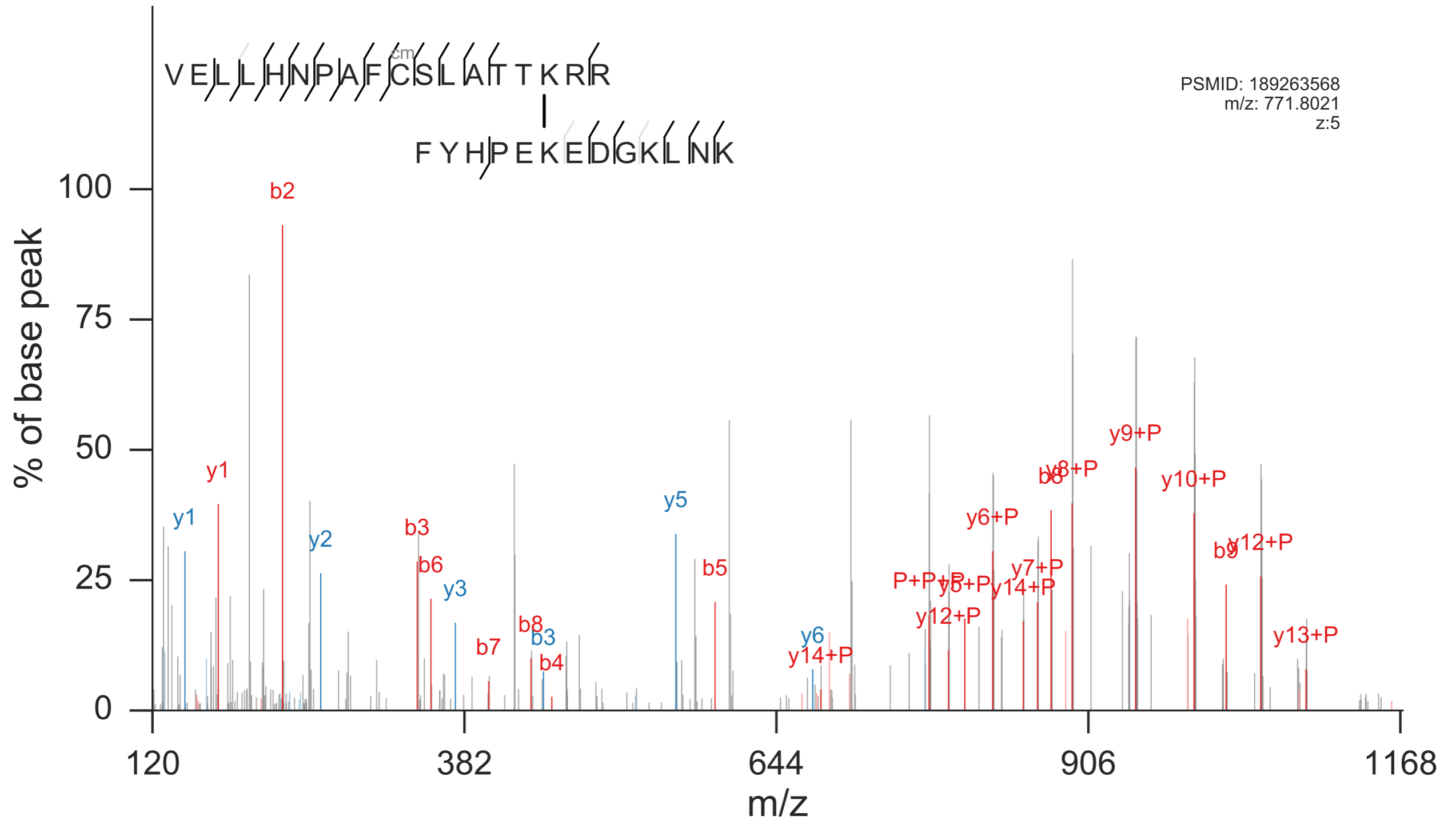

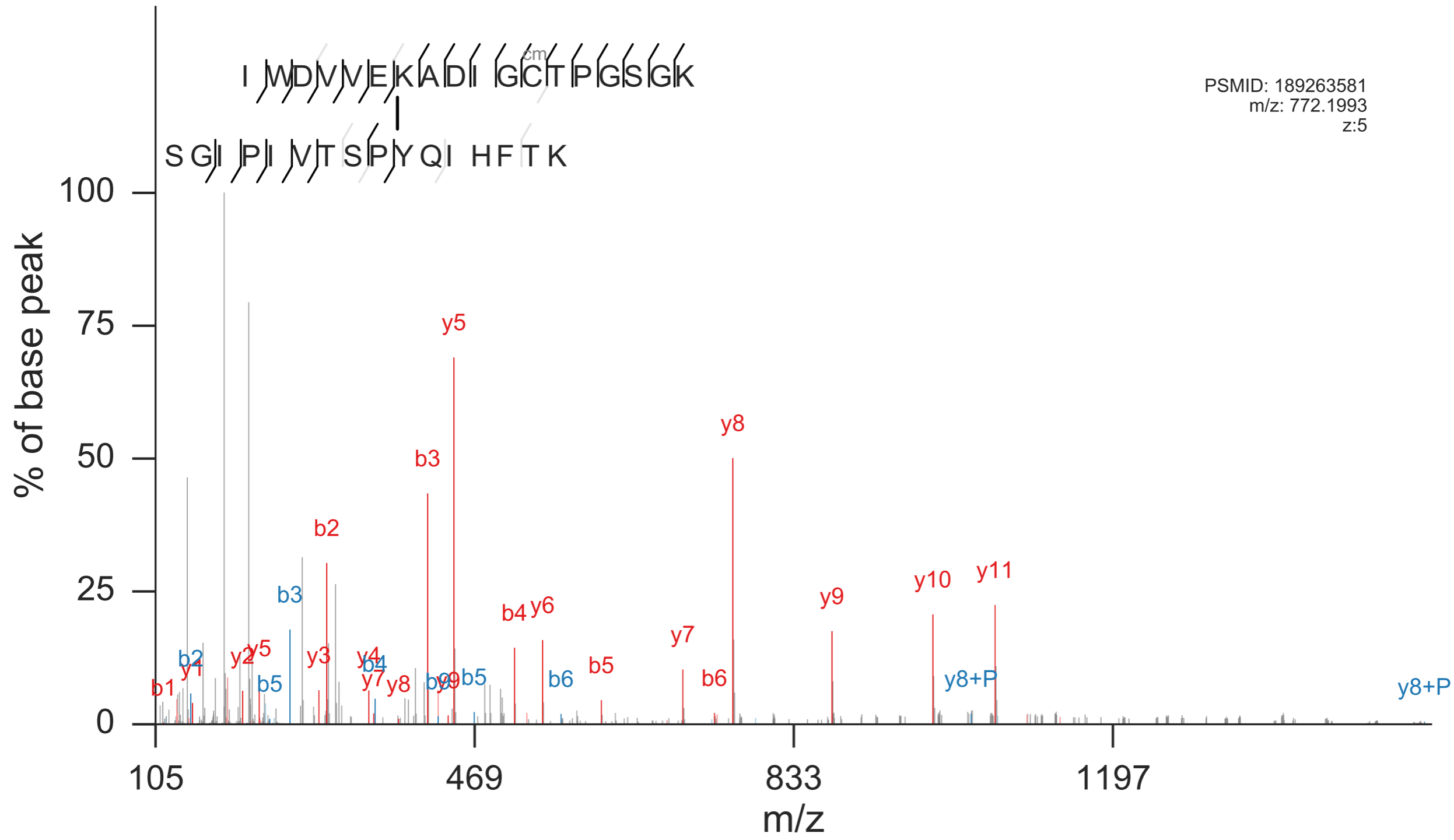

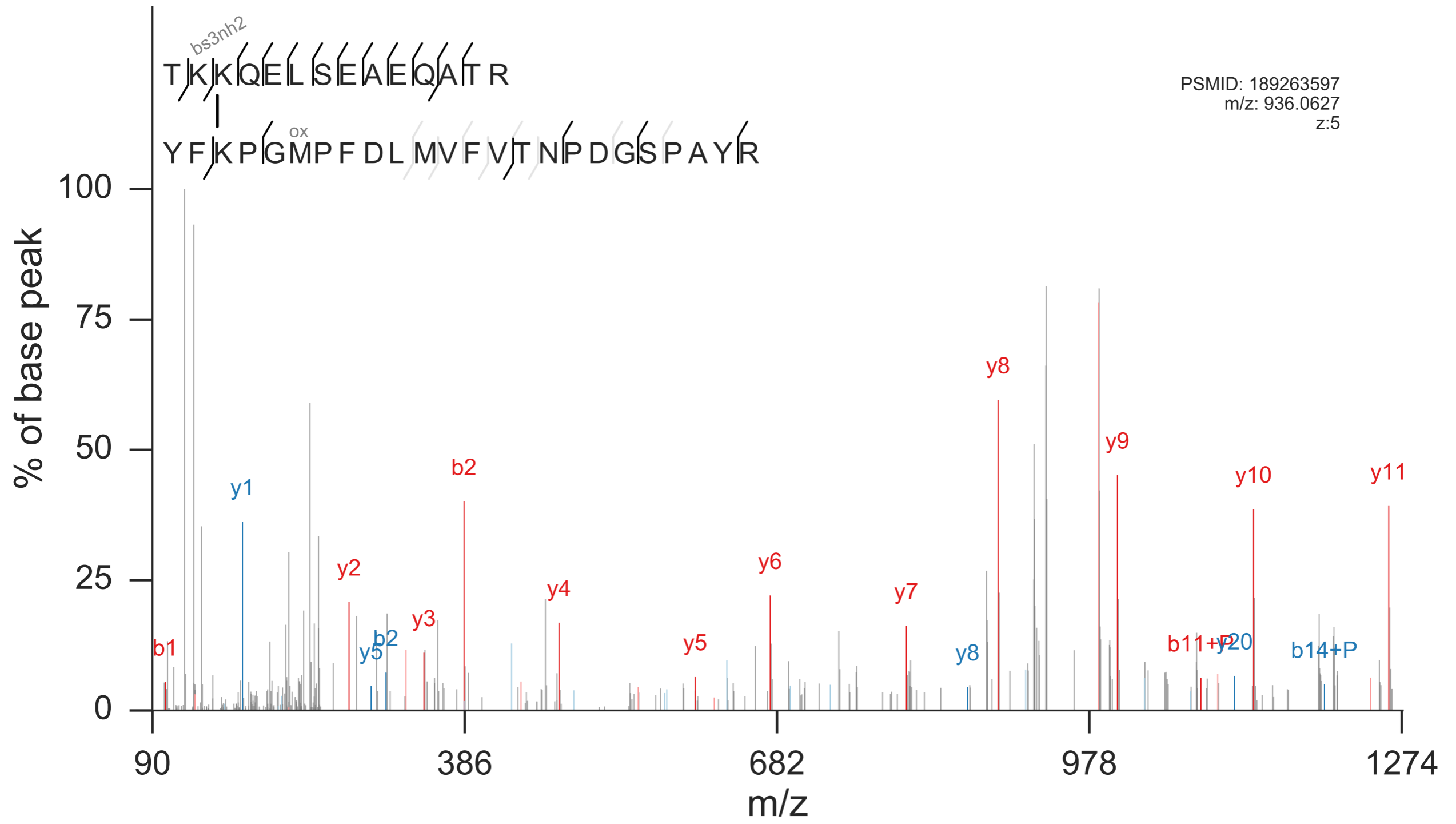

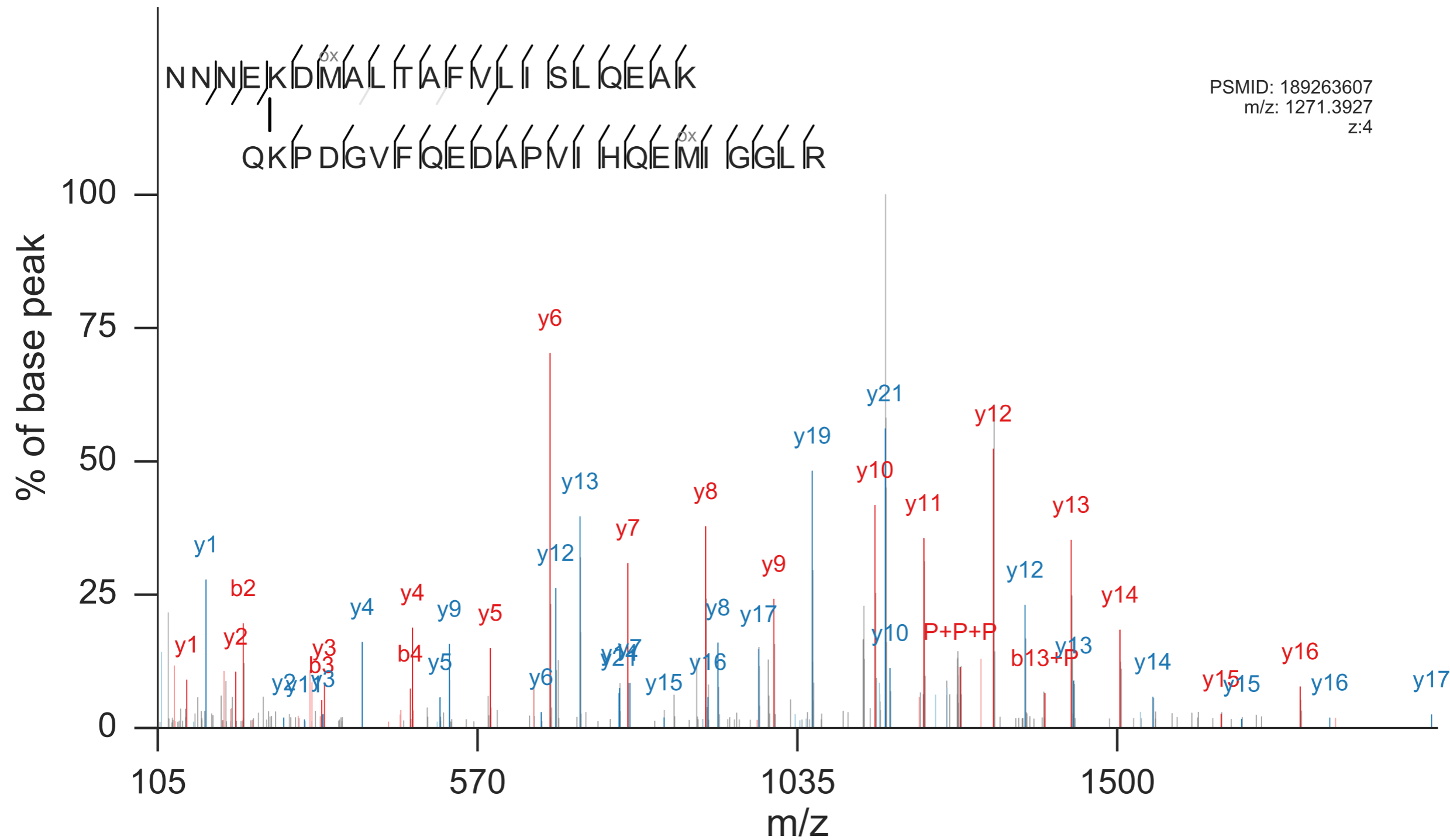

Supplement: Supplementary file 6 [file wellcomeopenres-1-10667-s0005.tgz › f238e93d-cce7-47fb-9402-9d9a2e4743eb.pdf]
