# Supplementary material for: Quantitative cross-linking/mass spectrometry reveals subtle protein conformational changes
Source: Wellcome Open Res. 2016 Nov 15;1:5. [Version 1] doi: 10.12688/wellcomeopenres.9896.1 (PMC5140025; doi:10.12688/wellcomeopenres.9896.1)
Supplement: Peer review history

# Peer review: Referee response for version 1

**DOI:** 10.21956/wellcomeopenres.10667.r17979

**Published:** 2016-12-19

**Authors:** Argyris Politis, Andy M. Lau

**Affiliations:** 1 Department of Chemistry, King's College London, London, UK

**Title and abstract:** In line with the article

**Content**: In this manuscript, Rappsilber and co-workers detail a comprehensive and robust chemical crosslinking MS method to sample the conformational dynamics of the complement protein C3 and one of its activated products, C3b. The use of crosslinking MS is a powerful tool for elucidating the solution state dynamics of proteins in their near-native environments. The study features the use of non-deuterated and deuterated crosslinking reagents which are used as molecular reporters of the accessibility distribution of crosslinked residues specific to the C3 and C3b structures. The data is of high quality and presented in an appealing and clear format. The structural transition of C3 to C3b is accompanied by large conformational changes (as seen with the crystal structures of C3 and C3b), and is a suitable and relatable model system to other researchers in structural biology who may deal with systems that exhibit similar dynamics.

 The authors describe a limitation with chemical crosslinking MS: “Cross-linking is renowned for low reproducibility”, which appears a bit of an unclear statement. While labelled residue pairs reported from experimental replicas may not be 100% identical, the strategy should be representative of the state-specific labelling status of the protein, and not dramatically influenced by downstream or other processing procedures.

 Although the geometry and accessibility of crosslinked residue pairs are important to consider when evaluating crosslinks, the authors have highlighted a limitation with projection of crosslinks onto crystal structures. The nature of models generated from X-ray crystallography represent a stable solid-state conformation which is often a poor indicator of the solution-state dynamics. Side chain orientations of crystallised proteins may be influenced depending on crystal packing and model bias introduced during refinement of the structure. A quick check of the C3 and C3b crystal structures (PDB ID 2A73 and 2I07) reveal them to be at 3.3 and 4.0 Å resolution, and as such it may be a stretch to use a theoretical crosslinking limit of 27.4 Å to demonstrate subtle conformational changes with a model derived from low resolution X-ray crystallography. It may be more reliable to use a lower-resolution distance limit such as 30 Å and measure from backbone atoms (which are more reliably fitted from electron density data). If considering the solvent accessibility and flexibility of specific residues, it may be appropriate to complement such data with b-factors deposited with the crystal structures, or RMSF values derived from molecular dynamic simulations.

 In the Supplementary Figure S2, the crosslinked residue pairs K1346-K1359 in panel (a) have been described as becoming “less favourable for cross-linking” in the C3b model. Could the authors describe what they mean by “less favourable”, and perhaps annotate the figure with the measured Euclidean distances for these examples? Without accompanying flexibility data, it is difficult to support and substantiate the probability of a crosslink being formed between rotamers of side chains found in the crystal structure. In panel (b) of the figure, it is difficult to see how the authors have deduced that the “flexibility of cross-linked residues reduce”.

**Minor points of revision:**

1. Figures 3 and 4 are referred to before Figure 2.
2. The legend of Figure 5. Lacks the (E) subpanel.
3. The figure legend of Supplementary Figure 1 (Supplemental Material 2) appears multiple times in the figure and overlaps with panels C and D.

We have read this submission. We believe that we have an appropriate level of expertise to confirm that it is of an acceptable scientific standard.

# Peer review: Referee response for version 1

**DOI:** 10.21956/wellcomeopenres.10667.r18163

**Published:** 2016-12-01

**Authors:** Perdita E. Barran, Rebecca Beveridge

**Affiliations:** 1 School of Chemistry, University of Manchester, Manchester, UK; 2 Research Institute of Molecular Pathology, Campus-Vienna-Biocenter 1, Vienna, Austria

**Title and Abstract**: - All in keeping with the article.

**Article content**:

 In this manuscript the authors describe an effective approach for quantitative cross-linking/ mass spectrometry (QCLMS). The method was previously used to investigate properties of two distinct conformations of complement C3(H2O) protein which arise due to proteolytic cleavage. Here the protocol and data analysis strategies are described in greater detail than in earlier publications, which is of benefit to researchers considering to implement the quantitative aspect in their own cross-linking/MS investigations. An important part of this workflow is the ‘label-swap’ aspect, whereby the deuterated cross-linker is used first on one sample, and then on the other. Combining this data then negates any effect of the cross-linker label, since it has been used with both samples. The approach appears to be robust, and it is certainly demonstrated on a very suitable protein.

**Conclusions and Data**:

 A slight disadvantage of this approach (which also applies to all other quantitative workflows) is that some cross-links of low abundance will not be identified. The non-crosslinked peptides from the digests of C3 and C3b will have the same m/z value, whilst the cross-linked peptides will have a different m/z because of the isotopic XL-reagent. Therefore, the relative signal intensity for the XL peptides in the mixed sample is 50% compared to non-mixed. Whilst this doesn’t affect the study in question which only considers the quantifiable cross-linked peptides, it would be useful to mention this limitation and to point out that if the unmixed samples are initially analysed more cross-links could be identified, which may help in further studies. If a given lab uses the D0-D4 x-linker as standard then this point is moot. But that point should be mentioned here.

 It would also be interesting to know how the researchers verified that they are mixing a 1:1 ratio of digested peptides from the different samples. On the gel, there is less monomeric protein in the C3b sample, because more multimers are formed. Is this taken into consideration?

 Is it possible to show if the ‘deuteration’ of the cross-linker affects the extent of cross-linking? If the C3-light and C3-heavy are analysed together it could be seen if more cross-links are found with one than with the other. This would support the need for label swaps.

 For the SCX ‘enrichment’ step… it would be interesting to know whether the peptides are enriched with respect to non-cross-linked, or just separated from each other. A comparison of PSMs of linear peptides and cross-linked peptides would be beneficial.

 A technical point is that the figures are not in the correct order. Figure 2 is not mentioned until after Figure 4 in the text.

 The authors should mention HDX-MS and other MS based foot-printing approaches (for example FPOP) as a competing/ complementary technologies. The use of HDX-MS along with chemical crosslinking could be highly informative here. The time scale effects mentioned in conclusion can be better explored with the use of robotics and/ or photo-induced labelling and this may be a good direction for further study.

 In general the article requires significant editing; there are many instances where definite articles are missing, words are not pluralised, and words are not spelt correctly. We also think that the article suffers a little from too much opinion regarding the technology for example:

 Page 1 second paragraph, Cross-linking is renowned for low reproducibility. What does this statement mean? Which part of the complex methodology is not reproducible?

We have read this submission. We believe that we have an appropriate level of expertise to confirm that it is of an acceptable scientific standard.

# Peer review: Referee response for version 1

**DOI:** 10.21956/wellcomeopenres.10667.r17585

**Published:** 2016-11-28

**Authors:** Alexander Leitner

**Affiliations:** 1 ETH Zurich, Department of Biology, Institute of Molecular Systems Biology, Zurich, Switzerland

Rappsilber and co-workers provide a detailed description of their quantitative cross-linking/mass spectrometry (QCLMS) workflow as applied to the complement protein C3 and its truncated form C3b. This article complements earlier reports from the group that focused on the application of the method to more extended biological problems. In the present work, the emphasis is on method design and how QCLMS data can give insight into conformational changes within proteins.

 QCLMS is an emerging technique in structural proteomics / structural mass spectrometry, and there have been only few applications so far. Therefore, only limited knowledge exists concerning how data emerging from such experiments should be interpreted. As Chen *et al,.* rightly point out, abundance changes of cross-linked peptides can be caused by different types of changes in the protein structure besides a major conformational rearrangement, and the C3 system actually provides several different examples that point to relatively small changes in the immediate vicinity of cross-linked residues (steric clashes, changes in solvent accessibility etc.). Therefore, the results presented here add valuable insight into what QCLMS can tell us (or not tell us) about protein structures, and these results can be extended to other studies.

 In addition, the article provides a more detailed description of the methods employed by the Rappsilber group in a concise format, including experimental design (label-swap experiment) and data analysis strategy. All results are properly documented and the raw mass spectrometry data have been deposited in the PRIDE repository.

 I have one comment regarding a somewhat vague statement in the introduction: "Cross-linking is renowned for low reproducibility." This may be an unconcious feeling that some of us working in this area have. Is there any reference that the authors can cite that supports this statement? Indeed, in view of the results presented in this work, the reproducibility of QCLMS, or CLMS in general, does not appear to be such a major concern, but rather seems to be affected by parameters not directly connected to the cross-linking procedure, such as undersampling in the mass spectrometer.

 Two minor technical points:

1. Figures 3 and 4 are referred to ahead of Figure 2, so the authors should consider renumbering the figures.
2. Starting on page 7, the authors refer to individual domains of the C3 protein. It would be helpful to add a respective illustration in one of the figures for reference.

I have read this submission. I believe that I have an appropriate level of expertise to confirm that it is of an acceptable scientific standard.
